# Brazilian Angiology and Vascular Surgery Society Guidelines for the treatment of extracranial cerebrovascular disease

**DOI:** 10.1590/1677-5449.202300942

**Published:** 2024-05-31

**Authors:** Arno von Buettner Ristow, Bernardo Massière, Guilherme Vieira Meirelles, Ivan Benaduce Casella, Marcia Maria Morales, Ricardo Cesar Rocha Moreira, Ricardo Jayme Procópio, Tércio Ferreira Oliveira, Walter Jr. Boim de Araujo, Edwaldo Edner Joviliano, Júlio Cesar Peclat de Oliveira

**Affiliations:** 1 Pontifícia Universidade Católica do Rio de Janeiro – PUC-RIO, Disciplina de Cirurgia Vascular e Endovascular, Rio de Janeiro, RJ, Brasil.; 2 Sociedade Brasileira de Angiologia e de Cirurgia Vascular – SBACV-RJ, Rio de Janeiro, RJ, Brasil.; 3 Sociedade Brasileira de Angiologia e de Cirurgia Vascular – SBACV-SP, São Paulo, SP, Brasil.; 4 Universidade Estadual de Campinas – UNICAMP, Hospital das Clínicas, Disciplina de Cirurgia do Trauma, Campinas, SP, Brasil.; 5 Universidade de São Paulo – USP, Faculdade de Medicina, São Paulo, SP, Brasil.; 6 Associação Portuguesa de Beneficência de São José do Rio Preto, Serviço de Cirurgia Vascular, São José do Rio Preto, SP, Brasil.; 7 Sociedade Brasileira de Angiologia e de Cirurgia Vascular – SBACV-PR, Curitiba, PR, Brasil.; 8 Pontifícia Universidade Católica do Paraná – PUC-PR, Hospital Cajurú, Serviço de Cirurgia Vascular, Curitiba, PR, Brasil.; 9 Universidade Federal de Minas Gerais – UFMG, Hospital das Clínicas, Setor de Cirurgia Endovascular, Belo Horizonte, MG, Brasil.; 10 Universidade Federal de Minas Gerais – UFMG, Faculdade de Medicina, Belo Horizonte, MG, Brasil.; 11 Sociedade Brasileira de Angiologia e de Cirurgia Vascular – SBACV-MG, Belo Horizonte, MG, Brasil.; 12 Sociedade Brasileira de Angiologia e de Cirurgia Vascular – SBACV-SE, Aracajú, SE, Brasil.; 13 Universidade de São Paulo – USP, Faculdade de Medicina de Ribeirão Preto – FMRP, Ribeirão Preto, SP, Brasil.; 14 Universidade Federal do Paraná – UFPR, Hospital das Clínicas – HC, Curitiba, PR, Brasil.; 15 Universidade Federal do Estado do Rio de Janeiro – UNIRIO, Departamento de Cirurgia, Rio de Janeiro, RJ, Brasil.

**Keywords:** carotid stenosis, stroke, guidelines, carotid endarterectomy, carotid artery diseases, carotid artery injuries

## Abstract

Extracranial cerebrovascular disease has been the subject of intense research throughout the world, and is of paramount importance for vascular surgeons. This guideline, written by the Brazilian Society of Angiology and Vascular Surgery (SBACV), supersedes the 2015 guideline. Non-atherosclerotic carotid artery diseases were not included in this document. The purpose of this guideline is to bring together the most robust evidence in this area in order to help specialists in the treatment decision-making process. The AGREE II methodology and the European Society of Cardiology system were used for recommendations and levels of evidence. The recommendations were graded from I to III, and levels of evidence were classified as A, B, or C. This guideline is divided into 11 chapters dealing with the various aspects of extracranial cerebrovascular disease: diagnosis, treatments and complications, based on up-to-date knowledge and the recommendations proposed by SBACV.

## INTRODUCTION AND METHODS

This guideline on the treatment of extracranial cerebrovascular disease patients was developed by the Brazilian Society of Angiology and Vascular Surgery (SBACV) to supersede the 2015 guideline. Non-atherosclerotic carotid artery diseases (arteritis, dissections, fibromuscular dysplasia, dissections, and aneurysms) were not included in this document. The purpose of this guideline is to bring together the most robust evidence in this area in order to help specialists in the treatment decision-making process. The authors adopted the AGREE II methodology in order to write and assess the guidelines.^1,2^ The working group in charge of writing the guideline consisted of full members of SBACV widely recognized for their expertise in treating carotid artery disease.

The working group held remote meetings to assess how the document progressed. Search strategies to select scientific papers involved the use of the MEDLINE platform until January 2023. Only peer-reviewed publications were included, based on the principle of the evidence pyramid. Randomized controlled trials and meta-analyses of randomized controlled trials were placed at the top of the pyramid, followed by randomized individual trials or large non-randomized trials, meta-analyses of non-randomized small studies, observational studies, case series, and retrospective studies. Expert opinions were placed at the bottom of the pyramid, while case reports were excluded.

The European Society of Cardiology system was used to develop recommendations and levels of evidence. Classes were graded from I to III, where I is the strongest.^3^ Levels of evidence were categorized as A, B, or C, with A the highest. Each member of the working group was assigned to specific areas and charged with developing recommendations, which were subsequently reviewed by the authors as a group and classified according to level of evidence.

Charts 1 and 2, below, summarize the classes and levels of evidence employed in this guideline. The goal is to help guide specialists regarding the diagnosis and treatment of extracranial cerebrovascular disease for which there are undeniable levels of recommendation and evidence in light of current scientific knowledge. In many areas, these goals are yet to be achieved, and might never be. When relevant, these subjects were also addressed, and their current situation discussed.

**Chart 1:** Classes of recommendation.

**Class**	**Indications and definition**	**Recommendation**
I	General agreement that the procedure or treatment is useful and effective	Is recommended
II	Conditions for which there is divergence of opinion about the efficacy or usefulness of the given procedure or treatment	
IIa	Weight of opinion is in favor of indicating the procedure or treatment	Should be considered
IIb	Usefulness or efficacy of procedure or treatment is less well established by evidence and opinion	May be considered
III	General agreement that the given procedure or treatment is not useful or effective, and in some cases may generate risks	Is not recommended, should not be done

**Chart 2:** Levels of evidence.

**Level**	**Definition**
A	Data derived from multiple high-quality randomized clinical trials
B	Data derived from a single high-quality randomized clinical trial or large non-randomized studies
C	Data derived from case series and/or records, consensus and/or opinion of experts

## CLINICAL INTRODUCTION

A stroke is a sudden-onset neurological syndrome caused by the interruption of blood flow to a specific region of the central nervous system, manifesting as sudden focal neurological dysfunction lasting longer than 24 hours. The stroke can be caused by several mechanisms, in isolation or in tandem. The Trial of ORG 10172 in Acute Stroke Treatment (TOAST) classification defined five types of stroke: large-artery atherosclerosis, small-vessel occlusion (lacunar), cardioembolism, stroke of undetermined etiology, and stroke of other determined etiology (infrequent).^4^

A transient ischemic attack (TIA) is defined as an episode of focal brain, retinal, or spinal cord dysfunction lasting less than 24 hours, of non-traumatic etiology.^5^ In large-artery atherosclerosis, ischemia may be caused by the embolization of atherosclerotic plaque or occlusion of the vessel with hemodynamic effect. The most frequent sites of atherosclerosis are the carotid bifurcation, the aorta, and vertebral arteries.

In cardioembolism, the factors may be high-risk, such as the presence of intracardiac thrombus, atrial fibrillation (AF), and rheumatic heart valve disease; or low-risk, such as patent foramen ovale, apical akinesia, and hypokinetic segment of the left ventricle. In Brazil, Chagas disease should be considered as a possible cause of cardioembolic stroke in cases of suggestive epidemiology. Lacunar infarction results from the occlusion of small arteries stemming from middle cerebral, vertebral or basilar arteries, or the branches forming the circle of Willis.

Neurological examination should be objective, considering the need to determine the best course of action as quickly as possible. Two scales have been used in recently published guidelines: the National Institute of Health Stroke Scale, more complete and complex, is used to measure neurological deficit, assess a prognosis, determine the topography of the injury, and facilitate communication between health care professionals (Chart 3); and the shorter and faster modified Rankin Scale (mRs) (Chart 4).^6-8^

**Chart 3:** National Institutes of Health Stroke Scale – NIHSS.^5^

	**Scale Definition**
**1a: Level of Consciousness**	0 = Alert; keenly responsive.
The investigator must choose a response if a full evaluation is prevented by such obstacles as an endotracheal tube, language barrier, orotracheal trauma/bandages. A 3 is scored only if the patient makes no movement (other than reflexive posturing) in response to noxious stimulation.	1 = Not alert; but arousable by minor stimulation to obey, answer, or respond.
	2 = Not alert; requires repeated stimulation to attend, or is obtunded and requires strong or painful stimulation to make movements (not stereotyped).
	3 = Responds only with reflex motor or autonomic effects, or totally unresponsive, flaccid, and areflexic.
**1b. Level of consciousness questions**	0 = Answers both questions correctly.
The patient is asked the month and his/her age.	1 = Answers one question correctly.
	2 = Answers neither question correctly.
**1c. Level of consciousness commands**	0 = Performs both tasks correctly.
The patient is asked to open and close the eyes and then to grip and release the non-paretic hand.	1 = Performs one task correctly.
	2 = Performs neither task correctly.
**2. Ocular motility (Best Gaze)**	0 = Normal.
Only horizontal eye movements will be tested.	1 = Partial gaze palsy.
	2 = Forced deviation.
**3. Visual fields**	0 = Normal.
	1 = Partial hemianopia.
	2 = Complete hemianopia.
	3 = Cortical blindness
**4. Facial Palsy**	0 = Normal.
	1 = Minor paralysis
	2 = Paresis/paralysis of lower face.
	3 = Paresis/paralysis of upper and lower face.
**5. Motor: upper limb**	0 = No drift.
	1 = Drift, but does not hit bed.
	2 = Some effort against gravity; but limb drifts down to bed.
	3 = No effort against gravity; but any motion is considered.
	4 = No movement.
**6. Motor: lower limb**	0 = No drift.
	1 = Drift, but does not hit bed.
	2 = Some effort against gravity; but limb drifts down to bed.
	3 = No effort against gravity; but any motion is considered.
	4 = No movement.
**7. Limb Ataxia**	0 = No ataxia.
	1 = Ataxia present in one upper or lower limb.
	2 = Ataxia present in one upper or lower limb.
**8. Pain sensitivity**	0 = Normal.
	1 = Unilateral deficit, but acknowledges stimulus.
	2 = Patient does not acknowledge stimulus; coma or bilateral deficit.
**9. Language**	0 = Normal.
	1 = Mild-to-moderate aphasia (intelligible).
	2 = Severe aphasia.
	3 = Mute, global aphasia, and coma.
**10. Dysarthria**	0 = Normal.
	1 = Mild-to-moderate dysarthria.
	2 = Severe dysarthria, unintelligible or mute.
	X = Intubated.
**11. Extinction and Inattention**	0 = Normal.
	1 = Inattention or extinction in one of the sensory modalities.
	2 = Inattention in more than one of the sensory modalities.

**Chart 4:** Modified Rankin Scale.^6,7^

**Description of symptoms**	**Scale Definition**
Asymptomatic.	0
No significant disability. Able to carry out all usual activities.	1
Slight disability. Able to look after own affairs without assistance, but unable to carry out all previous activities.	2
Moderate disability. Requires some help, but able to walk unassisted.	3
Moderately severe disability. Unable to attend to own bodily needs without assistance, and unable to walk unassisted.	4
Severe disability. Requires constant nursing care and attention, bedridden, incontinent.	5
Dead.	6

## DIAGNOSTIC IMAGING METHODS

Imaging in carotid stenosis patients is based on three main parameters—identifying the anatomical location of the obstruction, determining the degree of lumen reduction, and establishing plaque characteristics. Major stenoses and heterogeneous, large and hypoechogenic plaques are associated with higher risk of stroke.^9^

Color Doppler ultrasound (CDU) is the initial imaging modality of choice for patients suspected of carotid stenosis.^10^ The method is widely available, low cost, may be performed at bedside, does not require anesthesia, and does not involve ionizing radiation or the use of intravenous contrast. In addition, it is accurate and reliable when performed by experienced examiners and according to criteria established in the literature: 85 to 92% sensitivity and 84% specificity.^9,11^

Initially, carotids should be studied using B-mode scanning. When plaque is detected, the rate of lumen reduction and residual lumen should be measured in cross-section.^12^ Next, one should identify vulnerable or unstable plaques, i.e., those at higher risk of cerebrovascular events. This requires assessing plaque characteristics.^13^ It is recommended that the location, extension, echogenicity, texture, surface, presence of movable components, and anechoic areas next to the fibrotic capsule be studied and described when finding significant plaques, i.e., those with more than 50% stenosis.^12^

Plaque echogenicity is divided into three types. Darker, bloodlike plaques are known as hypoechogenic or echolucent, and consist of lipids, blood, or recent thrombi. Isoechogenic plaque is closer in appearance to muscle, and includes fibrous content. Hyperechogenic plaque is lighter than the adjacent muscle, and consists of denser fibrous tissue. When plaques become calcified, this creates acoustic shadowing, generating a dark band posterior to the plaque as the calcium prevents the propagation of ultrasound waves. Plaques are also classified as homogeneous when they have uniform echo levels or heterogeneous when featuring a mixture of echo levels.^14^ Plaques may be classified into five different types according to echogenicity and association with events:^15^

Type 1 - echolucent and homogeneous, with or without fibrous cap—considered of moderate risk for events.
Type 2 - predominantly echolucent, with echogenic areas smaller than 50% of total plaque area—a more unstable type of plaque, and therefore riskier.
Type 3 - predominantly echogenic, with hyperechogenic area greater than 50% of total plaque area—risk between types 1 and 2.
Type 4 - uniformly echogenic.
Type 5 - calcified, with acoustic shadow.

The last two are considered stable and, therefore, lower risk.

Regarding the surface, the plaque is categorized as smooth if the irregularities have less than 0.4 mm in depth, irregular from 0.4 to 2.0 mm in depth, and ulcerated when featuring an excavation greater than 2.0 mm in depth. However, this is not enough. Examiners should compare the weaker echoes in concavity in the plaque with the echoes obtained from segments adjacent to the plaque surface. This criterion has 85.7% sensitivity and 81.3% specificity.^16^

However, grading stenosis requires acurate assessment of the flow velocity in the carotid arteries. It is crucial that flow velocity be measured using rigorous technical criteria: small sample volume, positioned at the center of the vessel, parallel to the direction of the flow and at an angle between 45 and 60º—ideally ≤ 60º.^12^ The velocity criteria most frequently used in clinical practice can be found in Chart 5.^4,9^

**Chart 5:** End-diastolic velocity criteria for grading carotid stenosis.^12,17^

**% NASCET** **a**	**PSV** **b** **internal carotid**	**EDV** **c** **internal carotid**	**Ratio Internal PSV /Common PSV**	**St. Mary’s Internal PSV/Common EDV**
< 50%	< 125 or < 140	< 40	< 2	< 8
50-59%	> 125 or > 140	40 to 69	2-3.1	8-10
60-69%		70 to 100	3.2-4	11-13
70-79%	> 230	> 100	> 4	14-21
80-89%		> 140	From 4 to 5	22-19
> 90	> 400		> 5	> 30
Subocclusion	Variable		Variable	Variable
Occlusion	No flow		N.A.	N.A.

a Percentage stenosis by NASCET criteria;

b peak systolic velocity;

c end-diastolic velocity. All velocities in cm/s.

Peak systolic velocity (PSV) has higher accuracy in grading stenosis, and is the primary criteria used. Other parameters are useful to verify and stratify the narrowing estimated by PSV.

For stenoses greater than 50%, a higher PSV cutoff value (around 140 cm/s rather than 125 cm/s) tends to be adopted in order to improve accuracy.^18,19^ In addition, Morales et al.^20^ have shown that using PSV to assess stenosis in patients with plaques associated with major acoustic shadowing has low sensitivity and mismatches with computed tomography angiography (CTA).

Digital subtraction angiography has higher sensitivity, specificity and accuracy in detecting carotid stenosis greater than 95% stenosis.^21^ However, its invasive character and the risk of ischemic stroke, as confirmed by Asymptomatic Carotid Artery Study (ACAS), combined with the development of noninvasive methods, has led it to become less indicated.^21,22^

Currently, angiography is reserved for patients with suspected carotid stenosis not resolved by at least two noninvasive imaging methods. This occurs when diagnostic methods lead to diverging results for patients with contraindication for a given method—such as claustrophobia for magnetic resonance imaging (MRI)—or have major technical limitations, such as excess calcification preventing the propagation of sound waves or injuries beyond the mandibular angle. In addition, it should only be offered to patients who would likely benefit from revascularization if significant stenosis is detected.

### Recommendation 1

Digital subtraction angiography is limited to a preoperative examination for patients presenting mismatches on non-invasive imaging (I/B).^5,12^

CTA is one of the imaging modalities most frequently used by Brazilian vascular surgeons. Widely available, the method enables fast imaging as well as the visualization of various planes and projections, and multiplanar and three-dimensional reconstruction. It allows imaging of both extracranial and intracranial circulation, including the aortic arch and supra-aortic trunks (SATs). Lumen reduction measurements are accurate and performed analogous to the angiographic method. Its sensitivity for severe stenosis (70 to 99% lumen reduction) and occlusion is 85% and 97%, and its specificity, 93% and 99%, respectively. It cannot accurately assess plaque morphology and characteristics and provides low resolution for highly calcified plaques.^20^ In addition, it requires iodinated contrast and ionizing radiation.^23^ Therefore, it is usually performed to verify CDU findings. It can also determine the type of the aortic arch, the path, and the tortuosity of the artery to be treated, significant factors for carotid angioplasty candidates. It also allows physicians to examine arterial segment inaccessible to ultrasound, such as intracranial circulation.

Magnetic resonance angiography (MRA) is a noninvasive imaging method that does not require iodinated contrast or ionizing radiation. It enables imaging intrathoracic, cervical, and intracranial vessels from various projections and vascular reconstructions from the data it provides. It can also detect injuries to cerebral parenchyma. The sensitivity and specificity for stenosis greater than 70% is the same as for CDU.^24^ However, it tends to overestimate moderate injuries and subocclusions. On the other hand, it can assess plaque characteristics in terms of both content and surface. Its disadvantages include the long time required to obtain images involving the aortic arch, cervical circulation and intracranial circulation (which takes seconds for CTA), as well as the fact that it cannot be used in patients with implanted devices, such as defibrillators and pacemakers. Some, but not all types of gadolinium (paramagnetic contrast agent used in MRA) have been associated with nephrogenic systemic fibrosis in patients with creatinine clearance below 30 mL/min.^23^ Indications for MRA are similar to those for CTA—verifying CDU findings and surveying the aortic arch, SAT, and intracranial circulation.

There is no consensus on how to proceed with asymptomatic patients with internal carotid stenosis greater than 50% found in CDU imaging.^5^ In general, when patients are recommended for intervention, injuries detected should be confirmed by another imaging modality. The most frequently used modalities are CTA and MRA.^5^ Since it allows imaging of the aortic arch, supra aortic trunks (SAT), and branches of intracranial arteries and cerebral parenchyma, they help select individuals who, despite being asymptomatic, are at higher risk of ischemic stroke or TIA, as discussed elsewhere in this guideline.

Furthermore, they allow us to assess anatomical criteria, such as height of the carotid bifurcation, calcification, and tortuosity of the aortic arch and internal carotids. Combined with the patient's clinical history, these anatomic factors are crucial for choosing the best intervention technique—conventional surgery, transcarotid or transfemoral carotid angioplasty. Alternatively, patients who are candidates for carotid endarterectomy (CEA) may undergo an additional CDU, with a different examiner, to verify the presence and degree of the stenosis. When tests for patients with indication for intervention are mismatched, a third examination may be performed. This may be one of the few indications for digital subtraction angiography at the moment.^5^

Imaging tests are as accurate for asymptomatic individuals as they are for symptomatic patients. However, invasive treatment for acute stroke has become increasingly frequent.^25^ Patients with acute neurological deficit secondary to ischemic stroke benefit from intravenous thrombolysis when administered up to 4.5 hours after symptom onset.^8^ Ischemic stroke patients from 4.5 to 24 hours after symptom onset, however, may be candidates for mechanical thrombectomy. This requires an assessment of the extension of ischemia as well as the presence of occlusion of the internal carotid, the middle cerebral artery (MCA) and its branches or the anterior cerebral artery. Ruling out hemorrhage is essential for both interventions.^26^ Thus, suspected stroke patients should immediately undergo brain imaging examination.^27^

Non-contrast computed tomography (CT) of the brain is the most widely available imaging technique in Brazil. It has low sensitivity for detecting early ischemia—around 64% in the first 6 hours. However, it safely rules out hemorrhages. Therefore, it is enough for the indication for thrombolysis.^28^ Today, most acute cerebral ischemia patients undergo head CT and CTA. In addition to allowing physicians to survey both intracranial and extracranial cerebral circulation, it allows the use of perfusion computed tomography (PCT). PCT assesses perfusion of cerebral parenchyma using specially designed software. It enables the examiner to determine the extent of cerebral infarction as well as the presence and extent of penumbra—potentially viable brain tissue, salvageable if properly irrigated. CTA and PCT enable the selection of patients with indication for mechanical thrombectomy.^26^

Brain MRIs are highly accurate at detecting early strokes as well as recent and prior hemorrhages. However, it is not widely available, its performance and analysis are more technically complex, and it takes longer to perform. An MRI may be associated with MRA and diffusion-weighted and perfusion magnetic resonance imaging, the latter two specific image acquisition series.^27^ MRA studies intracranial and extracranial cerebral circulation, verifying, for instance, the presence of occlusion of the great arteries, an essential criterion for mechanical thrombectomy. Diffusion-weighted magnetic resonance imaging shows the area of cerebral infarction. Perfusion MRI shows the area of penumbra. Mechanical thrombectomy is indicated in case of mismatch between the infarction and penumbra areas in the image, or between the clinical condition and the infarction area—a neurological deficit greater than expected for the size of the cerebral infarction.^28^

A frequent question in the management of extracranial cerebrovascular disease patients is which methods are required for an indication for CEA; the same question is often asked for angioplasty with stenting (CAS). As a rule, there should be two concurring imaging examinations, i.e., showing similar obstructions: a CDU of the carotid and vertebral arteries associated with a CTA, or possibly an MRA. When a contrast-enhanced examination is not possible, a second CDU, performed by a different examiner, may be requested. Symptomatic patients require imaging of the cerebral parenchyma.

## OPTIMAL CLINICAL MANAGEMENT

### Introduction

All patients suffering from carotid artery disease, regardless of indication for surgical intervention, benefit from optimization of clinical management. Optimal clinical management consists of three key elements: (1) adopting healthy eating habits and physical activity; (2) controlling comorbidities and risk factors; and (3) optimization of antithrombotic and lipid-lowering therapy. These measures are necessary throughout the process of managing carotid artery disease, from the moment it is first diagnosed to the late postoperative period, since patients will always suffer from atherosclerotic disease.

### Lifestyle changes and controlling risk factors

The urgent need, as well as the positive contribution, of adopting healthy eating habits and engaging in physical activity to control cardiovascular disease in general is widely known. Obesity is a risk factor for developing atherosclerotic disease, and may contribute to the early onset of carotid artery disease.^29^ For atherosclerosis patients in general, adopting a balanced diet, rich in vegetables and whole grains, eschewing ultraprocessed foods, excess sodium, cold cuts, and fast-digesting carbohydrates, results in improved lab and quality of life indicators and lower rates of cardiovascular morbidity.^30,31^ The frequency at which individuals engage in physical activity is directly proportional to the risk reduction for cardiovascular disease in general and carotid artery disease in particular.^32^

Smoking is associated both with carotid artery disease itself and with increased risk of ischemic stroke, with an almost tenfold increase in relative risk for the latter. Smoking cessation or reduction is key in preventing the progression of carotid stenosis and embolic events.^33^ Approximately 77% of patients who have a first stroke are hypertensive.^34^ Blood pressure (BP) control is directly correlated to decreased incidence and lower personal risk of stroke.^35^ Blood pressure control should be seen as a key element for clinical management of carotid artery disease, and is the subject of specific guidelines.^35-37^

### Recommendation 2.1

It is recommended that patients with internal carotid artery (ICA) stenosis take proactive measures to adopt healthy eating habits, engage in physical activity appropriate for their limitations, and, when applicable, pursue weight loss, smoking cessation, and hypertension control (I/B).^30,35^

### Antiplatelet therapy in asymptomatic carotid disease

The benefits from antithrombotic therapy, particularly antiplatelet therapy, in lowering rates of cardiovascular events in patients with established atherosclerotic cardiovascular disease (heart disease, cerebrovascular disease, peripheral arterial disease [PAD]) have been clear for over a quarter of a century.^38^ However, the weight of evidence specific to antithrombotic therapy, and more specifically for asymptomatic carotid disease patients, is still limited. A randomized controlled trial comparing the use of acetylsalicylic acid (ASA) to placebo failed to find benefits from the therapy.^39^ However, an observational study of patients with asymptomatic stenosis greater than 70% found significant benefits from antiplatelet therapy.^40^ In addition, since atherosclerotic disease is often present in other sites (heart disease, PAD) in this patient population, antiplatelet therapy should be considered for patients who can tolerate the medication.^41^

### Recommendation 2.2

For patients with asymptomatic ICA stenosis, antiplatelet therapy with ASA at low doses (81-325 mg/d) is recommended over other antithrombotic strategies or over the nonuse of antiplatelet therapeutics (APT) (IIa/C).^39,41^

### Dual antithrombotic therapy in asymptomatic carotid disease

Dual antithrombotic therapy (DAT), combining APT and direct anticoagulants, consisting of ASA 100 mg per day associated with rivaroxaban 2.5 mg twice daily for patients with stable cardiovascular disease was the subject of the COMPASS clinical trial.^42^ The COMPASS-PAD substudy^43^ analyzed 7,470 patients from the parent study suffering from PAD and/or carotid artery disease.^43^ In this group, DAT resulted in a 28% decrease (95% CI 0.57-0.90; p < 0.004) in the primary outcome of cardiovascular death, myocardial infarction (AMI), or stroke (SAE), and a specific 46% decrease in all-cause ischemic strokes (95% CI 0.33-0.87). On the other hand, major bleeding increased 1.61 times for patients undergoing DAT (95% CI 1.12-2.31, p < 0.008), requiring a more careful analysis of the risk of bleeding for indication for DAT.^43,44^

An exclusive analysis of outcomes for the 1,919 patients with carotid stenosis greater than 50% failed to find specific benefits for lower SAE for the group, though the incidence of major bleeding was similar for both the groups with and without rivaroxaban. An analysis of the COMPASS study failed to find a specific decrease in ischemic stroke associated with carotid artery disease from DAT, though its use led to a decrease in the general incidence of strokes.^44^ Therefore, even though the specific benefit of DAT for patients with isolated carotid artery disease is unproven, its use may be considered in the presence of other cardiovascular comorbidities associated with carotid artery disease for patients at low risk of bleeding.

### Recommendation 2.3

For patients with asymptomatic ICA stenosis and other cardiovascular comorbidities (heart disease, PAD) and low risk of bleeding, a dual antithrombotic therapy strategy, with rivaroxaban 2.5 mg twice daily combined with ASA 100 mg per day, may be considered (IIb/B).^42,44^

### Lipid-lowering therapy in asymptomatic carotid disease

Lipid-lowering therapy with statins for cardiovascular disease patients should be considered even when serum levels of LDL-cholesterol (LDL-c) are in range.^45^ Statins were associated with a reduction in ischemic stroke for patients at increased risk of cardiovascular events and carotid artery disease, even for patients without severe dyslipidemia.^46,47^ There is enough evidence for the benefits of decreasing the rate of cardiovascular events for patients receiving continuous lipid-lowering therapy with statins, combined or not with intestinal cholesterol absorption inhibitors (especially ezetimibe).^48,49^ Despite the absence of prospective clinical trials into the impact of exclusive lipid-lowering therapy for carotid artery disease patients, data from less specific studies are enough to recommend their use.

### Recommendation 2.4

For patients with asymptomatic ICA stenosis, lipid-lowering therapy with statins associated or not with ezetimibe to prevent cardiovascular events (AMI, stroke, cardiovascular death) is recommended (I/B).^46,48^

Patients with severe dyslipidemia and cardiovascular disease need clear LDL-c reduction targets, since persistently high values keep individuals at increased risk of severe cardiovascular events. In the absence of effective LDL-c lowering, even when receiving optimal therapy with statins and ezetimibe, or when there are major side effects from their use, other strategies should be considered. Prospective randomized controlled trials have been performed for two PCSK9 (Proprotein Convertase Subtilisin/Kexin type 9) monoclonal antibodies or inhibitors, evolocumab and alirocumab. Both lower combined rates of cardiovascular death, ischemic stroke, AMI, myocardial revascularization or hospitalization for angina. Evolocumab lowers combined outcomes by 15% (95% CI 0.79-0,92; p < 0.001), while alirocumab achieves a reduction from 3.3% to 1.7% (95% CI 0.31-0.90; p = 0.02), with an odds ratio of 0.52.^49,50^ There was a 25% specific decrease in ischemic stroke for evolocumab users (95% CI 0.62-0.92; p < 0.005).^51^

Therefore, patients suffering from carotid artery disease and dyslipidemia unresponsive to conventional lipid-lowering therapy with statins/ezetimibe should be considered for additional use of PCSK9 inhibitor drugs.^49-52^

### Recommendation 2.5

For patients with asymptomatic ICA stenosis and dyslipidemia unresponsive to conventional therapy with statins, whether or not associated with ezetimibe, the use of PCSK9 inhibitors should be considered (IIa/C).^49-52^

- A note on the use of proton pump inhibitors (PPI) and their possible interactions with APT: the use of APT, either as single or dual therapy, increases the risk of gastrointestinal bleeding, particularly for higher risk patients.^53,54^ However, there is evidence, though conflicting, that PPIs used as gastric protectors can have an inhibiting effect on APT. Two retrospective studies have found that PPI use was associated with a higher rate of cardiovascular events among patients with a history of recent acute coronary syndrome, supposedly associated with decreased antiplatelet effectiveness of clopidogrel and ASA.^55,56^ A prospective study of healthy individuals found no decrease in the antiplatelet effects of clopidogrel when used concomitantly with the drugs pantoprazole, omeprazole, rabeprazole, esomeprazole, lansoprazole, or dexlansoprazole.^57^

The presence of CYP2C19*2 and CYP2C19*3 cytochrome polymorphism is associated with decreased metabolism of PPIs as well to the lower direct effectiveness of clopidogrel.^58^ However, for patients undergoing dual antiplatelet therapy, pantoprazole use did not interfere with antiplatelet effects, even in the presence of CYP2C19 cytochrome polymorphism.^59^

Therefore, for patients receiving antiplatelet therapeutics requiring the use of gastric protectors, both the choice of APT and of gastric protector should be given proper consideration. The drug of choice is pantoprazole; when it is not tolerated by the patient, H_2_-receptor antagonists (ranitidine, famotidine) are preferred.

## ASYMPTOMATIC CAROTID DISEASE

Strokes are the world's second leading cause of death, and third leading cause of disability. The incidence of stroke is high—12.2 million strokes per year, one every 3 seconds. These numbers have increased by 50% in the last 17 years, especially in lower-income countries, and there is a disastrous trend of strokes in younger patients (63% below the age of 70). Ischemic strokes account for 80-85% of all strokes, and around 25% of those are associated with cervical carotid disease. These numbers clearly show the pressure the disease places on health care systems, as well the relevance of developing strategies to lower their social impact—most ischemic strokes could have been avoided with lifestyle changes and better control of metabolic diseases.^60^

Despite the large body of work, especially recently published guidelines, there is still no consensus for a few key issues, especially when it comes to the management of asymptomatic carotid stenosis. The adoption of a set of rigorous clinical measures (hypertension and dyslipidemia control, smoking cessation, healthy diets, and physical activity) is mandatory, and has led major changes in the treatment of asymptomatic carotid disease. This has led to lower rates of ischemic stroke, but there is evidence that these measures are not enough to prevent ischemic strokes for all individuals. Clearly, not everyone has the same risk of ischemic stroke over time, and prophylactic CEA may be beneficial. Likewise, the role of CAS and of new technologies is the subject of much debate.

The goal of this guideline is to identify and discuss the most relevant issues for carotid artery disease, as well as to develop recommendations to help specialists in their clinical practice. The following questions have been formulated:

- When should screening for asymptomatic carotid stenosis be performed?
- What clinical measures should be adopted to treat asymptomatic carotid stenosis?
- What risk factors increase the risk of ischemic stroke in patients with asymptomatic carotid stenosis undergoing clinical treatment?
- When and how to revascularize an asymptomatic carotid stenosis?

### When should screening for asymptomatic carotid stenosis be performed?

The usefulness of population screening is based on the ability to identify severe carotid stenosis and take appropriate measures to eventually change the course of the disease and prevent cerebral ischemia. The cost-benefit ratio of performing tests to detect carotid stenosis depends on the prevalence of injuries requiring treatment in a given population. Due to its low prevalence—a rate of under 5% of significant carotid stenoses in the general population^61^ —recent recommendations are unanimous regarding carotid artery disease: routine population screening is not recommended.^5,9,62,63^

Screening may be justified for subgroups with stenosis rates of over 20%.^61^ However, identifying carotid stenosis patients is an opportunity to promote patient adherence to optimal clinical management (OCM) and work more effectively to fight the relevant risk factors, thus contributing to lower cardiovascular morbidity and mortality. In specific populations, with multiple risk factors for atherosclerosis, the prevalence of asymptomatic carotid disease rises significantly, justifying screening efforts. The populations at risk for carotid artery disease are patients with systemic hypertension (SHT), coronary artery disease, peripheral arterial disease (PAD), hypercholesterolemia, diabetes mellitus (DM), chronic renal failure, carotid bruit, silent infarction on neuroimaging tests, smokers, and those who have a first-degree relative with history of cerebral ischemia. The presence of two, three or four risk factors increases the prevalence of carotid stenosis ≥ 50%, from 2% to 14%, 16%, and 67%, respectively.^64,65^

Screening is only plausible in the presence of reliable testing, and CDU is considered perfectly adequate. It is the most widely used modality to identify asymptomatic extracranial carotid injuries, with sensitivity and specificity of 94% and 92%, respectively, for stenosis with 60-99% obstruction, though its accuracy is examiner-dependent.^66^ When there is an intent to treat asymptomatic lesions, the test can also be useful to track ≥ 50% stenoses every 6 to 12 months in order to identify the progression of the stenosis grade.^62^

### Recommendation 3.1.1

Population screening for asymptomatic carotid disease is not recommended for the general population (1/B).^67^

### Recommendation 3.1.2

Population screening for asymptomatic carotid disease should be recommended for individuals over 55 years of age with the following risk factors for atherosclerosis (IIb/C):^61,68^

1. SHT;
2. PAD;
3. obstructive heart disease;
4. smoking;
5. hypercholesterolemia;
6. DM;
7. chronic renal failure;
8. carotid bruit;
9. silent infarction on neuroimaging;
10. first-degree relative with history of disease.

### Recommendation 3.1.3

Doppler ultrasound is the examination of choice for asymptomatic carotid disease screening (I/B).^10,64,66^

### Recommendation 3.1.4

A Doppler ultrasound of the carotids every 6-12 months for asymptomatic stenoses greater than 50% is recommended. Expert panel opinion.^5,62^

### Recommendation 3.2

Carotid artery disease screening using Doppler ultrasound should be considered for individuals over 55 years of age with risk factors for atherosclerosis (IIb/C).^10,61,64,66,68^

### What clinical measures should be adopted to treat asymptomatic carotid stenosis?

OCM for obstructive carotid artery disease has become prominent over the past two decades, to the point of challenging one of the most frequent surgical procedures worldwide, i.e., carotid artery revascularization. It has led to the idea that OCM would have radically changed the natural history of the carotid bifurcation disease and that any intervention in asymptomatic patients may be considered obsolete, or even harmful. However, though OCM has mitigated the impact of atherosclerotic disease, globally, there are still significant shortcomings.^69^

With its undeniable effectiveness, OCM should be adopted for all carotid stenosis patients, whether symptomatic or asymptomatic, regardless of having been subjected to interventions of any kind, and have clear benefits in terms of lower rates of cerebral ischemia and cardiovascular mortality. Though the end goal of clinical treatment in this case is preventing cerebral ischemia, its impact on lowering cardiovascular mortality is even more relevant.^47^ OCM, as a key tool in the management of carotid artery disease, combines lifestyle changes, rigorous control of risk factors, and pharmacotherapy; the relevance of each element is discussed below.

### Controlling risk factors

Lifestyle changes and the better control of cardiovascular risk factors they produce are often underestimated by both patients and physicians, perhaps because it is the most difficult to achieve, requiring considerable effort from both parts.

Smoking, for instance, doubles the odds of developing carotid stenosis greater than 50%, and increases two to sixfold the odds of an ischemic stroke compared to rates for non-smokers.^45,69^ Carbon monoxide and thiocyanate, among other substances found in tobacco, promote endothelial dysfunction, lower HDL cholesterol, increase triglycerides and LDL-c, trigger platelet activation, and promote a prothrombotic state. Smoking is also responsible for dulling the beneficial effects of statins, promoting vasoconstriction, and accelerating atherosclerosis, linked to plaque progression. Second-hand smoking and consumption of other substances, such as marijuana, are equally harmful. As physicians, in addition to counseling patients to stop cigarette consumption, we should also provide resources to help them quit, referring them to specialists or prescribing medication of known effectiveness, such as non-toxic nicotine replacement products (e.g., gums and patches). Nicotinic antagonists, such as bupropion or varenicline, are even more effective. Bupropion doubles the odds that a patient will be able to quit smoking, and varenicline is considered even more effective.^45,69^

Obesity promotes arterial compliance alterations. Arterial wall thickening is related to dysfunction of the endothelium and of the smooth muscle cells in the vessel walls, increasing peripheral resistance to insulin and raising cholesterol and C peptide levels in obese individuals. These characteristics are correlated with increased cardiovascular risk overall. An objective marker of the effects of obesity is the intimal-medial thickening of common carotid arteries, classically associated with high risk of stroke and cardiovascular disease.^31^

Engaging in any physical activity lowers the odds of developing carotid stenosis by 20%, and exercising more frequently is correlated with even greater risk reductions. All carotid stenosis patients should be counseled to engage in at least 30 minutes of moderate-intensity physical activity per day, and not spend more than 2 consecutive days without engaging in physical activity.^32^ Another high-impact change to the clinical management of carotid artery disease is diet. Mediterranean diets, comprising vegetables, fruit, grains, olive oil, with some milk and fish and little to no red meat, allowing for low to moderate alcohol consumption in the form of wine, is the most widely recommended.^45^

In terms of risk factor control, SHT is probably the highest-impact factor for increased risk of both ischemic and hemorrhagic stroke. Seventy-seven percent of patients who suffered some form of stroke have SHT, and it is also a risk factor for carotid stenosis.^70^ The key point is that SHT is a modifiable risk factor, which means we there are effective measures at our disposal to lower BP, combined with lifestyle changes. Every 2 mmHg decrease in BP represents a 25% risk reduction (RR) for stroke. No randomized clinical trial has assessed the effect of antihypertensives on individuals with antihypertensives, but we do know from other studies, designed for individuals with no history of vascular disease, that there can be as much as a 45% RR for stroke with blood pressure control, a reduction directly influenced by the decrease in systolic blood pressure.^71^

DM is likely the modifiable risk factor most often neglected by physicians and patients, given the difficulty of maintaining strict glycemic control. Sixteen percent of diabetic patients over the age of 65 die from strokes. Diabetes is associated with an increased risk of carotid stenosis, and glycemic control should be encouraged.^63^

### Antiplatelet drugs and statins

Over the past two decades, there has been a major increase in the use medications now considered pillars of treatment for atherosclerosis: APT, antihypertensives, beta-blockers, and especially statins. The practice has led to a clear RR in strokes and cardiovascular mortality.^69^

Antiplatelet therapy is an independent factor in reducing TIAs and strokes ipsilateral to carotid stenosis. However, they have even greater benefits in terms of lowering AMI mortality, since over two thirds of asymptomatic carotid disease patients also suffer from obstructive heart disease. In addition to lowering cardiovascular mortality, ASA at low doses (75-325 mg/day) improves the prognosis after a stroke. There is no proven benefit from dual antiplatelet therapy for asymptomatic individuals, and clopidogrel should be used as monotherapy in case of intolerance to ASA.^40^

Over the past few decades, the increase in the use of statins has been incredible. Its impact on the progression of atherosclerotic disease is undeniable, though its mechanism of action is not entirely clear.^69^ Statins are competitive 3-Hydroxy-3-methylglutaryl-coenzyme A reductase inhibitors that limit intrahepatic cholesterol synthesis, increase the turnover rates of LDL-C (low-density lipoprotein) receptors, decrease the production of VLDL-c (very low-density lipoprotein), and, less importantly, increase the production of HDL-c (high-density lipoprotein). The combination of this properties likely results in stabilization of the atheromatous plaque, reversal of endothelial dysfunction, and lower thrombogenicity.

An analysis of the individuals in the ACST-1 trial shows that the 10-year risk of stroke and death for asymptomatic carotid stenosis patients undergoing clinical treatment and those treated with CEA was 13.4% and 7.6%, respectively.^47^ However, when we focus on the subgroup that did not use statins, the risk rises to 24.1% for individuals undergoing clinical treatment and to 17.9% for individuals who underwent a surgical procedure, corroborating the effectiveness of statins in the long-term treatment of carotid stenosis.^47^ The goal of long-term medical treatment should be to lower patient LDL-c levels below 70 mg/dL or to 50% of initial LCL-c levels.^72^

### Recommendation 3.3.1

OCM should be adopted for all carotid stenosis patients, regardless of degree of stenosis, to prevent cardiovascular events (I/b).^45,47,69^

### Recommendation 3.3.2

The following measures should be adopted for the clinical treatment of asymptomatic carotid stenosis:

1. healthy diet, physical exercise (I/B);^45,73^
2. ASA at low doses (75 to 325 mg) for stenoses > 50% (I/A);^41,45^
3. clopidogrel 75 mg/day in case of intolerance/allergy to ASA (IIa/B);^40^
4. statins: atorvastatin 40-80 mg/day or rosuvastatin 20-40 mg/day. LDL-c lowered to < 70 mg/dL or < 50 mg/dL;^47,48^
5. antihypertensives targeting BP < 140/90 mmHg and < 140/85 mmHg for diabetics (I/A);^34,35^
6. glycemic control for diabetics with HbA1c < 7% (I/C);^74^
7. medication-assisted smoking cessation (bupropion, varenicline, nortriptiline, nicotine replacement) (I/B);^75^
8. use of PCSK9 inhibitors in case of intolerance to statins/ezetimibe (IIa/C).^51^

### What risk factors increase stroke risk in patients with asymptomatic carotid stenosis undergoing clinical treatment?

The first mention of a group of “highly selected patients” with an asymptomatic carotid stenosis at high risk of stroke who would benefit from carotid revascularization, from a 2011 publication by the American Heart Association (AHA), does not, however, clearly differentiate these patients.^74^ Driven by advances in clinical treatment over the last 30 years, promoting exceptional stroke rates of 1% annually, the need to reassess indication criteria for carotid revascularization has become more urgent. Additional studies have identified factors for increased risk of ischemic stroke, related to the morphology of atheromatous plaques and the presence of silent embolisms.^76,77^

In m 2017, the European Society for Vascular Surgery (ESVS) for the first time recommended in its guideline that the indication for carotid revascularization for asymptomatic individuals with 60-99% stenosis, would depend on the presence of one or more imaging features associated with higher risk for ipsilateral stroke, as long as surgical risk was below 3%.^72^

A 2020 systematic review was the first to describe the features of high risk plaques, their relation to higher risk of ischemic stroke above acceptable estimates, and its 26.5% prevalence, leading to the conclusion that additional information, beyond degree of stenosis, might help identify patients at high risk of stroke.^77^

The role of the degree of stenosis on the risk of ischemic stroke remains under debate. Ultimately, the Asymptomatic Carotid Stenosis and Risk of Stroke Study Group (ACSRS) studies, the Oxford study and a recent systematic review of the literature reaffirmed that the degree of stenosis represents a risk marker, identifying ischemic stroke rates of 5% for moderate stenoses and 15% for severe stenoses under clinical treatment.^78-80^ Therefore, to more effectively select patients for carotid revascularization, elevated risk markers (imaging/clinical) should not replace degree of stenosis as criteria, and should actually be adjusted to it. Risk factors for ischemic stroke (imaging/clinical) are shown in Chart 6.

**Chart 6:** Factors increasing stroke risk in patients with asymptomatic carotid stenosis undergoing clinical treatment.

**Clinical characteristic/imaging**	**Annual rate of ipsilateral stroke/OR**	**References**
1. Silent infarction on CT	3.6% / 3.0	Kakkos et al.^81^
2. Contralateral stroke	3.4% / 3.0	Nicolaides et al.^80^
3. Stenosis progression		Conrad et al.,^82^ Kakkos et al.^83^
a. 50-99%	2.0% / 1.92	
b. 70-99%	4.7%	
4. Plaque area on computerized ultrasound plaque analysis (70-99%)		Nicolaides et al.^84^
a. < 40 mm^2^	1% / 1	
b. 40-80 mm^2^	1.4% / 2.08	
c. > 80 mm^2^	4.6% / 5.81	
5. Juxtaluminal black area on computerized ultrasound plaque analysis (50-99%)		Kakkos et al.^85^
a. < 4 mm^2^	0.4%	
b. 4-8 mm^2^	1.4%	
c. 8-10 mm^2^	3.2%	
c. >10 mm^2^	5.0%	
6. Intraplaque hemorrhage on MRI (50-99%)	/3.66	Gupta and Marshall^86^
7. Impaired cerebral vascular reserve (70-99%)	/6.14	King et al.^87^
8. Plaque lucency on ultrasound		Gupta et al.^88^
a. predominantly echolucent (50-99%)	4.2% / 2.1	
b. predominantly echogenic (50-99%)	1.6%	
9. Spontaneous embolization on TCD (50-99%) > 1 spontaneous embolization in 1 hour	/7.46	Gupta et al.^88^
10. Spontaneous embolization on TCD (50-99%) plus predominantly echolucent plaque on ultrasound (70-99%)	8.9% / 10.61	Markus et al.^89^
11. Degree of stenosis measured by two ultrasound or CT angiography		Kamtchum-Tatuene et al.,^78^ Howard et al.,^79^ Nicolaides et al.^80^
a. 70-99%	14.6% / 5 years	
b. 80-99%	18.3% / 5 years	
c. 50-69%	1% / 5 years	

CT: computed tomography; MRI: magnetic resonance imaging; TCD: transcranial Doppler ultrasonography. Modified from Naylor et al.^5^

### When and how to revascularize an asymptomatic carotid stenosis?

Choosing which treatment would most benefit a patient with asymptomatic carotid stenosis is the product of cultural, political, financial, and philosophical issues, and not simply a technical matter. This explains why different practices are recommended by major opinion makers in health care, based on questionable scientific evidence that still require greater validation and debate. An analysis of the indication for carotid revascularization based on the presence of symptoms reveals health care systems where revascularization is predominantly performed in asymptomatic individuals, such as the U.S. (70%), Germany and Italy (both 60%), and others where revascularization for asymptomatic individuals is an exception, such as the United Kingdom, Canada and Australia (15%), or Denmark, where asymptomatic individuals do not undergo revascularization.^90,91^ It should be stressed that approximately 10-15% of all ischemic strokes are caused by thromboembolisms from a previously asymptomatic carotid stenosis greater than 50%.^63^

According to the latest recommendation from the U.S. Society for Vascular Surgery (SVS), for low surgical risk patients with asymptomatic carotid stenosis greater than 70%, CEA with best medical therapy is recommended for long-term prevention of ischemic stroke (level of recommendation I and level of evidence B).^9^ The choice of treatment depends on the severity of the stenosis, the risk of the intervention, and the likelihood that the procedure will change the course of the disease. The recommendation is based on the results from the VACS,^92^ ACAS^22^ and ACST^47^ multicenter randomized controlled trials (RCT), which showed the superiority of CEA over clinical treatment alone in long-term prevention of ischemic stroke (for instance, 13.3% rate of ischemic stroke for CEA and 17.9% for clinical treatment of asymptomatic individuals 75 and older over 10 years).

The controversy over management approaches based on this data highlights the evolution of current clinical treatment practices with the addition of statins and other rigorous measures to control risk factors, culminating with annual rates of ischemic stroke of 0.9%, which may have a relevant impact an already modest rate, demonstrating the superiority of CEA in these cases.^93^ The fact is that we still do not have adequate, properly designed, and completed studies comparing CEA and OCM showing that clinical treatment alone is adequate for all patients with asymptomatic carotid stenosis.

Analyzing the data from more recent studies, we find that the inclusion of a number of patients with moderate stenosis (50-69%), with low risk of ischemic stroke when undergoing clinical treatment, leads us to underestimate the benefits from CEA for more severe stenoses.^79,94^ The ACSRS trial, the largest and most recent on the natural history of asymptomatic carotid stenosis, found that 70-89% stenosis is strongly associated with risk of ipsilateral ischemic stroke (15% over 5 years), as well as 90-99% stenosis (20% over 5 years), regardless of statin therapy and smoking, compared to moderate stenosis (60-69%; 5% in 5 years).^80^ The results are corroborated by other recent studies, such as the Oxford Vascular Study and a meta-analysis of 23 studies, suggesting that though rates of ischemic stroke have declined over time because of clinical management, patients with severe stenosis remain at high risk of cerebral ischemia.^79,94^

In order to enable clinical practice and develop uniform management practices for asymptomatic carotid stenosis, the degree of stenosis remains a simple, cheap, and reliable risk marker for ischemic stroke; one should not overlook it in favor of other, partially validated risk markers, such as morphological analyses of plaque features on imaging examination (CDU, CTA and MRA). Rather, ideally, one should adopt all proper tools available when selecting patients.^95-97^

In addition to degree of stenosis, plaque progression was also a relevant risk factor for ischemic stroke. Compared to the risk of ischemic stroke for patients receiving clinical treatment according to degree of stenosis and plaque progression, the 8-year risk of ischemic stroke was 4% (50-69%), 8% (70-89%), and 13% (90-99%) for patients whose plaques remained stable during the period; however, for patients with injury progression, risk levels rose to 8%, 15%, and 25%, respectively.^80,83,98^ It should be noted that the risk of ischemic stroke for patients with asymptomatic stenoses smaller than 60% is not currently considered enough to justify an intervention, though the presence of high-risk plaques merit additional studies regardless of the degree of stenosis.

The efficiency of OCM is undeniable in terms of mitigating rates of both ischemic stroke and other cardiovascular events. On the other hand, it is unable to prevent the progression of atheromatous plaques and subsequent carotid occlusion.^45^ Carotid occlusion, the natural history of which was long erroneously considered benign, responds for approximately 40% of all carotid stenosis-related ischemic strokes without prior symptoms, as well as high lifetime risk of ischemic stroke ipsilateral to the occlusion^99,100^ That fact leads us to infer that clinical treatment alone cannot be prescribed indiscriminately to all patients.

Seeking a middle ground, ESVS proposed stricter criteria and the adoption of additional parameters to select high-risk groups and to indicate revascularization, certain that using degree of stenosis as the single criteria would lead to an excessive number of unnecessary procedures, since only 15% of surgical patients would benefit.^101^

The 2017 and 2023 ESVS guidelines suggest identifying carotid stenosis patients at high risk of ischemic stroke using a list of clinical criteria, as well as others, especially those related to atheromatous plaque morphology and the presence of silent ischemia.^5,63^ The criteria were chosen from the results of systematic reviews, multicenter studies, and subgroup analyses from RCTs rather than from a single data source.^77-80^ They are: silent infarction on CT/MRI;^80^ presence of contralateral ischemic stroke^80;^ ≥ 20% stenosis progression;^83^ large plaque area (> 40 mm^2^)^84^ or large juxtaluminal black area (area of pixels with a greyscale value < 25)^102^ adjacent to the lumen without a visible echogenic cap after image normalization and area greater than 4 mm^2^ on computerized ultrasound plaque analysis;^85^ predominantly echolucent (lipidic) plaque on CDU;^88^ intraplaque hemorrhage on MRI;^88^ impaired cerebral vascular reserve on TCD by mean MCA velocities during CO_2_ inhalation or breath holding;^87^ ≥ 1 spontaneous embolization during ≥ 1 h TCD monitoring.^89^

In theory, these parameters might lead to selecting approximately 25% of asymptomatic individuals with at least one risk criteria who would actually benefit from revascularization, according to some authors.^78^ Based on that premise, the 2023 ESVS guideline recommends that average surgical risk patients with an asymptomatic 60–99% stenosis and one or more criteria associated with an increased risk of ischemic stroke should undergo CEA, provided the perioperative stroke/death rates are < 3% and that patient life expectancy exceeds 5 years (Class of Recommendation IIa and level of evidence B—see Recommendation 3.4.1).

The data discussed above represent distinct and well-founded points of view, which divide the opinions in the most widely respected circles in the world of carotid artery disease, leading us to conclude that this guideline cannot adopt an absolute formula for all patients with asymptomatic carotid disease. It is essential that the idiosyncrasies of each individual (age, gender, comorbidities), their ability to adhere to clinical treatment, and their personal preferences be weighed when making a decision about the best course of treatment.

### Recommendation 3.4.1

Carotid revascularization should be considered for asymptomatic patients with 60-99% stenosis, surgical risk < 3%, and life expectancy > 5 years with one or more risk factors for ischemic stroke (IIa/B).^22,47,80,83,84^

### Recommendation 3.4.2

Carotid revascularization should be considered for asymptomatic patients with > 70% stenosis, surgical risk < 3%, and life expectancy > 5 years, considering the individual needs and preferences of each patient (I/B).^22,47,79,94^

Once the decision has been made to perform revascularization on an asymptomatic carotid stenosis patient, a second controversy rears its head: which is the most appropriate technique, CEA or CAS? All current guidelines prioritize CEA as the most appropriate procedure for patients with ≥ 60% or > 70% stenosis, low surgical risk (under 3%), and good life expectancy.^5,9,62,103^ Ultimately, the choice is clearly influenced by the excellent recent results from CEA, with rates of ischemic stroke and death below 3% for asymptomatic patients, leading some authors to suggest that the ideal recommended rate be lowered from 3 to 2% to indicate interventions for asymptomatic patients.^9,78,103^

CAS has become a more attractive alternative because it is less invasive than CEA. The results from CREST and ACTI, the most recent RCTs, were optimistic, and equivalent to major ipsilateral stroke during the perioperative period, reflecting the high quality of the professionals and centers chosen for the studies.^104,105^ On CREST, the difference between the CEA and CAS groups was the lower incidence of strokes within 30 days, larger for the stenting group (2.4% vs. 1.1%); the CAS group had a lower rate of AMI, but with no impact on mortality. However, the equivalent results between the two techniques were not replicated in dataset registries of contemporary clinical practice.

A meta-analysis of 21 dataset registries involving more than 1,500,000 procedures reported significantly higher stroke rates after transfemoral stenting in 11 of 21 registries (52%), exceeding the 3% recommended acceptable complication risk threshold for asymptomatic individuals, while the rate of stroke after endarterectomy exceeded the recommended risk threshold in only one registry (5%).^106^ In another analysis, using data from the SVS Vascular Quality Initiative (VQI), transfemoral stenting was associated with a higher risk of major complications at 30 days (death, stroke or myocardial infarction) compared to endarterectomy (4.6% vs. 1.97%).^9,107^ The preliminary results from ACST-2, the largest RCT ever to compare CAS to CEA, confirm the findings from previous RCTs, with similar outcomes for both groups in terms of stroke, major ipsilateral stroke, and death at 30 days. However, when assessing any ischemic event at 30 days, there was an increase in the number of events in the CAS arm (3.6% vs. 2.4%), particularly non-disabling ischemic stroke (2.7% vs. 1.6%). During the 5-year follow-up, there was no difference in the rates of disabling ischemic stroke between the two groups (0.5% per year).^108^

The latest Cochrane review, with over 3,000 patients, corroborates the results from ACST-2. However, it reports a strong trend for lower risk of any form of stroke and perioperative death for CEA.^109^ Current evidence does not enable us to reach any conclusions about the safety and effectiveness of the two procedures. There is not enough data to recommend transfemoral CAS for asymptomatic individuals.

The new transcarotid CAS with flow reversal (TCAR) technology has had very promising preliminary results, very similar to those for endarterectomy, and may become a useful alternative in our toolkits, especially for high-risk patients.^110^ In case of high surgical risk for endarterectomy, CAS and TCAR may be considered a viable alternative.^9,110,111^ Since every technique available for carotid stenosis treatment (CEA, CAS, and TCAR) has advantages and drawbacks, one could reasonably harness these features in our favor in order to minimize risks and lower long-term stroke rates.

While waiting for new recommendations from ongoing trials comparing clinical treatment with endarterectomy or angioplasty in asymptomatic patients (CREST-2, ACTRIS, ECST-2,), specialists should combine the available diagnostic tools and scientific evidence and, using their clinical judgment, choose a treatment focused on the individual characteristics and needs of each patient in pursuit of the best immediate and long-term results for them.

### Recommendation 3.5.1

CEA is the procedure of choice for asymptomatic patients with 70-99% stenosis, surgical risk < 3%, and life expectancy > 5 years with indication for revascularization (IIa/B).^22,47,80,83,84,106,107^

### Recommendation 3.5.2

Provided anatomy is favorable, CEA may be an alternative for asymptomatic patients with 70-99% stenosis, surgical risk < 3%, and life expectancy > 5 years with indication for revascularization (IIb/B).^92,105,107,110,111^

### Recommendation 3.6.1

CEA should be considered for asymptomatic patients with (IIa/B):^22,47,80,83^

1. stenosis 70-99%;
2. appropriate surgical risk;
3. > 1 imaging criteria increasing risk of ischemic stroke;
4. perioperative stroke/death rate < 3%;
5. life expectancy > 5 years.
6. Low surgical risk.

The choice of treatment depends on the severity of the stenosis, the risk of the intervention, and the likelihood that the procedure will change the course of the disease.

### Recommendation 3.6.2

CAS may be an alternative for asymptomatic patients with (IIb/B):^80,93,105^

1. stenosis 70-99%;
2. appropriate surgical risk;
3. > 1 imaging criteria increasing risk of ischemic stroke;
4. perioperative stroke/death rate < 3%;
5. life expectancy > 5 years;
6. favorable anatomy.

### Recommendation 3.6.3

CAS may be considered for asymptomatic patients with (IIb/):^9,110,111^

1. stenosis 70-99%;
2. high risk for CEA;
3. perioperative stroke/death rate < 3%;
4. life expectancy > 5 years;
5. favorable anatomy.

## SYMPTOMATIC CAROTID DISEASE

### Introduction

The clinical manifestations of extracranial cerebrovascular disease are caused by ischemia—a sharp reduction in blood flow to the brain or eye.^112^ The clinical condition—signs and symptoms—from ischemia are known as a cerebral ischemic event or ocular ischemic event, when it exclusively affects the eyes. The term symptomatic carotid disease refers to patients who had one or more ischemic events in the carotid territory over the previous six months.

### Definitions

There is some confusion in the literature regarding the definition of the timing of a cerebral ischemic event.^113^ The initial event is the first symptom reported by the patient. The index event is the symptom that leads the patient to seek medical care. A recurrent event is one where the same symptom recurs after an asymptomatic period. The most recent event, as the name indicates, was the last to happen in the patient's clinical evolution.

Ischemic events are defined by the duration of clinical presentation, supplemented by findings from imaging tests when available in the health care system.

The World Health Organization (WHO) defines TIA as a “focal neurological deficit lasting of less than 24 hours of vascular origin”^114^ . The AHA suggests the TIA definition be refined based on imaging findings (CTA or MRI): “a brief episode of neurological dysfunction caused by focal temporal ischemia not associated with acute cerebral infarction”.^115^

Ischemic stroke: Likewise, the WHO defines stroke as a “focal (or global) neurological deficit lasting more than 24 hours, with vascular origin”.^114^

Amaurosis fugax (or transient monocular blindness) is defined as “painless temporary loss of vision in one eye, of vascular origin”.^116^ Vision loss may become permanent in case of central retinal artery occlusion.

Other manifestations of cerebral ischemia are defined by specific terms:

Crescendo transient ischemic attack (cTIA): multiple TIAs over a short period, with full recovery between events. The exact number of events that constitute a cTIA is not clearly defined.

Stroke in evolution (SIE): variable neurological deficits, without full functional recovery over time.

### Pathophysiology of cerebral ischemia

From 15 to 25% of all ischemic cerebral events are caused by atherosclerotic injuries in large vessels, especially the extracranial carotid artery.^117^ The remaining 75-85% are caused by cardioembolism, small-vessel occlusion (lacunar infarction), and ischemic cerebral events of indeterminate origin.

### Carotid atherosclerosis

The carotid artery is often affected by atherosclerosis. Atheromatous plaques may form throughout the carotid, from the common carotid artery at the aorta or the brachiocephalic trunk to the intracranial segment of the internal carotid. Anatomic pathology studies show that over 80% of all atheromatous plaques are located at the common carotid bifurcation, extending to the initial portion of the internal carotid, known as the carotid bulb.

### The atherosclerotic plaque

The characteristic lesion of atherosclerosis is the atherosclerotic plaque or atheromatous plaque, a complex lesion of the intimal layer of the vessel wall, consisting of a central core of cholesterol clefts, macrophages, and inflammatory cells, surrounded by layers of smooth muscle cells and connective tissue as well as a fibrous cap separating the core from the arterial lumen. The atherosclerotic plaque usually grows slowly, limiting the arterial lumen and causing a local stenosis that reduces blood flow to the distal vessels. Plaque progression is known to be nonlinear, and there may be one or more episodes of rapid growth from intraplaque events.^118,119^

### The concept of vulnerable plaque

The atheromatous plaque is living tissue, which undergoes changes in morphology and composition over time. These changes are usually slow, with the formation of cholesterol-rich plaque over decades; but they can also happen suddenly, such as intraplaque hemorrhage or necrosis. The changes to atherosclerotic plaque may cause it to become unstable, i.e., increase the likelihood of complications such as embolization and thrombosis. The characteristics which define vulnerable plaque include a large lipid-rich core, a thin fibrous cap, inflammation in or around the plaque, intraplaque hemorrhage and *vasa vasorum* neovascularization.^118,119^

### Intraplaque events

The most frequent pathological process is plaque rupture, with its content extruded into the arterial lumen and taken by the bloodstream to the distal internal carotid artery and its branches to the brain and to the ipsilateral eye.^118^ Another process resulting in embolization is the ulceration of atheromatous plaque, with the formation of thrombi on the plaque surface and the embolization of those thrombi to cerebral arterial vessels. An additional mechanism of cerebral ischemia associated with intraplaque events is internal carotid artery occlusion, which may be caused by intraplaque hemorrhage or by plaque progression to high degree/subocclusive stenosis with subsequent thrombosis.^112^

### Progression time of symptoms

Patients with acute cerebral ischemia (TIA, ischemic stroke and retinal ischemia) should be diagnosed early and treated within hours or days of the ischemic event. The progression time of cerebral ischemia has been categorized as acute, recent and chronic, as summarized in Chart 7.^120^ The interval between symptom onset and onset of care is key to preventing/reducing the damage caused by the ischemic injury and, consequently, neurological sequelae and risk of death.^121^

**Chart 7:** Definition of symptoms by time since onset.

**Definition**	**Ischemic time**
Acute	Up to 2 weeks
Recent	2 weeks to 6 months
Chronic	More than 6 months

### Management of patients with transient ischemia and acute ischemic stroke

The management of patients with neurological instability, categorized as Vollmar Stage III, is one of the most controversial topics in acute cerebral vascular insufficiency.^5,122^ Science is slowly learning to solve the issue of when to intervene on stenotic or even obstructive injuries after an acute cerebrovascular event. Though we have witnessed many advances in the diagnosis and treatment of ischemic and hemorrhagic stroke (HS) in recent decades, for some issues, there are still no clear and undeniable guidelines. The same applies to monocular ischemic manifestations of carotid origin, known as amaurosis, especially amaurosis fugax.^123,124^ The benefits from CEA in terms of preventing additional ischemic cerebrovascular events for patients who have suffered transient cerebral ischemia, either reversible or with small residual neurological deficit, are undeniable.^125,126^ However, the ideal opportunity for revascularization after an acute stroke is not year clear. Most indications are related to the time period between the index event and revascularization.^63,127^ Recently, increasing attention has been paid to the size of the ischemic area found in imaging examinations, especially on diffusion-weighted magnetic resonance imaging.^124,128-139^

Traditionally, symptomatic patients with carotid artery injuries related to an event could only undergo surgical procedures after a month.^127^ That period has grown increasingly shorter, but a waiting period of at least 14 days is still the most frequently recommended in the literature.^132^ However, the recurrence rate for cerebral ischemia is large after an index event—as much as one fourth of all cases at 14 days.^5,63,137,140,141^ Though Strömberg et al.^140^ estimate a 13.5% rate of recurrence for cerebral ischemia at 30 days, numerous publications confirm a worse prognosis.^8,140-144^ Tsantilas et al.,^141^ in a long meta-analysis, find an early risk of a new ischemic event after TIA, amaurosis fugax or non-disabling ischemic stroke at 6.4% at 2-3 days, 19.5% at 7 days, and 26.1% at 14 days. These data are confirmed by various recent papers.^8,142-144^

The consensus is that patients who have had disabling ischemic strokes, categorized as 3 or more in the mRs,^7,145^ or those with ischemia in more than one third of MCA territory (the most severely affected area in carotid artery events) should not undergo surgical procedures before achieving neurological stability, which usually takes weeks.^7,121,139,145^ Hause et al.^136^ have published an assessment of 646 recent carotid artery ischemia patients, of which 56.8% underwent early CEA, less than 14 days after the index event. They categorized events as either large (ischemic area > 4 cm^3^) or small infarctions (volume < 4 cm^3^ or absent). They found small infarcts in 266 patients (41.2%) and large infarcts in 101 cases (15.6%). All patients with small or absent infarcts progressed with no worsened neurological deficit after surgery, but worsening and death occurred in 12 of 101 patients with large infarcts (11.9%—10 encephalopathies, two haemorrhagic strokes).^136^ SHT management and antithrombotic therapy were identical for both groups.^136^ Several recent studies have found similar data,^128-139^ coinciding with a recently published paper where 50 patients who underwent early carotid surgery after acute ischemia, with ischemic areas of up to 3.4 cm^3^, had excellent results, with not neurological worsening or deaths.^124^ It is worth noting that in the same study, 34% of patients underwent surgery within 24 hours, and 68% within 5 days.^124^

### Recommendation 4.1.1.1

Patients with 50-99% carotid stenosis and recent disabling neurological deficit (mRS 2 or less) but no altered state of consciousness, with isolated and/or multiple ischemic areas on diffusion-weighted MR with volume under 4 cm^3^ should undergo surgery as soon as possible (I/C).^128-139^

### Recommendation 4.1.1.2

Patients with 50-99% carotid stenosis and recent disabling neurological deficit categorized as mRs 2 or less who underwent early revascularization should receive preoperative and postoperative hypertension care to minimize the risk of edema, cerebral hyperperfusion syndrome (CHS), and post-revascularization cerebral hemorrhage (I/C).^5,124,128-139,146^

### Recommendation 4.1.1.3

Patients with 50-99% carotid stenosis and recent disabling neurological deficit mRs 3 or more and/or altered state of consciousness, with isolated and/or multiple ischemic areas on diffusion-weighted MR with volume greater than 4 cm^3^ or ischemic area compromising one third or more MCA territory, should only undergo surgery after neurological symptoms fully stabilize (I/C).^136,137,139^

We believe the area will never be the object of randomized controlled trials, given the characteristics of the patients involved, so levels of evidence higher than B are very unlikely.

There are few studies about crescendo transient ischemic attacks and strokes in evolution. Internationally, the two are more widely by the acronyms cTIA and SIE. The prognosis for the two with pharmacological treatment is extremely poor.^147-149^

Though one study shows antiplatelet therapy reduces cerebral microemboli detected by transcranial Doppler ultrasound, what does not occur with heparin use, other papers found no difference in outcomes.^147-150^

The results of interventions on cTIA and SIE vary widely in the literature, with rates of stroke complications and death ranging from 0 to 11% for cTIA and from 2 to 20% for SIE.^149^ However, applying the criteria above for carotid revascularization in patients with neurological instability finds rates of 2-8% for SIE and 0-2% for cTIA.^129,133,134,149^ In these scenarios, even without high-quality evidence, one should administer heparin combined with ASA before opting for an urgent intervention.

What percent risk of stroke and death is considered appropriate for the subgroup of patients with neurological instability? The most recent ESVS guidelines, based on evidence from Biller et al.,^151^ Dellagrammaticas et al.^152^ and Naylor et al.,^153^ consider cumulative risk of up to 6% as appropriate, since the likelihood of neurological deficit and/or death in the absence of an intervention is much higher.^151-155^

### Recommendation 4.1.1.4

SIE or cTIA patients should be administered dual APT immediately and undergo CEA as soon as possible, preferably within 24 hours (IIa/C).^129,133,147-155^

### Management of symptomatic patients with atrial fibrillation (AF) and >50% carotid stenosis

Ischemic events may occur in carotid territory even if the source of the injury lies elsewhere. A meta-analysis found that 12% of patients with AF had carotid stenoses greater than 50%, while 9% of patients with carotid stenosis had AF.^156^ This is the subject of heated debate,^157,158^ and is also an area that will never be the subject of randomized controlled trials, given the characteristics of the patients involved. The individual opinion of each multidisciplinary team prevails; if carotid intervention is indicated, patients should receive anticoagulants as soon as possible after the procedure.^156-159^

### Recommendation 4.1.2

Patients with amaurosis fugax and/or recent neurological deficit, AF, and 50-99% carotid stenosis concurrently should be assessed by a multidisciplinary team, which should specifically assess the plaque's emboligenic features to decide if anticoagulation alone or combined with carotid intervention is the best course of action. After the intervention, the patient should receive anticoagulants as soon as possible (I/C).^153,156-159^

### Free-floating carotid thrombi

Free-floating carotid thrombi have been detected more often recently, thanks to the dissemination of CDU tests in case of neurological instability and the improved spatial resolution of more modern CDU devices. They may also be detected in angiographies and even CT angiography (which, though a static examination, can show thrombi that, from their shape, are clearly sessile). Singh found intraluminal thrombi in the cervico-cephalic circulation of 1.6% of 3,750 CTAs of these vessels, with neurological instability.^160^ They are more prevalent in men and in individuals with coagulation disorders, but are usually associated with atheromatous plaques.^161,162^ According to the meta-analysis from Fridman et al.,^162^ they have extremely poor outcomes, with a rate of silent ischemia, TIA, ischemic stroke, and death of 17.1%.^162^ The literature recommends immediate anticoagulation and antiplatelet therapy after the identification of free-floating carotid thrombi.^160,161^ Though there is no consensus standard therapy, it seems obvious that free-floating carotid thrombi need to be resolved as quickly as possible by a direct approach through CEA, if anatomically feasible.

### Recommendation 4.1.3

When imaging tests find free-floating carotid thrombi in patients, whether symptomatic or asymptomatic, anticoagulation therapy with heparin and APT should be administered, and the patient submitted to an emergency intervention, preferably CEA (IIb/C).^124,159-162^

### Intervention after thrombolysis and mechanical thrombectomy

The issue of when one can safely intervene on residual carotid obstructions after intravenous thrombolysis for treatment of acute ischemic strokes remains controversial in 2023. Approximately 10-20% of patients who undergo thrombolysis under these circumstances have cervical carotid injuries requiring treatment.^5,8,123,163-165^ The hesitation between an early surgical procedure and risking a higher rate of complications, stands in contrast with delaying the removal of carotid injuries, often the etiology behind ischemic strokes. Alteplase, the thrombolytic agent currently available in Brazil, has a half-life of 5 minutes in the blood, but the effects of fibrin degradation products, among other agents, leads to significantly longer-lasting changes.^166^ One of the most worrisome changes is the increased vascular barrier permeability, which can lead to intraparenchymal hemorrhaging and cause a hemorrhagic stroke.^166,167^ Full anticoagulation and even antiplatelet therapy should be postponed for at least 24 hours after lysis and until a CT scan shows absence of hemorrhages.^167^

Indications converging on an earlier intervention are: ischemic brain area below one third of MCA territory; recanalization of previously occluded MCA evidenced by control CTA; ipsilateral carotid artery obstruction with 50-99% stenosis; absence of intracerebral hemorrhage or edema, and especially regression of neurological impairment to 0 to 2 on the mRs.^163,165,168,169^ The contraindications are the mirror images of those criteria, such as: persistent ischemic brain area of more than one third of MCA territory; intracerebral hemorrhage or edema; high surgical risk; hostile neck; and persistent neurological deficit (mRs ≥ 3).^7,170-172^ Every paper listed categorically state the need for rigorous SHT control.

However, the time between lysis and the effective elimination of the source of cerebral ischemia remains under debate. Though there are papers on early CEA within 12 hours of final lysis with excellent outcomes,^170^ most published studies show that earlier interventions are followed by a high rate of CHS, parenchymal edema, and intracerebral hemorrhage.^165,169,171^ Most major guidelines on cerebral ischemia published in recent years do not discuss the ideal period between systemic lysis and carotid interventions.^8,63,123,154,158^ However, several recent high-quality studies state that a 6-day wait should be adopted before intervening.^5,163,165,169,171^

### Recommendation 4.1.4.1

Patients undergoing thrombolysis to treat acute cerebral ischemia should undergo strict BP control throughout the procedure as well as postoperatively to reduce the risk of intracerebral hemorrhage (I/C).^172^

### Recommendation 4.1.4.2

For patients who underwent thrombolysis to treat acute cerebral ischemia, definitive revascularization should be performed at least 6 days after thrombolysis (I/C).^163,164,170^

### Recommendation 4.1.4.3

For patients who underwent thrombolysis to treat acute cerebral ischemia, additional antithrombotic therapy with heparin and/or APT should be postponed for at least 24 hours after the final infusion of thrombolytic agents, and then sustained until definitive revascularization is performed (IIa/C).^164,165,168,169,171^

### Carotid webs as the cause of ischemic strokes

The presence of web- or mesh-like defects, usually located in the posterior wall of the carotid bulb, has become a frequent finding in imaging examinations in recent years, which is certainly related to the greater accuracy in spatial resolution of contemporary CDU and CTA studies.^173,174^

Known as carotid webs, they are often found in the absence of a more obvious etiology for cerebral ischemia, leading to an active search for rare causes, having been identified in 0.9% of cases in the Mr CLEAN RCT.^173^ Unfortunately, this level of imaging quality is not available in all hospitals. Found especially in women with an average age of 46, it is hypothesized that these arterial intimal defects are a variant of fibromuscular dysplasia which create niches for the formation of blood of platelet thrombi, which embolize toward the brain.^175-177^ Modern imaging methods should be used to actively search for carotid webs in young patients with cryptogenic TIA or ischemic stroke.^175,178,179^ Antiplatelet therapy has not been reported as effective to prevent recurrence in ischemia, and an intervention is recommended.^174,180^ There are reports of success with both direct surgical treatment, with segmental resection of the affected area and end-to-end anastomosis of the carotid, and with stenting.^174,178,180,181^

### Recommendation 4.1.5

For symptomatic patients for whom a carotid web ispsilateral to the symptomatic cerebral hemisphere has been detected, and for whom other, more obvious causes have been ruled out by a thorough investigation, segmental resection of the impaired portion with end-to-end anastomosis or stenting may be indicated (IIb/C).^173-181^

### Treatment of acute cerebral ischemia of carotid origin, at high risk for CEA

Most publications recommend CEA as the most effective form of therapy to treat cerebral ischemia of cervical carotid origin.^5,63,124,128-136,138,139,146^ However, in some situations, CEA is a high-risk procedure. Patient classification as at high risk for CEA (HR-CEA) is very variable. Yadav et al.^182^ categorize as HR-CEA asymptomatic individuals with 70-99% carotid stenosis who have one or more of the following risk factors: severe cardiac disease (congestive heart failure [CHF], abnormal stress test, need for open-heart surgery); severe chronic obstructive pulmonary disease (COPD); contralateral carotid occlusion; contralateral laryngeal-nerve palsy; previous radiation therapy to the neck; tracheostomy; recurrent stenosis after CEA; and age greater than 80 years. Serious adverse events (SAE) of 5.8% for CAS and 6.1% for CEA do not justify an intervention for any of these groups of asymptomatic individuals. Several of these factors cannot be supported as high risk for CEA, such as COPD (surgery may be performed under cervical plexus block), contralateral occlusion (the brain may be protected by a temporary internal shunt), and advanced age. In fact, this has become an indication for CEA in elderly patients, as evidenced by the CREST trial.^183^ The other factors do indeed represent high risk for CEA.^103,111,184,185^ In these specific cases, CAS should be considered.^5,103,111,124,182-185^

The indications for both forms of treatment are discussed in the specific chapters on indications for treatment in this guideline.

### Recommendation 4.1.6

For symptomatic patients with 50-99% carotid stenosis, confirmed to be at high risk for CEA, angioplasty with stenting should be considered (IIb/B).^104-112-184-186^

### Management of patients with subacute (after two weeks) and chronic ischemia

Management of patients with acute cerebral ischemia has been widely studied, and guidelines for the management of TIA/acute ischemic stroke patients are available in the literature and discussed in this guideline. However, the management of patients with symptoms lasting longer than 2 weeks remains controversial. Studies show that the risk of a new ischemic event is higher in the acute stage, up to 2 weeks after the index event.^124^ The high risk persists after the acute period, depending on the initial cause of the ischemia, controllable risk factors (e.g., SHT), and care offered to the patient.

Strömberg et al.,^140^ in their assessment of a series of cerebral ischemia patients at various points after symptom onset, found an increased risk of recurrent stroke at 3 months after the index event. Other studies have confirmed these findings.^142,186-193^

### Initial assessment

Patients treated more than 14 days after symptom onset face decreasing risk of recurrent ischemia. At this moment, clinical assessment of patients consists of:

1 – General clinical examination and detailed neurological examination:

Assessment of possible cause of ischemic event in addition to carotid artery disease, such as arrhythmia and SHT. Assessment of neurological sequelae and patient classifications in a neurological deficit scale, such as mRs.^194^ European guidelines recommend patients be assessed by a multidisciplinary team, consisting of clinicians, neurologists, radiologists, vascular surgeons, and interventional surgeons.^103^

2 – Imaging examinations of carotid arteries and the brain:

Patient should undergo carotid CDU and CTA from the aortic arch to the intracranial arteries.^195^ Contrast-enhanced MRA is more sensitive than CTA to assess ischemic brain injuries.^196^

3 – Assessment of surgical and anesthetic risk, considering the possibility of invasive treatment of carotid injuries.^194^

### Clinical treatment

All patients with recent cerebral ischemia should undergo OCM, consisting of lifestyle changes to control risk factors and the use of medications for secondary prevention of new ischemic events.^197^ The controllable risk factors are SHT, smoking, dyslipidemia, DM, sedentary lifestyle, and obesity.

Rigorous SHT control is the most impactful risk factor for preventing new events in patients with recent cerebral ischemia.^37,71^

SHT with systolic peaks may lead to new ischemic events via plaque embolization, intracranial small-vessel (lacunar) infarctions, and hemorrhages in the infarcted area or penumbra.^198^ Smoking cessation is also key.^197^ Risk factor control and medication use for secondary prevention are discussed in separate chapters in this guideline.

### Recommendation 4.2

Patients with recent cerebral ischemia should receive OCM, with rigorous BP and DM control, smoking cessation, and administration of statins and APT (I/A).^37,71,197,198^

### Invasive treatment

Invasive treatment (CEA or CAS) should be selective, according to the patient's neurological condition, their comorbidities, and the availability of an experienced surgical and interventional team.

### Patient selection for invasive treatment

The patient's neurological condition should preferably be assessed by a multidisciplinary team. The tool most frequently used to assess sequelae from recent ischemic events is mRs.^7^ Patients with normal neurological condition or minor sequelae, with autonomy in daily living, are candidates for invasive treatment.^139^ Patients with severe neurological sequelae (mRs 4 and 5) are not candidates for invasive treatment and should be kept on permanent OCM.^145^

Patients scoring as mRs 3—with moderately severe sequelae, but some degree of autonomy—should be assessed on a case by case basis. Patients with severe ipsilateral injury (stenosis greater than 70% or complex plaque at high risk of intraplaque event and risk of new cerebral event) and/or severe injury and occlusion of the contralateral carotid artery should be considered for CEA, even if there is no expectation for recovery from existing sequelae.

### Recommendation 4.3

Patients with severe neurological sequelae (mRs 4 and 5) are not candidates for invasive treatment and should be kept on permanent OCM (IIa/C).^139,145^

### Carotid endarterectomy (CEA)

Surgical treatment—carotid endarterectomy—for atherosclerotic injuries in the extracranial carotid artery was introduced in the 1950s and refined over the following decades.^199^

### Indications for carotid endarterectomy

Starting in the 1990s, the benefits from CEA in recently symptomatic severe extracranial carotid artery stenosis were shown by three major multicenter trials: NASCET, ECST, and VA Carotid Trial.^200-202^ The results from these trials, combined with other studies comparing CEA to clinical treatment, were analyzed by the Carotid Trialists Group in a series of meta-analyses.^203-205^ Those analyses led international guidelines to a level of recommendation A for CEA in patients with stenosis greater than 50% who had suffered cerebral ischemic events (TIA or ischemic stroke) over the previous 6 months.^5,9,23,63,68,198,206-210^

The benefits from CEA in terms of preventing ischemic events are compared to the surgical and anesthetic risk of CEA even for elderly patients with multiple comorbidities. Meta-analyses of studies and published case series have concluded that the benefits from CEA are only statistically significant when rates of severe complications (stroke, AMI and death) are under 6%.^203-205^

### Recommendation 4.4

For patients with stenosis greater than 50% and symptoms over the previous 6 months, ipsilateral CEA is recommended as long as the risk of perioperative stroke and death is lower than 6% (I/A).^5,9,23,63,68,198,203-210^

### Risk reduction from CEA in subgroups

Candidates for CEA are a heterogeneous population. The benefits from CEA in reducing the risk of new ischemic events are not the same for all patients. There are groups of patients who benefit more depending on clinical and imaging variables.

Subgroup analyses of patients randomized to the NASCET and ECST studies identified the variables listed in Chart 8 as influencing RR for new ischemic events and, consequently, the benefit from CEA. Benefits are measured as the number of patients that need to be treated to benefit a single patient, i.e., the number needed to treat (NNT). The lower the NNT, the greater the benefit. RR should be taken into consideration in the decision to refer symptomatic patients for CEA. The decision should always be made on a case by case basis, weighing the benefits of CEA compared to OCM.^203-205^

**Chart 8:** Variables influencing risk reduction for new ischemic events.

**VARIABLE**	**RR % (NNT)**
Sex	M: 11%^118^
	F: 2,8%^201^
Age	< 65 years old: 5.6%^187^
	65-75 years old: 8.6%^124^
	> 75 years old: 19.2%^116^
Number of comorbidities (risk reduction in 2 years)	CEA – 0-5:17%; 6: 23%; 7 or +: 39%
	OCM – 0-5: 11%; 6: 6%; 7 or +: 8%
Type of stroke: cortical *versus* lacunar	cortical: 15%^211^
	lacunar: 9%^121^
Plaque surface	smooth: 8%^140^
	irregular: 17%^117^
Hemispheric *versus* ocular symptoms	ocular: 5%^191^
	TIA: 15%^211^
	Stroke: 18%^117^
Intracranial occlusive disease associated with stenosis degree	50-69%: 19%^116^
	70-85%: 29%^114^
	> 85%: 45%^113^
Contralateral carotid occlusion	occlusion: 24%^115^
	no occlusion: 13%^119^

RR: risk reduction; NNT: number needed to treat; CEA: carotid endarterectomy; OCM: optimal clinical management; TIA: Transient ischemic attack.

### Recommendation 4.5

The decision to refer patients for CEA should be made on a case by case basis, considering the risk reduction for future ischemic events (I/B).^203-205^

### Patient prep for CEA

CEA is an elective midsize surgery, but often performed as an urgent procedure in elderly patients with multiple comorbidities. Patients are required to be undergoing OCM. The medication classes that make up OCM are listed below:

Antihypertensives: strict BP control before and during CEA significantly reduces the short and long-term risk of new ischemic events.^198,212,213^ Persistent SHT with systolic blood pressure > 180 mmHg associated with increased risk of perioperative stroke, CHS and death.^213^

Statins: In addition to lowering serum cholesterol levels, statins are known to have pleiotropic effects on the arterial wall resulting in stabilization of atherosclerotic plaques.^214-216^ Randomized controlled trials have confirmed these protective effects.^217-219^ A meta-analysis of randomized trials found a significant reduction in complications and mortality for patients receiving statin treatment during the perioperative period.^220^

### Recommendation 4.6

In candidates for invasive treatment, statin therapy should begin before surgery and continue indefinitely (I/A).^220^

Antiplatelet therapy (APT): traditionally, patients referred to CEA receive antiplatelet monotherapy with ASA at doses ranging from 81 to 325 mg.^215^ The consensus is that patients should continue receiving antiplatelet therapy with ASA during the CEA perioperative period.^221,222^ Combined antiplatelet therapy, ASA plus clopidogrel or dipiridamole or ticagrelor, for CEA candidates has been the subject of several randomized trials with conflicting results.^223-226^ Surgeons worry about intraoperative bleeding and cervical hematomas during the immediate postoperative period, but studies have found no increase in these forms of hemorrhagic complications.^227,228^ The decision to add other APT to ASA during the CEA perioperative period remains at the surgical team's discretion.^215,225^

### Recommendation 4.7

Candidates for invasive treatment of symptomatic extracranial carotid injuries should be placed on antiplatelet therapy with ASA (IIa/A).^220,221^

### Recommendation 4.8

Other antiplatelet therapeutics in addition to ASA during the CEA perioperative period may be added at the surgical team's discretion (IIa/B).^225^

Anticoagulants: candidates for CEA after the acute period (> 14 days) receiving anticoagulants should be managed like patients who are to undergo any other form of arterial surgery. Direct oral anticoagulants should be suspended 48 hours before the operation; vitamin K antagonists should be suspended until the international normalized ratio (INR) falls below 1.8.^229^ For patients at high risk of thromboembolic complications, low molecular weight heparin (LMWH) can be used as bridge therapy.^230^

Management of DM: there are no prospective studies about the importance of glycemic control for diabetic patients undergoing CEA. Clinical common sense suggests diabetic patients should have good glycemic control during the perioperative period.

### Carotid artery angioplasty with stenting

In the 1990s, a new procedure to treat atherosclerotic lesions of the extracranial carotid artery was introduced: angioplasty, initially without, later with stenting. After discouraging experiences early on, the routine use of self-expanding stents protection filters to prevent intraoperative embolization helped improve the procedure. Unlike CEA, only performed by vascular surgeons (and some neurosurgeons), various specialists can perform CAS: vascular surgeons, interventional radiologists, neuroradiologists, neurosurgeons, and interventional cardiologists. The involvement of so many medical specialties in the endovascular treatment (ET) of carotid artery injuries has led to the development of an extensive and heterogeneous literature.^9^ Various medical societies have published CAS guidelines, but opinions still vary regarding indications, cerebral protection techniques, and management of complications. Currently, CAS has a significant but limited role in the treatment of symptomatic extracranial carotid stenoses.

### Indications for CAS

The emergence of CAS as an alternative to CEA has led to prospective randomized trials comparing the two.^183,230-235^ Though several older studies found the same results, a 2019 meta-analysis of 20 prospective randomized trials showed that CAS had significantly higher rates of stroke, stroke/death, and stroke/AMI/death at 30 days than CEA.^236^ Subgroup analyses of those studies found that CAS had inferior results for women, patients over the age of 70, procedures performed within 14 days of the index event, patients with complex and sequential plaque, and patients with focal white-matter lesions on MRI. These studies enable us to state that CAS is not the procedure of choice for most symptomatic patients who are candidates for invasive treatment.

On the other hand, there are patients at high surgical and anesthetic risk and/or for whom CEA is technically more difficult or even prohibitively risky. In these real-life situations, CAS might be acceptable as a safer alternative to CEA (see Chart 9).^111,184,236-239^

**Chart 9:** Indications for carotid artery angioplasty with stenting.

**INDICATIONS FOR CAROTID ARTERY ANGIOPLASTY WITH STENTING**	
**Indication**	**References**
Distal extracranial internal carotid injury (C1 – C2)	AbuRahma^184^
In-stent restenosis	Arhuidese et al.^237^
Neck irradiation	Fokkema et al.^238^
Cervical spine deformities	Fokkema et al.^238^
Neoplasms, tracheostomy, etc.	Fokkema et al.^238^
Neck dissection	Fokkema et al.^238^
Contralateral recurrent laryngeal nerve injury	Batchelder et al.^236^
High anesthetic risk patients (COPD, CHF, etc.)	Gurm et al.,^111^ Fokkema et al.^238^
Contralateral carotid occlusion	Kokkinidis et al.^239^

The indications listed in Recommendation 4.9 are not corroborated by randomized prospective trials. Therefore, recommendations related to indications for CAS have level of recommendation II and level of evidence B or C.

### Recommendation 4.9

CAS may be considered for patients with recently symptomatic stenosis greater than 50% with distal extracranial internal carotid stenosis, recurrent postoperative stenosis, hostile neck (prior radiation therapy to the neck, neck dissection etc.), recurrent laryngeal nerve injury, high anesthetic/surgical risk, and contralateral carotid occlusion, as long as the risk of perioperative stroke/death is < 6% (IIb/B and C).^183,238,240,241^ .

Patient preferences should be respected as long as there are no contraindications for CAS.

### Transcarotid artery revascularization with stenting

In this new form of CAS, transcarotid artery revascularization with stenting (TCAR), access is provided directly through a short sheath placed via an open surgical access to the proximal common carotid artery. The device enables reverse flow in the internal carotid through a sheath for reinfusion into the femoral vein or ipsilateral jugular by temporary occlusion of the proximal common carotid. The primary advantage of TCAR is forgoing the navigation of guidewires and catheters from the femoral artery, bypassing the aortic arch, to reach the carotid for stenting. There are no prospective randomized trials comparing TCAR with femoral access for CAS. The best information available comes from the ROADST 1 and 2 studies, which showed the safety and effectiveness of the device and a paired analysis of TCAR with patients who underwent transfemoral CAS.^242-244^ The results show a significant reduction in stroke and death rates for patients treated with TCAR compared to transfemoral CAS.^245^

### Recommendation 4.10

Transcarotid CAS (TCAR) is a safer and more effective alternative to transfemoral CAS for candidates for CAS (IIa/C).^242-245^

### Patient prep for CAS

Clinical assessment and patient prep for CAS are the same as for CEA. The difference is the anticoagulation and antiplatelet therapy used in CAS. Studies have found conflicting results, but specialists who perform CAS prefer a loading dose of ASA (325 to 650 mg) or clopidogrel (150 to 300 mg) one day before the procedure or earlier.^185,246,247^

### Symptomatic carotid artery disease after 6 months

Conceptually, patients whose last ischemic event happened over 6 months prior are considered asymptomatic. Long-term follow-up studies show that the risk of an ischemic event in this population is small. These patients should remain under OCM. Patient risk under contemporary OCM is below 1% per year.^247^ Under OCM, a minority of patients with asymptomatic carotid injuries do suffer cerebral ischemia. The job of physicians caring for asymptomatic carotid stenosis patients is to identify which patients are at higher risk of cerebral ischemic events.^248^ Management of symptomatic carotid artery disease patients after 6 months is discussed in “Symptomatic carotid disease” chapter.

## OPEN SURGERY

The AHA defines CEA as an intermediate risk procedure.^249^ Determining the risk of serious adverse cardiac events enables us to determine the preoperative assessment and management required for each patient individually. Anatomical risk for surgical treatment of the carotid should also be established with the goal of choosing between open and endovascular surgical techniques by establishing which pose the lowest risk of complications for each patient. The markers of high anatomical risk for open carotid surgery are the presence of recurrent stenosis after CEA, previous radical neck dissection, previous cervical irradiation, contralateral laryngeal nerve injury, and tracheostomy.^250^

Most patients with bilateral carotid injuries have asymptomatic injuries or a single symptomatic side, and it is rare for symptoms to manifest on both sides. Simultaneous bilateral CEA has the risk of severe complications, such as bilateral injuries to the recurrent laryngeal or hypoglossal nerves. Therefore, in case of bilateral injuries, CEA should preferably be performed in stages. If simultaneous surgery is extremely necessary, physicians should opt for bilateral carotid artery angioplasty or unilateral CEA and contralateral angioplasty.^251-253^

CEA may be performed under general anesthesia or locoregional anesthesia (LRA), but their outcomes are cause for controversy. LRA may have benefits in term of lower response to surgical stress, using consciousness during the procedure to monitor brain perfusion, and lower rates of shunting. However, its drawbacks include possible patient anxiety during the procedure, the need for deep sedation for certain periods in case of discomfort, and the need to switch to general anesthesia during the procedure, a rare occurrence in most studies. The GALA (General anesthesia versus local anesthesia for carotid surgery trial) study was the largest randomized trial to compare anesthesia techniques; it included 3,526 patients, and found no significant differences in rates of stroke, AMI or death between groups.^254^ The decision depends on the experience and the choice of team tasked with performing the procedure.

The incision can be longitudinal, parallel to the anterior border of sternocleidomastoid muscle, or transverse, with no difference in the incidence of cranial nerve injuries.^255^ Doppler ultrasound may be used to outline the carotid bifurcation, enabling the use of smaller incisions, associated with lower incidence of cranial nerve injuries.^256,257^

The use of temporary internal shunts in CEA may be adopted routinely or selectively. Carotid clamping may promote a hemodynamic stroke, which shunting prevents. During clamping, brain perfusion may be measured using electroencephalography, carotid artery stump pressure, transcranial Doppler ultrasound, transcranial cerebral oximetry, or infrared spectroscopy. However, the most reliable method is to assess consciousness level under LRA. Surgeons who shunt routinely have lower associated rates of complications compared to surgeons who shunt selectively.^258^ A meta-analysis of 3,856 patients found a 1.4% average rate of perioperative stroke among patients submitted to routine shunting and 2% for routine nonshunting.

For patients submitted to selective use of shunting, the rate varied according to the monitoring method used: 1.1% for LRA, 1.6% for electroencephalography and retrograde carotid artery pressure, and 4.6% for transcranial Doppler ultrasound. Shunting may be used routinely or selectively, depending on the surgeon's choice.^259,260^

### Recommendation 5.1

Patients who undergo CEA may be submitted to routine or selective shunting, depending on the surgeon's choice (IIa/C).^258,260^

In conventional CEA, patches with different materials, such as autogenous vein, expanded polytetrafluoroethylene (ePTFE), polyester, and bovine pericardium, may be used. In a meta-analysis of randomized controlled trials involved 4,400 patients representing seven different techniques (primary carotid closure, n = 753; eversion endarterectomy, n = 431; vein patch closure, n = 973; ePTFE patch, n = 948; polyester patch, n = 828; bovine pericardium patch, n = 249; and polyurethane patch, n = 258), eversion CEA and conventional CEA with ePTFE and pericardium patches had the lowest rates of stroke and death. The lowest rates of recurrent stenosis came from the eversion CEA and ePTFE and bovine pericardium patch groups, while the highest rate came from the primary carotid closure and polyester patch groups.^261^ A meta-analysis of 10 randomized controlled trials involving 2,157 patients found that patch closure lowers the risk of perioperative stroke and later stroke, as well as the risks of perioperative arterial occlusion and recurrent stenosis in conventional longitudinal CEA.^262^

### Recommendation 5.2

Routine patch closure is recommended for patients undergoing conventional CEA (I/A).^261,262^

Eversion CEA involves an oblique transection of the ICA carotid bulb. Its advantages are avoiding the implantation of synthetic material, being quicker when compared to longitudinal endarterectomy, and enabling the correction of the ICA if redundant. Its drawbacks are the greater difficulty for shunting and distal access to the ICA. The EVEREST randomized controlled trial compared eversion and conventional endarterectomy techniques for 1,353 patients and found no significant differences in the rates of stroke, AMI and death between them. Recurrent stenosis rates were lower for the eversion endarterectomy group.^259^ A meta-analysis of 25 studies (five RCTs and 20 observational studies) assessed 49,500 patients, comparing 16,249 eversion endarterectomy patients to 33,251 conventional CEA patients. The data from the randomized trials found no significant difference in rates of stroke, death, AMI, and cervical hematomas at 30 days between the two groups. However, the rate of recurrent stenosis was lower for the eversion endarterectomy group (p = 0.001). The data from observational studies showed a significant reduction in rates of death at 30 days (p < 0.001), stroke (p < 0.001), death/stroke, (p < 0.001) and recurrent stenosis (p = 0.032).^263^

### Recommendation 5.3

Surgeons may choose between eversion CEA and conventional CEA with patch closure at their discretion (IIa/A).^263^

Only symptomatic patients with carotid coils, kinks and loops for which a clear link between the anatomical anomaly and their symptoms has been established should undergo surgery. A study analyzed 92 patients randomly assigned for surgery or medical treatment, with a median follow-up period of 5.9 years: the clinical treatment group had rates of 5.5% for carotid thrombosis and 6.6% for stroke, with no incidence in the surgical group.^264^

High carotid bifurcation represents technical challenges that increase the risk of perioperative stroke and cranial nerve injuries. Ideally, the condition should be established by preoperative examinations. CTA helps this process since bone structures are part of the modality. Several procedures may be used to enable access to the internal carotid artery distal to Blaisdell's line: nasotracheal intubation (promotes a wider angle between the mastoid process and the mandible), ligation of branches of the external carotid artery, and resection of the digastric muscle. Mandibular subluxation should be planned preoperatively, with nasotracheal intubation and the use of interdental steel wiring between the lower premolar and upper canine teeth. Braiding the two wires enables the anterior displacement of the mandible by approximately 1 cm.^265^

Carotid artery venous bypasses may be indicated to treat infected patches, removing carotid artery stents, or technical issues caused by injuries to the arterial wall during CEA. Bypasses may use devalvulated great saphenous vein, ePTFE or Dracon grafts.^241,266,267^ Obviously, sintetic material is contraindicated in the presence of infection.

## ENDOVASCULAR SURGERY

As described in previous sections of this guideline, the prevalence of carotid artery disease and its severe complications, especially TIA and ischemic stroke, require the attention of the medical community as a whole.^268^ Adequate prophylactic measures and indication for early treatment may prevent plaque progression and unfavorable outcomes for patients.^269^

### Introduction and history

Mathias described carotid angioplasty in 1981 and despite initially favorable results, the procedure was associated with several complications, especially the dislodgement of plaque fragments, leading to brain embolisms, dissections, and recurrent stenoses. Following coronary interventions, stenting was combined with balloon angioplasty, and since 1994 have become part of the endovascular treatment (ET) for carotid artery disease.^270^ There has been major progress in the development of devices in general, catheters, wiring, cerebral embolic protection devices (CEPD), balloons, and stents, as well as the technical skills of medical professionals. An especially important development was the introduction of CEPDs by Theron, as well as double-layer stents.^271^

### Preparation for carotid artery angioplasty

Historically, most CAS procedures involved interrupting anticoagulation therapy preoperatively. In 2019, guidelines from several societies recommended not interrupting anticoagulation therapy except in cases of high risk of bleeding.^272^ The AHA recommended daily use of ASA as cardiovascular prophylaxis for patients with cardiac morbidity based on the decrease in morbidity and mortality from antiplatelet therapy.^273^ Evidence from antithrombotic therapy for secondary prevention of recurrent stroke in symptomatic patients with carotid atherosclerosis are even more robust.^105^

The benefits of combining ASA with clopidogrel for patients submitted to carotid stenting are clear, decreasing the rate of neurological complications. Extrapolating from the CREST protocol, the optimal duration of dual antiplatelet therapy is of at least 4 weeks.^274^ The choice between dual therapy versus single therapy should consider the risk of intracranial or systemic hemorrhage (major bleeding: 3.4% vs. 1.5%, respectively).^275^

Particularly for ischemic stroke patients with AF receiving oral anticoagulation therapy with direct oral antiacoagulants (DOACs), there was no significant difference in rates of stroke or in-stent occlusion compared to dual or single antiplatelet therapy with DOACs (3% vs. 5% (p = 0.72) and 2 vs. 0% (p = 0.20)), complicating the issue of which antithrombotic combination is best for AF patients submitted to CAS.^276^ A retrospective cohort study of 91 patients found a 23.8% increase in the rate of bleeding during the first month of triple therapy (dual antiplatelet therapy + DOACs) compared to dual therapy (4%) or DOACs with one antiplatelet drug (0%).

There were also no thromboembolic events in the triple therapy group compared to one event each in the other groups.^277^ At 90-day follow-up, there were similar rates of good functional outcomes for dual antiplatelet therapy compared to DOACs + antiplatelet drug (36% vs. 42%, p = 0.77), but late mortality was significantly higher for the group not receiving anticoagulation therapy (32% vs. 0.4%, p = 0.020), as confirmed by age-adjusted logistic regression (p = 0.021).^278^

For patients undergoing CAS, antiplatelet therapy with ASA (75-325 mg daily, usually 100 mg/day) combined with clopidogrel (75 mg daily) is indicated. The latter should start at least 3 days before stenting; in urgent cases where a waiting period is not possible, as a 300 mg loading dose followed by a maintenance dose to be administered indefinitely.^105,279-281^

Monotherapy with clopidogrel after dual antiplatelet therapy for 1 month after the procedure, compared to ASA combined with clopidogrel for 12 months, had the benefit of reducing major bleeding events without association with increased cardiovascular events in coronary stents.^282^ Patients requiring antiplatelet monotherapy indefinitely after percutaneous coronary intervention had lower rates of adverse events (stroke/AMI) with clopidogrel compared to ASA.^220,277,278,283^

Statins are also frequently studied. A meta-analysis of six observational studies (n = 7.503) found patients on statins before CEA had lower perioperative mortality (0.2% vs. 1.3%) compared to patients not on statins (OR 0.26; 95% CI 0.1-0.61), and decreased perioperative rate of stroke, though not significantly lower, i.e., 1.4% vs. 3.0% (OR 0.4; 95% CI 0,15-1.09).^283^ In 11 other observational studies (n = 4,088), the same benefits were achieved with statins for mortality (OR 0.30; 95% CI 0.10-0.96) and perioperative stroke (OR 0.39; 95% CI 0.27-0.58).^284^ Statin therapy before carotid procedures has decrease peri and postoperative complications and shows promise in reducing ischemic events both for CEA and for CAS.^213,285^

Blood pressure control is also a contributing factor for better outcomes, but physicians should be careful not to lower BP too much and compromise cerebral perfusion. Systolic BP above 180 mmHg is an independent risk factor for stroke after CEA (there is no published data for CAS).^213^

### Recommendation 6.1.1

The use of statin therapy before CAS is recommended (I/A).^213,284,285^

### Recommendation 6.1.2

The use of ASA therapy (75-325 mg) daily before CAS is recommended (I/C).^105,275^

### Recommendation 6.1.3

The use of clopidogrel (75 mg/day) should begin at least 3 days before CAS; if not possible, a single loading dose of 300 mg is recommended (I/C).^220,274,275,277,278,282,283^

### Recommendation 6.1.4

Continuation of ASA therapy combined with clopidogrel for at least 4 weeks after stenting and continuation of antiplatelet monotherapy indefinitely (preferably clopidogrel 75 mg) are recommended.^282^

### Recommendation 6.1.5

Patients on oral anticoagulation therapy who for other causes do not need to suspend it for percutaneous access should proceed with postoperative DOACs + anticoagulation monotherapy. Expert panel.^276-278^

### Recommendation 6.1.6

Rigorous BP control is recommended. Hypertension (> 180/90 mmHg) and hypotension (systolic < 100 mmHg) should be treated pharmacologically (IIa/C).^212^

### Pre or postdilation in carotid artery angioplasty

The advantages of predilation of carotid injuries over postdilation after stenting are still controversial. Avoiding postdilation was protective against persistent hemodynamic depression in a meta-analysis of six cohort studies involving 4,652 patients (RR 0.59; 95% CI 0.39-0.87, p = 0.03). The impact of hemodynamic instability in the clinical progression of patients remains murky. There were no statistically significant increases in the rates of death, stroke, TIA or AMI in these groups of patients. It is worth noting that the study did not discriminate between closed-cell and open-cell stents.^286,287^

When a retrospective study compared the two groups, one with suboptimal predilation (3.0-4.0 mm balloon at 8-10 atm) followed by postdilation (5.0-5.5 mm balloon at 8-10 atm) after stent release (n = 130), and the other with stent deployment after optimal dilation (4.0-50 mm balloon at 10-14 atm) not submitted to balloon postdilation (n = 237), perioperative asymptomatic ipsilateral microemboli were observed in cranial DW-MRI of 25 patients (10.5%) in the CAS group with one dilation and 24 (18.5%) in the pre and postdilation group. The difference between the two groups was statistically significantly different (p = 0.033). It is worth noting that different brands of both open-cell and closed-cell stents were used, and various filters and proximal protection systems, features not assessed in the study.^288,289^

### Recommendation 6.2

The surgeon should define the timing of the carotid artery angioplasty, taking into consideration the anatomy of the carotid injury, degree of stenosis, and choice of stent. Expert panel.^286,288,289^

### Recommendation 6.3

During CAS, avoiding repeat ballooning is recommended. Expert panel.^286-288,290^

A retrospective analysis of SVS-VQI data identified a total of 10,074 patients, including 688 patients treated with primary stenting (6.8%) and 9,386 patients treated with predilation (93.2%). Perioperative stroke (30 days/death) was found in 3.5% of cases (n = 353), including stroke in 2.4% (n = 237) and death in 1.5% (n = 152) of cases.^289^ Cerebral protection devices were used in 76.6% of primary stenting patients, compared to 96.7% in the predilation group (p < 0.001). In multivariate logistic regression analysis, adjusting for baseline differences between groups, the primary stenting group had no significantly different outcomes (stroke and death) compared to the angioplasty group (OR, 1.15; 95% CI 0.72-1.83).^288-290^

### Cerebral embolic protection

The use of cerebral embolic protection devices has become extremely useful in decreasing the rate of ischemic cerebral events during CAS, as seen in a wide range of studies, though not in all. Though embolic material is regularly removed from filters, the Carotid Stenosis Trialists Collaboration, a meta-analysis of three RCTs with n = 1,557, reports that cerebral embolic protection device (CEPD) were unable to reduce the rate of stroke and death at 30 days (RR 1.1; 95% CI 0.71-1.70, p = 0.67).^290^ A meta-analysis of 13 RCTs, and of 193 registries (n = 54,713) and 22 studies (n = 11,655) reports lower rates of perioperative stroke and death, favoring CEPDs (OR 0.57; 95% CI 0.43-0.76, p < 0.01).^291,292^ The German national registry (n = 13,086) corroborates these findings, with lower rates of severe stroke and death (RR 0.60; 95% CI 0.43-0.84) or of any stroke (RR 0.57; 95% CI 0.43-0.77) with the use of protection devices.^293^ Using multivariate logistic regression analyses, adjusting for baseline differences between groups, the previously mentioned SVS-VQI retrospective study (n = 10,074) found a higher rate of stroke and death at 30 days when CEPDs were not used (OR 3.97; 95% CI 2.47-6.37).^287^ Currently, two forms of CEPD are available in Brazil: proximal occlusion and distal filters.^290^ Flow reversal systems have been discontinued and are no longer marketed in Brazil, meriting only being analyzed superficially.

### Distal filter devices

Filters are unquestionably the most widely used cerebral embolic protection devices in ET of carotid artery disease. Differences in filter construction, type and mesh diameter, as well as design and anchorage, lead to perform differences in terms of cerebral embolic protection.^290-295^ After the publication of the *Centers for Medicare and Medicaid Services* and the AHA/American Stroke Association recommendations, CEPD use during CAS became standard. Both guidelines are based on data showing distal CEPDs reduce the risk of stroke or death at 30 days after CAS by up to 67%, as well as the incidence of new ipsilateral cerebral injuries in MRI by up to 27%.^294,295^ The studies assessed for this section are not randomized controlled trials, and so do not have the force of scientific evidence behind them—however, it is still the consensus among specialists that some form of embolic protection should be used during CAS.

### Proximal occlusion devices

The access and the form of cerebral protection work as to change or mitigate some of the potential risks of CAS, especially in symptomatic patients and those over the age of 80, reducing perioperative cerebral embolization. In a meta-analysis of two different proximal occlusion devices with 2,397 patients from six databases assessing 30-day SAE, including ischemic stroke, AMI and death, the incidence of ischemic stroke was 1.71%, AMI was 0.02%, and death was 0.40%. The composite primary endpoint at 30 days was 2.25%. Age and diabetic status were found to be the only significant independent risk predictors; however, total stroke rates remained below 2.6% in all subgroups, including symptomatic octogenarians. The other baseline demographic variables including patient gender, symptomatic status, and contralateral carotid occlusion were not found to be independent risk predictors.^296-298^ Currently, only one proximal occlusion device is available in Brazil.

The decrease in embolic signals during lesion crossing, as observed in transcranial Doppler ultrasound when compared to the use of filters, also suggests that proximal cerebral protection is more effective. Though some studies have described the reduction of perioperative events in CAS, the results of CEPD use do not have the level of evidence of a randomized trial.^297^ It is worth noting that proximal cerebral embolic protection devices should not be used in patients with proximal common carotid injuries or extensive carotid stenoses, external carotid artery disease and patients with contralateral occlusion uncompensated by other arteries.

### Recommendation 6.4.1

The use of cerebral embolic protection devices in patients undergoing carotid stenting is recommended in order to reduce cerebral embolization (IIa/C).^288-291^

### Recommendation 6.4.2

The cerebral protection system (filter, proximal flow occlusion, flow reversal) to be used in ICA stenting should be chosen at the surgeon's discretion (IIa/B).^294-296,298^

### Recommendation 6.4.3

Proximal cerebral protection devices should not be used in patients with extensive or proximal common carotid injuries, external carotid artery disease or controlateral occlusion uncompensated by other arteries (III/C).^296,298^

### Transcarotid access for flow reversal devices

The TCAR procedure currently in use in the United States uses proximal carotid artery approach with dissection in a healthy segment of the artery at the base of the neck.^299,300^ Cerebral protection uses proximal common carotid artery (CCA) clamping plus flow reversal via an extracorporeal circuit from the CCA to femoral vein or ipsilateral jugular vein.^301^ This combines the advantages of proximal occlusion devices to bypassing the aortic arch, thus reducing the risk of embolization. In the study by Kashyap et al.^242^ there was a 33% decrease in new ischemic cerebral injuries in diffusion-weighted MRI after transfemoral CAS compared to 13% for TCAR (p = 0.03).^242^ ROADSTER-2 is a prospective registry, assessing patients at high risk for CEA with symptomatic stenosis ≥ 50% or asymptomatic stenosis ≥ 80%. Out of 692 patients in the study, 11 failed to meet inclusion criteria and were excluded. In addition, 48 patients underwent TCAR but were discovered post procedurally to have not started or to have discontinued their medications; they were also excluded. Of the 632 patients left, the high risk criteria for CEA were: 44% anatomic, 32% clinical risk, 24% both. The technical success rate was 99.7%. Among the per-protocol population, there were four strokes (0.6%), six AMIs (0.9%), and one death (0.2%), totaling a composite stroke/death at 30 days of 0.8% and a stroke/death/AMI rate of 1.7%.^242^

In a systematic review of TCAR including 18 observational studies with 2,110 patients (technical success rate 98.25%), the 30-day major ischemic stroke rate was 0.71%, the minor ischemic stroke rate was 0.90%, the AMI rate was 0.57%, and cranial nerve injury occurred in 0.28% of the procedures. Conversion to CEA was required for 1.04% of cases.^301^ In another systematic review of TCAR featuring 45 studies, 14,588 patients who met predefined eligibility criteria were included in the meta-analysis. The technical success rate was 99% (95% CI 98-99%). The reasons for technical failure included an inability to cross the lesion and/or failure to deploy the stent. Access site complications occurred in 2% of all cases (95% CI, 1%-2%; 30 studies). The incidence of cranial nerve injuries was only 33 of 8,994 patients. Hemorrhagic complications were reported by 20 studies and occurred in 2% (95% CI, 1%-3%) of all cases. The overall periprocedural stroke and all-cause mortality rate was 1.3% and 0.5%, respectively. In-stent restenosis was observed in four of 260 patients (1.5%; 7 studies), and early (30-day) reocclusion or acute thrombosis of the stent occurred in 12 of 1,243 patients (∼1%; 11 studies), leading to the conclusion that the procedure is associated with low rates of stroke and neurological injury.^302^

Part of the difficulty of this transcarotid overlaps with those for CEA: due to the need for surgical dissection, though at the base of the neck, hostile necks (previous cervical irradiation, kyphosis, morbid obesity, immobility) or even high surgical risk may cause difficulties. Other factors are inherent to the technique itself: calcified plaques, short and small carotid artery (diameter under 6 mm and distance of less than 5 cm from arterial puncture to carotid bifurcation).^242,299-302^

### Radial/brachial access

The RADCAR (RADial access for CARotid artery stenting) study randomized 260 patients to transradial (TRA) or transfemoral access (TFCAS). Procedural success was 100%, the crossover rate was 10% in the TRA to TFCAS group and 1.5% in the TFCAS to TRA group (p < 0.05). The rates of access-site complication were low (0.9% vs. 0.8%), as well as the rates of cardiac complications and/or cerebral events (0.9% vs. 0.8%), but the radiation dose was significantly higher in the TRA group.^269^

Carotid stent deployment via the radial artery, especially in the left carotid artery, is not always possible.^303^ In a multicenter study involving 214 patients, deployment of distal filters was not possible for 7% of patients, while proximal protection was not possible for 1.6%.^304^

In a study of 23,965 patients undergoing CAS in the VQI database, a transbrachial or transradial approach was employed in 819 patients (3.4%), while the transfemoral approach was used in 23,146 patients (96.6%). Anatomic features were more frequent for males (69.4% vs. 64.9%, p = 0.009). On univariate analysis, patients with transbrachial or transradial approach experienced higher rates of adverse outcomes. After adjusting for potential risk factors, there were no differences in stroke or death (OR 1.10 [0.69-1.76], p = 0.675); however, there was a twofold increase in risk for in-hospital myocardial infarction (OR 2.39 [1.32-4.30], p = 0.004) and a twofold increase in risk of technical failure (OR 2.21 [1.31-3.73] p = 0.003] compared to the use of transfemoral access. The use of transbrachial or transradial access was also associated with a 50% reduction in the risk of access site complications (OR 0.53 [0.32-0.85], p = 0.009).^305^

### Recommendation 6.5.1

The following factors should be considered unfavorable for transfemoral CAS: age > 75 years, extensive plaque calcification (> 13-15 mm) or situations in which two or more stents are required, carotid artery tortuosity, and type 3 atherosclerotic plaques in the aortic arch (II/B).^242,301,304^

### Recommendation 6.5.2

Radial/brachial access should be considered as an alternative for transfemoral access for CAS, particularly for right carotid artery injuries, and left carotid with bovine arch anatomy (IIa/B).^269,303,304^

### Recommendation 6.5.3

The following factors should be considered unfavorable for TCAR: hostile neck, previous cervical irradiation, kyphosis, morbid obesity, immobility, long and very calcified plaque near the clavicle (< 5 cm), small-diameter carotid artery (< 6 mm), and high clinical risk (III/C).^242,299-301^

### Recommendation 6.5.4

Transcarotid artery revascularization should be considered as an alternative to transfemoral stenting when stents increase the risk of complications (IIa/B).^242,299-301^

### Stent mesh design

#### Open-cell and closed-cell stents

Stents consist of cells between metal struts and can be categorized into two types: closed-cell, characterized by small free cell areas; and open-cell, with larger free cell areas. The consequences to these structures include stent flexibility and support. Rigid closed-cell stents are more prone to kinking, while plaque debris tends to protrude, by extrusion, through the larger cells of more flexible stents.^306^

There are conflicting results for open-cell compared to closed-cell stents. Results from studies with significant sample sizes point in opposite directions. In a German registry with n = 13,086, with 4,356 patients with open-cell stents, 6,554 with closed-cell stents, and 1,416 with hybrid stents, there was non-significant trend of lower in-hospital stroke and death rates for closed-cell stents (2,3% vs. 2.8% RR 0.86; 95% CI 0.65-1.14, p = 0.30).^307^ In the SVS-VQI database with 1,384 CAS procedures using closed-cell stents and 1,287 using open-cell stents, multivariate analyses found that closed-cell stents were associated with higher rates of stroke and death when used in carotid artery bifurcation (OR, 5.5; 95% CI, 1.3-22.2, p = 0.02).^306^ In a metaregression with n = 46,728, the risk of stroke/death at 30 days after CAS was similar for open-cell and closed-cell or hybrid stents. The result persisted at 1-year follow-up. Open-cell stents are associated with a significantly higher chance of developing postprocedural new ischemic lesions on diffusion-weighted MRI (RR 1.25; p = 0;03), with no differences in the incidence of restenosis, stent fracture, or intraprocedural hemodynamic depression.^306^

#### Double-mesh stents

Double-mesh stents are self-expanding nitinol devices with an inner mesh of various materials (Chart 10). Pore sizes when the stent is fully expanded range from 150 to 180 µm, close to the diameter of some CEPD meshes. Their purpose is to establish a barrier to prevent plaque protrusion and possible cerebral embolization.

**Chart 10:** Proximal occlusion devices available in Brazil in 2023.

**Device and manufacturer**	**Studies**	**30-day stroke rate**	**Class/Level of evidence**	**Relevant refereces**
Mo.Ma^®^ Medtronic	Bersin ARMOUR trial Stabile	0.92% - 2.30%	IIb/B	Ansel et al.,^296^ Kassavin and Clair,^297^ Stabile et al.^298^

A systematic review and meta-analysis of clinical studies of first generation (single layer) and second generation (micromesh — double-layer) was published. Casper^®^ and CGuard^®^ stents of that design are available in Brazil. The analysis included data from 68,422 patients from 112 eligible studies and compared the results for the two groups. The 30-day stroke and death rate were lower for Casper^®^ (1.33%) and CGuard^®^ (1.08%), (2.78 and 3 absolute percent, p = 0.02 and p < 0.001) compared to 4.11% stroke or death rates for first-generation stents. At 12 months, in relation to single-layer stents, Casper^®^ reduced ipsilateral ischemic stroke (-3.25%, p < 0.05) but increased in-stent restenosis to 7.16% (+3.19%, p = 0.04). CGuard^®^, in turn, showed a reduction in both ipsilateral ischemic stroke (-3.13%, p < 0.01) and in-stent restenosis to 0.34% (-3.63%, p < 0.01), compared to 3.97% for single-layer stents.^308,309^

In a publication from three high-volume Italian centers, 150 patients were treated with Casper^®^ stents. Intraprocedural optical coherence tomography (OCT) evaluation was performed in 26 patients, with an off-line analysis by a dedicated core laboratory. All patients underwent duplex ultrasound and neurological evaluation at 24 hours and at 30 days. CAS was technically successful in all cases, and no in-hospital cerebral events were observed at 30 days. OCT evaluation detected a low rate of plaque prolapse (two patients, 7.7%). Duplex ultrasound showed stent and external carotid artery patency in all cases both before discharge and at 30-day follow-up.^309^

The IRONGUARD Study enrolled 733 consecutive patients undergoing CAS using the CGuard^®^ embolic prevention system in 20 centers. An embolic protection device was used in 731 (99.72%) patients. Procedural success was 100%, technical success was obtained in all but 1 (99.86%) patient, who died in hospital due to a hemorrhagic stroke. Six TIAs, 2 minor strokes, and 1 AMI occurred during in-hospital stay (0.82%). External carotid artery occlusion was evident in 8 (1.09%) patients. Between hospital discharge and 30-day follow-up, 2 TIAs, 1 minor stroke, and 3 AMIs occurred. The cumulative stroke rate was 0.54%.^309^Chart 11 shows the structural differences between currently available double-layer stents.

**Chart 11:** Structural differences between double-layer stents.

**Differences in materials**	**Casper®**	**CGuard®**
Structure of nitinol stent	Closed cell	Open cell
Mesh material	Nitinol	Polyethylene terephthalate
Mesh design	Woven	Knitted
Mesh position	Inner mesh	Inner mesh

## VERTEBRAL ARTERY DISEASE

### Introduction, management of asymptomatic individuals and vertebrobasilar insufficiency patients

The establishment of guidelines with high levels of recommendation and evidence for vertebral artery (VA) disease clashes with the fact that there are no randomized clinical trials comparing therapy modalities for these vessels, both for clinical-pharmacological treatment and for endovascular and direct surgical therapy.^5^

The vertebrobasilar vascular system has countless particularities, including major collateral circulation, in large part due to the peculiarity of being the only site in human anatomy where two arteries combine into a single trunk rather than branch off. The two vertebral arteries combine at the V4 segment at the base of the skull to make up the basilar trunk. Therefore, vertebral artery obstructive disease is usually compensated by the other artery, as long as it is patent. Embolic phenomena, even when present, are not as common as for the carotids.^310^

In a major prospective study, Compter et al.^311^ found asymptomatic VA stenoses in 282 (7.6%) out of 3,717 arterial atherosclerotic disease patients. During a follow-up of 4.6 years, vertebrobasilar ischemic stroke occurred in five of them, with a low annual stroke rate of 0.4%. The data corroborate our personal opinion that asymptomatic individuals with VA stenoses should be placed in OCM and that there are no indications for direct intervention.

Asymptomatic individuals and symptomatic patients with VA disease should receive optimal clinical management with SHT control, smoking cessation, adequate diet, metabolic control (obesity, DM and hyperlipidemia), single or dual APT, and, ultimately, anticoagulation therapy.^5^ Similar measures are adopted for carotid artery disease patients.

### Recommendation 7.1

Asymptomatic individuals and symptomatic patients with VA disease should receive OCM (I/B).^5^

The low incidence of conversion from asymptomatic individuals into vertebrobasilar insussiciency (VBI) patients does not justify prophylactic intervention for asymptomatic VA disease.^311-313^

### Recommendation 7.2

Individuals with asymptomatic obstructive vertebral artery disease should not be treated prophylactically with invasive procedures (III/C).^311-313^

Even so, approximately 20% of cerebral ischemic events are estimated to occur in vertebrobasilar (VB) territory.^314^ There are multiple causes, but obstructive VA and basilar artery disease are responsible for approximately 20-25% of the total. The rest are caused by AF (25%), thrombosis/microemboli in intracerebral arteries (also 25%), and other causes, such as obstructive supra-aortic trunks (SAT) disease. Previously, vertebro-basilar (VB) symptoms were primarily attributed to hemodynamic causes, but most cases are now known to be embolic.^314-316^ In relation to the clinical condition for VA disease and VBI, we suggest reading the recently published book chapter on the subject.^310^

### Diagnostic imaging

The modality of choice for examinations of the VB system is MRA, which enables us to survey patients from the aortic arch to intracranial circulation, as well as providing more accurate information about cerebral parenchymal involvement.^317-319^ CTA offers the best spatial resolution to study blood vessels, but inadequate information about brain tissue. These methods offer accuracy levels above 95% for detecting stenoses and obstructions of large- and medium-sized vessels, and have come to replace digital subtraction angiography as the gold standard for VB circulation.^317-319^ CDU tests are widely used to assess the vertebral artery origin, identifying VA stenosis, occlusion, hypoplasia and aplasia, but does not provide more accurate information about the rest of the artery. Accuracy levels for the proximal segment with CDU examination is of approximately 70%, even in recent studies.^318,320-322^ The primary advantage of CDU is found in dynamic real-time diagnosis of subclavian steal syndrome.^320,323,324^

### Recommendation 7.3

Patients suspected of vertebrobasilar ischemia should preferably undergo MRA of the aortic arch, cervical and intracranial arteries along with evaluation of cerebral parenchyma. A similar study using CTA is a valid second choice (I/B).^317-319,323^

### Interventional treatment of symptomatic VA disease

As discussed above, currently there is no indication for prophylactic treatment of obstructive VA injuries in asymptomatic individuals.^311-313^ The symptoms most frequently attributed to VBI are dizziness and vertigo, especially related to sudden head and neck movements, spontaneous events, physical exercise, or chiropractic maneuvers. Systematic studies have found no significant changes to blood flow using various measurement methods, including transcranial Doppler ultrasound during cervical spine rotation within normal therapeutic limits.^325,326^ The studies suggest investigating other causes for symptoms, and that imaging methods only be employed if physical examination reveals alterations suggesting obstructive arterial disease of the supra-aortic trunks.

Since interventions for VBI treatment are only indicated for symptomatic patients, it is important to understand the natural progression of the disease: Gulli et al.,^327^ in a recent prospective 90-day follow-up study after TIA or ischemic stroke in VB territory, found a 7% risk of recurrence in the absence of VA disease, 16% for those with extracranial VA stenosis, and 33% for intracranial stenosis. The findings confirm our impression, and that of many researchers, that patients with VBI should undergo protocols similar to those used with carotid artery ischemia patients.

### How to treat VBI patients with persistent symptoms despite OCM?

Currently, most patients receive endovascular treatment, though the evidence regarding their effectiveness is marginal. A critical analysis of recently published large-scale studies shows that results favor ET of the proximal VA, but with no major statistical significance. In the intracranial portion, however, the statistics clearly favor OCM over an endovascular approach, and the latter is not recommended.^328-333^

### Recommendation 7.4

Patients with persistent VBI symptoms after OCM with evidence of 50-99% stenosis of the proximal VA may undergo endovascular treatment, taking into account its limitations (IIa/B).^328-333^

Several recent studies recommend the use of drug-eluting balloon-expandable stents to treat VA origin injuries.^334-336^ The publications state that long-term patency outcomes are better than when bare metal stents are used, with better results in terms of recurrence of symptoms and need for reinterventions.^334-336^

### Recommendation 7.5

In ET of proximal vertebral artery stenoses, preference should be given to drug-eluting stents (IIa/C).^334-336^

Though surgical techniques to treat proximal VA and subclavian injuries have existed for over 60 years, and to treat distal cervical VA for approximately half a century, there are still few significant studies, as well as several smaller experiences with a wide range of results, some bad, and the discussion of this disease in most guidelines published by major associations is of questionable quality.^5,8,63,154^ The only one to recommend surgical treatment for proximal VA lesions are the American is the SVS guidelines, for low-surgical risk symptomatic patients.^9^

There are many forms of VA open surgical repair, and they all require surgeons with microsurgery skills.^310^ In the proximal segment, the most common technique is common carotid artery transposition; in the distal segment, V3, devalvulated great saphenous vein bypass grafts are recommended, with the common or external carotid arteries as sources.^310^ In this section we analyze four surgical revascularization studies with over 50 patients each, authored by Habozit, Berguer, Kieffer, and Ramirez.^337-340^ In them, with over 1,339 surgery patients, the rate of SAE (stroke/death) was 2.3% for proximal vertebral revascularization, 3.0% for distal, at the base of the skull, but increased to 7.7% for vertebral artery-carotid artery surgery.^337-340^ Though smaller studies have excellent results, including zero mortality in the latter scenario (which coincides with our experience), the numbers from larger-scale results overall recommend against vertebral artery-carotid artery surgery.^341,342^

### Recommendation 7.6

Patients with persistent VBI symptoms after OCM with contraindications for ET can undergo open surgical repair with VA reconstruction (IIa/C).^337-342^

### Recommendation 7.7

For patients with symptomatic carotid artery disease and vertebral artery disease, simultaneous vertebral artery-carotid artery surgery is not recommended (III/C).^337-340^

## CONCOMITANT MULTIFOCAL ARTERY DISEASE

Atherothrombosis may be a local process, but once diagnosed, it presents more often as a multifocal disease. The coronary arteries are one of the most frequently affected sites, and the carotids are often impaired simultaneously. Physicians have long known about the issue of concomitance, the subject of an excellent study by Schlosser et al.,^343^ who estimate that 3 to 8% of all coronary patients also suffer from carotid artery disease. The reverse is much larger: 35 to 40% of carotid obstruction patients suffer from obstructive coronary artery disease.

**Figure 1:**
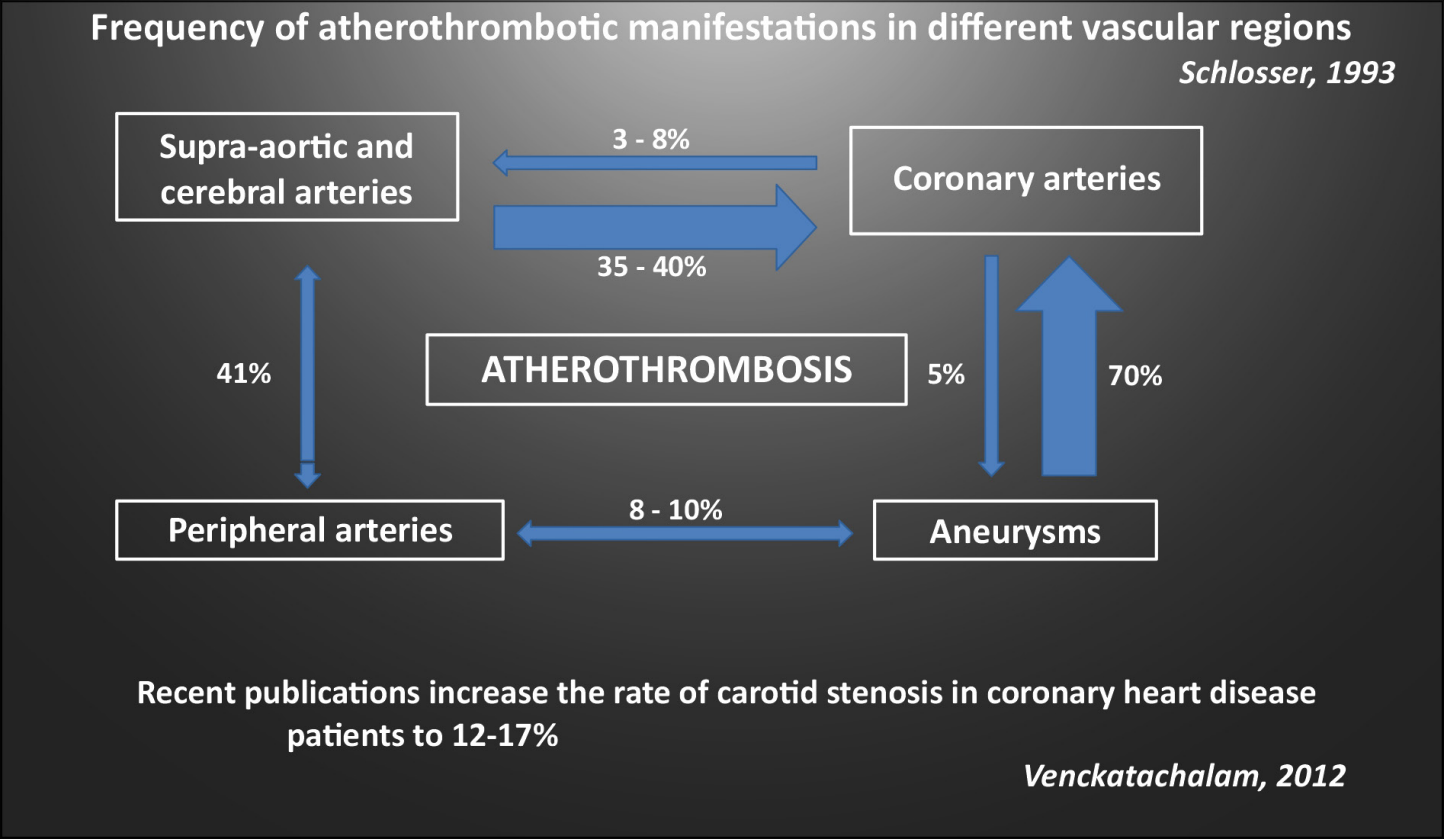
Frequency of atherothrombotic manifestations in different vascular regions.

Venkatachalam et al.,^344^ in a more recent assessment, raised the rate of carotid artery stenoses in coronary patients to 12-17%. Figure 1 summarizes these findings, including PAD and aneurysms.

In this section of the Guideline, we discuss concomitant carotid and coronary artery disease, the interaction between obstructive carotid artery disease with non-cardiac surgery, and obstructive artery disease of the brachiocephalic trunk and common carotid artery.

### Concomitant carotid and coronary artery disease

In the presence of severe coronary and carotid artery disease (SCCAD), CEA and coronary artery bypass graft (CABG) have been performed sequentially or synchronously for over 40 years. Controversy has been a hallmark of the work on the management of SCCAD from the earliest publications on the subject, in which Bernhard (1972) has pride of place, down to the latest studies.^344,345^ Approximately one decade ago, CAS was added to the discussion.^346-348^ Deciding on an optimal strategy—the safest, simplest, and shortest (in length of treatment)—remains a challenge.

There are four basic groups of patients with severe concomitant carotid and coronary artery disease:

1. asymptomatic in both territories;
2. with isolated cerebrovascular symptoms;
3. with isolated coronary symptoms;
4. symptomatic in both territories;

All are potential candidates for revascularization of both territories. But which patients benefit? How to proceed, and when? Traditionally, there are three strategies: first CEA, followed by CABG; combined CEA and CABG; and staged CABG followed by CEA. More options have joined the controversy recently: performing CAS weeks before CABG, performing CAS immediately before CABG or even CABG without carotid artery revascularization, regardless of the degree of carotid stenosis.

Over the last 50 years, we have accumulated an incredible wealth of information on this subject, with thousands of patients and management experiences, but the truth is that all approaches have drawbacks. Adding procedures to carotid surgery seems to place the patient at higher risk of perioperative ischemic cerebral complications, AMI, and/or death. The incidence of stroke after CABG is known to oscillate between 1 and 2%, but in the presence of severe obstructive disease the rate of stroke and brain death can reach as much as 17%.^343,344,349-351^

There are many causes of perioperative stroke during CABG: air embolism, calcium, aortic atheromatous debris, extracorporeal circuit debris, and thrombi. Along with aortic dissection in cannulation, these are responsible for approximately 70% of events, followed by hypotension during extracorporeal circulation (ECC), the cause of the remaining 30%.^352^ If carotid stenosis were the most important cause of strokes during heart surgery, cerebral infarctions should always be ipsilateral to a severely injured vessel. Several studies confirm that this is not the case.^343,344,349,350^ A severely stenotic or occluded carotid artery may in fact reduce embolization to the affected hemisphere.^345^ After cannulation, during ECC, cerebral embolization seems unlikely. Perioperative hypotension seems to have an important role, reducing flow through stenotic arteries and favoring thrombosis. Since hypotension prevention during ECC is incomplete, all patients with severe carotid stenosis would be at risk. So why are concomitant SCCAD patients at higher risk of stroke and death? Probably because this subgroup of patients has higher rates of multifocal atherothrombotic disease, which increases the overall risk of procedures. As we have seen, the two relevant pathophysiological mechanisms in this situation are hemodynamic changes and embolisms.

According to the literature, patients with prior cerebral ischemic events requiring CABG are at higher risk of perioperative stroke when undergoing heart surgery—18% of those with 70-99% carotid stenosis, and as much as 26% for those with bilateral stenoses (or ICA occlusion).^352,353^ The presence of asymptomatic carotid stenosis did not significantly increase the rate of post-CABG stroke, however, regardless of degree of obstruction.^354,355^ Illuminati et al.,^356^ in a randomized controlled trial, evaluated synchronous and staged CEA and CABG in individuals with asymptomatic 70-99% carotid stenosis individuals undergoing heart surgery. The data shows that when CEA was performed first, and when CEA and CABG were synchronous, mortality rates were the same (1%), but when CEA was performed later, the rate of stroke and death rose to 4%. However, when CEA was delayed and performed after CABG, the rate of stroke and death was 9%. Evidently, symptomatic patients with SCCAD should be treated for carotid artery disease before or at the same time as they undergo CABG. But what are the results of various therapeutic options for coronary patients with indication for CABG who have asymptomatic carotid injuries?

And which therapeutic modality, CEA or CAS, offers the best results for SCCAD patients, whether symptomatic or asymptomatic?

CAS only has applications for patients in emergency situations if the method is used concurrently with CABG, since a prior endovascular procedure requires the use of dual APT for a period of at least one month, making CABG prohibitive due to the risk of bleeding, or delays CABG, harming the patient. In the extensive review by Paraskevas et al.,^349^ a meta-analysis of 2,727 patients undergoing staged or same-day CAS+CABG, the stroke and death rate was 7.9%. Most carotid stenoses (80%) were asymptomatic.^349^ For patients with prior history of cerebral ischemia, regardless of degree, had a 30-day stroke rate of 15%. The data contraindicate CAS in emergency scenarios. If performing CAS with CABG is actually required, preoperative antiplatelet therapy with ASA should be maintained, and clopidogrel begun 12 hours after the procedure.

In their reviews of the subject, D’Agostino et al.^353^ and Naylor et al.^5^ conclude that there is indication for staged or synchronous treatment of carotid injuries with CABG in patients with prior history of TIA or stroke and in individuals with bilateral 70-99% carotid stenoses, or with that level of obstruction associated with contralateral carotid occlusion.

One of the most controversial areas in medical literature concerns the management of patients requiring CABG but presenting with asymptomatic severe carotid stenosis. There are many valuable prospective and retrospective single-center and multicenter registries, many randomized clinical trials, and meta-analyses with thousands of enrolled patients, but no definitive conclusion. Even guidelines published by major associations are in disagreement. Most U.S. publications, including the current SVS guidelines, recommend carotid imaging examinations (CDU at first) for all patients undergoing CABG. CEA and eventually a CAS should be performed before CABG if possible, or concomitantly in case of severe injury in both territories. The guidelines consider 50-99% stenosis as significant.^9^ Management was based on several U.S. studies, but is also complemented by German ones.^9,305,357-360^ There are already multicenter studies from the U.S. indicating there is not statistical difference between synchronous CEA, staged CEA, and CABG (with or without ECC) without an associated carotid procedure.^361-363^ On the other hand, there are significant studies, mostly British, recommending against even examining the carotid arteries of asymptomatic individuals undergoing CABG, since there is not statistically significant difference in outcomes from concomitant and staged procedures.^364-370^ A new perspective has recently joined the fray: the possibility of accessing the common carotid artery through sternotomy for open CAS surgery using flow reversal and stenting immediately before or soon after deploying ECC; therefore, without additional access sites and little extra surgery time, severe carotid stenosis can be treated concomitantly.^371^

Given the extensive clinical experience in the area, individuals with severe concomitant carotid and coronary artery disease are at much higher risk of complications when the condition is prevalent in one of these territories. Obviously, the addition of a carotid artery procedure places the patient at high risk of perioperative complications in during CABG. Therefore, if a carotid and coronary artery approach is indicated, the evidence indicates one should:

- treat the symptomatic territory first whenever possible;
- whenever feasible, perform CEA before CABG;
- synchronous interventions only in the presence of:
  - unstable angina and severe carotid artery disease;
  - coronary and cerebrovascular symptoms;
- in staged procedures beginning with carotid artery surgery, perform CABG as quickly as possible;
- select cases may undergo CABG without treatment of the carotids.

With absolutely no intention of offering a recommendation, considering the compelling arguments above, the coordinator of this guideline takes the liberty to present here the management approach adopted for 35 years for concomitant carotid and coronary artery disease. We prefer staged surgery whenever possible, with CEA performed under LRA and the patient under light sedation but awake, at least 4 days prior to CABG.^372,373^ In SCCAD patients symptomatic for both territories or severe coronary artery disease with asymptomatic carotid stenosis, with unilateral stenosis greater than 75% or contralateral occlusion, we indicate synchronous surgery. SCCAD patients with bilateral carotid impairment and symptomatic coronary artery disease represent a complicated situation. In these rare cases, we opt for an endarterectomy of the most obstructed carotid artery along with CABG. If symmetrical, we opt for the dominant hemisphere first; the second carotid artery is treated as soon as the patient’s clinical condition allow. Individuals with asymptomatic carotid stenosis greater than 75% detected during the CABG preoperative period are treated with synchronous surgery. The cardiac and vascular surgery teams need to work in tandem to ensure the best outcome for the patient. This management approach is widely corroborated in the literature.^9,305,357-360^

### How to perform a simultaneous CEA and CABG procedure?

Though the individuals involved in this topic do not completely agree about the management approach due to its specificity and the lack of publications on the subject, we will summarize our management and surgical tactics for synchronous procedures, standardized since 1988: in the presence of asymptomatic or symptomatic carotid artery disease with surgical indication, and in the presence of asymptomatic coronary artery disease, patients initially undergo a carotid procedure, preferably awake and under LRA and light sedation.^372-374^

Surgical tactics have been standardized since our first cases: under general anesthesia, while cardiac surgeons collect and prepare the saphenous veins or other autologous grafts, we access the carotid artery. Later, the cervical approach extends to sternotomy and to preparation of the internal thoracic arteries. Meticulous dissection and hemostasis by cauterization are key. After completing the access sites and obtaining the grafts, the patient is heparinized with 1.2 mg/kg of body weight and the carotids are sequentially clamped; longitudinal arteriotomy involving the entire extent of the plaque to be removed is performed, and a temporary internal shunt implanted; next comes endarterectomy of the whole injury under magnification; all visible plaques are removed, detailed finishing (end point). Closure of the carotid arteriotomy is usually performed with a proximal saphenous vein patch, harvested by the cardiac surgery team.

This is absolutely the most controversial section of this guideline. The number of studies discussed and their often conflicting conclusions are proof that a decision has to be left for a later moment.^9,282,305,357-369^

In addition to CABG, other cardiac procedures have significant rates of in-hospital stroke, and the perioperative period is always the riskiest. Rates of stroke vary depending on specific procedures: the incidence for CABG with concomitant valve replacement ranges from 4.2 to 13%, while for percutaneous aortic valve replacement (PAVR) the rate can be as little as 3%.^5,343,344^ The mechanisms are the same as described above, and the management approach in terms of the carotids is also similar.^5^

### Carotid artery disease and other non-cardiac surgeries

Carotid stenoses with high degrees of obstruction are known to be associated with a high risk of ischemic stroke.^268^ Theoretically, any asymptomatic individual with major carotid artery stenosis may be at risk when undergoing any form of surgical procedure, which always involve emotional stress, BP changes, and often volemic factors, among others.

### How can we identify which patients are at risk?

The criteria for indicating interventions for asymptomatic individuals with stenoses 70% and greater would lead to an excessive number of unnecessary interventions. Are the intervention costs justified? What happened to common sense?^370,375-383^

We are often confronted with questions about the need for carotid interventions before various types and categories of procedures for individuals with asymptomatic carotid stenoses, due to the concern with perioperative ischemic strokes. In general, surgical risk is very low—0.2%. It rises to 0.5% for patients with carotid bruit, to 2.9% for patients with prior ischemic stroke, to 3.6% for those with symptomatic carotid stenosis, and up to 6% for those with vertebrobasilar symptoms.^282,364-388^

From the Schlosser and Venkachathalam studies mentioned above, we know that there is a high rate of > 70% carotid stenosis in patients with abdominal aortic aneurysm and aortoiliac PAD (AI-PAD).^343,344^

PAD patients have higher rates of major concurrent carotid artery disease (14-49%). This prevalence is directly related to PAD stage as well as age: for individuals below 50, it is almost zero, but it reaches 41% for those over the age of 80. In 2008, the Coordinator of this guideline conducted a survey of his records and found 166 AAA patients and 184 AI-PAD patients. In AAA patients, a retrospective study found major carotid stenoses in 8.3% of asymptomatic patients and 73% of patients with prior ischemic strokes. In AI-PAD patients, the rate was 13.3% for asymptomatic individuals and 60% for prior ischemic stroke patients (Arno von Ristow, unpublished data).

The most important markers of obstructive carotid artery disease are age over 70, history of prior ischemic stroke, history of coronary disease, PAD and aortic aneurysm, as well as low ankle-brachial index and DM. Based on these risk factors, patients in any of these groups should undergo SAT examinations, primarily CDU.^282,343,344,375-388^

### Recommendation 8.1.1

Asymptomatic cerebrovascular disease patients with indication for elective procedures in general and no known risk factors for atherothrombotic cerebrovascular disease may undergo procedures without specific physical examinations or SAT imaging examinations (I/A).^370,375-383,388^

### Recommendation 8.1.2

Asymptomatic cerebrovascular disease patients with indication for elective procedures in general, but with one or more risk factors for atherothrombotic cerebrovascular disease (age over 70, history of prior ischemic stroke, history of coronary artery disease, history of PAD, low ankle-brachial index, and DM) should undergo tests for carotid artery disease, with specific physical examinations and SAT CDU tests (I/A).^281,343,344,370,375-388^

The meta-analysis by Jørgensen et al.^389^ confirms that out of 7,137 surgery patients, usually within 3 months of cerebral ischemia (out of 481,183 individuals), 11.9% had ischemic strokes after surgery. The rate falls to 4.5% for 3-6 months and to 1.8% for six months to 1 year. Most strokes were ischemic, caused by obstructive artery disease, or cardioembolic. For surgery patients with no prior history of stroke, the rate was 0.1%.

### Recommendation 8.1.3

Patients with indication for elective procedures in general who had symptoms of cerebrovascular disease during the previous 6 months should undergo tests for carotid artery disease, with specific physical examinations and SAT CDU tests (I/A).^343,344,370,384-387,389^

### Recommendation 8.1.4

For patients with indication for elective procedures in general who had symptoms of cerebrovascular disease during the previous 6 months related to carotid artery disease for which 50-99% carotid stenoses have been detected, carotid revascularization should be performed before the scheduled surgery (I/B).^384-387,389-391^

In addition to the numerous factors listed above, suspending APT and anticoagulation medications lead to poorer prognoses, and the therapy should be withdrawn for as little time as possible and replaced by short-term agents, such as LMWH or even unfractioned heparin until close to the procedure.^390-392^ Statins do not require the same approach and should not be suspended or their dosage reduced.^390-392^

### Recommendation 8.2.1

Patients with indication for elective procedures in general who are under statin therapy should not have their dosages suspended or reduced before the scheduled procedure (I/B).^390-392^

### Recommendation 8.2.2

Patients with indication for elective procedures in general who are under APT should have their risk of bleeding determined on an individual basis. If temporary suspension of APT due to risk of bleeding is necessary, its use should resume as soon as possible after the intervention, preferably on the following day (I/B).^390-392^

### Recommendation 8.2.3

Patients with indication for elective procedures in general who are under anticoagulation therapy should have their risk of bleeding determined on an individual basis. If temporary suspension of anticoagulation therapy due to risk of bleeding is necessary, additional protection may be obtained by the use of LMWH or unfractioned venous heparin until as close to the procedure as possible, and the usual anticoagulants resumed as soon as possible after the intervention, preferably on the following day (I/B).^390-392^

Guidelines from major international associations disagree about the management of asymptomatic obstructive carotid artery disease individuals for general non-cardiac surgery. The German-Austrian guideline does not discuss the subject.^154^

ESVS does not recommend carotid interventions as prophylaxis for intracranial complications in the presence of asymptomatic 50-99% stenoses.^5^ The American SVS indicates recommends applying the same examination and treatment protocols as for the general population.^9^

### Recommendation 8.4

The treatment of carotid artery disease for individuals with asymptomatic cerebrovascular disease with indication for elective procedures in general who have significant (> 75%) carotid stenoses should be based on the experience and expertise of the group responsible for their treatment. The indication should be the same as for individuals with asymptomatic carotid disease without indication for general elective surgery. If an intervention is indicated, it should preferably be performed before general elective surgical procedures (III/B).^5,9,385^

A frequent question is whether one should suspend DOACs and especially APT before ophthalmologic procedures, specifically cataract surgery. Since the age range for these procedures coincides with a population with many cardiovascular diseases on this class of medications, there is cause for concern for everyone involved—ophthalmologists, cardiologists, vascular surgeons, and the patients themselves. Approximately one in every four patients undergoing cataract surgery were on antithrombotic medications in three large meta-analyses.^393-395^ Meta-analyses with tens of thousands of patients^393-395^ and several well-designed single-center randomized trials suggest phacoemulsification procedures may be performed without suspending APT, as long as the procedure is performed under topical anesthesia and sedation.^396,397^ Retrobulbar anesthesia has higher hemorrhagic risk, and suspending these medications before the procedure is recommended (the risk increases 0.2-1.0%, but does not negatively affect the outcome of the intervention).^393,394^ Some studies recommend the same management approach an anticoagulant of the warfarin group is administered, which is currently unusual, since most patients under anticoagulation therapy now use direct anticoagulant (rivaroxaban, dabigatran, apixaban, and edoxaban). There are no conclusive studies regarding DOACs, but common sense suggests they should be suspended 48 hours before ocular surgery and restarted on the following day, if retrobulbar anesthesia is employed. For patients at extremely high cardiovascular from suspension of APT and DOACs, the recommendation is to perform the intervention without suspending the medication and employing topical anesthesia and sedation.^393-399^

### Recommendation 8.5.1

Phacoemulsification cataract surgeries and intraocular lens implantations for which APT suspension is contraindicated due to high risk of cardiovascular events may be performed under topical anesthesia and sedation (I/A).^393-399^

### Recommendation 8.5.2

In phacoemulsification cataract surgeries and intraocular lens implantations under retrobulbar anesthesia, APT and anticoagulation therapy should be suspended for the period required to interrupt the specific effect of each medication. APT and/or anticoagulants should be restarted on the day following the procedure (I/A).^393-396,398^

### Recommendation 8.5.3

Patients on anticoagulation therapy at high risk of thrombotic events from their suspensions, with indication for phacoemulsification cataract surgery and intraocular lens implantation, may undergo procedures under topical anesthesia without first suspending anticoagulation therapy at low risk of hemorrhagic complications. Access to highly skilled ophthalmology teams is recommended (II/B).^394,395,399^

### Obstructive artery disease supra-aortic trunks

Supra-aortic trunks (SATs) are branches originating directly from the aortic arch: the brachiocephalic trunk (BCT), the left common carotid artery (LCCA), and the left subclavian artery (LSA). In surgical practice, the right common carotid artery (RCCA) and the right subclavian artery, even though the latter is rarely involved in obstructive processes, may also fall under this classification, since in addition to being part of cerebral and upper limb circulation, like the three proximal trunks, they share the same pathologies and surgical issues.^310^ The most frequent cause of SAT obstructions is atherothrombosis. Less frequent causes include arterites (mainly Takayasu's disease and Horton's disease), dissections, aneurysms, and tortuosities associated with stenotic kinking, congenital defects with obstructive impairment (agenesias, Kommerell diverticulum), and others, such as embolisms, actinic damage, fibromuscular dysplasia, ergotism, and mediastinal fibrosis.^310^ SAT revascularization procedures are mainly performed to treat cerebrovascular insufficiency symptoms, upper extremity ischemia and to revascularize an obstructed subclavian artery, in preparation for use of the internal thoracic artery in coronary bypasses.^310^

SAT surgery has two particularities within vascular surgery: many pathologies are involved and their treatment requires high-level skills in cerebral revascularization, thoracic surgery, and, more recently, angioradiology. SAT reconstruction surgery has always been unusual in vascular surgery services, and became even rarer with the possibility of ET for most cases.^310,400,401^ Only a small percentage of cases, between 3 and 7% of revascularization procedures of cerebral circulation arteries, involve SATs.^310,400,401^

### History

The first brachiocephalic trunk (BCT) aneurysm ressection was performed by Oudot in 1952. Bahnson was the first to use a bypass graft to treat obstructive BCT injuries, and Davis the first to perform an endarterectomy of that artery. Cate & Scott performed the first left subclavian endarterectomy in 1957, via thoracotomy. In 1961, North, DeBakey and Crawford performed the procedure via cervical access, and in the same study published the first carotid-subclavian bypass. In 1963, Parrot performed a subclavian implantation into the carotid artery. Next came several transcervical bypass procedures by Warren, Ehrenfeld & Myers.^310,400,401^ The name “subclavian steal syndrome” was suggested by Fischer after Contorni described the clinical condition in 1960. This interesting circulatory phenomenon absolutely raised the interest on vertebrobasilar disease.^310,400,401^

The introduction of endovascular treatment breathed new life into the treatment of obstructive SAT artery disease. Mathias performed the first balloon angioplasty of the subclavian artery in Germany in 1980.^402^ There is some debate about who first performed subclavian stenting, since many deployed the device in various arteries almost simultaneously after its introduction.^400,401^ Currently, ET is the most frequent treatment modality for obstructive SAT disease, sometimes combined with surgery for a hybrid procedure.^310,400,401^

The literature contains relatively few publications with a large number of cases involving treatment of obstructive SAT artery disease, especially aortic arch ostial injuries. Thus, the recommendations in these sections are based on many single-center studies and few multicenter ones, as well as a single systematic meta-analysis.^9,403-410^ Few guidelines from other associations discuss the subject in-depth.^5^

The first recommendation is derived from the expert consensus that asymptomatic SAT injuries do not require surgical or endovascular therapy and should receive conservative treatment. This is the situation of many patients with obstructions of the proximal third of the left subclavian artery; if asymptomatic, they do not require revascularization.

### Recommendation 9.1

Individuals with asymptomatic obstructive SAT injuries do not require surgical or endovascular interventions and should receive conservative treatment. III/C – Expert consensus.

Chronic VA and SAT arterial occlusions can lead to a wide variety of signs and symptoms, often identical to those associated with carotid bifurcation disease. A detailed clinical history and thorough physical examination are the key to detecting these injuries. The clinical condition can present in four basic forms: anterior cerebral ischemia, vertebrobasilar ischemia, upper extremity ischemia, and associated symptoms.^310^ During physical examination, physicians should pay special attention to palpation of carotid, subclavian, and upper extremity arterial pulse. Arteries should be auscultated at the level of the aortic focus, sternal notch, supraclavicular fossa, and in the middle and distal thirds of the neck (angle of the jaw). Bruits are found in most cases (85%), as well as pulses with different amplitudes from the upper limbs (65%).^310^

CDU has been used extensively to diagnose SAT pathologies, often with inaccurate and contradictory results.^5,310^ A valuable assessment requires deep knowledge of anatomy, of the physiology of blood flow, and the pathophysiology involved, as well as an examiner particularly attuned to this possibility. Very useful in assessing the direction of blood flow, often altered in these situations, the determination of the degree of stenosis and the extent of the injuries is still imprecise with this method.

Detailed study of the aortic arch and SATs is essential for surgical planning. CTA progressively replaced angiographic examinations for diagnostic purposes. Although digital subtraction arteriography is still considered the gold standard in evaluating obstructive SAT disease, CTA obtained with multi-detector scanners, combined with different types of image reconstruction, provides accurate images of pathological anatomy and allows assessment of the presence and extent of injuries, vessel dimensions and lumen, as well as characteristics of the vascular wall. Chest CTA significantly helps assess aneurysms and congenital anomalies. It is currently the most complete examination to study SATs.^5,9,310,400,401,403,405,407,409,410^

### Recommendation 9.2

In diagnostic assessments of SAT obstructions, CTA tests, whether or not complemented by digital angiography, is the examination modality that most provides information for therapeutic planning (IIa/A).^5,9,403,405,407,409,410^

Like other territories in our specialty, as for abdominal aortic aneurysms, SAT approaches used open surgery for decades, with excellent results and low morbidity and mortality.^310,406,408^ Over the last 30 years, ET has taken the lead, and now very few cases require a completely open surgical approach.^310,403-405,407,409,410^ There are multiple possibilities of direct surgical reconstruction, and they deserve the attention of extensive publications in this field.^310^ Among studies employing open surgery, we should note the classic paper by Fry et al.^406^ (1992), with 20 subclavian-carotid bypasses, four of which combined with simultaneous CEA. There was one in-hospital death from AMI and no late restenosis at 5 years. Most bypass grafts were made out of autologous saphenous veins at the time, but late degeneration, especially graft lengthening and the improvement of other graft materials, especially ePTFE, make it the most widely used conduits today.^406-408,410^ Another important study was published by Takach et al.^408^ in 2005, with 113 patients treated using a transthoracic approach and 44 with an extrathoracic approach, with mean age of 54 years. Concomitant CABG was performed in 37 patients (23.6%), and concomitant CEA in 26 (16.6%). Surprisingly, there were more strokes in the extrathoracic revascularization group (2.7% vs. 6.8%), but operative mortality was similar for both groups (2.7% vs. 2.3%). Late outcomes were better for the open surgery group (94.4% patency at 10 years vs. 60.3%) regardless of the number of distal anastomoses. Among studies comparing open surgery to hybrid procedures and exclusively endovascular approaches, the following stand out: de Vries et al.,^404^ as early as 2005, reported that ET achieved technical success in 93% of their 110 cases. A femoral approach was employed for 81% of patients, a brachial approach for 5.4%, and a combined femoral and brachial approach for 13.6%. Stents were only deployed for 58% of patients. They report strokes in 2% of cases and morbidity and mortality in 3.6%. 5-year patency: 89%

Reoperation was necessary due to recurrence of symptoms in 7.2% of cases, half of which required open surgery. They call attention to the need for more frequent follow-up for the first 2 years post-ET.^403^ Bakken et al.^403^ (2008) report a 98% technical success rate for ET of the SATs, with zero mortality and major morbidity of 2%. Patency at 3 years was 88%, with a reintervention rate of 7%. Another single-center event was published by van de Weijer et al.;^409^ it enrolled 114 patients with 144 SAT injuries and median age 66.3 years. ET was indicated for 137 injuries, most treated by recanalization, angioplasty and stenting (angioplasty without/with stenting): BCT-9/54; LCCA 0/7; LSA 11/56. There were seven failures, and the intervention was terminated before the procedure could be performed. Technical success was achieved in 94.4%, with zero mortality at 30 days. Patency at 5 years was 83.2%, strongly correlated with the absence of recurrence of symptoms in 82.3% of patients (most within 2 years). Recurrences may be treated by ET again. In diagnostic assessments of SAT obstructions, CTA tests, whether or not complemented by digital angiography, is the examination modality that provides most information for therapeutic planning. Recent noteworthy studies include the one authored by Zacharias et al.,^410^ who published results from outcomes from open, hybrid, and endovascular BCT revascularizations at their center in 2020: out of 33 cases, 64% were symptomatic, while for the remaining 36% the procedure was combined with CEA. In four patients, aorto-innominate bypass was required due to heavily calcified aortic arch injuries involving BCT ostia and the trunk itself, or due to failed endovascular interventions. Cerebral embolic protection maneuvers or devices were used whenever possible, either by distal clamping in hybrid procedures and by distal filters for exclusively endovascular ones. The group had good results—a 97% technical success rate with a single death (3%), caused by stroke/reperfusion. They also achieved excellent long-term patency, with restenosis in 9% of cases.^410^

The systematic meta-analysis of 1,969 patients from 77 SAT revascularization studies over 57 years assessed by Robertson et al.^407^ in 2020 had similar results: 7% morbidity and mortality for open surgery, 3.3% for hybrid surgery, and 1.5% for endovascular procedures. Late restenosis ranged from 2.6 to 10.5%, and the need for lifelong follow-up is also worth noting. The multicenter study based on the U.S. SVS-VQI database published by DeCarlo et al.^405^ in 2021 assessed 18,886 CAS cases, out of which 809 had tandem lesions in the proximal common carotid arteries combined with carotid bifurcation stenosis. The risk of stroke and death doubled for concomitant ET in both lesions (3.4 vs. 1.8%) regardless of whether patients were symptomatic or asymptomatic. They recommend against pure ET for combined injuries.

All of these studies recommend ET for SATs as safe and effective and as the method of choice when feasible. Specific situations may require extra-anatomical or hybrid procedures, including those involving sternotomy, with a small increase in surgical risk and good long-term results.^406-408,410^ Hybrid procedures have better outcomes for SAT injuries associated with severe carotid bifurcation stenosis: ET for proximal injury and CEA for the bifurcation, combined with embolic protection measures.^5,9,310,401,405,407,410^ The literature as a whole highlights the need for more frequent follow-up during the first 2 years after ET, when recurrences are more frequent, and recommend the need for lifelong follow-up, since most patients are younger than the usual arteriopathy patients suffering from atherothrombosis and may survive for longer periods.^403-405,407-410^

### Recommendation 9.3

Patients with symptomatic SAT injuries should preferably be treated with endovascular or hybrid procedures (IIa/B).^403-405,407,409,410^

### Recommendation 9.4

When anatomical, anatomopathological or clinical conditions prevent ET, obstructive SAT injuries in symptomatic patients may be treated with extra-anatomical or open surgical procedures (I/B).^405,406,408,410^

### Recommendation 9.5

Hybrid procedures have better outcomes for SAT injuries associated with severe carotid bifurcation stenosis: starting with ET for proximal injury and CEA for the bifurcation, combined with embolic protection measures (I/B).^5,9,310,401,405,407,410^

Current medical knowledge and available diagnostic methods now enable precise diagnosis and highly accurate prognosis. Ischemic stroke and HS prevention methods are efficient and cost-effective. Asymptomatic individuals in known risk groups should be carefully assessed, and those at high risk for cerebral ischemia should be treated specifically for this condition if they have access to health care services with minimal rates of complications.

## COMPLICATIONS

### Complications of open carotid surgery

Surgical treatment by CEA has had a relevant role in the treatment of atherosclerotic disease of the extracranial carotid artery since the 1950s. Over seven decades, every aspect of CEA has been thoroughly studied—indications, techniques, and complications. The vast literature on the subject enables us to analyze the complications associated with CEA and their prevention.

CEA complications may be categorized as:

- anesthetic and hemodynamic;
- surgical;
- vascular;
- neurological.

### Anesthetic and hemodynamic complications

General or locoregional anesthesia may be used in CEA. General anesthesia is the original technique, used since the 1950s. The use of cervical locoregional block (CLB) for CEA began in the 1970s. Both techniques are widely used. Few services use local infiltration anesthesia with mild sedation to monitor the patient's neurological status throughout the procedure.^411^

### General anesthesia vs. cervical locoregional block

The advantages of general anesthesia are: airway control throughout the procedure, no patient anxiety, enabling use of neuroprotective anesthetics (e.g., propofol, isofluorane), and better BP control. The drawbacks are: myocardial depression with hypotension, need to monitor cerebral ischemia during carotid clamping, higher rates of myocardial ischemia, and pulmonary complications.^412^

The main advantage of CLB is continuous monitoring of brain perfusion in conscious patients. The drawbacks are patient anxiety and discomfort, which can cause tachycardia and SHT; higher rates of cervical hematomas in patients undergoing dual antiplatelet therapy;^413^ and anesthetic complications, such phrenic nerve blockade and inadvertent subdural anesthesia.^414^ The GALA study,^254^ the largest RCT ever to compare general anesthesia to CLB, and a Cochrane review^415^ found no significant differences for rates of stroke/death at 30 days between the two techniques. However, other meta-analyses of RCTs show that CLB anesthesia significantly reduces postoperative cerebral ischemic injuries, pulmonary complications, cranial nerve injuries, duration of surgery, length of hospital stay, blood loss, and aggregate stroke/death rates at 30 days.^416-422^

Like any other clinical decision, the choice of anesthesia method for CEA should take into consideration anesthesiology care team expertise, clinical condition, and patient preferences.

### Recommendation 10.1

The surgical/anesthesia care team should choose the anesthesia method (general or locoregional block) based on local experience, clinical condition, and patient preferences (IIa/B).^253,415-422^

### Systemic hypertension

SHT, defined as systolic BP > 180 mmHg during CEA and in the immediate postoperative period, has significant influence on the following complications: bleeding and formation of hematomas; heart failure; AMI; acute CHF; intracerebral hemorrhage (ICH); and CHS.^423^ Eversion CEA is associated with significantly higher rates of intra and postoperative SHT than conventional CEA.^424^

Strict BP control during the procedure and the first 24-48 hours afterward is the most important measure to reduce these complications.^215,425^ BP control protocols during the critical period, comprehending the procedure itself and in the immediate postoperative period, are available in the literature.^215^ Though there are no published studies, one can reasonably assume that the risks of uncontrolled SHT also apply to patients undergoing CAS.

### Recommendation 10.2

Strict SHT control during and after CEA reduces surgical, cardiac and neurological complications. BP control protocols should be implemented in centers performing CEA (IIa/B).^215,425^

### Hypotension

There are several possible causes for hypotension during CEA.^426^ When inducing anesthesia, hypotension is caused by the depressive effect of anesthetics on the myocardium. During the procedure, by the level of anesthesia and the use of protamine to reverse the effects of heparin. Postoperatively, a frequent cause is the hypotensive effect of carotid bifurcation baroreceptor stimulation.^427^ In this condition, removal of a rigid atheromatous plaque leads the carotid artery wall to recover its natural pulsatility, strongly stimulating baroreceptors located on the carotid sinus and triggering bradycardia and hypotension. Hypotension may last several days. In series of patients who had hypotension/bradycardia after CEA and CAS, no severe adverse effects or increased morbidity and mortality were found.^428^

### Surgical complications

#### Cranial and cervical nerve injuries

In its path through the neck, the common carotid artery and its two branches, the internal carotid artery and the external carotid artery, cross or run adjacent to five cranial nerves: facial (marginal mandibular branch) (VII); glossopharyngeal (IX); vagus (X); accessory (XI); and hypoglossal (XII). Given the anatomical proximity, cranial nerve injuries are a relatively common complication in CEA, occurring in 5% of all procedures, most frequently to the marginal mandibular branch.^236^ Predictors of cranial nerve injuries are GA, emergency procedures, reoperation for bleeding, and procedures in irradiated necks.^429^

The most frequent injuries happen to the mandibular branch of the facial nerve, the hypoglossal nerve, and the recurrent laryngeal nerve (a branch of the vagus nerve).^430^ Injuries to the mandibular branch of the facial nerve cause deviation of labial commissure. Injuries to the hypoglossal and glossopharyngeal nerves cause difficulty chewing and dysphagia.

Almost all injuries to cranial nerves resolve spontaneously within weeks or months. Injuries to the recurrent laryngeal nerve are the exception, potentially leading to permanent vocal cord paralysis, speech impairments, and difficulty swallowing. Patients with a permanent injury to the recurrent laryngeal nerve should not undergo contralateral CEA.^431^

#### Cervical hemorrhage and hematoma

Practically all patients who undergo CEA are under the effect of antiplatelet agents.^432^ During the procedure, therapeutic doses of IV heparin are administered to achieve full anticoagulation. These two factors, combined with the abundant blood flow in the neck, lead to persistent bleeding and the formation of cervical hematomas, requiring reoperation for 2 to 10% of procedures.^433^ Hematomas nearly always form during the first few hours after surgery, usually in patients with uncontrolled BP. Another cause of persistent bleeding is heparinization, which must be reverted by slow infusion of protamine before wound closure. A meta-analysis of seven studies found that administering protamine reduces the formation of hematomas requiring reoperation by over 50%.^434^

Strict BP control, as stated in Recommendation 10.2, and protamine administration to reverse the effects of heparin, are required to reduce the hemorrhagic complications of CEA.

### Recommendation 10.3

For patients undergoing CEA, protamine should be administered routinely before the incision is closed (IIa/B).^434^

Draining the CEA incision is also a controversial issue. A meta-analysis of six prospective studies found no benefits from routine drain placement.^435^ However, when SHT control and protamine fail to correct persistent bleeding, surgeons should exercise clinical judgment to decide on drain placement.

### Recommendation 10.4

For patients undergoing CEA, selective placement of drains in surgical incisions is recommended (IIa/C).^435^

### Surgical wound infection

Cervical wound infection is rare for CEA. In large case series, infection rates are lower than 1%. Though CEA is categorized as clean surgery, antibiotic administration is a common practice, given the fact that it consists of arterial surgery involving synthetic or biological patch.

### Patch contamination

There is strong evidence that patches should be used routinely when closing carotid endarterectomies.^261^ Patches may be made of autologous veins, sheathes of synthetic material (Dacron or ePTFE), and bovine pericardium. Infection with patch contamination happens in 0.5 to 1% of procedures.^436^ It may take the form of early infection, with inflammatory signs in the area of the operation, abscess formation, and purulent drainage. Cases of chronic infection are more frequent, with discrete inflammatory signs and the formation of a cutaneous fistula at the incision site, with intermittent purulent drainage. The infection contaminating the patch may lead to the formation of a pseudoaneurysm and a catastrophic rupture of the carotid artery.^436^ Treatment of patch contamination consists of reoperation, with resection of the portion of the artery containing the patch and reconstruction of the internal carotid artery with saphenous vein or autologous arterial bypass.^437,438^

For high-surgical-risk patients and/or those with infected pseudoaneurysms, the EndoVAC technique has been proposed.^439^ The technique consists of: 1. relining of the infected reconstruction with a stent graft; 2. surgical debridement of the infected area and excision of the patch without carotid clamping; 3. VAC therapy, to permit granulation and secondary delayed healing, and long-term antibiotic treatment.

### Recommendation 10.5

For patients with patch contamination, reoperation with resection of the portion containing the contaminated patch and reconstruction with autologous vein bypass graft (saphenous veins) or autologous arterial bypass (IIa/B).^436-440^

### Restenosis

During follow-up for patients who undergo CEA, sometimes a new stenosis of the carotid is detected. The incidence depends on the criteria used to define restenosis; in large case series, restenosis rates range from 3 to 12%.^240^ The mechanisms of restenosis are intimal hyperplasia of the endarterectomy site, injuries from arterial clamps and, at later stages, recurrence of carotid atherosclerosis.^284^ Restenoses are usually asymptomatic, and the risk of new ischemic events attributable to them is low.^240,284^

Restenoses should be monitored by periodic CDU. If the stenosis progressively increases to more than 70%, an elective intervention should be considered: new CEA or CAS. The decision should preferably be made by a multidisciplinary team, taking in account age, comorbidities, surgical risk, and team expertise in the two treatment modalities.

Occasionally, patients have ischemic events (TIA or stroke) during follow-up presenting symptoms corresponding to the operated carotid artery. Imaging examinations are required to confirm the presence of restenosis. In case of stenosis under 50%, the patient should be kept under OCM and closely monitored; in case of stenosis greater than 50%, a new CEA or a CAS should be considered.^441,442^

### Recommendation 10.6

For patients with late ischemic events ipsilateral to the operated carotid artery and restenosis greater than 50%, a new CEA or a CAS is recommended (I/B).^441,442^

### Vascular complications

#### Intraoperative ischemia from perfusion deficit

The carotid clamping required for CEA causes a sudden decrease in cerebral perfusion. In patients with complete circle of Willis, collateral circulation by the contralateral carotid artery and VA maintains sufficient blood flow for adequate cerebral perfusion during carotid clamping. However, in approximately 10% of cases, collateral circulation is not sufficient to maintain perfusion to that hemisphere.^442^ In these cases, shunting is required to prevent an ischemic stroke. Shunting may cause complications, such as carotid dissection, thrombosis with distal embolization, and rupture of the internal carotid artery. In patients under CLB, the decision to shunt is based on the neurological status of the conscious patient. In patients under GA, cerebral perfusion monitoring methods are required during clamping. The methods include stump pressure, electroencephalogram, somatosensory evoked potentials, transcranial Doppler ultrasound, transcranial cerebral oximetry, and near infrared spectroscopy.^443^

The literature on methods of monitoring in shunting is vast. A Cochrane review^444^ and a SVS-VQI analysis^260^ found no difference between the various methods of monitoring nor even between selective and routine shunting.^258^

The 2021 European guidelines^103^ and the 2022 U.S. guidelines^9^ agree that there are no statistical differences between routine shunting, selective shunting, and never shunting for CEA outcomes.

#### Recommendation 10.7

In patients undergoing CEA, surgeons may decide whether to shunt or not at their discretion (IIa/C).^9,103,258,260,444^

#### Intraoperative embolization

Cerebral embolization may occur at any stage in CEA. In patients with unstable plaque, carotid dissection itself may trigger cerebral embolism, as detected by transcranial Doppler monitoring.^445^ Embolization may occur during shunting or through the shunt during the procedure.^446^ The highest embolic load occurs during the unclamping of the carotid arteries.^447^

Measures to reduce the likelihood of intraoperative embolization include: delicate dissection of the carotid arteries; full heparinization; shunting only when necessary; complete removal of the atheromatous plaque, without leaving fragments in the endarterectomy bed; elimination of potential debris to the exterior before closure of the arteriotomy; and sequential unclamping, directing antegrade flow initially to the external carotid, before releasing flow to the internal carotid.

#### Perioperative and postoperative thrombosis of the internal carotid after endarterectomy

Carotid thrombosis during or immediately after CEA is found in approximately 3% of procedures.^448^ The causes of thrombosis are: technical failure during endarterectomy, internal carotid stenosis by residual plaque, arteriorrhaphy defects, dissection of distal internal carotid, inadequate heparinization, and thrombophilia with heparin resistance.^449^

Management of perioperative thrombosis consists of immediately detecting and correcting the cause of the obstruction. The condition may be suspected in patients with absent ICA pulse, absent flow in intraoperative Doppler ultrasound or evidence of neurological after waking when GA was employed during the procedure. The patient should not leave the operating room before waking up! Distal internal carotid artery stenosis caused by plaque may be treated by implanting a coronary stent (usually available in all tertiary hospitals), in the distal ICA; technical failures during endarterectomy may be treated by a supplementary procedure (in case of residual plaque) or replacement of the affected portion by an ePTFE graft; arteriorrhaphy defects usually require patch graft angioplasty; distal dissections of the ICA and cranial to the Blaisdell line usually require stenting, as reported above. Coagulation disorders should be corrected with adequate heparinization controlled by activated coagulation time. An intraoperative angiography of the carotid may be necessary, which can be obtained by CCA puncture with a simple scalp needle, to be replaced by a 5F sheath if an intervention is required.^5,9,448-451^

In case of early postoperative neurological deficit, with the patient already in the Intensive Care Unit (ICU), timing suggests etiology. Up to 6 hours after surgery, the most frequent cause is thrombosis of the internal carotid artery or embolism from mural thrombus in the endarterectomy zone. After 6 hours, the causes may include CHS, cerebral edema, intracranial hemorrhage, or even an embolism. Until recently, the recommendation was immediate re-exploration under these conditions. In recent years, the European consensus, followed by other studies and guidelines, suggests that for all patients developing neurological deficit in the early postoperative hours, fast imaging examination of the carotid is required before re-exploration of the surgical site.^5,446,450^

A CDU may be performed at the bedside and, if evidences of ICA obstructions are found, the next step is immediate re-exploration of the surgical site. In case of adequate patency of the carotid axis after surgery, cervical and intracranial circulation should be quickly examined with a CTA.^9,446-448^ The test may establish if the condition consists of an edema, CHS, or incranial hemorrhage and distal thrombosis or embolism. Thrombi in the endarterectomy zone can be removed under direct visualization, and those extending into the internal carotid artery should be gently removed with a Fogarty 3F catheter.^9^ Infusions of thrombolytic agents at small doses may help dissolve thrombi/emboli not accessible for mechanical removal. Doses starting at 3 to 5 mg and reaching as much as 20 mg have been recommend, always accompanied by intraoperative angiography. In centers staffed by neurointerventional specialists, catheter-directed thrombolytic therapy infused into the distal internal carotid artery and MCA is suggested.^451^ Revision of the arteriorrhaphy, with deployment of a patch (preferably a venous patch graft) and maintenance of therapeutic doses of heparin. Some authors suggest the continuous infusion of Dextran-40therapy (currently unavailable in Brazil) for the next 24 hours.^215^

### Recommendation 10.8.1

When central neurological deficit is detected at the end of CEA, the cause of the deficit should be corrected immediately, before the patient exits the surgical ward (I/B).^5,9,215,448-451^

### Recommendation 10.8.2

Patients who develop neurological deficit hours after waking normally from CEA should be examined with CDU at the bedside. If an occlusion is detected at the surgical site, the patient should undergo reoperation immediately to correct the cause of the thrombosis. If the carotid artery remains patent after the procedure, the patient should undergo an immediate CT angiography to determine the therapeutic approach (I/C).^5,9,215,448-451^

Routine patching for arteriotomy closure significantly reduces the risk of acute thrombosis as well as late restenosis.^262,452,453^ An alternative to patched conventional CEA is eversion CEA. The technique significantly reduces the risk of acute thrombosis and carotid artery restenosis.^263^

### Recommendation 10.9

For patients undergoing CEA, arteriotomy closure with patching is recommended, and primary closure is not recommended. Eversion endarterectomy is an alternative (I/A).^262,263,452,453^

### Neurological complications

#### Ischemic stroke

Perioperative ischemic strokes during and after CEA may be caused by cerebral ischemia during clamping, embolization of plaque fragments or thrombi during or immediately after surgery, and endarterectomy site thrombosis at the internal carotid artery.

Measures which reduce the risk of ischemic stroke are rigorous BP control during and after surgery, selective shunting, adequate heparinization, and routine use of arteriotomy patch closure or eversion CEA.^5,9,260,262,443,444,452-454^

#### Cerebral hyperperfusion syndrome

Cerebral hyperperfusion syndrome (CHS) is a rare but severe complication of invasive treatment of carotid artery disease by CEA or CAS.^263^ The primary risk factor for CHS is persistent high BP during or in the immediate postoperative period. Adjuvant factors include chronic deficit of brain perfusion due to occlusion or critical stenosis of the contralateral carotid and recent cerebral infarction.^455-457^ The risk of CHS is much higher for patients undergoing CAS compared to CEA.^458^ Prospective studies show CHS may be minimized by monitoring and aggressive BP control during CEA and in the immediate postoperative period (24-48 hours).^459,460^

#### Intracerebral hemorrhage

ICH is the most severe complication of CEA, fatal in over 50% of cases.^236^ ICH is caused by uncontrolled SHT, by hemorrhagic transformation of a recent cerebral infarction or as a complication of CHS. ICH is much more frequent in patients undergoing CAS than for CEA.^461,462^

Management consists of strict BP control, assisted ventilation, and intracranial pressure monitoring. A consultation with the neurosurgery team to assess and measure intracranial pressure and discuss possible surgical treatment of cerebral hematoma and cranial decompression.

#### Complications of endovascular treatment of the carotid arteries

Most complications were described in the previous sections and are related to the choice of access, stent type, constitution, and anatomical features of the plaques.^308^ The Specialist Advisers of the UK's National Institute for Health and Care Excellence (NICE) listed the known adverse events for CAS treatment, with thrombosis, embolism, pseudoaneurysm or arterial rupture possibly related to the access site and to the carotid artery. In terms of systemic complications, they are associated with reactions to contrast media (allergies and induced renal failure) and radiation-induced neoplasia.^279^

Verbal memory decline can also be present after carotid angioplasty (1-month post-intervention, p = 0.039). While for patients under the age of 80, severe atherosclerotic carotid disease and low baseline episodic memory scores benefit from carotid intervention (specifically CEA), individuals 80 and older have memory decline post-intervention (1 month, p = 0.046; 6 months, p = 0.043), with carotid artery stenting being an independent predictor of verbal memory decline.^463^

#### Postoperative care after carotid angioplasty

Hemodynamic instability requiring vasopressor support occurs in up to 19% of CAS procedures. In a meta-analysis (27 studies, n = 4,204), 12% of CAS patients were treated for hypotension, 12% for bradycardia, and 13% for both. There was a significant association between persistent hypotension after CAS and history of ipsilateral CEA, calcification with carotid bulb involvement, more significant stenosis, and eccentric plaque.^464^

Preventing hemodynamic instability during CAS involves hydration, suspending antihypertensives (depending on specific protocols and in conjunction with the anesthesia care and the intensive care unit teams) and continuous, real-time echocardiography and blood pressure (BP) monitoring. Glycopyrrolate (a synthetic atropine derivative) was compared to atropine in a retrospective study (n = 115), and was found to be more effective in preventing postoperative bradycardia (30% vs. 72%, p = 0.002) and hypotension (2.5% vs. 36%, p = 0.001), with lower rates of rebound hypertension (2.5% vs. 16%, p = 0.047).^464^ Hypotension is associated with decreased vascular resistance with lower sympathetic tone, not hypovolemia. Therefore, the use of vasopressors (norepinephrine, dobutamine, phenylephrine) may be necessary to keep SBP > 90 mmHg.^465^

The use of invasive methods for blood pressure control and deep vein puncture to treat hypotension helps facilitate patient monitoring and management, but depend on the individual protocols at each service, which define access sites and the cases for which they are to be performed.^464,465^

### Recommendation 10.10.1

The suspension of the administration of beta-blockers in the days leading to CAS should follow the protocol defined by the anesthesia care and surgical team. Expert panel.^463^

### Recommendation 10.10.2

The suspension of antihypertensive treatment after CAS after CAS should follow the protocol defined by the intensive care and surgical team. Expert panel.^463^

### Recommendation 10.11

The use of invasive methods for blood pressure control and deep vein puncture during and after CAS to treat hypotension should follow the protocol defined by the surgical team. Expert panel.^463,464^

## LIST OF ABBREVIATIONS

AHA American Heart Association

ASA Acetylsalicylic acid

APT Antiplatelet therapy/antiplatelet therapy

ACAS Asymptomatic Carotid Artery Study

ICA Internal carotid artery

MCA Middle cerebral artery

DOACs Direct oral anticoagulants

ACRS Asymptomatic Carotid Stenosis and Risk of Stroke Study

CAS Carotid artery angioplasty with stenting

ACST-1 Asymptomatic Carotid Surgery Trial

ACTI Asymptomatic Carotid Surgery Trial

ACTRIS Endarterectomy combined with optimal medical therapy (OMT) vs OMT alone in patients with asymptomatic severe atherosclerotic carotid artery stenosis at higher-than-average risk of ipsilateral stroke

GA General anesthesia

cTIA Crescendo transient ischemic attack

TIA Transient ischemic attack

LRA Locoregional anesthesia

HR-CEA High risk for carotid endarterectomy

MRA Magnetic resonance angiography

CTA Computed tomography angiography

VA Vertebral artery

CVA Stroke

SIE Stroke in evolution

HS Hemorrhagic stroke

I-CVA Ischemic stroke

VKA Vitamin K antagonist (e.g., anticoagulant and warfarin)

CLB Cervical locoregional block

CREST Carotid Revascularization Endarterectomy vs Stenting Trial

CABG Coronary artery bypass graft

CAD Coronary artery disease

PAD Peripheral arterial disease

DM Diabetes mellitus

CEPD Cerebral embolic protection devices

COPD Chronic obstructive pulmonary disease

DAT Dual antithrombotic therapy (antiplatelet drugs + DOAC)

SAE Serious adverse event — stroke and death

CEA Carotid endarterectomy

ACS Asymptomatic carotid stenosis

ECST European Carotid Surgery trial

CDU Color Doppler ultrasound

PRT Prospective randomized trial

mRs Modified Rankin Scale

ESVS European Society for Vascular Surgery

AF Atrial fibrillation

SHT Systemic hypertension

LMWH Low molecular weight heparin

ICH Intracerebral hemorrhage

HMG-CoA 3-hydroxy-3-methylglutaryl coenzyme A

AMI Acute myocardial infarction

PPI Proton pump inhibitor

CHF Congestive heart failure

VBI Vertebrobasilar insufficiency

LDL-c LDL-cholesterol (*low-density lipoprotein cholesterol*)

NASCET North American Symptomatic Carotid Artery Trial

NICE National Institute for Health and Care Excellence

NIHSS National Institutes of Health Stroke Scale

NNT Number needed to treat

OCT Optical coherence tomography

WHO World Health Organization

BP Blood pressure

PCSK9 Proprotein convertase subtilisin/kexin type 9

PO Postoperative

ePTFE expanded polytetrafluoroethylene

RCT Randomized controlled trial

MRI Magnetic resonance imaging

dMRI Diffusion-weighted magnetic resonance imaging

RR Risk reduction

CHS Cerebral hyperperfusion syndrome

SVS Society for Vascular Surgery

CT Computed tomography

TCAR Transcarotid artery revascularization with stenting

OCM Optimal clinical management

PAD Peripheral arterial disease

PCT Perfusion computed tomography

ET Endovascular treatment

SAT Supra-aortic trunks

VACS Veterans Affairs Cooperative Study

VB Vertebrobasilar

EDV End-diastolic velocity

PSV Peak systolic velocity

VQI Vascular Quality Initiative (Society for Vascular Surgery)
