# Supplementary material for: Brazilian Angiology and Vascular Surgery Society Guidelines for the treatment of extracranial cerebrovascular disease
Source: J Vasc Bras. 2024 May 31;23:e20230094. doi: 10.1590/1677-5449.202300942 (PMC11296686; doi:10.1590/1677-5449.202300942)
Supplement: Translation

# Translation: Diretrizes da Sociedade Brasileira de Angiologia e Cirurgia Vascular para o tratamento da doença cerebrovascular extracraniana

**DOI:** 10.1590/1677-5449.202300941

**Authors:** Arno von Buettner Ristow, Bernardo Massière, Guilherme Vieira Meirelles, Ivan Benaduce Casella, Marcia Maria Morales, Ricardo Cesar Rocha Moreira, Ricardo Jayme Procópio, Tércio Ferreira Oliveira, Walter Jr. Boim de Araujo, Edwaldo Edner Joviliano, Júlio Cesar Peclat de Oliveira

**Affiliations:** 1 Pontifícia Universidade Católica do Rio de Janeiro – PUC-RIO, Disciplina de Cirurgia Vascular e Endovascular, Rio de Janeiro, RJ, Brasil.; 2 Sociedade Brasileira de Angiologia e de Cirurgia Vascular – SBACV-RJ, Rio de Janeiro, RJ, Brasil.; 3 Sociedade Brasileira de Angiologia e de Cirurgia Vascular – SBACV-SP, São Paulo, SP, Brasil.; 4 Universidade Estadual de Campinas – UNICAMP, Hospital das Clínicas, Disciplina de Cirurgia do Trauma, Campinas, SP, Brasil.; 5 Universidade de São Paulo – USP, Faculdade de Medicina, São Paulo, SP, Brasil.; 6 Associação Portuguesa de Beneficência de São José do Rio Preto, Serviço de Cirurgia Vascular, São José do Rio Preto, SP, Brasil.; 7 Sociedade Brasileira de Angiologia e de Cirurgia Vascular – SBACV-PR, Curitiba, PR, Brasil.; 8 Pontifícia Universidade Católica do Paraná – PUC-PR, Hospital Cajurú, Serviço de Cirurgia Vascular, Curitiba, PR, Brasil.; 9 Universidade Federal de Minas Gerais – UFMG, Hospital das Clínicas, Setor de Cirurgia Endovascular, Belo Horizonte, MG, Brasil.; 10 Universidade Federal de Minas Gerais – UFMG, Faculdade de Medicina, Belo Horizonte, MG, Brasil.; 11 Sociedade Brasileira de Angiologia e de Cirurgia Vascular – SBACV-MG, Belo Horizonte, MG, Brasil.; 12 Sociedade Brasileira de Angiologia e de Cirurgia Vascular – SBACV-SE, Aracajú, SE, Brasil.; 13 Universidade de São Paulo – USP, Faculdade de Medicina de Ribeirão Preto – FMRP, Ribeirão Preto, SP, Brasil.; 14 Universidade Federal do Paraná – UFPR, Hospital das Clínicas – HC, Curitiba, PR, Brasil.; 15 Universidade Federal do Estado do Rio de Janeiro – UNIRIO, Departamento de Cirurgia, Rio de Janeiro, RJ, Brasil.

**Keywords:** estenose carotídea, acidente vascular encefálico, diretrizes, endarterectomia de carótidas, doenças das artérias carótidas, lesões de artérias carótidas

## Abstract

A doença cerebrovascular extracraniana tem sido intensamente investigada em todo o mundo, sendo tema de suma importância para os cirurgiões vasculares. A presente Diretriz foi elaborada pela Sociedade Brasileira de Angiologia e Cirurgia Vascular (SBACV) em sucessão à Diretriz de 2015. As doenças de etiologia não ateroscleróticas não foram incluídas nesse documento. O objetivo desta Diretriz é congregar as evidências mais robustas nessa área para auxiliar os especialistas no processo decisório do tratamento. Foi utilizada a metodologia AGREE II e o sistema da Sociedade Europeia de Cardiologia para as recomendações e níveis de evidências. As recomendações foram graduadas de I a III, e os níveis de evidência classificados em A, B e C. A presente Diretriz foi dividida em 11 capítulos, que tratam dos vários aspectos da doença cerebrovascular extracraniana: diagnóstico, tratamentos e complicações, de forma atualizada e com as recomendações propostas pela SBACV.

## INTRODUÇÃO E METODOLOGIA

A presente Diretriz para tratamento de pacientes portadores da doença cerebrovascular extracraniana foi elaborada pela Sociedade Brasileira de Angiologia e Cirurgia Vascular (SBACV) em sucessão à Diretriz de 2015. As doenças de etiologia não ateroscleróticas, com exceção das dissecções (arterites, displasia fibromuscular, dissecções e aneurismas) não foram incluídas nesse documento. A presente Diretriz tem o objetivo de congregar as evidências mais robustas nessa área para auxiliar no processo decisório do tratamento. Para sua elaboração, foi adotada a metodologia AGREE II para produção de diretrizes e avaliação^1,2^ . O comitê de trabalho para a produção da Diretriz foi composto por membros titulares da SBACV com notório saber no tratamento das doenças carotídeas.

O comitê de trabalho realizou reuniões remotas para revisão do progresso do documento. As estratégias de pesquisa para seleção dos trabalhos envolveram a utilização da plataforma MEDLINE até janeiro de 2023. Apenas publicações revisadas por pares foram incluídas, seguindo o princípio da pirâmide de evidência. Estudos randomizados e metanálises de estudos randomizados foram colocados no topo, seguidos por estudos randomizados individuais ou estudos grandes não randomizados, metanálises de pequenos estudos não randomizados, estudos observacionais, série de casos e estudos retrospectivos. Opiniões de especialista encontram-se na base da pirâmide, enquanto relatos de casos foram excluídos.

O sistema da Sociedade Europeia de Cardiologia foi utilizado no desenvolvimento das recomendações e níveis de evidências. As classes foram graduadas de I a III, sendo I a mais forte^3^ . Os níveis de evidência foram classificados em A, B e C, sendo A o maior. Foram designadas áreas específicas para cada membro do comitê de trabalho desenvolver as recomendações, sendo essas subsequentemente revisadas em colegiado e classificadas por nível de evidência.

Os Quadros 1 e 2 abaixo sumarizam as classes e os níveis de evidências empregadas nestas Diretrizes. O objetivo é orientar o especialista em relação a assuntos relacionados ao diagnóstico e tratamento da doença cerebrovascular extracraniana sobre os quais existam graus de recomendação e níveis de evidência incontestáveis à luz dos conhecimentos científicos na atualidade. Há várias áreas em que esses objetivos ainda não foram atingidos, e talvez nunca o sejam. Esses temas também serão abordados sempre que relevantes, e a situação atual ressaltada neste texto.

**Quadro 1:** Classes de recomendação.

**Classe**	**Indicações e definição**	**Recomendação**
I	Consenso de que o procedimento/tratamento é eficaz e útil	É recomendável
II	Condições para as quais não há consenso acerca da eficácia utilidade do procedimento/tratamento	
IIa	A opinião favorece a indicação do procedimento/tratamento	Deve ser considerado
IIb	A eficácia/utilidade do procedimento/tratamento é menos comprovada pela experiência/opinião	Pode eventualmente ser
III	Consenso de que o procedimento/tratamento não é eficaz ou útil e que em certos casos, pode gerar risco	Não é recomendado e não deve ser realizado

**Quadro 2:** Níveis de evidência.

**Nível**	**Definição**
A	Dados obtidos a partir de múltiplos estudos randomizados de boa qualidade
B	Dados obtidos de um único estudo randomizado de boa qualidade ou de grandes estudos não randomizados
C	Dados obtidos de estudos de séries de casos e/ou dados de registros, consensos e/ou opinião de especialistas

## PROPEDÊUTICA CLÍNICA

O acidente vascular cerebral (AVC) é uma síndrome neurológica de início súbito decorrente da interrupção do fluxo sanguíneo em região específica do sistema nervoso central, manifestando-se por disfunção neurológica focal súbita com duração superior a 24 horas. O AVC pode ocorrer por diversos mecanismos isolados ou em conjunto. A classificação Trial of ORG 10172 in Acute Stroke Treatment (TOAST) definiu cinco tipos etiológicos de AVC: aterosclerose em grandes artérias, oclusão de pequenas artérias (lacunar), cardioembólico, indeterminado e outras causas (pouco frequentes)^4^ .

O ataque isquêmico transitório (AIT) é definido como ocorrência de disfunção focal encefálica, retiniana ou medular com menos de 24 horas de duração, de etiologia não traumática^5^ . Na aterosclerose de grandes artérias, a isquemia pode ser decorrente da embolização de placas ateroscleróticas ou da oclusão do vaso com efeito hemodinâmico. Os locais mais comuns de aterosclerose são a bifurcação carotídea, a aorta e as artérias vertebrais.

Na etiologia cardioembólica, as fontes podem ser de alto risco, como a presença de trombo intracardíaco, fibrilação atrial (FA) e doença valvar reumática, e de baixo risco, como forame oval patente, acinesia apical e segmento hipocinético do ventrículo esquerdo. Em nosso meio, deve-se pensar em doença de Chagas como causa de AVC cardioembólico, caso haja história epidemiológica sugestiva. O infarto lacunar resulta da oclusão de pequenas artérias originadas das artérias cerebrais médias, vertebrais, basilares ou vasos ramos do polígono de Willis.

O exame neurológico deve ser objetivo, tendo em vista a necessidade da rápida definição da conduta. Duas escalas têm sido empregadas nas Diretrizes mais recentemente publicadas: a National Institute of Health Stroke Scale, mais completa e complexa, que é empregada para quantificar o déficit neurológico, avaliar o prognóstico, determinar a localização topográfica e facilitar a comunicação entre os profissionais de saúde (Quadro 3) e a escala de Rankin modificada (eRm), como apresentado no Quadro 4, mais sintética e expedita^6-8^ .

**Quadro 3:** National Institutes of Health Stroke Scale – NIHSS^5^ .

	Definição na escala
**1a – Nível de consciência**	0 = Alerta; reponde com entusiasmo.
O investigador deve escolher uma resposta mesmo se uma avaliação completa eì prejudicada por obstáculos como um tubo orotraqueal, barreiras de linguagem, trauma ou curativo orotraqueal. Um 3 eì dado apenas se o paciente não faz nenhum movimento (outro além de postura reflexa) em resposta aÌ estimulação dolorosa.	1 = NaÞo alerta, mas ao ser acordado por miìnima estimulaçaÞo obedece, responde ou reage.
	2 = NaÞo alerta, requer repetida estimulaçaÞo ou estimulaçaÞo dolorosa para realizar movimentos (naÞo estereotipados).
	3 = Responde somente com reflexo motor ou reaçoÞes autonômicas, ou totalmente irresponsivo, flaìcido e arreflexo.
**1b. Perguntas de nível de consciência**	0 = Responde ambas as questoÞes corretamente.
O paciente eì questionado sobre o mês e sua idade.	1 = Responde a uma questaÞo corretamente.
	2 = NaÞo responde a nenhuma questaÞo corretamente.
**1c. Comandos de nível de consciência**	0 = Realiza ambas as tarefas corretamente.
O paciente eì solicitado a abrir e fechar os olhos e então abrir e fechar a mão não parética.	1 = Realiza uma tarefa corretamente.
	2 = NaÞo realiza nenhuma tarefa corretamente.
**2. Motricidade ocular**	0 = Normal.
Somente os movimentos oculares horizontais são testados.	1 = Paresia do olhar conjugado.
	2 = Desvio do olhar conjugado.
**3. Campos visuais**	0 = Normal.
	1 = Hemianopsia parcial.
	2 = Hemianopsia completa.
	3 = Cegueira cortical
**4. Paralisia facial**	0 = Normal.
	1 = Paresia mínima.
	2 = Paresia/segmento inferior da face.
	3 = Paresia/segmento superior e inferior da face.
**5. Motor: membro superior**	0 = Sem queda.
	1 = Queda, mas não atinge o leito.
	2 = Força contra a gravidade, mas não sustenta.
	3 = Sem força contra a gravidade, mas qualquer movimento mínimo é considerado.
	4 = Sem movimento.
**6. Motor: membro inferior**	0 = Sem queda.
	1 = Queda, mas não atinge o leito.
	2 = Força contra a gravidade, mas não sustenta.
	3 = Sem força contra a gravidade, mas qualquer movimento mínimo é considerado.
	4 = Sem movimento
**7. Ataxia apendicular**	0 = Sem ataxia.
	1 = Ataxia em membro superior ou inferior.
	2 = Ataxia em membro superior ou inferior.
**8. Sensibilidade dolorosa**	0 = Normal.
	1 = Déficit unilateral, mas reconhece estímulo.
	2 = Paciente não reconhece estímulo; coma ou déficit bilateral.
**9. Linguagem**	0 = Normal.
	1 = Afasia leve a moderada (compreensível).
	2 = Afasia grave.
	3 = Mudo, afasia global e coma.
**10. Disartria**	0 = Normal.
	1 = Afasia leve à moderada.
	2 = Grave, inteligível ou mudo.
	X = Entubado.
**11. Extinção/negligência**	0 = Normal.
	1 = Negligência ou extinção em uma modalidade sensorial.
	2 = Negligência em mais de uma modalidade sensorial.

**Quadro 4:** Escala de Rankin modificada^6,7^ .

**Descrição dos sintomas**	**Definição na escala**
Assintomático.	0
Sem déficit significativo e apto a desempenhar quase todas as ações.	1
Leve incapacidade. Incapaz de desempenhar algumas atividades que conseguia previamente, mas capaz de suprir suas necessidades básicas sem auxílio.	2
Incapacidade moderada. Necessita de ajuda para suprir suas necessidades básicas, mas consegue caminhar sem assistência.	3
Incapacidade moderada a grave. Incapacidade de andar sem ajuda, incapacidade de realizar suas necessidades sem ajuda.	4
Incapacidade grave. Acamado, incontinente e demandando cuidados constantes de atenção e de enfermagem.	5
Óbito.	6

## MÉTODOS DIAGNÓSTICOS POR IMAGEM

A investigação por imagem dos pacientes com estenose carotídea está baseada em três parâmetros principais – identificar a localização anatômica da obstrução, definir o grau de redução luminal e as características da placa. As estenoses maiores e as placas heterogêneas, volumosas e hipoecogênicas se relacionam a maior risco de AVC^9^ .

O eco-Doppler colorido (eDc) é o exame de escolha inicial para a investigação dos pacientes com suspeita de estenose carotídea^10^ . Trata-se de método amplamente disponível, de baixo custo, que pode ser realizado à beira do leito, sem necessidade de anestesia, sem radiação ionizante ou de contraste venoso. Além disso, sua acurácia e confiabilidade são elevadas quando realizado por examinadores experientes e dentro dos critérios já estabelecidos na literatura: sensibilidade de 85 a 92% e especificidade de 84%^9,11^ .

As carótidas devem ser inicialmente estudadas pela varredura em modo B. Ao se detectar uma placa, a porcentagem de redução luminal e o lúmen residual devem ser medidos no corte transversal^12^ . A seguir, deve-se identificar as placas vulneráveis ou instáveis, isto é, que apresentam maior risco de eventos cerebrovasculares. Para isso, analisam-se as características da placa^13^ . Recomenda-se que o local, extensão, ecogenicidade, textura, superfície, presença de componentes móveis e de zona anecoica junto à cápsula fibrótica sejam pesquisados e descritos ao se encontrar placas significativas, isto é, que promovam estenose maior que 50%^12^ .

A ecogenicidade da placa é dividida em três tipos. A mais escura, de aspecto similar ao do sangue, é denominada hipoecogênica ou ecolucente e composta por lipídios, sangue ou trombos recentes. A isoecogênica tem aparência próxima à musculatura, tem conteúdo fibroso. A hiperecogênica é mais branca que o músculo adjacente e é constituída por tecido fibroso mais denso. Quando algumas dessas placas se tornam calcificadas, é formada a sombra acústica, gerando uma faixa escura na região posterior à placa, formada pelo impedimento de propagação do ultrassom através do cálcio. As placas ainda são ditas homogêneas quando apresentam nível de ecos uniformes, ou heterogêneas, se compostas por ecos de diferentes níveis^14^ . As placas podem ser agrupadas em cinco tipos, de acordo com sua ecogenicidade e consequente associação com eventos^15^ :

Tipo 1 - ecolucente e homogênea, com ou sem cápsula fibrosa definida – considerada de risco moderado para eventos.
Tipo 2 - predominantemente ecolucente, com áreas ecogênicas inferiores a 50% do total da placa – placa de maior instabilidade, portanto, de maior risco.
Tipo 3 - predominantemente ecogênicas, com área hiperecogênica superior a 50% da área total da placa – risco entre o tipo 1 e 2.
Tipo 4 - uniformemente ecogênica.
Tipo 5 - calcificada, com sombra acústica.

As duas últimas são consideradas estáveis e, portanto, de menor risco.

Em relação à superfície, se as irregularidades forem inferiores a 0,4 mm de profundidade, a placa é classificada como regular. É irregular quando a profundidade se situa entre 0,4 e 2,0 mm. As placas ulceradas apresentam depressão maior que 2,0 mm. Porém, isso não é suficiente: deve-se observar ecos menos intensos no interior da escavação quando comparados aos ecos obtidos na superfície dos segmentos adjacentes da mesma placa. Esse critério apresenta uma sensibilidade de 85,7%, e uma especificidade de 81,3%^16^ .

Para quantificação do grau de estenose, porém, é imprescindível a avaliação das velocidades do fluxo nas artérias carótidas. É muito importante que as mensurações das velocidades sejam feitas com critérios técnicos rigorosos: volume de amostra pequeno, posicionado no centro do vaso, em paralelo à direção do fluxo e com ângulo, entre 45 e 60º – idealmente ≤ 60º^12^ . Os critérios de velocidades comumente utilizados na prática clínica se encontram no Quadro 5^4,9^ .

**Quadro 5:** Critérios de velocidades para quantificação do grau de estenose carotídea^12,17^ .

**% NASCET** **a**	**VPS** **b** **carótida interna**	**VDF** **c** **carótida interna**	**Razão VPS interna/VPS comum**	**St. Mary’s VPS interna/VDF comum**
< 50%	< 125 ou < 140	< 40	< 2	< 8
50-59%	> 125 ou > 140	40 a 69	2-3,1	8-10
60-69%		70 a 100	3,2-4	11-13
70-79%	> 230	> 100	> 4	14-21
80-89%		> 140	Entre 4 e 5	22-19
> 90	> 400		> 5	> 30
Suboclusão	Variável		Variável	Variável
Oclusão	Sem fluxo		N.A.	N.A.

a porcentagem de estenose pelos correspondentes ao critério NASCET;

b velocidade de pico sistólico;

c velocidade diastólica final. Todas as velocidades em cm/seg.

A velocidade de pico sistólico (VPS) possui a maior acurácia na graduação das estenoses, sendo o critério principal a ser observado. Os demais parâmetros são úteis para confirmar e estratificar o estreitamento estimado pela VPS.

Em relação às estenoses maiores que 50%, há uma tendência de se adotar valor de corte de VPS mais alto (em torno de 140 cm/seg e não de 125 cm/seg), de forma a melhorar a acurácia do método^18,19^ . Além disso, Morales et al.^20^ demonstraram que a avaliação da estenose através da VPS nos pacientes com placas associadas à sombra acústica importante mostrou baixa sensibilidade e discordância em relação à angiotomografia computadorizada (ATC).

A angiografia com subtração digital é o exame de maior sensibilidade, especificidade e acurácia na detecção da estenose carotídea acima de 95% de estenose^21^ . No entanto, seu caráter invasivo e o risco associado de acidente vascular cerebral isquêmico (AVCI), confirmado no Asymptomatic Carotid Artery Study (ACAS), aliado ao desenvolvimento dos métodos não invasivos, restringiu a sua indicação^21,22^ .

Atualmente, a angiografia é reservada na investigação dos pacientes com suspeita de estenose carotídea que não foi esclarecida por pelo menos dois métodos de imagem não invasivos. Isso pode ser visto quando os métodos apresentam resultados divergentes nos pacientes com contraindicação a determinado método – como claustrofobia para a ressonância magnética (RM) – ou apresentam limitação técnica importante, como, por exemplo, excesso de calcificação que impeça a propagação das ondas sonoras ou lesões além do ângulo da mandíbula. Além disso, deve ser oferecida apenas aos pacientes com provável benefício de revascularização, se detectada estenose significativa.

### Recomendação 1

A angiografia com subtração digital é reservada como exame pré-operatório para pacientes que apresentaram resultados discordantes em métodos de imagem não invasivos (I/B)^5,12^ .

A ATC é considerada uma das modalidades de imagem mais requisitada pelos cirurgiões vasculares em nosso meio. Trata-se de método muito disponível, de rápida aquisição e que permite visibilizações em vários planos e projeções e reconstruções multiplanares e em três dimensões. Permite estudar a circulação extra e intracraniana, incluindo o arco aórtico e os troncos supra-aórticos (TSAs). A mensuração da redução luminal é feita em analogia ao método angiográfico e de forma precisa. A sua sensibilidade para estenoses graves – entre 70-99% de redução luminal – e oclusão é de 85% e 97%, e a especificidade, de 93% e 99%, respectivamente. Não permite avaliar com grande precisão a morfologia e características da placa e tem pouca resolução em placas muito calcificadas^20^ . Além disso, requer contraste iodado e radiação ionizante^23^ . Dessa forma, é usualmente realizada para confirmar os achados do eDc. Além disso, permite definir o tipo de arco aórtico, o trajeto e a tortuosidade da artéria a ser tratada, fatores importantes nos candidatos à angioplastia de carótida. Também examina segmentos arteriais inacessíveis ao ultrassom, como a circulação intracraniana.

A angiorressonância magnética (ARM) é um método de imagem não invasivo, que não requer radiação ionizante ou contraste iodado. Permite o estudo dos vasos intratorácicos, cervicais e intracranianos em diversas projeções e reconstruções vasculares que podem ser obtidas de seus dados. Pode também detectar lesões do parênquima cerebral. A sensibilidade e especificidade para estenoses maiores que 70% é a mesma do eDc^24^ . Tende, porém, a superestimar as lesões moderadas e as suboclusões. Por outro lado, é capaz de avaliar as características da placa: tanto do seu conteúdo quanto a superfície. Tem como desvantagem a longa duração para a obtenção de estudos envolvendo o arco aórtico, a circulação cervical e intracraniana (que na ATC demanda segundos), e não pode ser realizada em pacientes com dispositivos implantáveis, tais como geradores de marcapasso e desfibriladores. Alguns, mas não todos os tipos de gadolínio (contraste paramagnético utilizado na ARM), se associaram à fibrose sistêmica nefrogênica nos pacientes com *clearance* de creatinina menor que 30 mL/min^23^ . As indicações para a ARM são semelhantes à da ATC – confirmar os achados do eDc e estudar o arco aórtico, os TSAs e a circulação intracraniana.

Não há consenso em como avançar na investigação dos pacientes assintomáticos com estenose maior que 50% na carótida interna, detectados em estudo de eDc^5^ . De forma geral, quando os pacientes têm proposta de intervenção, as lesões detectadas devem ser confirmadas por outro método de imagem. Os métodos mais utilizados são a ATC ou a ARM^5^ . Como permitem o estudo do arco aórtico, dos TSAs e dos ramos das artérias intracranianas e do parênquima cerebral, auxiliam na seleção dos indivíduos que, mesmo assintomáticos, tenham maior risco de AVCI ou de AIT, como será abordado em outras partes destas Diretrizes.

Ademais, permitem avaliar critérios anatômicos, como a altura da bifurcação, calcificação e tortuosidade do arco aórtico e das carótidas internas. Todos esses fatores anatômicos, aliados à história clínica do paciente, são essenciais na escolha da melhor técnica de intervenção – cirurgia convencional, angioplastia de carótida transfemoral ou transcarotídea. Como alternativa, os pacientes candidatos à endarterectomia de carótida (EC) podem ser submetidos a um segundo eDc, com outro examinador, para que se confirme a presença e grau da estenose. No caso de exames discordantes em pacientes com indicação de intervenção, um terceiro exame pode ser realizado. Talvez essa seja uma das poucas indicações de angiografia com subtração digital atualmente^5^ .

Os exames de imagem apresentam a mesma acurácia para indivíduos assintomáticos e pacientes sintomáticos. No entanto, o tratamento invasivo do AVC agudo vem se tornando cada vez mais frequente^25^ . Os pacientes com déficit neurológico agudo secundário a AVCI se beneficiam da trombólise venosa quando realizada em até 4,5 horas do primeiro sintoma^8^ . Já aqueles com AVCI entre 4,5 e 24 horas podem ser candidatos à trombectomia mecânica. Para isso, é necessário a avaliação da extensão da isquemia além da presença de oclusão da carótida interna, da artéria cerebral média (ACM) ou ramos ou da artéria cerebral anterior. Condição essencial de ambas as intervenções é a exclusão de hemorragia^26^ . Dessa forma, pacientes com suspeita de AVC devem ser submetidos de forma imediata a exame de imagem do encéfalo^27^ .

A tomografia computadorizada (TC) de encéfalo sem contraste é o método de imagem mais disponível em nosso meio. Apresenta baixa sensibilidade para detectar lesão isquêmica precoce – por volta de 64% nas primeiras 6 horas. Exclui, porém, com segurança, a presença de hemorragia. Assim, é suficiente para se indicar a trombólise^28^ . Hoje em dia, a maioria dos pacientes com isquemia cerebral aguda é submetida à TC de encéfalo e à ATC. Esta, além de permitir o estudo da circulação cerebral intra e extracraniana, permite a realização da TC de perfusão (TCP). A TCP avalia a perfusão do parênquima cerebral através de *software* específico. Assim, permite delimitar a extensão do infarto cerebral e a presença e extensão da área de penumbra – tecido cerebral potencialmente viável se novamente irrigado. A ATC e a TCP permitem a seleção dos pacientes com indicação de trombectomia mecânica^26^ .

A RM de encéfalo apresenta alta acurácia para a detecção do AVC precoce, hemorragia recente e antiga. Entretanto, é pouco disponível, de maior complexidade técnica de execução e análise e requer mais tempo de realização. A RM pode ser associada à ARM e à RM de difusão e perfusão, estas duas sequências específicas de obtenção de imagem^27^ . A ARM estuda a circulação cerebral intra e extracraniana, confirmando, por exemplo, a presença de oclusão de grandes artérias, critério imprescindível para a trombectomia mecânica. A RM de difusão demonstra a área de infarto cerebral. A RM de perfusão mostra a área de penumbra. A trombectomia mecânica está indicada quando há um *mismatch* (discordância) entre a área de infarto e a área de penumbra na imagem, ou entre o quadro clínico e a área de infarto – déficit neurológico maior que o previsto para o tamanho do infarto cerebral^28^ .

Uma pergunta surge com frequência no manejo dos pacientes com doença cerebrovascular de origem extracraniana: quais métodos de imagem são necessários para se indicar uma EC, e a mesma indagação é comum em relação à angioplastia com implante de *stent* (ACS). Como regra geral, deve haver dois exames concordantes, ou seja, que demonstrem obstruções similares: um exame de eDc das artérias carótidas e vertebrais, associado a uma ATC, ou eventualmente uma ARM. Na impossibilidade de realização de um exame contrastado, pode-se solicitar um segundo eDc realizado por outro examinador. Pacientes com sintomas devem ter estudo do parênquima cerebral realizado.

## TERAPIA CLÍNICA OTIMIZADA

### Introdução

Todos os portadores de doença carotídea, com ou sem indicação de intervenção cirúrgica, são beneficiados pela otimização do seu tratamento clínico. A terapia clínica otimizada consiste em três elementos principais: 1 - adoção de hábitos saudáveis alimentares e de atividade física; 2 - controle das comorbidades e fatores de risco; e 3 - otimização da terapia antitrombótica e hipolipemiante. Tais medidas são necessárias em todas as fases da abordagem da doença carotídea, desde o seu imediato diagnóstico até o período pós-operatório tardio, uma vez que o paciente será sempre um portador de doença aterosclerótica.

### Mudança de hábitos e controle de fatores de risco

É notória a necessidade, bem como a contribuição positiva, da adoção de hábitos saudáveis de alimentação e atividade física no controle das doenças cardiovasculares em geral. A obesidade é fator de risco para o desenvolvimento de doença aterosclerótica, podendo contribuir para o desenvolvimento precoce da doença carotídea^29^ . Para os portadores de doença aterosclerótica em geral, a adoção de dietas caloricamente balanceadas, ricas em vegetais e grãos integrais, evitando alimentos ultraprocessados, com sódio em excesso, embutidos e carboidratos de absorção rápida, resulta em melhora dos indicadores laboratoriais e de qualidade de vida e na redução de eventos mórbidos cardiovasculares^30,31^ . A frequência com que indivíduos realizam atividades físicas é diretamente proporcional à redução do risco de doença cardiovascular em geral, e doença carotídea em especial^32^ .

O tabagismo está associado tanto à doença carotídea em si como ao risco aumentado de AVCI, com aumento do risco relativo em quase 10 vezes para este último. A redução ou abandono do hábito tabágico é primordial na prevenção da evolução da estenose carotídea e de seus eventos embólicos^33^ . Cerca de 77% dos pacientes que apresentam um primeiro episódio de AVC são hipertensos^34^ . O controle da pressão arterial (PA) apresenta correlação diretamente proporcional à redução da incidência e do risco pessoal de AVCs^35^ . O controle da hipertensão arterial deve ser visto como elemento essencial na abordagem clínica da doença carotídea e segue diretrizes específicas^35-37^ .

### Recomendação 2.1

Em pacientes com estenose da artéria carótida interna (ACI), recomendam-se ações proativas de orientação de hábitos alimentares saudáveis, atividade física compatível com as limitações do paciente e, quando aplicáveis, perda ponderal, cessação do tabagismo e controle da hipertensão arterial (I/B)^30,35^ .

### Antiagregação na doença carotídea assintomática

Os benefícios da terapia antitrombótica, em particular a terapia antiagregante, na redução de eventos cardiovasculares em pacientes com doença cardiovascular aterosclerótica estabelecida (coronariopatia, doença cerebrovascular, doença arterial periférica [DAP]) foram estabelecidos há mais de 1/4 de século^38^ . No entanto, o volume de evidências científicas específicas à terapia antitrombótica, especificamente em portadores de doença carotídea assintomática, ainda é limitado. Um ensaio clínico randomizado comparando o uso de ácido acetilsalicílico (AAS) e placebo falhou em identificar benefícios daquela terapia^39^ . Já um estudo observacional de pacientes com estenose assintomática maior que 70% identificou expressivo benefício da terapia de antiagregação plaquetária^40^ . Adicionalmente, por se tratar de uma população na qual a presença de doença aterosclerótica em outros sítios (coronariopatia, DAP) é frequente, a antiagregação plaquetária deve ser considerada para pacientes com tolerância à medicação^41^ .

### Recomendação 2.2

Em pacientes com estenose da ACI assintomática, deve-se considerar antiagregação com AAS em baixas doses (81 a 325 mg/d) sobre outras estratégias antitrombóticas ou não uso de antiagregantes plaquetários (AAP) (IIa/C)^39,41^ .

### Dupla terapia antitrombótica na doença carotídea assintomática

A estratégia de dupla terapia antitrombótica (DTAT), com uso de AAP e anticoagulante direto, composta por AAS 100 mg ao dia associada à rivaroxabana 2,5 mg duas vezes ao dia, para portadores de doença cardiovascular estável foi investigada no ensaio clínico COMPASS^42^ . O subestudo COMPASS-PAD^43^ é uma análise de 7.470 pacientes do estudo geral, portadores de DAP e/ou doença carotídea^43^ . Nesse grupo, a DTAT resultou em redução de 28% (IC95% 0,57-0,90; p < 0.004) dos eventos de desfecho principal de morte cardiovascular, infarto agudo do miocárdio (IAM), AVC (EAG), com redução específica de 46% nos AVCIs de todas as causas (IC95% 0,33-0,87). Por outro lado, houve aumento de 1,61 vezes na incidência de sangramentos maiores nos usuários de DTAT (IC95% 1,12-2,31; p < 0.008), o que demanda uma análise parcimoniosa do risco de sangramento na indicação da DTAT^43,44^ .

A análise exclusiva dos resultados dos 1.919 pacientes que apresentavam estenose carotídea maior que 50% falhou em apresentar benefícios específicos de redução de EAG para este grupo, embora a incidência de sangramentos maiores tenha sido similar para os grupos com ou sem adição de rivaroxabana. Uma subanálise do estudo COMPASS não conseguiu identificar redução específica na incidência de AVCI associados à doença carotídea com a DTAT, embora a incidência geral de AVCs tenha reduzido com o seu uso^44^ . Assim, ainda que não tenha sido comprovado o benefício específico da DTAT para pacientes com doença carotídea isolada, seu uso pode ser considerado na presença de outras comorbidades cardiovasculares associadas à doença carotídea em pacientes com baixo risco de sangramento.

### Recomendação 2.3

Em pacientes com estenose da ACI assintomática com outras comorbidades cardiovasculares (doença coronariana, DAP) e baixo risco de sangramento, pode ser considerada a estratégia de dupla terapia antitrombótica, com rivaroxabana 2,5 mg a cada 12 horas mais AAS 100 mg ao dia (IIb/B)^42,44^ .

### Terapia hipolipemiante na doença carotídea assintomática

A terapia hipolipemiante com estatinas em portadores de doença cardiovascular deve ser considerada mesmo quando os níveis séricos de LDL-colesterol (LDL-c) estão dentro do alvo^45^ . As estatinas foram associadas à redução de AVCI em pacientes com risco aumentado de eventos cardiovasculares e doença carotídea, mesmo em pacientes sem dislipidemia severa^46,47^ . Há evidências suficientes que comprovam os benefícios de redução de eventos em portadores de doenças cardiovasculares tratados cronicamente com terapia hipolipemiante com estatinas associadas ou não a inibidores da absorção intestinal do colesterol (ezetimiba, principalmente)^48,49^ . Apesar da ausência de estudos clínicos prospectivos investigando o impacto da terapia hipolipemiante exclusivamente em portadores de doenças carotídeas, dados de estudos menos específicos são suficientes para a recomendação do seu uso.

### Recomendação 2.4

Em pacientes com estenose da ACI assintomática, recomenda-se terapia hipolipemiante com estatina associada ou não a ezetimiba na prevenção de eventos cardiovasculares (IAM, AVC, morte cardiovascular) (I/B)^46,48^ .

Pacientes com dislipidemia severa e doença cardiovascular devem ter metas de redução do LDL-c claras, pois a persistência de valores altos mantém o indivíduo em risco aumentado para eventos cardiovasculares graves. Na ausência de redução efetiva do LDL-c, mesmo diante da terapia otimizada com estatinas e ezetimiba ou quando ocorrem efeitos colaterais importantes com seu uso, outras estratégias devem ser consideradas. Estudos prospectivos randomizados controlados foram realizados com dois anticorpos monoclonais inibidores da PCSK9 (do inglês Proprotein Convertase Subtilisin/Kexin type 9), o evolocumab e o alirocumab. Ambos demonstraram redução das taxas de desfechos combinados de morte cardiovascular, AVCI, IAM, revascularização miocárdica ou hospitalização por angina. O evolocumab reduziu os desfechos combinados em 15% (IC95% 0,79-0,92; p < 0.001); enquanto o alirocumab apresentou uma redução de 3,3% para 1,7% (IC95% 0,31-0,90; p = 0,02), com razão de risco de 0,52^49,50^ . A redução específica de AVCI foi de 25% nos usuários de evolocumab (IC95% 0,62-0,92; p < 0.005)^51^ .

Assim, em pacientes com doença carotídea e dislipidemia não responsiva à terapia hipolipemiante clássica com estatinas/ezetimiba deve ser considerado o uso em associação de drogas inibidoras da PCSK-9^49-52^ .

### Recomendação 2.5

Em pacientes com estenose da ACI assintomática e dislipidemia não responsiva a terapia convencional com estatinas isoladas ou associadas a ezetimiba, o uso de inibidores da PCSK9 deve ser considerado (IIa/C)^49-52^ .

- Nota sobre o uso de inibidores de bomba de prótons (IBP) e suas possíveis interações com AAPs: o uso de AAPs, sob a forma de mono ou dupla terapia, eleva o risco de sangramento gastrointestinal, particularmente em pacientes mais suscetíveis^53,54^ . No entanto, há evidências, por vezes conflitantes, de que os IBPs usados como protetores gástricos podem apresentar ações inibitórias sobre AAPs. Dois estudos retrospectivos observaram que o uso de IBPs foi associado a maior incidência de eventos cardiovasculares em pacientes com história de síndrome coronariana aguda recente, o que supostamente esteve associado à redução da efetividade plaquetária do clopidogrel e AAS^55,56^ . Em investigação prospectiva de indivíduos saudáveis não se observou redução da ação do clopidogrel sobre a agregação plaquetária quando utilizado em concomitância com as drogas pantoprazol, omeprazol, rabeprazol, esomeprazol, lansoprazol ou dexlansoprazol^57^ .

A presença de polimorfismo do citocromo CYP2C19*2 e CYP2C19*3 está associada à redução da metabolização dos IBPs e também à eficácia direta do clopidogrel^58^ . No entanto, em pacientes submetidos à dupla terapia antiagregante, o uso de pantoprazol não interferiu no efeito antiplaquetário, mesmo na presença de polimorfismo do citocromo CYP2C19^59^ .

Assim, para pacientes sob antiagregação plaquetária que necessitem o uso de fármacos protetores gástricos, deve-se considerar adequadamente tanto a escolha do AAP quanto do protetor gástrico em questão, com preferência pelo pantoprazol, ou, quando esse não é tolerável, por antagonistas do receptor H_2_ (ranitidina, famotidina).

## DOENÇA CAROTÍDEA ASSINTOMÁTICA

Os AVCs são a segunda causa de mortalidade e a terceira de invalidez, mundialmente. A incidência é elevada – 12,2 milhões AVCs/ano, ocorrendo um AVC a cada 3 segundos. Esses números cresceram 50% nos últimos 17 anos, especialmente nos países de baixa renda, com tendência desastrosa de afetar pacientes mais jovens (63% abaixo de 70 anos). Os AVCIs correspondem a 80-85% de todos os eventos, e cerca de 25% deles estão relacionados à doença das carótidas cervicais, o que torna evidente a pressão exercida sobre os sistemas de saúde bem como a relevância de se desenvolver estratégias para reduzir o impacto social – a maior parte desses AVCIs poderia ser evitada com mudança de hábito e controle de doenças metabólicas^60^ .

Apesar do grande número de trabalhos, sobretudo de diretrizes, publicados recentemente, algumas questões permanecem com respostas discordantes, especialmente no que se refere ao melhor manejo para uma estenose carotídea assintomática. A adoção de um grupo de medidas clínicas rigorosas (controle de hipertensão arterial [HAS] e de dislipidemia, abandonar o fumo, adotar dietas adequadas e atividade física) é mandatória e modificou o tratamento da doença carotídea assintomática, levando a uma redução das taxas de AVCI; no entanto, há evidências de que essas medidas não são suficientes para evitar a ocorrência de AVCI em todos os indivíduos. Está claro que nem todos apresentam o mesmo risco de AVCI ao longo do tempo, e que uma EC profilática pode ser benéfica. Da mesma forma, muito se discute a respeito do papel da ACS e de novas tecnologias em curso.

Esta Diretriz tem o objetivo de identificar e discutir as questões mais relevantes sobre doença carotídea, bem como elaborar recomendações para auxiliar os especialistas em sua prática clínica. As seguintes questões foram formuladas:

- Rastreamento para estenose carotídea assintomática: quando realizar?
- Quais medidas clínicas adotar no tratamento da estenose carotídea assintomática?
- Quais fatores elevam o risco de AVCI em indivíduos com estenose carotídea assintomática sob tratamento clínico?
- Quando e como revascularizar uma estenose carotídea assintomática?

### Rastreamento para estenose carotídea assintomática: quando realizar?

A utilidade do rastreamento populacional fundamenta-se na capacidade de identificar uma estenose carotídea grave e tomar medidas para, eventualmente, mudar o curso da doença e prevenir uma isquemia cerebral. O custo-benefício de se realizar exames para detectar uma estenose carotídea depende da prevalência de lesões que demandem tratamento em determinada população. Devido a uma prevalência baixa, menor que 5% de estenoses carotídeas significativas na população geral^61^ , há uma unanimidade de conduta entre todas as recomendações mais recentes sobre doença carotídea: não se recomenda rastreamento na população geral^5,9,62,63^ . Este parece justificável nos subgrupos onde a prevalência de estenose ultrapassa 20%^61^ . No entanto, identificar portadores de estenose carotídea é uma oportunidade de estimular a aderência do paciente ao tratamento clínico otimizado (TCO) e atuar mais efetivamente sobre os fatores de risco, contribuindo para redução da morbimortalidade cardiovascular. Em algumas populações específicas, com múltiplos fatores de risco para aterosclerose, a prevalência de doença carotídea assintomática se eleva de maneira relevante, justificando a pesquisa. São consideradas populações de risco para doença carotídea os portadores de HAS, doença arterial coronariana, DAP, hipercolesterolemia, diabetes melito (DM), insuficiência renal crônica, sopro cervical, infartos silenciosos em exame de neuroimagem, os tabagistas e aqueles com antecedente familiar de primeiro grau de isquemia cerebral. A presença de dois, três ou quatro fatores de risco eleva a prevalência de estenose carotídea ≥ 50%, de 2% para respectivamente 14%, 16% e 67%^64,65^ .

Considerando que o rastreamento somente é plausível na presença de um teste confiável, o eDc é perfeitamente adequado. É o método mais utilizado para identificar lesões carotídeas assintomáticas extracranianas, com sensibilidade e especificidade de 94% e 92%, respectivamente, para estenoses de 60-99% de obstrução, embora a sua acurácia seja examinador dependente^66^ . Quando se tem a intenção de tratar lesões assintomáticas, o exame também pode ser útil para o acompanhamento de estenoses ≥ 50% a cada 6 a 12 meses, com o propósito de identificar progressão do grau de estenose^62^ .

### Recomendação 3.1.1

O rastreamento populacional de doença carotídea assintomática não é recomendado para a população geral (1/B)^67^ .

### Recomendação 3.1.2

O rastreamento populacional de doença carotídea assintomática deve ser recomendado em indivíduos com mais de 55 anos que apresentem os seguintes fatores de risco para aterosclerose (IIb/C)^61,68^ :

1. HAS;
2. DAP;
3. doença obstrutiva coronariana;
4. tabagismo;
5. hipercolesterolemia;
6. DM;
7. insuficiência renal crônica;
8. sopro cervical;
9. infartos silenciosos em exame de neuroimagem;
10. antecedente familiar de primeiro grau.

### Recomendação 3.1.3

O exame de escolha para rastreamento de doença carotídea assintomática é o eco-Doppler (I/B)^10,64,66^ .

### Recomendação 3.1.4

Recomenda-se a realização de eco-Doppler de carótidas a cada 6-12 meses para estenoses assintomáticas > 50%. Opinião de painel de especialistas^5,62^ .

### Recomendação 3.2

O rastreamento de doença carotídea por meio de eco-Doppler deve ser considerado em indivíduos com mais de 55 anos que apresentem fatores de risco para aterosclerose (IIb/C)^10,61,64,66,68^ .

### Quais medidas clínicas adotar no tratamento da estenose carotídea assintomática?

O TCO para a doença carotídea obstrutiva ganhou grande projeção nas últimas duas décadas, ao ponto de colocar em xeque um dos procedimentos cirúrgicos mais realizados ao redor do mundo: a revascularização carotídea. Trouxe à tona a ideia de que o TCO teria mudado radicalmente a história natural da doença da bifurcação carotídea e de que qualquer intervenção em um paciente assintomático poderia ser considerada obsoleta ou até mesmo prejudicial. No entanto, o TCO, embora tenha amenizado o impacto da doença aterosclerótica, de forma global, ainda apresenta deficiências em vários aspectos^69^ .

De eficácia inquestionável, o TCO deve ser implementado em todos os portadores de estenose carotídea, sintomáticos ou assintomáticos, independentemente de serem submetidos a algum tipo de intervenção, com benefícios claros quanto à redução de isquemia cerebral e de mortalidade cardiovascular. Embora o alvo final do tratamento clínico, nesse caso, seja a prevenção da isquemia cerebral, seu impacto na redução de mortalidade cardiovascular é ainda mais relevante^47^ . O TCO, como peça chave no tratamento da doença carotídea, deve reunir mudanças no estilo de vida, controle rigoroso dos fatores de risco e terapias farmacológicas, cuja relevância vamos discutir a seguir.

### Controle dos fatores de risco

As mudanças de estilo de vida e consequentemente melhor controle dos fatores de risco para doença cardiovascular são, com frequência, subestimadas tanto pelo paciente quanto pelo médico. Talvez por ser a etapa mais difícil de se obter sucesso, pela necessidade de esforço excessivo de ambas as partes.

O tabagismo, por exemplo, dobra a chance de um indivíduo ter estenose carotídea acima de 50% e aumenta de 2 a 6 vezes a chance de se ter um AVCI, quando comparamos às taxas em não fumante^45,69^ . O monóxido de carbono e o tiocianato, entre outras substâncias contidos no tabaco, promovem disfunção endotelial, redução do HDL-colesterol, aumento dos triglicérides e do LDL-c, além de ativar plaquetas e promover um estado protrombótico. O tabaco também é responsável por reduzir os efeitos benéficos das estatinas, promover vasoconstrição e acelerar a aterosclerose, relacionando-se a ocorrência de progressão de placa. O fumo passivo e o consumo de outras substâncias, como maconha, são igualmente prejudiciais. Devemos não somente orientar o paciente a cessar o consumo de cigarros, mas também providenciarmos assistência para isso, indicando um especialista ou prescrevendo medicamentos disponíveis e sabidamente eficazes, como os substitutos não tóxicos da nicotina em forma de adesivos e gomas, por exemplo. Ainda mais eficientes são os medicamentos que agem no receptor da nicotina, como a bupropiona ou a vareniclina. O uso de bupropiona dobra a chance de um paciente parar de fumar, e a vareniclina é considerada ainda mais eficaz^45,69^ .

A obesidade promove alterações da complacência arterial. Esse endurecimento da parede das artérias está relacionado com disfunção do endotélio e das células musculares lisas da parede do vaso, com aumento da resistência periférica à insulina e elevação de taxas de colesterol e peptídeo C nos obesos. Essas características correlacionam-se com aumento de risco cardiovascular de forma global. Um marcador objetivo dos efeitos da obesidade é o espessamento médio intimal medido na carótida comum e classicamente associado a um elevado risco de AVC e doença cardiovascular^31^ .

A realização de qualquer atividade física reduz a chance em 20% de se ter uma estenose carotídea, e a maior frequência do exercício se correlaciona com a redução ainda maior de risco. Todo paciente com estenose carotídea deve receber recomendação de fazer no mínimo 30 minutos de exercício ao dia, de moderada intensidade, de forma que não fique mais de 2 dias consecutivos sem atividade física^32^ . Outra mudança de grande impacto no tratamento clínico da doença carotídea está relacionada com a dieta. A dieta mediterrânea à base de vegetais, frutas, grãos, azeite de oliva, com pouco leite e peixe e pouquíssima carne vermelha, permitindo um consumo baixo a moderado de álcool em forma de vinho, é a mais recomendada^45^ .

Em relação ao controle dos fatores de risco, a HAS provavelmente é o fator mais impactante na elevação de risco de AVC, seja isquêmico ou hemorrágico. Setenta e sete por cento dos pacientes com algum tipo de AVC são portadores de HAS, e este também é fator de risco para ocorrência de estenose carotídea^70^ . O mais importante é que a HAS é um fator de risco modificável, ou seja, temos medidas eficazes para reduzir a PA, aliadas às mudanças do estilo de vida. A cada 2 mmHg que reduzimos na medida da PA observamos uma redução de risco (RR) de AVC de 25%. Não há estudos clínicos randomizados que tenham avaliado o efeito dos anti-hipertensivos em indivíduos com estenose carotídea assintomática, mas o que se sabe de outros estudos, desenhados para indivíduos sem história de doença vascular, é que a RR de AVC chega a 45% com o controle da pressão, redução diretamente influenciada pela redução da pressão sistólica^71^ .

É provável que o DM seja o fator de risco modificável mais negligenciado por médicos e pacientes, dada a dificuldade de manter controle glicêmico estrito. Dezesseis por cento dos pacientes diabéticos acima de 65 anos morrem de AVC. Existe uma associação de aumento de risco de ocorrência de estenose carotídea nos diabéticos, e devemos encorajar o controle glicêmico^63^ .

### Antiagregantes e estatinas

Nas últimas duas décadas, houve grande aumento no uso dos medicamentos que hoje consideramos como pilares no tratamento da aterosclerose: AAP, anti-hipertensivos, betabloqueadores e, especialmente, as estatinas. Essa prática resultou em evidente RR de AVC e de mortalidade cardiovascular^69^ .

A antiagregação plaquetária é um fator independente na redução de AITs e AVC ipsilateral a uma estenose carotídea. No entanto, o seu benefício é ainda maior na redução de mortalidade por IAM, já que mais de 2/3 dos portadores de estenose carotídea assintomática apresentam doença obstrutiva coronariana associada. O AAS em baixas doses (75 a 325 mg/dia), além de reduzir a mortalidade cardiovascular, melhora o prognóstico do AVC quando ele ocorre. Não há benefício comprovado para dupla antiagregação plaquetária nos indivíduos assintomáticos, e o clopidogrel deve ser reservado como monoterapia para casos de intolerância a AAS^40^ .

Nas últimas décadas, houve um incrível aumento no uso das estatinas. Seu impacto na evolução da doença aterosclerótica é indiscutível, embora seu mecanismo de ação ainda não esteja totalmente elucidado^69^ . As estatinas são inibidores competitivos da hidroximetilglutaril coenzima A redutase, com propriedades de limitar a síntese intra-hepática de colesterol, de aumentar o *turnover* dos receptores de LDL-c (*low-density lipoprotein*), de reduzir a produção de VLDL-c (*very low-density lipoprotein*) e, de forma menos importante, elevar a produção de HDL-c (*high-density lipoprotein*). É provável que a somatória dessas propriedades culmine com estabilização de placa de ateroma, reversão da disfunção endotelial e redução de trombogenicidade.

Quando analisamos os indivíduos acompanhados no ACST-1, o risco de AVC e óbito em 10 anos dos portadores de estenose carotídea assintomática sob tratamento clínico e tratados por EC foi respectivamente de 13,4% e 7,6%^47^ . No entanto, se focarmos no subgrupo que não usou estatina, o risco se elevou para 24,1% nos indivíduos sob tratamento clínico e 17,9% nos operados, corroborando a eficácia das estatinas no tratamento a longo prazo para a estenose carotídea^47^ . Os pacientes devem ser tratados a longo prazo com objetivo de reduzir LDL-c abaixo de 70 mg/dL ou a 50% do LDL-colesterol inicial^72^ .

### Recomendação 3.3.1

O TCO deve ser implementado em todos os pacientes com estenose carotídea, independentemente do grau de estenose, para prevenção de eventos cardiovasculares (I/b)^45,47,69^ .

### Recomendação 3.3.2

Devem ser adotadas as seguintes medidas para o tratamento clínico da estenose carotídea assintomática:

1. dieta saudável, exercício físico (I/B)^45,73^ ;
2. AAS em baixas doses (75 a 325 mg) para estenoses > 50% (I/A)^41,45^ ;
3. clopidogrel 75 mg/dia se intolerância/alergia a AAS (IIa/B)^40^ ;
4. estatinas: atorvastatina 40-80 mg/dia ou rosuvastatina 20-40 mg/dia. LDL-c deve ser reduzido a < 70 mg/dL ou < 50 mg/dL^47,48^ ;
5. antihipertensivos com alvo de PA < 140x90 mmHg e < 140x85 mmHg em diabéticos (I/A)^34,35^ ;
6. controle da glicemia nos diabéticos com HbA1c < 7% (I/C)^74^ ;
7. cessação de tabagismo, encorar auxílio de medicamentos (bupropiona, vareniclina, nortriptilina, reposição de nicotina) (I/B)^75^ ;
8. utilização de inibidores da PCSK9 se houver intolerância a estatinas/ezetimiba (IIa/C)^51^ .

### Quais fatores elevam o risco de AVC em pacientes com estenose carotídea assintomática sob tratamento clínico?

A primeira menção sobre a existência de um grupo de pacientes portadores de estenose carotídea assintomática “altamente selecionados”, por estarem sob risco elevado de AVCI, e que se beneficiariam de revascularização carotídea, foi feita em 2011, em uma publicação da American Heart Association (AHA), sem, no entanto, discriminar claramente esses pacientes^74^ . Impulsionadas pelos avanços do tratamento clínico nos últimos 30 anos, promovendo taxas excepcionais de 1% de AVC anualmente, a necessidade de reavaliar os critérios de indicação para revascularização carotídea se tornou iminente. Outros estudos elencando fatores responsáveis por elevar o risco de AVCI, relacionados à morfologia da placa de ateroma e à presença de embolias silenciosas foram realizados^76,77^ .

Em 2017, a European Society for Vascular Surgery (ESVS) recomendou nas suas Diretrizes, pela primeira vez, que a indicação de revascularização carotídea em indivíduos assintomáticos com estenose de 60-99% estaria condicionada à presença de uma ou mais características de imagem associadas à elevação de fator de risco de AVC ipsilateral, desde que o risco cirúrgico fosse < 3%^72^ .

Uma revisão sistemática publicada em 2020 descreveu, pela primeira vez na literatura, as características de uma placa de alto risco, sua relação com elevação de risco de AVCI acima das estimativas aceitáveis e sua prevalência de 26,5%, levando a concluir que informações adicionais, além do grau de estenose, poderiam auxiliar a melhor identificar pacientes sob elevado risco de AVC^77^ .

O papel do grau de estenose no risco de AVCI permanece em debate. Em última análise, os estudos Asymptomatic Carotid Stenosis and Risk of Stroke Study Group (ACSRS), o estudo Oxford e uma revisão sistemática recente da literatura reafirmaram o grau de estenose como marcador de risco, ao identificar taxas de AVCI de 5% em estenoses moderadas, e de 15% em estenoses acentuadas sob tratamento clínico^78-80^ . Dessa forma, para termos maior eficácia na seleção dos pacientes para revascularização carotídea, os marcadores de elevação de risco (imagem/clínico) não devem substituir o grau de estenose e, de fato, devem ser devidamente ajustados a ele. Os fatores de elevação de risco (imagem/clínico) de AVCI estão descritos no Quadro 6.

**Quadro 6:** Fatores que elevam o risco de AVC em pacientes com estenose carotídea assintomática sob tratamento clínico.

**Característica clínica/imagem**	**Risco anual AVC ipsilateral/OR**	**Referências**
1. Infartos silenciosos na TC	3,6% / 3,0	Kakkos et al.^81^
2. AVC contralateral	3,4% / 3,0	Nicolaides et al.^80^
3. Progressão da estenose		Conrad et al.^82^ , Kakkos et al.^83^
a. 50-99%	2,0% / 1,92	
b. 70-99%	4,7%	
4. Área da placa análise computadorizada por ECO (70-99%)		Nicolaides et al.^84^
a. < 40 mm^2^	1% / 1	
b. 40-80 mm^2^	1,4% / 2,08	
c. > 80 mm^2^	4,6% / 5,81	
5. Zona preta justaluminal na análise computadorizada por ECO (50-99%)		Kakkos et al.^85^
a. < 4 mm^2^	0,4%	
b. 4-8 mm^2^	1,4%	
c. 8-10 mm^2^	3,2%	
c. >10 mm^2^	5,0%	
6. Hemorragia intraplaca na RM (50-99%)	/3,66	Gupta e Marshall^86^
7. Reserva cerebral diminuída (70-99%)	/6,14	King et al.^87^
8. Ecolucência da placa pelo ECO		Gupta et al.^88^
a. predominantemente ecolucente (50-99%)	4,2% / 2,1	
b. predominantemente ecogênica (50-99%)	1,6%	
9. Embolização espontânea no DTC (50-99%) > 1 embolização espontânea em 1 hora	/7,46	Gupta et al.^88^
10. Embolização espontânea no DTC (50-99%) mais placa predominantemente ecolucente no ECO (70-99%)	8,9% / 10,61	Markus et al.^89^
11. Grau de estenose medido por dois exames de ECO ou AngioTC/angiografia		Kamtchum-Tatuene et al.^78^ , Howard et al.^79^ , Nicolaides et al.^80^
a. 70-99%	14,6% / 5 anos	
b. 80-99%	18,3% / 5 anos	
c. 50-69%	1% / 5 anos	

TC: tomografia computadorizada; ECO: ecografia vascular; RM: ressonância magnética; DTC: doppler transcraniano. Modificada de Naylor et al.^5^ .

### Quando e como revascularizar uma estenose carotídea assintomática?

A escolha do tratamento que mais beneficiaria um paciente portador de uma estenose carotídea assintomática é produto da interação de questões não somente técnicas, mas culturais, políticas, financeiras e filosóficas. Por essa razão, hoje temos práticas divergentes recomendadas por grandes serviços formadores de opinião e embasadas por evidências científicas que ainda são questionáveis e exigem maior validação e debate. Ao analisar a indicação de revascularização carotídea baseada na presença de sintomas, encontramos serviços em que a revascularização ocorre predominantemente em indivíduos assintomáticos, como nos EUA (70%), na Alemanha e na Itália (60%), e cenários onde revascularizar assintomáticos é uma exceção, como no Reino Unido, Canadá e Austrália (15%), ou ainda na Dinamarca, onde assintomáticos não são revascularizados^90,91^ . É importante ressaltar que cerca de 10-15% de todos os AVCIs isquêmicos ocorrem devido ao tromboembolismo proveniente de uma estenose carotídea de mais de 50%, que era previamente assintomática^63^ .

De acordo com a última recomendação da Sociedade de Cirurgia Vascular Norte-Americana (SVS), para pacientes com estenose carotídea assintomática acima de 70%, com baixo risco cirúrgico, recomenda-se EC, associada a TCO, como medida para prevenção de AVCI a longo prazo (grau I de recomendação e nível de evidência B)^9^ . O tratamento é escolhido de acordo com a gravidade da estenose, o risco para a intervenção proposta e a probabilidade desse procedimento alterar o curso da doença. Essa recomendação fundamenta-se nos resultados de estudos multicêntricos randomizados e controlados (RCT) VACS^92^ , ACAS^22^ e ACST^47^ , que demonstraram a superioridade da EC sobre o tratamento clínico isolado na prevenção de AVCI em longo prazo (por exemplo, 13,3% de AVCI na EC e 17,9% no tratamento clínico de indivíduos assintomáticos acima de 75 anos, em 10 anos).

A controvérsia a respeito das condutas embasadas por esses dados ressalta a evolução do tratamento clínico contemporâneo com a adição de estatinas e outras medidas rigorosas de controle dos fatores de risco, culminando com taxas anuais de AVCI de 0,9%, o que poderia impactar de forma relevante a taxa já considerada modesta, demonstrando a superioridade da EC nesses casos^93^ . É fato que ainda não temos estudos concluídos adequados e desenhados para comparar EC e TCO, que demonstrem que o tratamento clínico isoladamente seja adequado para todos os pacientes com estenose carotídea assintomática.

Quando analisamos os dados disponíveis de estudos mais recentes, notamos que a inclusão de grande número de pacientes com estenoses moderadas, de 50-69%, cujo risco de AVCI sob tratamento clínico é baixo, induz a subestimar o benefício da EC em pacientes com estenoses mais graves^79,94^ . O estudo ACSRS, o maior e mais recente sobre a história natural da estenose carotídea assintomática, registrou que as estenoses de 70-89% se mostraram fortemente associadas ao risco de AVCI ipsilateral (15% em 5 anos), bem como as de 90-99% (20% em 5 anos), independentemente do uso de estatinas e de tabagismo, quando comparadas às estenoses moderadas de 60-69% (5% em 5 anos)^80^ . Esses resultados foram corroborados por outros estudos contemporâneos, como o Oxford Vascular Study e uma metanálise de 23 estudos, sugerindo que embora as taxas de AVCI tenham declinado ao longo do tempo com o tratamento clínico, os pacientes com estenoses graves permanecem sob risco elevado de isquemia cerebral^79,94^ .

Como medida para viabilizar a prática clínica e uniformizar as condutas em relação à estenose carotídea assintomática, o grau de estenose se reafirma como uma ferramenta simples, barata e confiável como marcador de risco de AVCI; não é prudente menosprezar o grau de estenose em favor de outros marcadores de risco parcialmente validados, como a análise de morfologia de placa por exames de imagem (eDc, ATC e ARM), mas, sim, de forma ideal, adotarmos todas as ferramentas adequadas e disponíveis para seleção dos pacientes^95-97^ .

Não somente o grau de estenose, mas também a progressão da placa se comportou como fator relevante de elevação de risco de AVCI. Quando comparamos o risco de AVCI em pacientes tratados clinicamente de acordo com o grau de estenose e sob o ponto de vista da progressão da placa, temos um risco de AVCI em 8 anos de 4% (50-69%), de 8% (70-89%) e de 13% (90-99%) nos pacientes em que as placas permaneceram estáveis no período; no entanto, nos pacientes em que houve progressão da lesão, os riscos se elevaram para 8%, 15% e 25%, respectivamente^80,83,98^ . Vale ressaltar que o risco de AVCI em pacientes com estenoses assintomáticas menores de 60%, por ora, não justifica intervenção, embora a presença de placas consideradas de alto risco mereçam estudos futuros, independentemente do grau de estenose.

No que se refere ao TCO, sua eficiência é inegável tanto para mitigar as taxas de AVCI quanto as de outros eventos cardiovasculares. No entanto, devemos ponderar sobre a sua inabilidade em impedir a progressão da placa de ateroma com consequente oclusão carotídea^45^ . A oclusão carotídea, cuja história natural foi, por muito tempo, erroneamente considerada benigna, é responsável por aproximadamente 40% dos AVCIs relacionados à estenose carotídea sem sintomas prévios, bem como pelo risco elevado ao longo da vida de AVCI ipsilateral à oclusão^99,100^ . Este fato nos leva a inferir que o tratamento clínico isoladamente não seria uma alternativa terapêutica a ser utilizada indiscriminadamente para todos os pacientes.

Em busca de um ponto de equilíbrio, a ESVS propôs uma seleção mais criteriosa e a adoção de outros parâmetros para selecionar subgrupos de risco e indicar a revascularização, certa de que o uso do grau de estenose como critério único de seleção promoveria número excessivo de procedimentos desnecessários, em que somente 15% dos operados se beneficiariam^101^ .

As Diretrizes da ESVS, de 2017 e de 2023, propuseram que se identifique pacientes portadores de estenose carotídea e que estejam sob elevado risco de AVCI utilizando alguns critérios clínicos e outros relacionados especialmente à morfologia da placa de ateroma e à presença de isquemias silenciosas^5,63^ . Esses critérios foram eleitos de acordo com resultados de revisões sistemáticas, estudos multicêntricos, análise de subgrupos de RCT, e não por meio de uma única fonte de dados^77-80^ . São eles: infarto silencioso diagnosticado na TC ou na RM^80^ ; presença de AVCI contralateral^80^ ;-progressão da estenose em ≥ 20%^83^ ; presença de placas com grandes áreas (> 40mm^2^)^84^ ou com grande “área preta” justaluminal (área com *gray scale median* < 25)^102^ adjacente à luz do vaso, sem que se possa identificar uma capa fibrosa após a normalização da imagem e área de mais de 4 mm^2^ identificada na análise computadorizada por ultrassonografia^85^ ; placas predominantemente ecolucentes (lipídicas) ao eDc^88^ ; hemorragia intraplaca na RM^88^ ; reserva cerebral comprometida no DTC por análise da velocidade média na ACM durante inalação de CO_2_ ou apneia^87^ ; presença de ≥ 1embolização espontânea presente nos registros de ≥ 1 hora de DTC^89^ .

Utilizando esses parâmetros, em teoria, pode-se selecionar cerca de 25% de indivíduos assintomáticos que apresentam ao menos um dos critérios de risco e que realmente se beneficiariam de revascularização, conforme defendem alguns autores^78^ . Partindo dessa premissa, a Diretriz da ESVS 2023 recomenda que indivíduos assintomáticos com estenose de 60-99%, sem risco cirúrgico elevado e com um ou mais critérios que elevem risco de AVCI, devam ser submetidos à EC, desde que a taxa de AVC/óbito perioperatórios seja < 3%, e que a expectativa de vida seja > 5 anos (Classe de Recomendação IIa e nível de evidência B – ver Recomendação 3.4.1).

Os dados acima descritos demonstram pontos de vista distintos e fundamentados, que dividem o espaço nos meios mais respeitados de difusão do conhecimento sobre doença carotídea, o que nos leva a concluir que não é possível adotarmos uma fórmula resolutiva para todos os pacientes com doença carotídea assintomática. É essencial que se pondere as particularidades de cada indivíduo (idade, sexo, comorbidades), sua capacidade de se adequar ao tratamento clínico e suas preferências na tomada de decisão sobre o melhor tratamento.

### Recomendação 3.4.1

A revascularização carotídea deve ser considerada em pacientes assintomáticos com estenose 60-99% com risco cirúrgico < 3% e expectativa de vida > 5 anos que apresentem um ou mais fatores de elevação de risco para AVCI (IIa/B)^22,47,80,83,84^ .

### Recomendação 3.4.2

A revascularização carotídea deve ser considerada em pacientes assintomáticos com estenose > 70% com risco cirúrgico < 3% e expectativa de vida > 5 anos, considerando as necessidades e preferências individuais de cada paciente (I/B)^22,47,79,94^ .

Uma vez que se decide revascularizar o portador de estenose carotídea assintomática, outra controvérsia se instala para eleger a técnica mais adequada, se EC ou ACS. Atualmente, todas as recomendações vigentes priorizam a EC como procedimento mais adequado aos pacientes com estenoses ≥ 60%, ou > 70%, com baixo risco cirúrgico (menos de 3%) e boa expectativa de vida^5,9,62,103^ . Em última análise, essa conduta está nitidamente influenciada pelos excelentes resultados atingidos recentemente pela EC, com taxas de AVCI e óbito inferiores a 3% nos pacientes assintomáticos, levando alguns autores a propor que se reduza a taxa ideal recomendada para se indicar uma intervenção em paciente assintomático, de 3 para 2%^9,78,103^ .

A ACS surgiu como uma alternativa atraente por ser menos invasiva do que a EC. Os resultados de RCTs mais recentes, o CREST e o ACTI foram otimistas e demonstraram resultados equivalentes de AVC maior ipsilateral no perioperatório, refletindo a habilidade dos profissionais e dos centros selecionados para participar dos estudos^104,105^ . O que diferiu no CREST entre os grupos EC e ACS foi a incidência de AVC menor em 30 dias, maior no grupo *stent* (2,4% *versus* 1,1%); no entanto, houve incidência mais baixa de IAM no grupo ACS, porém sem impactar a taxa de mortalidade. Entretanto, essa equivalência de resultados entre as duas técnicas não se confirmou em sucessivos registros contemporâneos da prática clínica habitual.

Uma metanálise de 21 registros, envolvendo mais de 1.500.000 procedimentos, mostrou taxas de AVC pós-implante de *stent* transfemoral significativamente maiores em 11 dos 21 registros (52% deles), excedendo os 3% preconizados como taxa de complicação aceitável para indivíduos assintomáticos, enquanto somente em um registro (5% deles) a taxa de AVC após endarterectomia excedeu o risco recomendado^106^ . Em outra análise, com dados do Vascular Quality Initiative (VQI) da SVS, o *stent* transfemoral esteve associado com maior risco de complicações maiores em 30 dias, óbito, AVC ou infarto, quando comparado à endarterectomia (4,6% *versus* 1,97%)^9,107^ . O ACST-2, o maior RCT já realizado para comparar ACS e EC, confirmou, em seus resultados preliminares, os achados de RCTs anteriores, com similaridade de desfechos entre os grupos para AVC, maior ipsilateral e óbito em 30 dias. No entanto, quando se avalia qualquer evento isquêmico em 30 dias, houve um aumento do número de eventos no braço ACS (3,6% *versus* 2,4%), particularmente de AVCI não incapacitante (2,7% *versus* 1,6%). Durante o acompanhamento de 5 anos, não houve diferença entre os dois grupos quanto à incidência de AVCI incapacitante (0,5% ao ano)^108^ .

A última revisão Cochrane, com mais de 3.000 pacientes avaliados, corrobora os resultados do ACST-2. No entanto, relata uma forte tendência de que a EC apresenta risco mais baixo de qualquer AVC e óbito periprocedimento^109^ . As evidências atuais não nos permitem tirar conclusões sobre a segurança e a eficácia dos dois procedimentos. Não há dados suficientes que justifiquem a recomendação de ACS transfemoral em indivíduos assintomáticos.

A nova tecnologia para realização de ACS via transcarotídea e sob fluxo carotídeo reverso (TCAR) apresentou resultados preliminares muito promissores e muito semelhantes aos da endarterectomia, surgindo como uma ferramenta alternativa útil, especialmente nos pacientes considerados de alto risco para EC^110^ . Na presença de elevação de risco cirúrgico para endarterectomia, ACS e TCAR podem ser considerados como uma alternativa factível^9,110,111^ . Considerando que cada uma das técnicas disponíveis para tratamento da estenose carotídea (EC, ACS e TCAR) apresenta limitações e vantagens, é razoável que utilizemos essas características racionalmente em nosso favor, em prol de minimizar riscos e reduzir as taxas de AVC em longo prazo.

Enquanto aguarda novas recomendações dos estudos em andamento, que comparam tratamento clínico com endarterectomia ou angioplastia em pacientes assintomáticos (CREST-2, ACTRIS, ECST-2,), o especialista deve utilizar da combinação de ferramentas para diagnóstico e evidências científicas disponíveis para que, empregando seu julgamento clínico, escolha um tratamento focado nas características e necessidades individuais de cada paciente, visando ao melhor resultado imediato e de longo prazo.

### Recomendação 3.5.1

A EC é o procedimento de escolha para pacientes assintomáticos com estenose 70-99% com risco cirúrgico < 3% e expectativa de vida > 5 anos que tenham indicação de revascularização (IIa/B)^22,47,80,83,84,106,107^ .

### Recomendação 3.5.2

Na presença de anatomia favorável, a ACS pode ser uma alternativa em pacientes assintomáticos com estenose 70-99% com risco cirúrgico < 3% e expectativa de vida > 5 anos que tenham indicação de revascularização (IIb/B)^92,105,107,110,111^ .

### Recomendação 3.6.1

Deve ser considerada a realização de EC em pacientes assintomáticos que apresentem (IIa/B)^22,47,80,83^ :

1. estenose 70-99%;
2. risco cirúrgico adequado;
3. > 1 critério de imagem que eleve o risco de AVCI;
4. taxa de AVC/óbito perioperatório < 3%;
5. expectativa de vida > 5 anos;
6. Baixo risco cirúrgico.

O tratamento é escolhido com base na gravidade da estenose, no risco da intervenção proposta e na probabilidade do procedimento alterar o curso da doença.

### Recomendação 3.6.2

A ACS pode ser uma alternativa em pacientes assintomáticos que apresentem (IIb/B)^80,93,105^ :

1. estenose 70-99%;
2. risco cirúrgico adequado;
3. > 1 critério de imagem que eleve o risco de AVCI;
4. taxa de AVC/óbito perioperatório < 3%;
5. expectativa de vida > 5 anos;
6. anatomia favorável.

### Recomendação 3.6.3

A ACS pode ser considerada em pacientes assintomáticos que apresentem (IIb/B)^9,110,111^ :

1. estenose 70-99%;
2. risco elevado para EC;
3. taxa de AVC/óbito perioperatório < 3%;
4. expectativa de vida > 5 anos;
5. anatomia favorável.

## DOENÇA CAROTÍDEA SINTOMÁTICA

### Introdução

As manifestações clínicas da doença cerebrovascular extracraniana são causadas por isquemia – redução aguda do fluxo sanguíneo para o cérebro ou para o globo ocular^112^ . O quadro clínico – sintomas e sinais – resultante da isquemia é chamado evento isquêmico cerebral ou ocular, quando atinge exclusivamente os olhos. O termo doença carotídea sintomática se refere ao paciente que desenvolve um ou mais eventos isquêmicos no território carotídeo nos 6 meses anteriores.

### Definições

Existe confusão na literatura quanto às definições do momento de um evento isquêmico cerebral^113^ . Evento inicial (ou primeiro evento) é o primeiro sintoma relatado pelo paciente. Evento índice é o sintoma que leva o paciente a procurar cuidados médicos. Evento recorrente é quando o mesmo sintoma se repete, após um período sem sintomas. Evento mais recente é, como o próprio nome indica, o que ocorreu por último na evolução clínica do paciente.

Os eventos isquêmicos são definidos pela duração da apresentação clínica, suplementada pelos achados de métodos de imagem, quando disponíveis no sistema de saúde.

AIT – Acidente Isquêmico Transitório é definido pela Organização Mundial de Saúde (OMS) como “déficit neurológico focal com duração menor do que 24 horas, que tem etiologia vascular.”^114^ A AHA propõe um refinamento na definição de AIT, baseada nos achados dos exames de imagem (ATC ou RM): “um episódio breve de disfunção neurológica resultante de isquemia focal temporal, que não está associada com infarto agudo cerebral.”^115^

AVCI – Acidente Vascular Cerebral Isquêmico: da mesma forma, a OMS define o AVC como “déficit neurológico focal ou eventualmente global, com duração maior do que 24 horas, que tem etiologia vascular.”^114^

Amaurose fugaz (ou cegueira monocular transitória): é definida como “perda indolor temporária da visão de um olho, de etiologia vascular”.^116^ A perda visual pode ser permanente quando ocorre oclusão da artéria central da retina.

Outras manifestações de isquemia cerebral são definidas por termos específicos:

AIT em *crescendo* (AITc): múltiplos AITs em um curto período de tempo, com recuperação completa entre os eventos. O número exato de eventos que constituem um AITc não está definido.

AVC em evolução ou flutuante (AVCe): déficits neurológicos variáveis, sem recuperação funcional plena ao longo do tempo.

### Fisiopatologia da isquemia cerebral

De 15-25% dos eventos cerebrais isquêmicos são causados por lesões ateroscleróticas em grandes vasos, em especial na carótida extracraniana^117^ . Os outros 75-85% são causados por embolia de origem cardíaca, por oclusão de pequenas artérias cerebrais (infartos lacunares) e acidentes isquêmicos cerebrais de origem indeterminada.

### Aterosclerose da carótida

A carótida é uma das artérias mais afetadas pela aterosclerose. Placas de ateroma podem se formar em toda a extensão da carótida, da origem da carótida comum na aorta ou no tronco braquiocefálico, até o segmento intracraniano da carótida interna. Estudos anatomopatológicos mostram que mais de 80% das placas de ateroma se localizam na bifurcação da carótida comum, que se estende ao segmento inicial da carótida interna, denominado bulbo carotídeo.

### A placa aterosclerótica

A lesão característica da aterosclerose é a placa aterosclerótica ou placa de ateroma, uma lesão complexa da camada íntima da parede arterial, formada por um núcleo central de detritos de colesterol, macrófagos e células inflamatórias, cercada por camadas de músculo liso e tecido conjuntivo e por uma capa fibrosa que separa o núcleo da luz arterial. A placa aterosclerótica geralmente cresce lentamente, ocupando a luz da artéria e provocando estenose local com redução do fluxo sanguíneo para o leito distal. É conhecido que a progressão da placa não é linear, podendo haver um ou mais episódios de crescimento rápido, decorrentes de eventos intraplaca^118,119^ .

### O conceito de placa vulnerável

A placa de ateroma é um tecido vivo, que sofre mudanças na morfologia e composição ao longo do tempo. Essas mudanças são geralmente lentas, como a formação de uma placa rica em colesterol ao longo de décadas; mas podem ocorrer subitamente, como uma hemorragia intraplaca ou necrose. As mudanças que acontecem em uma placa aterosclerótica podem torná-la instável, isto é, propensa a complicações, tais como embolização e trombose. As características que definem uma placa vulnerável incluem: um núcleo grande rico em lipídios; uma capa fibrosa tênue; inflamação dentro ou ao redor da placa; hemorragia intraplaca e neovascularização nos *vasa vasorum* ^118,119^ .

### Eventos intraplaca

O processo patológico mais comum é a ruptura da placa, com extrusão do seu conteúdo para a luz da artéria, que é levado pela corrente sanguínea para a carótida interna distal e seus ramos ao cérebro e também ao olho ipsilateral^118^ . Outro processo que resulta em embolização é a ulceração da placa de ateroma, com a formação de trombos na superfície da placa e embolização desses trombos para o leito arterial cerebral. O outro mecanismo de isquemia cerebral relacionado ao evento intraplaca é oclusão da carótida interna, que pode ser causada por hemorragia intraplaca, ou por progressão da placa para estenose de alto grau/suboclusiva, com trombose subsequente^112^ .

### Tempo de evolução dos sintomas

Pacientes com isquemia cerebral aguda – AIT, AVCI e isquemia de retina – devem ser diagnosticados precocemente e tratados, horas ou dias depois do evento isquêmico. O tempo de evolução da isquemia cerebral tem sido definido como agudo, recente e crônico, sumarizado no Quadro 7^120^ . O intervalo entre os sintomas iniciais e o início do atendimento é crucial para evitar/reduzir o dano causado pela lesão isquêmica e, consequentemente, reduzir as sequelas neurológicas e o risco de morte^121^ .

**Quadro 7:** Definição de sintomas quanto ao tempo de evolução.

**Definição**	**Tempo de isquemia**
Agudo	Até 2 semanas
Recente	2 semanas a 6 meses
Crônico	Mais de 6 meses

### Manejo do paciente com isquemia transitória e AVCI agudo

O manejo dos pacientes com instabilidade neurológica, classificados no Estágio III de Vollmar, é um dos temas que causa maior controvérsia na área da insuficiência vascular cerebral aguda^5,122^ . A pergunta sobre quando se pode intervir em uma lesão estenótica ou até obstrutiva após um evento cerebrovascular agudo, vem lentamente sendo respondida pela ciência. Apesar de termos testemunhado muitos avanços no diagnóstico e tratamento do AVCI e hemorrágico (AVCH) nas últimas décadas, ainda existem várias questões que não têm diretrizes claras e irrefutáveis. O mesmo se aplica a manifestações isquêmicas monoculares de etiologia carotídea, as amauroses, sobretudo sua forma fugaz^123,124^ . Os benefícios da EC na prevenção de novos eventos cerebrovasculares isquêmicos em paciente que sofreu isquemia cerebral transitória, reversível ou até com pequeno déficit neurológico residual, é inquestionável^125,126^ . No entanto, a oportunidade ideal de revascularizar após um AVCI agudo ainda não está claramente definida. A maioria das indicações se relaciona com o tempo decorrido entre o evento índice e a revascularização^63,127^ . Recentemente, cada vez mais ênfase é dada ao tamanho da área isquêmica identificada em estudos de imagens, sobretudo na RM em estudos de difusão^124,128-139^ .

Classicamente, um paciente sintomático com lesão carotídea relacionada com o evento somente seria operado após um mês^127^ . Esse tempo foi sendo reduzido progressivamente, mas um limite de pelo menos 14 dias é o mais preconizado na literatura^132^ . Mas, é conhecido que a taxa de recorrência de uma isquemia a cerebral é grande após um evento índice inicial, podendo chegar a mais de 1/4 dos casos em 14 dias^5,63,137,140,141^ . Embora Strömberg et al.^140^ tenham estimado em 13,5% a recorrência de isquemia cerebral em 30 dias, são muitas as publicações que confirmam pior prognóstico^8,140-144^ . Tsantilas et al.^141^ , em extensa metanálise, correlacionaram o risco precoce de um novo evento isquêmico após um AIT, amaurose fugaz ou AVCI não incapacitante em 6,4% dentro de 2-3 dias, de 19,5% dentro de 7 dias, e de 26,1% em até 14 dias. Estes dados são confirmados por várias publicações recentes^8,142-144^ .

É consenso que pacientes com AVCIs debilitantes, classificados em graus de mais de 3 na eRm^7,145^ , ou aqueles que apresentem isquemia de mais de 1/3 do território da ACM (a área mais afetada nos acidentes carotídeos), não devam ser operados antes de uma completa estabilidade neurológica, o que demora geralmente semanas^7,121,139,145^ . Hause et al.^136^ publicaram recentemente uma avaliação de 646 pacientes com isquemia carotídea recente, dos quais 56,8% foram submetidos à EC precoce, antes de 14 dias do evento índice. Usaram como determinante classificatório entre infartos grandes, aqueles com áreas isquêmicas de > 4 cm^3^, e pequenos se o volume fosse < 4 cm^3^ ou ausentes. Infartos pequenos foram aferidos em 266 pacientes (41,2%), e grandes em 101 casos (15,6%). Todos os pacientes com infartos menores ou ausentes evoluíram sem agravamento de déficits neurológicos após a cirurgia, mas agravamento e morte ocorreram em 12 dos 101 pacientes com infartos maiores (11,9% – 10 encefalopatias e duas transformações hemorrágicas)^136^ . Os cuidados com a HAS e o manejo de drogas antitrombóticas foram idênticos nos dois grupos^136^ . Dados semelhantes são apresentados em vários trabalhos recentes^128-139^ . Esses dados coincidem com artigo publicado recentemente, no qual 50 pacientes submetidos à cirurgia carotídea precoce após isquemia aguda, com áreas isquêmicas de até 3,4 cm^3^, tiveram excelente evolução, sem agravamentos neurológicos ou óbitos^124^ . Vale ressaltar que, nesse mesmo trabalho, 34% dos pacientes foram operados em menos de 24 horas, e 68% em até 5 dias^124^ .

### Recomendação 4.1.1.1

Pacientes com estenose carotídea entre 50-99%, apresentando déficit neurológico recente incapacitante (classificação eRm ≥ 2), mas sem alterações da consciência, com áreas isquêmicas isoladas e/ou múltiplas identificáveis na RM de difusão com volume menor que 4 cm^3^, devem ser operados o mais cedo possível (I/C)^128-139^ .

### Recomendação 4.1.1.2

Pacientes com estenose carotídea entre 50-99%, déficit neurológico recente incapacitante, classificados como mRs 2 ou menos e revascularizados precocemente devem ser submetidos a cuidado com níveis de hipertensão arterial no pré- e pós-operatório, visando minimizar os riscos de edema, síndrome de hiperperfusão cerebral (SHC) e hemorragia cerebral pós-revascularização (I/C)^5,124,128-139,146^ .

### Recomendação 4.1.1.3

Pacientes com estenose carotídea entre 50-99%, déficit neurológico recente incapacitante (classificação mRs 3 ou mais) e/ou alteração da consciência, com áreas isquêmicas isoladas e/ou múltiplas identificáveis na RM de difusão com volume maior que 4 cm^3^ ou área isquêmica comprometendo 1/3 ou mais do território da ACM, somente devem ser operados após plena estabilização dos sintomas neurológicos (I/C)^136,137,139^ .

Acreditamos que esta é uma área que nunca será objeto de estudos randomizados, pelas características dos pacientes envolvidos, portanto os níveis de evidência dificilmente ultrapassarão o nível B.

Existem poucos estudos centrados em AITs e AVCIs recidivantes ou repetitivos e AVCIs de evolução rápida. Essas manifestações são mais conhecidas internacionalmente pelos termos AITc e AVCe. O prognóstico dessas duas situações com tratamento farmacológico é muito ruim^147-149^ .

Embora um trabalho mostre que a antiagregação reduz a ocorrência de microêmbolos cerebrais detectados por Doppler transcraniano, o que não ocorre com o uso de heparina, outras publicações não mostram diferença nos resultados^147-150^ .

Os resultados de intervenções nos AITc e AVCe variam muito na literatura, com taxas de complicações de AVC e morte variando de 0-11% para os AITc, e de 2-20% para o AVCe^149^ . Todavia, aplicando-se os mesmos critérios acima para a revascularização carotídea em pacientes com instabilidade neurológica, as taxas obtidas ficam entre 2-8% para AVCe, e 0-2% para AITc^129,133,134,149^ . Nessas situações, mesmo sem evidências elevadas, devemos empregar heparina associada a AAS antes da realização de uma intervenção urgente.

Qual é o percentual de risco de AVC e morte que é considerado adequado nesse subgrupo de pacientes com instabilidade neurológica? As Diretrizes mais recentes da ESVS, baseadas nas evidências dos trabalhos de Biller et al.^151^ , Dellagrammaticas et al.^153^ e Naylor et al.^152^ , consideram que um risco acumulado de até 6% é adequado, pois a probabilidade de ocorrência de um déficit neurológico e/ou morte sem intervenção é muito superior^151-155^ .

### Recomendação 4.1.1.4

Pacientes com AITc ou AVEc devem receber AAP dupla de imediato e ser submetidos a EC o mais rápido possível, de preferência antes de decorridas 24 horas (IIa/C)^129,133,147-155^ .

### Manejo de pacientes sintomáticos com fibrilação atrial (FA) e estenoses carotídeas > 50%

Eventos isquêmicos no território carotídeo podem ocorrer sem que a fonte seja a lesão dessa artéria. Uma metanálise evidenciou que 12% dos pacientes com FA apresentavam estenoses carotídeas de mais de 50%, ao passo que 9% dos pacientes com estenoses carotídeas apresentavam FA^156^ . O assunto é motivo de grande debate^157,158^ . Essa é outra área que provavelmente nunca será objeto de um estudo randomizado, pelas características dos pacientes envolvidos. Prevalece o julgamento individual por uma equipe médica multidisciplinar e, se indicada uma intervenção carotídea, após esta o paciente deverá ser anticoagulado o mais rápido possível^156-159^ .

### Recomendação 4.1.2

Pacientes apresentando amaurose fugaz e/ou déficit neurológico recente, FA e estenose carotídea entre 50-99% simultaneamente devem ser avaliados por equipe médica multidisciplinar, a qual deve verificar especificamente as características emboligênicas da placa para decidir se anticoagulação isolada ou uma intervenção carotídea associada é a melhor opção. Após a intervenção, o paciente deve ser anticoagulado o mais rápido possível (I/C)^152,156-159^ .

### Trombos carotídeos flutuantes

Trombos carotídeos flutuantes têm sido detectados com mais frequência recentemente, pela disseminação dos estudos com eDc no cenário de instabilidade neurológica e pela melhor resolução espacial dos aparelhos mais modernos dessa modalidade diagnóstica. Eventualmente, também são detectados em angiografias e até por angiotomografia (que, mesmo sendo um exame estático, pode mostrar trombos que por sua forma certamente são sésseis). Singh evidenciou trombos intraluminais na circulação cervicocerebral em 1,6% de 3.750 ATC desses vasos, com instabilidade neurológica^160^ . São mais prevalentes em homens e em portadores de alterações da coagulação, mas geralmente associados a placas de ateroma^161,162^ . A metanálise publicada por Fridman et al.^162^ apresenta resultados muitos ruins, com uma taxa de isquemias silenciosas, AIT, AVCI e morte de 17,1%. A literatura recomenda anticoagulação e antiagregação imediata após a visualização de trombos carotídeos flutuantes^160,161^ . Embora não exista uma padronização terapêutica definitiva, parece óbvio que os trombos carotídeos flutuantes devam ser eliminados o mais rapidamente possível, por uma abordagem direta por EC.

### Recomendação 4.1.3

Pacientes nos quais são evidenciados trombos carotídeos flutuantes por métodos de imagem, sintomáticos ou não, devem imediatamente receber anticoagulação com heparina e antiagregação com heparina e AAP e ser submetidos à intervenção em caráter de emergência, de preferência EC (IIb/C)^124,159-162^ .

### Intervenção após trombólise e trombectomia mecânica

A controvérsia sobre quando podemos intervir com segurança em uma lesão obstrutiva carotídea residual após trombólise venosa realizada para tratamento de um AVCI agudo permanece aberta em 2023. Cerca de 10-20% dos pacientes submetidos à lise nessas circunstâncias, têm lesões na carótida cervical que demandam tratamento^5,8,123,163-165^ . A indecisão entre operar precocemente e arriscar uma maior taxa de complicações se contrapõe à procrastinação em remover a lesão carotídea, muitas vezes a etiologia do AVCI. Embora a vida média do trombolítico disponível no Brasil atualmente – a alteplase, seja de 5 minutos, as alterações geradas pelos produtos de degradação da fibrina, entre outros agentes, levam a alterações muito mais duradouras^166^ . Uma das alterações que mais preocupa é o aumento da permeabilidade da barreira vascular, podendo levar à hemorragia intraparenquimatosa, gerando um AVCH^166,167^ . Anticoagulação e mesmo antiagregação plenas devem ser postergadas pelo menos até 24 horas após a lise e até que uma TC mostre ausência de hemorragia^167^ .

Indicações que convergem para uma intervenção mais precoce são: área cerebral isquêmica de menos de 1/3 do território da ACM; recanalização de uma ACM previamente ocluída evidenciada na ATC de controle; obstrução carotídea ipsilateral com estenose entre 50-99%; ausência de edema ou hemorragia intracerebral, e, sobretudo, regressão do grau de comprometimento neurológico para um grau de eRm de 0 a 2^163,165,168,169^ . Entre as contraindicações figuram os contrários das indicações, como: área cerebral isquêmica persistente de mais de 1/3 do território da ACM; hemorragia ou edema intracerebral; risco cirúrgico muito elevado; pescoço hostil; e déficit neurológico persistente (eRm ≥ 3)^7,170-172^ . Todos trabalhos citados são categóricos sobre a necessidade de um controle rigoroso dos níveis de HAS.

Já o lapso de tempo entre a lise e uma efetiva eliminação de fonte da isquemia cerebral permanece em debate. Embora existam publicações sobre EC precoce até em menos de 12 horas depois do final da lise com excelente resultado^170^ , a maioria dos trabalhos publicados mostra que intervenções mais precoces são seguidas de uma elevada incidência de síndrome de hiperperfusão cerebral (SHC), edema parenquimatoso e hemorragia intracerebral^165,169,171^ . A maior parte das grandes diretrizes sobre isquemia cerebral recentemente publicadas são omissas sobre qual deve ser o tempo ideal de intervir nas carótidas após lise sistêmica^8,63,123,154,158^ . Todavia vários trabalhos atuais de valor sustentam que um retardo de 6 dias deve ser mantido antes de intervir^5,163,165,169,171^ .

### Recomendação 4.1.4.1

Pacientes submetidos a trombólise para tratamento de isquemia cerebral aguda devem ser submetidos a um rigoroso controle de PA durante todo o procedimento e após ele, visando reduzir o risco de hemorragia intracerebral (I/C)^172^ .

### Recomendação 4.1.4.2

Em pacientes submetidos a trombólise para tratamento de isquemia cerebral aguda, a revascularização carotídea definitiva deve ser realizada pelo menos 6 dias após a trombólise (I/C)^163,164,170^ .

### Recomendação 4.1.4.3

Em pacientes submetidos a trombólise para tratamento de isquemia cerebral aguda, a terapia antitrombótica adicional com heparina e/ou AAP deve ser postergada por pelo menos 24 horas após o final da infusão do trombolítico e, então, mantida até que a revascularização carotídea definitiva seja realizada (IIa/C)^164,165,168,169,171^ .

### Teias carotídeas – *carotid webs* – como etiologia de AVCI

A presença de um defeito evidenciado como uma teia ou malha, geralmente localizado na parede posterior do bulbo carotídeo, tem sido demonstrada sobretudo recentemente em exames de imagem, o que certamente também está relacionado com maior precisão na resolução espacial dos estudos de eDc e ATC contemporâneos^173,174^ .

Conhecidas pelo termo inglês *carotid web*, essas teias carotídeas geralmente são evidenciadas na ausência de uma etiologia mais óbvia de isquemia cerebral, gerando uma busca ativa de causas raras, tendo sido identificadas em 0,9% dos casos de AVCI no estudo Mr CLEAN RCT^173^ . Infelizmente, essa qualidade de imagens não está disponível em todos os hospitais. Presentes sobretudo em mulheres com idade média de 46 anos, postula-se que tais defeitos da íntima arterial sejam uma variante da displasia fibromuscular, criando nichos nos quais haveria formação de trombos plaquetários ou hemáticos, que embolizariam em direção ao encéfalo^175-177^ . Pacientes jovens com AITs ou AVCIs criptogênicos devem ser submetidos a uma busca ativa de teias carotídeas pelos modernos métodos de imagem^175,178,179^ . Antiagregação não tem sido relatada como efetiva na prevenção da recorrência de isquemia, sendo recomendada intervenção^174,180^ . Há relatos de sucesso tanto com tratamento cirúrgico direto, com ressecção segmentar da área afetada e anastomose terminoterminal da carótida, como com implante de *stents* ^174,178,180,181^ .

### Recomendação 4.1.5

Em pacientes sintomáticos nos quais é detectada uma teia carotídea ipsilateral ao hemisfério cerebral sintomático e, após investigação minuciosa, é constatada a ausência de outras etiologias mais óbvias, uma ressecção do segmento comprometido com anastomose terminoterminal ou um implante de *stent* pode ser indicado (IIb/C)^173-181^ .

### Tratamento de pacientes com isquemia cerebral aguda de origem carotídea, com alto risco para EC

A maioria dos trabalhos publicados preconiza a EC como o método terapêutico mais eficaz no tratamento da isquemia cerebral com origem na carótida cervical^5,63,124,128-136,138,139,146^ . Todavia, há situações nas quais uma EC é um procedimento de alto risco. A estratificação de pacientes com alto risco cirúrgico para EC (ARC-EC) é muito variável. Yadav et al.^182^ consideraram ARC-EC nos indivíduos assintomáticos com estenoses carotídeas de 70-99%, com um ou mais dos fatores a seguir associados: doença cardíaca grave (insuficiência cardíaca congestiva [ICC], teste de esforço anormal, pré-operatório de revascularização coronariana); doença pulmonar obstrutiva crônica grave (DPOC); oclusão carotídea contralateral; paralisia do nervo recorrente contralateral; irradiação cervical prévia; dissecção radical de pescoço prévia; traqueostomia; restenose pós EC; e idade superior a 80 anos. Os eventos adversos maiores (EAM) de 5,8% para ACS e de 6,1% para EC não justificaram a intervenção em nenhum desses grupos de indivíduos assintomáticos. Vários desses fatores não se sustentam como de alto risco para EC, como por exemplo DPOC (pode-se realizar a cirurgia sob bloqueio cervical), oclusão contralateral (o cérebro pode ser protegido por uma derivação interna temporária) e a idade avançada. Esta, na verdade, comprovou ser uma das indicações de EC no idoso, conforme demonstrado no estudo CREST^183^ . As demais realmente configuram alto risco para EC^103,111,184,185^ . Nesses casos específicos, uma ACS deve ser considerada^5,103,111,124,182-185^ .

As indicações dos dois tipos de tratamento são discutidas nos capítulos específicos sobre indicações de tratamento desta Diretriz.

### Recomendação 4.1.6

Em pacientes sintomáticos com estenose da carótida entre 50-99%, com alto risco para EC comprovado, uma angioplastia com implante de *stent* deve ser considerada (IIb/B)^104-112-184-186^.

### Manejo do paciente com isquemia subaguda (após duas semanas) e crônica

A abordagem do paciente com isquemia cerebral aguda tem sido amplamente estudada e diretrizes de manejo do paciente com AIT/AVCI agudo estão disponíveis na literatura e são discutidas nestas Diretrizes. No entanto, a abordagem dos pacientes com sintomas há mais de 2 semanas permanece controvertida. Estudos mostram que o risco de novo evento isquêmico é mais alto na fase aguda, até 2 semanas depois do evento inicial^124^ . O risco elevado persiste depois do período agudo, dependendo da causa inicial da isquemia, dos fatores de risco controláveis (por exemplo, HAS) e do tratamento oferecido ao paciente.

Strömberg et al.^140^ , avaliando uma série de pacientes com isquemia cerebral em tempos variados após o início dos sintomas, encontraram um risco aumentado de isquemia recorrente até 3 meses depois do evento inicial. O achado foi confirmado por outros investigadores^142,186-193^ .

### Avaliação inicial

Os pacientes que são atendidos após 14 dias do início dos sintomas estão na fase de risco decrescente de isquemia recorrente. Neste momento, a avaliação clínica do paciente consiste em:

1 – Exame clínico geral e neurológico detalhado:

Avaliação da possível causa do evento isquêmico além da doença carotídea, como arritmia cardíaca e HAS; avaliação das sequelas neurológicas e classificação do paciente em uma das escalas de déficit neurológico, como a eRm^194^ . As Diretrizes europeias recomendam que o paciente seja avaliado por uma equipe multidisciplinar, composta por clínicos, neurologistas, radiologistas, cirurgiões vasculares e intervencionistas^103^ .

2 – Exames de imagem das carótidas e do encéfalo:

O paciente deve ser submetido a exame de eDc das carótidas e a ATC do arco aórtico às artérias intracranianas^195^ . ARM com contraste é mais sensível do que a ATC para avaliação de lesões isquêmicas do encéfalo^196^ .

3 – Avaliação do risco anestésico-cirúrgico, tendo em vista a possibilidade de tratamento invasivo da lesão carotídea^194^ .

### Tratamento clínico

Todo paciente com isquemia cerebral recente deve ser submetido ao TCO, que consiste no controle dos fatores de risco por mudanças no estilo de vida e o uso de medicações para prevenção secundária de novos eventos isquêmicos^197^ . Os fatores de risco controláveis são: HAS, tabagismo, dislipidemias, DM, sedentarismo e obesidade.

Dos fatores de risco, o controle rigoroso da HAS é o que tem maior impacto na prevenção de novos eventos em pacientes que tiveram isquemia cerebral recente^37,71^ .

HAS com picos sistólicos pode provocar novo evento isquêmico, por embolização a partir da placa, infarto de pequenos vasos intracranianos (lacunares) e hemorragia na área enfartada ou de penumbra^198^ . O tabagismo também deve ser eliminado^197^ . O controle dos fatores de risco e a prevenção secundária por meio de medicamentos foram discutidos em tópicos específicos destas Diretrizes.

### Recomendação 4.2

Pacientes com isquemia cerebral recente devem receber TCO, com controle rigoroso da PA e do DM, cessação do tabagismo e administração de estatinas e AAP (I/A)^37,71,197,198^ .

### Tratamento invasivo

O tratamento invasivo – EC ou ACS – deve ser seletivo, de acordo com o quadro neurológico do paciente, suas comorbidades e a disponibilidade de equipe cirúrgica e intervencionista experientes.

### Seleção dos pacientes para tratamento invasivo

O quadro neurológico do paciente deve ser avaliado, de preferência, por uma equipe multidisciplinar. A forma mais usada de avaliação das sequelas de evento isquêmico recente é a eRm^7^ . Pacientes com exame neurológico normal ou com sequelas menores, que permitem autonomia nas atividades diárias, são candidatos ao tratamento invasivo^139^ . Pacientes com sequelas neurológicas severas (eRm 4 e 5) não são candidatos ao tratamento invasivo e devem ser mantidos em TCO perene^145^ .

Pacientes na eRm 3 – com sequelas moderadamente graves, mas que conservam algum grau de autonomia – devem ser avaliados individualmente. Pacientes com lesão grave ipsilateral (estenose > 70% ou placa complexa com alto risco de evento intraplaca, com risco de novo evento cerebral) e/ou lesão grave ou oclusão da carótida contralateral devem ser considerados para EC, mesmo que não haja expectativa de recuperação das sequelas já existentes.

### Recomendação 4.3

Pacientes com sequelas neurológicas severas (eRm 4 e 5) não são candidatos a tratamento invasivo e devem ser mantidos em TCO perene (IIa/C)^139,145^ .

### Endarterectomia da carótida (EC)

O tratamento cirúrgico – endarterectomia da carótida – das lesões ateroscleróticas da carótida extracraniana foi introduzido na década de 1950 e refinado ao longo das décadas seguintes^199^ .

### Indicações para endarterectomia da carótida

A partir dos anos 1990, os benefícios da EC nas estenoses graves sintomáticas recentes da carótida extracraniana foram demonstrados por três grandes estudos multicêntricos: o NASCET, o ECST e o VA Carotid Trial^200-202^ . Os resultados desses estudos, somados aos outros estudos comparando EC e o tratamento clínico, foram analisados pelo Carotid Trialists Group numa série de metanálises^203-205^ . Essas análises levaram as diretrizes internacionais à recomendação grau A de se realizar a EC em pacientes portadores de estenose > 50% com eventos isquêmicos cerebrais (AIT e AVCI) nos últimos 6 meses^5,9,23,63,68,198,206-210^ .

Os benefícios da EC na prevenção de eventos isquêmicos são avaliados frente ao risco anestésico-cirúrgico de se realizar EC mesmo em pacientes idosos, com várias comorbidades. Metanálises de estudos e séries publicadas concluíram que a EC só tem benefício estatisticamente significativo quando as taxas de complicações graves (AVC, IAM e óbito) forem menores do que 6%^203-205^ .

### Recomendação 4.4

Nos pacientes com estenose > 50%, com sintomas nos 6 meses precedentes, é recomendada a EC ipsilateral, desde que o risco de AVC/óbito perioperatório seja < 6% (I/A)^5,9,23,63,68,198,203-210^ .

### Redução de risco com a EC em subgrupos

Os candidatos à EC formam uma população heterogênea. Os benefícios da EC em reduzir o risco de novos eventos isquêmicos não são os mesmos para todos os pacientes. Existem subgrupos de pacientes nos quais o benefício é maior, em função de variáveis clínicas e de imagem.

Nas análises de subgrupos de pacientes randomizados para os estudos NASCET e ECST foram identificadas as variáveis apresentadas no Quadro 8, que influenciam a RR de novos eventos isquêmicos e, consequentemente, o benefício da EC. O benefício é medido pelo número de pacientes que devem ser tratados para beneficiar um paciente (NNT). Quanto menor o NNT, maior o benefício. A RR deve ser levada em conta na decisão de se indicar a EC no paciente sintomático. A decisão deve ser sempre individualizada, pesando os benefícios da EC *versus* o TCO^203-205^ .

**Quadro 8:** Variáveis que influenciam a redução de risco de novos eventos isquêmicos.

**VARIÁVEL**	**RR % (NNT)**
Sexo	H: 11%^118^ M: 2,8%^201^
Idade	< 65 anos: 5,6%^187^ 65-75 anos: 8,6%^124^ > 75 anos: 19,2%^116^
Número de comorbidades (redução de risco em 2 anos)	EC – 0-5:17%; 6: 23%; 7 ou +: 39% TCO – 0-5: 11%; 6: 6%; 7 ou +: 8%
Tipo de AVC: cortical *versus* lacunar	cortical: 15%^211^ lacunar: 9%^121^
Aparência da placa	lisa: 8%^140^ irregular: 17%^117^
Sintomas hemisféricos *versus* oculares	oculares: 5%^191^ AIT: 15%^211^ AVC: 18%^117^
Doença oclusiva intracraniana associada ao grau de estenose	50-69%: 19%^116^ 70-85%: 29%^114^
Oclusão da carótida contralateral	> 85%: 45%^113^ oclusão: 24%^115^ sem oclusão: 13%^119^

RR: redução de risco; NNT: número de pacientes necessários que tem que ser tratados para beneficiar um paciente; EC: endarterectomia da carótida; TCO: tratamento clínico otimizado; AIT: ataque isquêmico transitório; AVC: acidente vascular cerebral.

### Recomendação 4.5

A decisão de se indicar a EC deve ser individualizada, levando em conta a redução de risco de eventos isquêmicos futuros (I/B)^203-205^ .

### Preparação do paciente para EC

EC é uma operação de médio porte realizada eletivamente ou em caráter de urgência muitas vezes em pacientes idosos, com múltiplas comorbidades. Os pacientes devem estar sob TCO. As classes de medicamentos que fazem parte do TCO são apresentadas a seguir:

Anti-hipertensivos: o controle estrito da PA antes e durante a EC reduz significativamente o risco de novos eventos isquêmicos a longo prazo^198,212,213^ . HAS persistente com níveis sistólicos > 180 mmHg está associada a risco aumentado de AVC perioperatório, SHC e óbito^213^ .

Estatinas: sabe-se que as estatinas, além de reduzir os níveis séricos de colesterol, têm efeitos pleiotrópicos sobre a parede arterial que resultam na estabilização da placa aterosclerótica^214-216^ . Esses efeitos protetores foram comprovados por estudos prospectivos randomizados^217-219^ . Uma metanálise de estudos randomizados mostrou redução significativa das complicações e mortalidade nos pacientes sob tratamento com estatinas no período perioperatório^220^ .

### Recomendação 4.6

Nos pacientes candidatos a tratamento invasivo, recomenda-se iniciar estatinas antes da operação e continuar indefinidamente (I/A)^220^ .

Antiagragantes Plaquetários (AAP): tradicionalmente, pacientes encaminhados para EC estão recebendo monoterapia antiplaquetária com AAS, em doses que variam de 81 a 325 mg^215^ . Existe consenso que os pacientes devem ser mantidos em terapia antiplaquetária com AAS no período perioperatório da EAC^221,222^ . A terapia antiplaquetária combinada, AAS + clopidogrel ou dipiridamol ou ticagrelor, nos pacientes candidatos a EC tem sido objeto de vários estudos randomizados, com resultados conflitantes^223-226^ . Os cirurgiões se preocupam com o sangramento intraoperatório e a formação de hematomas cervicais no pós-operatório imediato, mas estudos não mostraram aumento dessas complicações hemorrágicas^227,228^ . A decisão de acrescentar outros AAP ao AAS no período perioperatórios da EAC deve ficar a critério da equipe cirúrgica^215,225^ .

### Recomendação 4.7

Pacientes candidatos a tratamento invasivo de lesões sintomáticas da carótida extracraniana devem ser colocados em terapia antiplaquetária com AAS (IIa/A)^220,221^ .

### Recomendação 4.8

O acréscimo de outras drogas antiplaquetárias além do AAS no período perioperatório da EC deve ficar a critério da equipe cirúrgica (IIa/B)^225^ .

Anticoagulantes: pacientes candidatos a EC depois do período agudo (> 14 dias) que estão recebendo anticoagulantes devem ser devem ser manejados como em qualquer outra cirurgia arterial. Anticoagulantes orais diretos devem ser suspensos 48 horas antes da operação; as drogas antivitamina K devem ser suspensas até que o valor de razão normatizada internacional (RNI) seja < 1,8^229^ . Nos pacientes com alto risco de complicações tromboembólicas, pode-se fazer uma ponte até o dia da operação com heparina de baixo peso molecular (HBPM)^230^ .

Manejo do DM: não há estudos prospectivos sobre a importância do controle da glicemia nos pacientes diabéticos submetidos a EC. O bom senso clínico sugere que o paciente diabético deve estar com bom controle glicêmico no período perioperatório.

### Angioplastia da carótida com stent

Na década de 1990, foi introduzido um novo procedimento no tratamento de lesões ateroscleróticas da carótida extracraniana: a angioplastia, inicialmente sem e evolutivamente com a implantação de *stent*. Depois de experiências iniciais desencorajadoras, o procedimento foi aprimorado, com o uso rotineiro de *stents* autoexpansíveis e de filtros de proteção para evitar a embolização intraoperatória. Ao contrário da EC, que é realizada somente por cirurgiões vasculares (e alguns neurocirurgiões), a ACS é um procedimento realizado por vários especialistas: cirurgiões vasculares, radiologistas intervencionistas, neurointervencionistas, neurocirurgiões e cardiologistas intervencionistas. A consequência de tantas especialidades estarem envolvidas no tratamento endovascular (TE) das lesões de carótidas foi o desenvolvimento de uma literatura extensa e heterogênea^9^ . Diretrizes sobre ACS têm sido publicadas por diversas sociedades de especialidades, mas persistem divergências sobre as indicações, técnicas de proteção cerebral e manejo das complicações. Atualmente, a ACS tem um papel relevante, porém limitado, no tratamento dos pacientes com estenoses sintomáticas da carótida extracraniana.

### Indicações para ACS

O surgimento da ACS como uma técnica alternativa à EC levou a estudos prospectivos randomizados, comparando as duas técnicas^183,230-235^ . Embora vários estudos mais antigos demonstrassem o mesmo, uma metanálise recente, de 2019, com 20 estudos prospectivos randomizados, mostrou que a ACS resultou em taxas significativamente mais altas de AVC, AVC/óbito e AVC/IAM/óbito em 30 dias do que a EC^236^ . Análises de subgrupos desses estudos evidenciaram que os resultados inferiores da ACS ocorreram em mulheres, em pacientes > 70 anos, em procedimentos realizados nos primeiros 14 dias do evento isquêmico inicial, em pacientes com placas complexas e sequenciais, e em pacientes com lesões focais na substância branca detectadas na RM. Esses estudos permitem afirmar que a ACS não é o procedimento de primeira escolha na maioria dos pacientes sintomáticos candidatos a tratamento invasivo.

Por outro lado, existem pacientes que têm risco cirúrgico/anestésico elevado e/ou nos quais a EC é tecnicamente mais difícil ou até de risco proibitivo de ser realizada. Nessas situações de vida real, a ACS tem indicações aceitáveis como uma alternativa mais segura à EC, listadas no Quadro 9^111,184,236-239^ .

**Quadro 9:** Indicações para angioplastia da carótida com stent.

**INDICAÇÕES PARA ANGIOPLASTIA DA CARÓTIDA COM STENT**	
**Indicação**	**Referências**
Lesões na carótida interna extracraniana distal (C1 – C2)	AbuRahma^184^
Restenoses da carótida operada	Arhuidese et al.^237^
Pescoço irradiado	Fokkema et al.^238^
Deformidades da coluna cervical	Fokkema et al.^238^
Neoplasias, traqueostomia etc.	Fokkema et al.^238^
Pescoço submetido a esvaziamento cervical	Fokkema et al.^238^
Lesão no nervo laríngeo recorrente contralateral	Batchelder et al.^236^
Pacientes com risco anestésico elevado (DPOC, ICC etc.)	Gurm et al.^111^ , Fokkema et al.^238^
Oclusão da carótida contralateral	Kokkinidis et al.^239^

As indicações listadas na Recomendação 4.9 não foram corroboradas por estudos prospectivos randomizados. Dessa forma, as recomendações referentes às indicações da ACS são de Grau II, com Níveis de Evidência B ou C.

### Recomendação 4.9

A ACS pode ser considerada em pacientes com estenoses > 50% sintomáticas recentes em portadores de estenoses na carótida interna extracraniana distal, com restenoses pós-operatórias, pescoço hostil (história de irradiação ou esvaziamento cervical etc.), lesão do nervo laríngeo recorrente, alto risco anestésico/cirúrgico e oclusão da carótida contralateral, desde que o risco de AVC/óbito periprocedimento seja < 6% (IIb/B e C)^183,238,240,241^ .

A preferência do paciente deve ser respeitada, desde que não haja contraindicações para a realização de ACS.

### Angioplastia transcervical da carótida com stent

Nesta nova forma de ACS, a angioplastia transcervical da carótida com *stent* (em inglês, transCarotid artery revascularization [TCAR]), o acesso é feito diretamente através de uma bainha curta, colocada por acesso cirúrgico aberto na carótida comum proximal no pescoço. O dispositivo permite fluxo reverso da carótida interna através de uma bainha para reinfusão na veia femoral ou na jugular ipsilateral, por oclusão temporária da carótida comum proximal. A principal vantagem do TCAR é evitar a navegação dos fios-guia e catéteres desde a artéria femoral, evitando a passagem pelo arco aórtico, até atingir a carótida onde vai ser implantado o *stent*. Não há estudos prospectivos randomizados comparando o TCAR com o acesso femoral para ACS. A melhor informação disponível são os estudos ROADSTER 1 e 2, que mostraram a segurança e eficácia do dispositivo e uma análise pareada do TCAR com pacientes submetidos à ACS transfemoral^242-244^ . Os resultados mostram uma redução significativa das taxas de AVC/óbito nos pacientes tratados pelo método TCAR, quando comparadas à ACS transfemoral^245^ .

### Recomendação 4.10

ACS transcarotídea – TCAR é uma alternativa mais segura e efetiva do que a ACS transfemoral em candidatos a ACS (IIa/C)^242-245^ .

### Preparação do paciente para ACS

A avaliação clínica e a preparação do paciente para ACS são as mesmas do paciente para EC. A diferença é o esquema de anticoagulantes e AAP usados na ACS. Os estudos são conflitantes, mas os especialistas que realizam ACS preferem fazer uma dose de ataque de AAS (de 325 a 650 mg) ou de clopidogrel (150 a 300 mg) no dia anterior ao procedimento ou antes^185,246,247^ .

### Doença carotídea sintomática há mais de 6 meses

Conceitualmente, pacientes cujo último evento isquêmico ocorreu há mais de 6 meses são considerados assintomáticos. Estudos de seguimento de longo prazo mostram que o risco de um evento isquêmico nessa população é baixo. Esses pacientes devem permanecer em TCO perene. O risco destes pacientes sob TCO contemporâneo é < 1% ao ano^247^ . Sob o TCO, uma minoria dos pacientes com lesões assintomáticas da carótida vai desenvolver isquemia cerebral. A missão dos médicos envolvidos em cuidar de pacientes com estenose assintomática da carótida é identificar quais pacientes têm risco mais elevado de desenvolver eventos isquêmicos cerebrais^248^ . O manejo dos pacientes com doença carotídea sintomática há mais de 6 meses é discutido em “Doença carotídea assintomática”.

## TÉCNICA CIRÚRGICA ABERTA

A AHA define a EC como um procedimento de risco intermediário^249^ . A determinação do risco de eventos adversos cardíacos maiores permite determinar a avaliação e conduta pré-operatórias necessárias para cada paciente individualmente. O risco anatômico para a realização do tratamento cirúrgico da carótida também deve ser determinado com o objetivo de selecionar entre as técnicas cirúrgicas aberta ou endovascular, aquela que oferecerá menor risco de complicações a cada paciente. São definidores de elevado risco anatômico carotídeo para cirurgia aberta a presença de restenose após EC, cirurgia cervical radical prévia, radioterapia cervical, lesão contralateral do nervo laríngeo recorrente e traqueostomia^250^ .

A maioria dos pacientes portadores de lesão carotídea bilateral apresenta lesões assintomáticas ou apenas um lado sintomático, sendo rara a apresentação de sintomas bilateralmente. A realização de EC bilateral simultânea apresenta risco de complicações graves, como a lesão bilateral dos nervos laríngeo recorrente ou hipoglosso. Assim, deve-se realizar preferencialmente, em caso de lesões bilaterais, a EC de forma estagiada. Caso seja extremamente necessária a realização de cirurgia simultânea, deve-se optar por angioplastia carotídea bilateral ou EC unilateral e angioplastia contralateral^251-253^ .

A EC pode ser realizada sob anestesia geral ou anestesia locorregional (ALR), havendo controvérsias quando comparamos os resultados. A ALR apresenta como benefícios possíveis a diminuição resposta ao estresse cirúrgico, a monitorização da perfusão cerebral pela consciência durante o procedimento e a menor taxa do uso de *shunts*. No entanto, apresenta como desvantagens a possível ansiedade do paciente durante o procedimento, a necessidade de sedação profunda em alguns períodos em caso de desconforto e a necessidade de conversão para anestesia geral durante o procedimento, em raros casos na maioria dos estudos. O estudo GALA (General anesthesia versus local anesthesia for carotid surgery trial) foi o maior estudo randomizado realizado comparando as técnicas anestésicas, incluindo 3.526 pacientes, não sendo observada diferença significativa nas taxas de AVC, IAM ou óbito entre os grupos^254^ . A decisão é dependente da experiência e escolha da equipe responsável pelo procedimento.

A incisão pode ser realizada de forma longitudinal, paralela à borda anterior do músculo esternocleidomastóideo ou transversal, não sendo identificada diferença na incidência de lesão de nervos cranianos^255^ . O eco-Doppler pode ser empregando para a marcação da bifurcação carotídea, permitindo a realização de incisões menores, sendo essas associadas a menor incidência de lesões de nervos cranianos^256,257^ .

O uso de derivações internas temporárias na EC, conhecidas como *shunts,* pode ser realizado em caráter de rotina ou seletivo. O clampeamento carotídeo pode promover um AVC hemodinâmico, prevenido pelo emprego do *shunt*. Durante o clampeamento, a perfusão cerebral pode ser medida por eletroencefalografia, pressão retrógrada carotídea, Doppler transcraniano, oximetria cerebral transcraniana ou espectroscopia de infravermelho. No entanto, o método mais confiável é a avaliação do nível de consciência sob ALR. Cirurgiões que fazem uso de *shunt* em caráter de rotina apresentam taxa de complicações associada ao seu uso menores que quando comparada a cirurgiões que utilizam *shunt* seletivamente^258^ . Estudo de metanálise envolvendo 3.856 pacientes evidenciou taxa média de AVC peroperatório em pacientes submetidos a *shunt* rotineiro de 1,4%, e para o não uso de *shunt* de 2%.

Em pacientes submetidos ao uso seletivo de *shunt*, a taxa variou dependendo do método de monitorização empregado: 1,1% com ALR, 1,6% com eletroencefalograma e pressão carotídea retrógrada, e 4,6% para Doppler transcraniano. O *shunt* pode ser empregado em caráter rotineiro ou seletivo, dependendo da opção do cirurgião^259,260^ .

### Recomendação 5.1

Pacientes submetidos a EC podem ser submetidos a uso de *shunt* em caráter rotineiro ou seletivo (IIa/C)^258,260^ .

Podem ser utilizados como remendo (em inglês, *patch*) na EC convencional diversos tipos de materiais, como veia autógena, PTFE, poliéster e pericárdio bovino. Em metanálise de estudos randomizados com 4.400 pacientes estudados para sete diferentes tipos de tratamento (síntese primária, n = 753; endarterectomia por eversão, n = 431; *patch* de veia, n = 973; *patch* de PTFE, n = 948; *patch* de poliéster, n = 828; *patch* de pericárdio bovino, n = 249, e *patch* de poliuretano = 258), a EC por eversão e a EC convencional com *patch* de PTFE e pericárdio apresentaram as menores incidências de AVC e óbito. A menor incidência de restenose foi evidenciada nos grupos de EC por eversão, *patch* com pericárdio bovino e PTFE, enquanto a maior incidência foi identificada no grupo de síntese primária e *patch* de poliéster^261^ . Uma metanálise de 10 estudos randomizados, envolvendo 2.157 pacientes, demonstrou que o emprego do *patch* diminui o risco de AVC perioperatório e AVC tardio, além da redução do risco de oclusão arterial perioperatória e restenose na EC longitudinal convencional^262^ .

### Recomendação 5.2

O uso rotineiro de *patch* é recomendado em pacientes submetidos a EC convencional (I/A)^261,262^ .

A EC por eversão é realizada com a secção oblíqua da ACI do bulbo carotídeo. Apresenta como vantagens evitar o implante de material sintético, ser mais rápida quando comparada à endarterectomia longitudinal e possibilitar a retificação da ACI, se redundante. Apresenta como desvantagens a maior dificuldade para utilização de *shunt* e o acesso distal à ACI. O estudo randomizado EVEREST comparou as técnicas de endarterectomia por eversão e convencional em 1.353 pacientes, não identificando diferença significativa na incidência de AVC, IAM e óbito entre as duas técnicas. A incidência de restenose foi menor para o grupo submetido à endarterectomia por eversão^259^ . Uma metanálise de 25 estudos (cinco randomizados e 20 observacionais) avaliando 49.500 pacientes, comparou 16.249 pacientes submetidos à endarterectomia por eversão com 33.251 pacientes submetidos à EC por técnica convencional. Os dados dos estudos randomizados não demonstraram diferença significativa nas incidências de AVC, óbito, IAM e hematoma cervical em 30 dias entre os dois grupos. No entanto, foi observada menor incidência de restenose no grupo de endarterectomia por eversão (p = .001). Os dados dos estudos observacionais demonstraram, para o grupo de eversão, redução significativa na incidência de óbito em 30 dias (p < .001), AVC (p < .001), óbito/AVC (p < .001) e restenose (p = .032)^263^ .

### Recomendação 5.3

A opção entre EC por eversão ou EC convencional com *patch* deve ficar a critério do cirurgião (IIa/A)^263^ .

Os pacientes que apresentam alongamentos, acotovelamentos e laços carotídeos, conhecidas pelos termos em inglês *coils, kinks* e *loops,* apenas devem ser submetidos a tratamento cirúrgico em casos sintomáticos, nos quais uma clara relação entre a anomalia e a sintomatologia possa ser estabelecida. Um estudo analisou 92 pacientes randomizados para tratamento cirúrgico ou melhor tratamento clínico, submetidos a acompanhamento por tempo médio de 5,9 anos: no grupo submetido a tratamento cínico, foi observada incidência de trombose carotídea de 5,5% e de AVC de 6,6%, não havendo incidência no grupo cirúrgico^264^ .

A bifurcação carotídea alta promove desafios técnicos que elevam os riscos de AVC e lesão de nervos cranianos no perioperatório. Idealmente, essa situação deve ser identificada nos exames pré-operatórios. A ATC facilita essa identificação pelo fato de as estruturas ósseas estarem incluídas no exame. Diversas manobras podem ser empregadas para permitir o acesso à carótida interna distal à linha de Blaisdell: intubação nasotraqueal (promove a abertura do ângulo entre o processo mastóideo e a mandíbula), ligadura de ramos da artéria carótida externa e secção do músculo digástrico. A subluxação da mandíbula deve ser planejada no pré-operatório, com intubação nasotraqueal e a utilização de fios metálicos interdentários entre os dentes pré-molar inferior e canino superior. Trançando os dois fios, desloca-se a mandíbula anteriormente cerca de 1 cm^265^ .

Pontes carotídeas podem ser indicadas para o tratamento de infecção local, contaminação de patches, retirada de *stent* carotídeo ou problemas técnicos decorrentes de lesão da parede arterial durante a EC. Como condutos para a realização de pontes podem ser empregados a veia safena magna devalvulada, PTFE ou dácron^241,266,267^ . Obviamente, o uso de material sintético é contraindicado na presença de infecção.

## TÉCNICA CIRÚRGICA ENDOVASCULAR

Como já descrito em seções anteriores desta Diretriz, a prevalência da doença carotídea e suas graves complicações, sobretudo AITs e AVCIs, exigem atenção de toda sociedade médica^268^ . Uma profilaxia adequada e indicação de tratamento precoce pode prevenir a progressão da placa ou a evolução para um desfecho desfavorável ao paciente^269^ .

### Introdução e histórico

A angioplastia carotídea foi descrita por Mathias em 1981 e, apesar de resultados iniciais favoráveis, foi associada a várias complicações, em especial o deslocamento de fragmentos da placa com embolização cerebral, dissecções e restenoses. Seguindo as intervenções coronarianas, o uso de *stent* somou-se às angioplastias simples com balão, e desde 1994 passaram a integrar o tratamento endovascular (TE) da doença carotídea^270^ . Grande progresso se obteve em relação aos dispositivos em geral, cateteres, fios, dispositivos de proteção antiembólica cerebral (DPACs), balões, *stents* assim como o aperfeiçoamento técnico das equipes envolvidas. Entre esses, destacamos os DPACs, introduzidos por Theron, e o desenvolvimento de *stents* de dupla camada^271^ .

### Preparo para angioplastia carotídea

Historicamente, a maioria dos procedimentos de ACS foram realizadas com interrupção da anticoagulação, no pré-operatório. Em 2019, várias diretrizes societárias orientaram não interromper o uso de anticoagulantes, a menos que haja risco elevado de sangramento^272^ . A AHA recomendou uso diário de AAS como profilaxia cardiovascular em pacientes com morbidade cardíaca, baseada em uma redução de morbidade e morte com a terapia de antiagregação plaquetária^273^ . Evidências de tratamento antitrombótico para prevenção secundária de AVC recorrente em pacientes sintomáticos com aterosclerose carotídea são ainda mais robustas.^105^

O benefício da associação do AAS com clopidogrel no paciente submetido a implante de *stent* carotídeo é clara, reduzindo as complicações neurológicas. A duração ideal da dupla antiagregação por pelo menos 4 semanas é extrapolada do protocolo do CREST^274^ . Na decisão entre dupla ou monoterapia deve-se considerar o risco de hemorragia intracraniana ou sistêmica (hemorragia maior: 3,4% *versus* 1,5%, respectivamente)^275^ .

Particularmente nos pacientes com AVCI com FA em uso de anticoagulação oral (ACO), não se observou diferença significativa na taxa de AVC ou oclusão intra*stent* comparando o uso da dupla ou monoantiagregação com o ACO (3% *versus* 5% (p = 0,72) e 2 *versus* 0% (p = 0.20), deixando dúvidas sobre qual combinação antitrombótica é ideal em pacientes com FA tratados por ACS^276^ . Em um estudo de coorte retrospectivo com 91 pacientes observou-se aumento da taxa de sangramento de 23,8% no 1º mês com terapia tripla (dupla ação antiagregante + ACO) em comparação com dupla antiagregação (4%) ou ACO com um antiagregante (0%).

Paralelamente, não houve eventos tromboembólicos no grupo de terapia tripla em comparação com um evento cada dos outros grupos^277^ . Aos 90 dias de seguimento, a taxa de bom resultado funcional foi semelhante para dupla antiagregação em comparação com ACO + antiagregante (36% *versus* 42%, p = 0,77), mas a mortalidade tardia foi significativamente maior no grupo sem anticoagulante (32% *versus* 0,4%, p = 0,020). Isso foi confirmado por regressão logística ajustada para idade (p = 0,021)^278^ .

Nos pacientes a serem submetidos a ACS, a terapia antiplaquetária com AAS (75-325 mg diariamente – geralmente usa-se 100 mg/dia) combinada com clopidogrel (75 mg ao dia) é indicada. Recomenda-se que essa última deva ser iniciada pelo menos 3 dias antes do implante de *stent,* ou, em casos urgentes, sem a possibilidade de espera, como uma dose de ataque de 300 mg, seguida da dose de manutenção, que deve ser continuada indefinidamente^105,279-281^ .

A monoterapia com clopidogrel após a dupla antiagregação por 1 mês pós procedimento, em comparação ao uso de AAS + clopidogrel por 12 meses, teve um benefício na redução de eventos hemorrágicos maiores, sem estar associada ao aumento de eventos cardiovasculares em *stent* coronarianos^282^ . Pacientes que necessitam de monoterapia antiplaquetária indefinida após intervenção coronária percutânea apresentaram menores taxas de eventos clínicos adversos (AVC/IAM) com clopidogrel do que com AAS^220,277,278,283^ .

Outro fármaco bastante estudado é a estatina. Em metanálise com seis estudos observacionais (n = 7.503), pacientes que tomavam estatinas antes de EC tiveram menor mortalidade perioperatória (0,2% *versus* 1,3%) comparado com os que não faziam uso (OR 0,26; IC95% 0,1-0,61), e uma redução de AVC perioperatório, apesar de não significativo, de 1,4% *versus* 3,0% (OR 0,4; 95%CI 0,15-1,09)^283^ . Em outros 11 estudos observacionais (n = 4.088), obtiveram a manutenção dos mesmos benefícios do uso de estatinas em relação à mortalidade (OR 0,30; IC95% 0,10-0,96) e de AVC periprocedimento (OR 0,39; 95% CI 0,27-0,58)^284^ . O tratamento com estatinas na pré-intervenção carotídea vem diminuindo as complicações peri e pós-operatórias, mostrando-se promissor na redução dos eventos isquêmicos tanto na EC quanto na ACS^213,285^ .

O controle pressórico é outro fator contribuinte para melhores resultados, mas com cautela em não reduzir a PA de forma demasiada, comprometendo o fluxo de perfusão cerebral. Uma PA sistólica acima de 180 mmHg é um fator de risco independente para AVC após EC (não há dados publicados referente à ACS)^213^ .

### Recomendação 6.1.1

Recomenda-se o uso de terapia com estatina no pré-operatório de ACS (I/A)^213,284,285^ .

### Recomendação 6.1.2

Recomenda-se o uso de terapia com AAS (75-325 mg) diariamente no pré-operatório de ACS (I/C)^105,275^ .

### Recomendação 6.1.3

Recomenda-se que o uso de clopidogrel (75 mg/dia) seja iniciado pelo menos 3 dias antes do procedimento de ACS; caso isso não seja possível, recomenda-se a administração de uma dose única de ataque 300 mg (I/C)^220,274,275,277,278,282,283^ .

### Recomendação 6.1.4

Recomenda-se a continuação da terapia com AAS associado a clopidogrel por pelo menos 4 semanas após o implante de *stent* e a continuação da monoterapia antiplaquetária por tempo indefinido (preferencialmente clopidogrel 75 mg)^282^ .

### Recomendação 6.1.5

Pacientes em uso de anticoagulante oral que por outro motivo não necessitam suspender o uso para realização do acesso percutâneo e devem prosseguir o pós-operatório com ACO + monoterapia antiagregante. Painel de especialistas^276-278^ .

### Recomendação 6.1.6

Recomenda-se o controle rigoroso da PA. Hipertensão (> 180/90 mmHg) e hipotensão (< 100 mmHg sistólica) devem ser tratadas farmacologicamente (IIa/C)^212^ .

### Pré- ou pós-dilatação na angioplastia carotídea

Ainda existem controvérsias entre as vantagens de pré-dilatar as lesões carotídeas ou pós dilatá-las após o implante do *stent*. Evitar a pós-dilatação funcionou como proteção contra a depressão hemodinâmica persistente em uma metanálise de seis estudos de coorte envolvendo 4.652 pacientes (RR 0,59; IC95% 0,39-0,87, p = 0,03). O impacto da instabilidade hemodinâmica na evolução clínica dos pacientes permanece obscuro. Seus resultados não mostraram um aumento estatisticamente significativo nas taxas de morte, AVC, AIT ou IAM entre esses grupos de pacientes. Vale ressaltar que não foi diferenciado o tipo de células de *stent* (fechada ou aberta) no estudo^286,287^ .

Quando em um estudo retrospectivo foram comparados dois grupos, um com pré-dilatação subótima (com um balão de 3,0-4,0 mm a 8-10 atm), seguido de pós-dilatação (com um balão de 5,0-5,5 mm a 8-10 atm) após a liberação do *stent* (n = 130), e outro, no qual um *stent* foi implantado após a dilatação ideal (com um balão de 4,0-5,0 mm a 10-14 atm) e não foi submetido à pós-dilatação do balão (n = 237), observou-se microembolia ipsilateral assintomática periprocedimento em DW-MRI craniana em 25 (10,5%) pacientes no grupo ACS com uma dilatação e 24 (18,5%) no grupo com pré- e pós-dilatação. A diferença entre os dois grupos foi estatisticamente significativa (p = 0,033). Importante ressaltar que foram utilizadas marcas diferentes de *stents*, tanto de célula aberta quanto fechada, e vários sistemas de proteção proximal e filtros, não sendo esses aspectos avaliados no estudo^288,289^ .

### Recomendação 6.2

O momento da angioplastia carotídea deve ser definido pelo cirurgião, levando em consideração a anatomia da lesão carotídea, o grau da estenose e a escolha do *stent.* Painel de especialistas^286,288,289^ .

### Recomendação 6.3

Durante a ACS, recomenda-se evitar balonamento de repetição. Painel de especialistas^286-288,290^ .

Em uma auditoria retrospectiva da SVS-VQI, um total de 10.074 pacientes foram identificados, incluindo 688 pacientes tratados com *stent* primário (6,8%) e 9.386 pacientes tratados com pré-dilatação (93,2%). O AVC perioperatório (30 dias/morte) ocorreu em 3,5% dos casos (n = 353), incluindo AVC em 2,4% (n = 237) e morte em 1,5% (n = 152)^289^ . Dispositivos de proteção cerebral foram usados em 76,6% dos pacientes com *stent* primário, em comparação com 96,7% do grupo de pré-dilatação (p < 0,001). Na análise de regressão logística multivariável, ajustando as diferenças basais entre os grupos, o grupo de *stent* primário não apresentou resultado (AVC e óbito) significativamente diferente do grupo com angioplastia pré-*stent* (OR, 1,15; 95%CI 0,72-1,83)^288-290^ .

### Proteção cerebral antiembólica

O uso de proteção para a prevenção de embolização cerebral mostrou-se extremamente importante na redução de eventos cerebrais isquêmicos durante a ACS em diversas publicações, contudo não em todas. Apesar de material embólico ser regularmente retirado de filtros, o Carotid Stenosis Trialists Collaboration, uma metanálise de três RCT com n = 1.557, relatou que os dispositivos de proteção cerebral antiembólico (DPCA) não foram capazes de reduzir o acidente vascular cerebral/morte em 30 dias (RR 1,1; IC95% 0,71-1,70, p = 0,67)^290^ . Uma metanálise de 13 RTCs e de 193 registros (n = 54.713) e de 22 estudos (n = 11.655) relatou menor prevalência de AVC/óbito perioperatório, favorecendo os DPCAs (OR 0,57; IC95% 0,43-0,76, p < .01)^291,292^ . O registro nacional alemão (n = 13.086) corroborou esses achados, com taxas mais baixas de AVC grave/morte (RR 0,60; 95%CI 0,43-0,84) ou qualquer AVC (RR 0,57; 95%CI 0,43-0,77) com o uso de proteção^293^ . O estudo da auditoria do SVS-VQI, já citado anteriormente (n = 10.074), mostrou em análise de regressão logística multivariável, ajustando as diferenças basais entre os grupos, uma maior taxa AVC/morte em 30 dias quando os DPCAs não foram usados (OR 3,97; 95%CI 2,47-6,37)^287^ . Existem atualmente dois conceitos de DPCA disponíveis no Brasil: os filtros distais e os de oclusão proximal^290^ . Os sistemas de reversão de fluxo foram descontinuados ou ainda não são comercializados entre nós, e, portanto, serão analisados superficialmente.

### Dispositivos de filtro distal

Sem dúvida os filtros são os dispositivos de proteção cerebral mais utilizados no TE da doença carotídea. Diferenças na construção dos filtros, tipo e diâmetro de malha, bem como desenho e ancoragem, criam performances distintas na proteção de embolização cerebral^290-295^ . Após a publicação das recomendações do *Centers for Medicare and Medicaid Services* e da AHA/American Stroke Association, o uso de DPCAs durante ACS tornou-se o padrão de atendimento. Essas Diretrizes são baseadas em dados que demonstram que DPCAs distais reduzem o risco de AVC ou morte em 30 dias após CAS em até 67%, e reduzem em até 27% a incidência de novas lesões cerebrais ipsilaterais na RM^294,295^ . Os estudos avaliados não são ensaios controlados ou randomizados, e, portanto, não possuem força de evidência científica, permanecendo o consenso de especialistas de que alguma forma de proteção antiembólica deve ser usada durante a ACS.

### Dispositivos de oclusão proximal

O acesso e a forma de proteção cerebral funcionam de maneira a alterar ou mitigar alguns dos riscos potenciais da ACS, especialmente em pacientes sintomáticos e octogenários, reduzindo a embolização cerebral perioperatória. Em uma metanálise com dois diferentes dispositivos de oclusão proximal com 2.397 pacientes de seis bancos de dados, avaliando eventos adversos maiores (EAM) em 30 dias, incluindo AVCI, IAM e morte, a incidência de AVCI foi de 1,71%, IAM de 0,02% e óbito de 0,40%. O desfecho primário composto de EAM em 30 dias foi de 2,25%. A idade e o estado diabético foram os únicos preditores de risco independentes significativos; no entanto, as taxas totais de AVCI permaneceram abaixo de 2,6% em todos os subgrupos, incluindo octogenários sintomáticos. As outras variáveis demográficas iniciais, incluindo sexo do paciente, estado sintomático e oclusão da carótida contralateral, não foram preditores de risco independentes^296-298^ . Atualmente, somente há um dispositivo de oclusão proximal disponível no Brasil.

A diminuição de sinais embólicos durante o cruzamento da lesão, como o observado com o Doppler transcraniano quando comparado com o uso do filtro, é outro fator sugestivo que a proteção cerebral proximal é mais efetiva. Embora alguns estudos que descreverem redução nos eventos peri ACS, os resultados do uso de DPCA não têm o nível de evidência de um estudo randomizado^297^ . Devemos lembrar que os dispositivos de proteção cerebral proximal não devem ser usados em pacientes com lesão na carótida comum proximal ou estenoses extensas desta artéria, doença da artéria carótida externa ou em pacientes com oclusão contralateral e não compensado pelas demais artérias.

### Recomendação 6.4.1

Recomenda-se o uso de dispositivo de proteção cerebral antiembólica em pacientes submetidos a implante de *stent* na artéria carótida para redução de embolização cerebral (IIa/C)^288-291^ .

### Recomendação 6.4.2

O sistema de proteção cerebral (filtro, oclusão proximal de fluxo, reversão de fluxo) a ser utilizado no implante de *stent* na ACI é de escolha do cirurgião (IIa/B)^294-296,298^ .

### Recomendação 6.4.3

Os dispositivos de proteção cerebral proximal não devem ser usados em pacientes com lesão na carótida comum proximal ou extensa, com doença da artéria carótida externa ou com oclusão contralateral não compensada pelas demais artérias (III/C)^296,298^ .

### Dispositivo de reversão de fluxo por acesso transcarotídeo

O TCAR atualmente em uso nos EUA é realizado por abordagem proximal da artéria carótida, por dissecção, em um segmento saudável da mesma, na base do pescoço^299,300^ . A proteção cerebral é realizada pela oclusão proximal da artéria carótida comum (ACC), associado a reversão do fluxo via um circuito extracorpóreo da ACC para a veia femoral ou para a jugular ipsilateral^301^ . Associa-se assim as vantagens dos dispositivos de oclusão proximal a não manipulação do arco aórtico, visando a eliminar o risco de embolização. No trabalho de Kashyap et al.^242^ ocorreu redução de novas lesões cerebrais isquêmicas na imagem ponderada por difusão por RM de 33% após ACS via transfemoral *versus* 13% com TCAR (p = 0,03). O estudo ROADSTER-2 é um registro prospectivo, avaliando pacientes considerados de alto risco para EC com estenose sintomática ≥ 50% ou com estenose assintomática ≥ 80%. Dos 692 pacientes incluídos no estudo, 11 pacientes não atenderam aos critérios de inclusão, sendo descartados. Outros 48 pacientes foram submetidos à TCAR e foi descoberto após o procedimento que não haviam iniciado ou haviam interrompido seus medicamentos e foram excluídos. Dos 632 pacientes estudados, os critérios de alto risco para EC foram: 44% relacionados à anatomia, 32% risco clínico, e ambos em 24%. O sucesso técnico foi de 99,7%. Nos que terminaram o estudo, houve AVC em quatro pacientes (0,6%), IAM em seis pacientes (0,9%) e morte em um paciente (0,2%), levando a uma taxa composta de AVC/morte em 30 dias de 0,8% e taxa de AVC/morte/IAM de 1,7%^242^ .

Em uma revisão sistemática de TCAR com 18 estudos observacionais com 2.110 pacientes tratados (sucesso técnico de 98,25%), em 30 dias a taxa de AVCI maior foi 0,71%, AVCI menor em 0,90% e IAM de 0,57%, enquanto lesão de nervos cranianos ocorreu em 0,28%. Houve conversão para EC em 1,04%^301^ . Outra revisão sistemática do TCAR com 45 estudos envolvendo 14.588 pacientes que preencheram os critérios de elegibilidade pré-definidos foram incluídos na metanálise. A taxa de sucesso técnico foi de 99% (IC95% 98-99%). Os motivos da falha técnica incluíram incapacidade de cruzar a lesão e/ou falha na implantação do *stent*. As complicações no local de acesso ocorreram em 2% (IC95% 1%-2%; 30 estudos). Lesões de nervos cranianos ocorreram em 33 de 8.994 pacientes. Complicações hemorrágicas foram relatadas em 20 estudos e ocorreram em 2% (IC95% 1%-3%) de todos os casos. A taxa geral de AVC e a mortalidade por todas as causas periprocedimento foi de 1,3% e 0,5%, respectivamente. Restenose *intrastent* foi observada em quatro de 260 pacientes (1,5%; 7 estudos), e oclusão precoce (30 dias), assim como trombose aguda do *stent*, em 12 de 1.243 pacientes (∼1%; 11 estudos), concluindo que esse procedimento está associado a baixas taxas de AVC e lesão neurológica^302^ .

Parte das dificuldades desse procedimento transcarotídeo se sobrepõe com as da EC: devido à necessidade de dissecção cirúrgica, apesar de ser na base do pescoço, algumas situações como pescoço hostil (radioterapia cervical prévia, cifose, obesidade mórbida, imobilidade) ou mesmo o risco cirúrgico elevado podem impor alguma dificuldade. Outros fatores são próprios da técnica em si: placas calcificadas, carótida curta e pequena (diâmetro de menos de 6 mm e distância de menos de 5 cm do ponto de punção arterial até a bifurcação carotídea)^242,299-302^ .

### Acesso radial/braquial

O estudo RADCAR (acesso RADial para *stent* na artéria carótida) randomizou 260 pacientes para acesso transradial (TRA) e transfemoral (TFCAS). O sucesso do procedimento foi de 100%, com cruzamento de 10% durante a TRA para TFCAS, e 1,5% com TFCAS para TRA (p < 0,05). As complicações de acesso foram baixas (0,9% *versus* 0,8%), assim como as complicações cardíacas e/ou eventos cerebrais (0,9% *versus* 0,8%), mas as doses de radiação para o paciente foram significativamente maiores com TRA^269^ .

Não é sempre possível fazer o implante do *stent* carotídeo pela artéria radial, em particular na carótida esquerda^303^ . Em uma publicação multicêntrica com 214 pacientes, a implantação de filtro distal não foi possível em 7%, enquanto a proteção proximal não foi possível em 1,6%^304^ .

Em avaliação banco de dados da VQI com 23.965 pacientes submetidos à ACS, a abordagem transbraquial ou radial foi empregada em 819 pacientes (3,4%), enquanto a transfemoral, em 23.146 (96,6%). As características anatômicas foram mais propensas no sexo masculino (69,4% *versus* 64,9%, p = 0,009). Na análise univariada, os pacientes abordados por via braquial ou radial apresentaram taxas mais altas de resultados adversos, sendo que após ajuste para possíveis fatores de risco não houve diferença entre AVC ou morte (OR 1,10 [0,69-1,76], p = 0,675); no entanto, houve um aumento de mais de duas vezes no risco de infarto do miocárdio intra-hospitalar (OR 2,39 [1,32-4,30], p = 0,004) e de duas vezes no risco de falha técnica (OR 2,21[1,31-3,73] p = 0,003) quando comparado ao uso do acesso transfemoral. No uso de acesso, braquial ou radial, também foi observada uma redução de 50% no risco de complicações no local de acesso (OR 0,53 [0,32-0,85], p = 0,009)^305^ .

### Recomendação 6.5.1

Os seguintes fatores devem ser considerados desfavoráveis para ACS por meio de acesso femoral: idade superior a 75 anos, placa com calcificação extensa (> 13-15 mm) ou situações em que dois ou mais *stents* são necessários, tortuosidade dos vasos carotídeos e arco aórtico com placas ateroscleróticas tipo 3 (II/B)^242,301,304^ .

### Recomendação 6.5.2

Deve ser considerado o acesso radial/braquial para ACS como alternativa ao acesso femoral, particularmente nas lesões de carótida direita ou em situações de arco bovino, quando da carótida esquerda (IIa/B)^269,303,304^ .

### Recomendação 6.5.3

Os seguintes fatores devem ser considerados desfavoráveis para a realização de TCAR: pescoço hostil, radioterapia cervical prévia, cifose, obesidade mórbida, imobilidade, placas muito calcificadas e longas próximas da clavícula (< 5 cm), carótida de pequeno calibre (< 6 mm) e risco clínico elevado (III/C)^242,299-301^ .

### Recomendação 6.5.4

A revascularização da artéria por acesso transcarotídeo deve ser considerada como uma alternativa ao implante de *stent* transfemoral quando o *stent* conferir maior risco de complicações (IIa/B)^242,299-301^ .

### Desenho da malha do stent

#### *Stents* de células abertas e fechadas

Os *stents* são constituídos de células entre as hastes metálicas, classificadas em dois tipos: fechadas, caracterizados por pequenas áreas de células livres, e abertas, com lacunas descobertas maiores. Entre outras consequências dessas estruturas, estão a flexibilidade e sustentação do *stent*. Existe maior propensão a acotovelamento nos *stents* rígidos, os de células fechadas, e maior facilidade de protusão de detritos oriundos das placas, por extrusão, através das células maiores dos *stents* mais flexíveis^306^ .

Os resultados em relação aos *stents* de células abertas e fechadas são conflitantes. Alguns estudos com casuísticas significativas apontam para lados opostos. Em um registro alemão, com n = 13.086, com 4.356 casos com uso de *stent* de célula aberta, 6.554 com célula fechada e mista em 1.416 pacientes, houve uma tendência não significativa para menor taxa de AVC/morte intra-hospitalar com o uso de *stents* de células fechadas (2,3% *versus* 2,8% RR 0,86; IC95% 0,65-1,14, p = 0,30)^307^ . Já o registro SVS-VQI, de 1.384 ACS com uso de *stents* de células fechadas *versus* 1.287 de células abertas, com análises multivariadas, revelou que os *stents* de células fechadas foram associados a maior AVC/morte quando implantados na bifurcação carotídea (OR 5,5; IC95% 1,3-22,2, p = 0,02)^306^ . Em análise de metanálise de regressão com n = 46.728, o risco de ocorrência de AVC/morte dentro de 30 dias após a ACS foi semelhante em pacientes tratados com *stents* de células abertas *versus stents* fechados ou híbridos. Esse resultado persistiu nas análises de 1 ano. Os *stents* de células abertas foram associados com um número significativamente maior de NIBLs (novas lesões detectadas por RM de difusão - RR 1,25; p = 0,03), sem diferenças em relação à restenose, fratura de *stent* ou queda da pressão intraprocedimento^306^ .

#### *Stents* revestidos com malha de camada dupla

Os *stents* revestidos com malha de dupla camada são dispositivos autoexpansíveis de nitinol, que apresentam uma malha interna de diferentes composições (Quadro 10). O tamanho dos poros da malha quando o *stent* está totalmente expandido variam de 150-180 µm, muito próximo do calibre de algumas malhas dos DPCAs. O objetivo é criar uma barreira, impedindo a protusão da placa e eventual embolização cerebral.

**Quadro 10:** Dispositivo de oclusão proximal disponível no Brasil em 2023.

**Dispositivo e fabricante**	**Estudos**	**Taxa de AVC em 30 dias**	**Classe/Nível de evidência**	**Referências relevantes**
Mo.Ma^®^ Medtronic	Bersin ARMOUR trial Stabile	0.92% - 2.30%	IIb/B	Ansel et al.^296^ , Kassavin e Clair^297^ , Stabile et al.^298^

Foi publicada uma revisão sistemática e metanálise de estudos clínicos com *stents* de primeira geração (camada única) e *stents* de segunda geração (*micro mesh* – camada dupla). Os *stents* Casper^®^ e CGuard^®^ deste tipo são disponíveis no Brasil. Foram analisados dados de 68.422 pacientes de 112 estudos elegíveis, comparando os resultados entre os dois grupos. Houve uma redução na taxa de AVC e morte em 30 dias com o Casper^®^ (1,33%) e com o CGuard^®^ (1.08%), (2,78 e 3% absoluto, p = 0,02 e p < 0,001), comparado a 4,11% de AVC ou óbito obtidos com uso de *stents* de primeira geração. Aos 12 meses, em relação aos *stents* de única camada, o Casper^®^ reduziu a taxa de AVCI ipsilateral (-3,25%, p < 0,05), mas aumentou a taxa de restenose intra*stent* para 7.16% (+3,19%, p = 0,04). O CGuard^®^, por sua vez, apresentou redução da taxa de AVCI ipsilateral (-3,13%, p < 0,01) e a taxa de restenose intra*stent* para 0.34% (-3,63%; p < 0,01), comparado a 3,97% com os *stents* de única camada^308,309^ .

Em uma publicação de três centros italianos de alto volume, 150 pacientes foram tratados com *stent* Casper^®^. A avaliação da tomografia de convergência ótica (OCT) intraoperatória foi realizada em 26 pacientes, com uma análise *off-line* por um laboratório dedicado. Todos os pacientes foram submetidos à ultrassonografia duplex e avaliação neurológica em 24 horas e em 30 dias. A ACS foi tecnicamente bem sucedida em todos os casos e não foram observados eventos cerebrais intra-hospitalares em 30 dias. A avaliação com OCT detectou uma baixa taxa de prolapso de placa (dois pacientes, 7,7%). A ultrassonografia duplex mostrou perviedade do *stent* e da artéria carótida externa em todos os casos, tanto antes da alta quanto em 30 dias de seguimento^309^ .

O IRONGUARD Study é um estudo com 733 pacientes consecutivos submetidos à ACS usando o *stent* CGuard^®^ em 20 centros. Dispositivos de proteção embólica foram utilizados em 731 (99,72%) pacientes. O sucesso do procedimento foi de 100%, o sucesso técnico foi obtido em todos, exceto em um (99,86%) paciente, que morreu no hospital devido a um AVCH. Seis AITs, dois AVCs menores e um IAM ocorreram durante a internação (0,82%). A oclusão da artéria carótida externa foi evidente em oito (1,09%) pacientes. Entre a alta hospitalar e 30 dias de acompanhamento, ocorreram dois AITs, um AVC menor e três IAMs. A taxa de AVC cumulativa foi de 0,54%^309^ . O Quadro 11 mostra as diferenças estruturais entre os *stents* de dupla camada disponíveis atualmente.

**Quadro 11:** Diferenças estruturais entre stents de dupla camada.

**Diferenças de material**	**Casper®**	**CGuard®**
Estrutura do stent de nitinol	Célula fechada	Célula aberta
Material da malha	Nitinol	Tereftalato de polietileno
Desenho da malha	Trançada	Tricotada
Posição da malha	Malha interna	Malha interna

## DOENÇA ARTERIAL VERTEBRAL

### Introdução, conduta em indivíduos assintomáticos e pacientes com insuficiência vertebrobasilar

O estabelecimento de Diretrizes com Classes de Recomendação e de Níveis de Evidência de alto valor em relação à doença arterial vertebral (AV) esbarra no fato de que nunca foram realizados estudos clínicos randomizados avaliando a terapia desses vasos, seja em relação ao tratamento clínico-farmacológico, como com a terapêutica cirúrgica direta e a endovascular^5^ .

O sistema de irrigação vertebrobasilar tem inúmeras particularidades, sendo uma delas a grande circulação colateral, em grande parte pela peculiaridade de que é a única situação anatômica do corpo humano em que duas artérias se fundem para formarem um tronco único e não se bifurcarem. Isto ocorre quando as duas vertebrais se juntam no segmento V4 na base do crânio, formando o tronco basilar único. Assim, a doença obstrutiva de uma vertebral geralmente é compensada pela outra, se pérvia. Os fenômenos embólicos, mesmo existentes, não são tão comuns como das carótidas^310^ .

Em um importante estudo prospectivo, Compter et al.^311^ detectaram estenoses assintomáticas da AV em 282 (7,6%) de 3.717 portadores de doença aterosclerótica arterial. Acompanhando esses indivíduos por 4,6 anos, AVCI vertebrobasilar ocorreu em cinco desses, com um risco anual de 0,4%, ou seja, baixo. Os dados corroboram nossa impressão pessoal, de que indivíduos assintomáticos com estenoses das AVs devem receber TMO e não têm indicação de intervenção direta.

Indivíduos assintomáticos e pacientes sintomáticos portadores de doença AV devem receber tratamento médico otimizado, com controle da HAS, abolição do tabagismo, dieta adequada, controle metabólico (obesidade, DM e hiperlipidemia), AAP simples ou dupla e eventualmente anticoagulantes^5^ . Essas medidas são semelhantes às aplicadas nos pacientes portadores de doença arterial carotídea.

### Recomendação 7.1

Indivíduos assintomáticos e pacientes sintomáticos portadores de doença AV devem receber tratamento médico otimizado (I/B)^5^ .

A baixa incidência de transformação de indivíduos assintomáticos em pacientes com IVB não justifica uma intervenção profilática na doença AV sem sintomas^311-313^ .

### Recomendação 7.2

Indivíduos portadores de doença arterial obstrutiva vertebral assintomática não devem ser tratados profilaticamente com procedimentos invasivos (III/C)^311-313^ .

Mesmo assim, estima-se que cerca de 20% das isquemias cerebrais ocorram no território vertebrobasilar (VB)^314^ . As causas são múltiplas, sendo que a doença obstrutiva das AV e da basilar são responsáveis por cerca de 20-25% deste total. As demais são decorrentes de FA (25% do total), de trombose/microembolias a nível de pequenas artérias intracerebrais (outros 25%) e por outras causas, como doença obstrutiva dos TSAs. Antigamente se atribuía os sintomas VB sobretudo a causas hemodinâmicas, mas hoje se sabe que a maioria dos casos são embólicos^314-316^ . Em relação ao quadro clínico da doença das AVs e da IVB, sugerimos a leitura de extensa publicação recente nossa sobre o tema^310^ .

### Diagnóstico por imagem

O método de eleição para estudo do sistema VB é a ARM, permitindo estudar desde o arco aórtico até a circulação intracraniana, além de informar de maneira mais precisa sobre o parênquima cerebral envolvido^317-319^ . A ATC fornece melhor resolução espacial no estudo dos vasos, mas informa inadequadamente sobre o tecido cerebral. Esses métodos fornecem uma acurácia acima de 95% na detecção de estenoses/obstruções de vasos de grande e médio calibre e substituíram a angiografia por subtração digital como exame padrão-ouro no estudo da circulação VB^317-319^ . O estudo com eDc é muito empregado para a avaliação da origem das vertebrais, com demonstração de estenoses, oclusão, hipoplasia e aplasia da AV, mas não fornece informações mais acuradas sobre o restante dessa artéria. A acurácia nesse segmento proximal do estudo com eDc é em torno de 70%, mesmo em estudos recentes^318,320-322^ . A grande vantagem do eDc é no diagnóstico dinâmico, em tempo real, do fenômeno de furto de subclávia^320,323,324^ .

### Recomendação 7.3

Pacientes com suspeita de isquemia vertebrobasilar devem ser preferencialmente submetidos a estudos com ARM das artérias do arco aórtico, cervicais e intracranianas, com estudo simultâneo do parênquima cerebral. Estudo semelhante por ATC é uma segunda opção válida (I/B)^317-319,323^ .

### Tratamento intervencionista da doença sintomática da AV

Conforme demonstrado acima, não há indicação atual para tratamento profilático de lesões obstrutivas das AVs em indivíduos assintomáticos^311-313^ . Os sintomas mais comumente atribuídos à IVB são tonteiras e vertigens, sobretudo quando relacionadas à movimentação brusca da cabeça e pescoço, espontâneas ou em exercícios ou quiropraxia. Estudos sistemáticos não demonstraram alterações significativas de fluxo com diferentes métodos de aferição, inclusive Doppler transcraniano durante essas manobras, dentro de limites terapêuticos normais^325,326^ . Esses trabalhos sugerem que outras causas para os sintomas devam ser procuradas, e que investigação com métodos de imagem somente seja realizada se o exame físico revelar alterações sugestivas de doença arterial obstrutiva dos troncos supra-aórticos.

Como as intervenções para o tratamento de IVB somente têm indicação em pacientes sintomáticos, é importante que se conheça a evolução natural dessa enfermidade: Gulli et al.^327^ , em estudo recente de acompanhamento prospectivo de 90 dias após um AIT ou de um AVCI do território VB, mostraram que o risco de recorrência foi de 7% na ausência de doença da AV, de 16% com estenose da AV extracraniana associada e de 33% quando a lesão era intracraniana. Esses achados confirmam a nossa impressão e de muitos investigadores de que os pacientes que apresentam IVB devem ser submetidos a protocolos semelhantes aos pacientes com isquemia carotídea.

### Como tratar os pacientes com IVB que persistem sintomáticos apesar de TCO?

Atualmente, a maioria desses pacientes são tratados pelo método endovascular, embora as evidências de sua eficácia sejam limítrofes. Analisando criticamente os grandes estudos publicados recentemente, observamos resultados que favorecem sem significância estatística importante o TE da AV proximal. Já no segmento intracraniano, as estatísticas favoreceram claramente o TCO sobre uma abordagem endovascular, sendo esta não recomendada^328-333^ .

### Recomendação 7.4

Pacientes que persistirem com sintomas de IVB após TCO e apresentarem evidências de estenose 50-99% das AV proximais podem ser tratados pelo método endovascular, respeitadas suas limitações (IIa/B)^328-333^ .

Vários trabalhos recentes recomendam o uso de *stents* recobertos com drogas, sempre do tipo expansíveis por balão, no tratamento das lesões da origem da AV^334-336^ . Essas publicações afirmam que os resultados de perviedade em longo prazo são melhores do que com o uso de *stents* simples, com melhor resultado em relação à ausência de recorrência de sintomas e necessidade de reintervenções^334-336^ .

### Recomendação 7.5

No TE das estenoses das artérias vertebrais proximais, deve ser dada preferência ao uso de *stents* recobertos com drogas (IIa/C)^334-336^ .

Embora as técnicas cirúrgicas para tratamento de lesões da AV proximal e das subclávias tenham sido desenvolvidas há mais de 60 anos, e as da AV cervical distal há cerca de meio século, há poucos trabalhos de peso relatando os resultados e várias experiências menores com resultados muito díspares, algumas ruins, sendo a maioria das diretrizes das grandes sociedades que tratam desta enfermidade dúbias^5,8,63,154^ . A única que recomenda o tratamento cirúrgico de lesões da AV proximal é a SVS estadunidense, em pacientes sintomáticos de baixo risco cirúrgico^9^ .

As cirurgias diretas das AVs são várias, todas exigindo habilidades microcirúrgicas dos cirurgiões^310^ . No segmento proximal, a mais empregada é a transposição para a carótida comum; no segmento distal, o V3, preconizam-se pontes com safena devalvulada, usando a carótida comum ou a externa como fontes^310^ . Analisaremos aqui os quatro trabalhos de revascularização cirúrgica com mais de 50 pacientes tratados, de autoria de Habozit, Berguer, Kieffer e Ramirez^337-340^ . Nesses trabalhos, com um total de 1.339 pacientes operados, a taxa de EAM (AVC/óbito) foi de 2,3% na revascularização da vertebral proximal, de 3,0% nas distais, na base do crânio, mas atingiu 7,7% na cirurgia carotídea e vertebral combinada^337-340^ . Embora trabalhos menores apresentem resultados excelentes, inclusive sem mortalidade nesse cenário (o que coincide com nossa experiência), os números destes grandes estudos desautorizam de uma forma geral a realização da cirurgia carotídea e vertebral simultânea^341,342^ .

### Recomendação 7.6

Pacientes que persistirem com sintomas de IVB após TCO e apresentarem contraindicação ao TE podem receber tratamento cirúrgico direto, com reconstrução da AV (IIa/C)^337-342^ .

### Recomendação 7.7

Em pacientes com doenças carotídea e vertebral sintomáticas, a revascularização simultânea da carótida e da vertebral não é recomendada (III/C)^337-340^ .

## DOENÇA ARTERIAL MULTIFOCAL CONCOMITANTE

A aterotrombose pode ocorrer como um processo local, mas, com maior frequência, quando diagnosticada, apresenta uma distribuição multifocal. As artérias coronárias são um dos locais mais comumente afetados pela enfermidade, e as carótidas são muitas vezes comprometidas simultaneamente. A concomitância é conhecida de longa data e foi muito bem estudada por Schlosser et al.^343^ , que estimaram que 3-8% dos coronariopatas apresentam doença carotídea. O reverso é bem maior: 35-40% dos pacientes com obstrução carotídea apresentam enfermidade obstrutiva coronária.

Venkatachalam et al.^344^ , em avaliação mais recente, elevaram o percentual de estenoses carotídeas em coronariopatas para 12-17%. A Figura 1 sumariza tais achados, incluindo a DAP e os aneurismas.

**Figura 1:** Frequência das manifestações aterotrombóticas em diferentes territórios.

Nesta seção das Diretrizes, abordaremos a doença carotídea e coronariana concomitante, a interação entre a enfermidade obstrutiva carotídea com cirurgia não cardíaca e a doença arterial obstrutiva do tronco braquiocefálico e da carótida comum.

### Doença carotídea e coronária concomitante

Na presença de doença coronariana e carotídea grave (DCCG), a EC e a cirurgia de revascularização do miocárdio (CRM) têm sido empregadas em sequência ou de forma simultânea por mais de 40 anos. Desde publicações iniciais do manejo da DCCG, das quais destacamos a de Bernhard (1972), às mais atuais, a controvérsia persiste^344,345^ . Há cerca de uma década, a ACS passou a integrar essa disputa^346-348^ . Decidir qual a conduta ideal – a mais segura, simples e curta (em extensão do tratamento) – permanece um desafio.

Nos pacientes com doença carotídea e coronariana grave concomitante há quatro grupos básicos:

1. assintomáticos em ambos os territórios;
2. com sintomas cerebrovasculares isolados;
3. com sintomas coronarianos isolados;
4. sintomáticos em ambos os territórios.

Todos são potenciais candidatos à revascularização de ambos os territórios. Mas quais são os pacientes beneficiados, como proceder e quando? Tradicionalmente, há três estratégias: EC inicialmente, seguida de CRM; EC e CRM combinadas e CRM estagiada, seguida de EC. Recentemente, mais opções entraram nessa controvérsia: a realização de ACS semanas antes da CRM, realizar uma ACS imediatamente antes de CRM ou até a CRM sem revascularização carotídea, independentemente do grau de estenose carotídea.

Nos últimos 50 anos, uma incrível massa de informações foi acumulada neste tema, com milhares de pacientes e condutas; mas, na verdade, todas abordagens têm deficiências. A adição de procedimentos sobre as carótidas parece colocar o paciente em maior risco perioperatório de complicações cerebrais isquêmicas, IAM e/ou morte. Sabe-se que a incidência de AVC após CRM oscila entre 1-2%, mas na presença de doença obstrutiva grave pode atingir 17% de AVC e óbito neurológico^343,344,349-351^ .

A etiologia dos AVCs perioperatórios durante CRM é múltipla: embolismo de ar, cálcio, detritos ateromatosos aórticos, detritos do circuito extracorpóreo e trombos. Estes, junto com dissecção aórtica nas canulações, são responsáveis por cerca de 70% dos eventos, seguidos de hipotensão durante a circulação extracorpórea (CEC) propriamente dita, que é a etiologia dos demais 30%^352^ . Se a estenose carotídea fosse a causa mais importante de AVC durante a cirurgia cardíaca, os infartos cerebrais deveriam ser sempre ipsilaterais a um vaso gravemente lesado. Vários estudos confirmam que isso não é a verdade^343,344,349,350^ . Uma carótida gravemente estenótica ou ocluída pode até reduzir embolização ao hemisfério afetado^345^ . Após as canulações, durante o tempo de CEC, embolização cerebral parece improvável. Hipotensão perioperatória parece ter um papel importante, reduzindo o fluxo através de artérias estenosadas, favorecendo as tromboses. Como a prevenção da hipotensão durante a CEC não é total, todos pacientes com estenose grave das carótidas estariam em risco. Assim, por que pacientes com DCCG concomitante têm risco mais elevado de AVC e morte? Provavelmente porque este subgrupo de enfermos apresenta uma maior incidência de doença aterotrombótica multifocal, que eleva o risco geral dos procedimentos. Como já vimos, os dois mecanismos fisiopatológicos que são importantes neste cenário são as alterações hemodinâmicas e o embolismo.

A literatura evidencia que pacientes com eventos prévios de isquemia cerebral e que necessitam de CRM são os que estão em maior risco de AVC periprocedimento cardíaco – 18% quando apresentavam estenose carotídea de 70-99%, chegando a 26% quando as lesões eram bilaterais (ou com oclusão de uma ACI)^352,353^ . Já a presença de uma estenose carotídea assintomática não elevou significativamente a taxa de AVC pós-CRM, independentemente do grau de obstrução^354,355^ . Illuminati et al.^356^ , em um estudo prospectivo randomizado, avaliaram a realização de EC e CRM simultânea ou estagiada em indivíduos com estenoses carotídeas de 70-99% assintomáticas, submetidos à cirurgia cardíaca. Os dados mostraram que quando a EC foi realizada antes, e quando a EC e a CRM foram simultâneas, a mortalidade foi igual (1%), mas quando a EC foi realizada após, a taxa de AVC e morte foi de 4%. Já quando a EC foi postergada para após a CRM, a taxa de AVC e morte foi de 9%. É evidente que os pacientes sintomáticos com DCCG devem ser tratados das suas carótidas, ou antes ou simultaneamente com a CRM. Mas quais são os resultados das diferentes opções terapêuticas em coronariopatas com indicação de CRM que têm lesões carotídeas assintomáticas?

E qual método terapêutico – EC ou ACS – que oferece os melhores resultados nos pacientes com DCCG, sintomáticos ou assintomáticos?

A ACS somente tem aplicação nos pacientes com situações de urgência ou emergência se este método for usado simultaneamente com a CRM, pois um procedimento endovascular realizado previamente exige o uso de AAP dupla por um período mínimo de um mês, o que torna a CRM proibitiva pelo risco de sangramento, ou leva a uma procrastinação da CRM, o que pode ser prejudicial ao paciente. Na extensa revisão de Paraskevas et al.^349^ , em metanálise de 2.727 pacientes submetidos as ACS+CRM estagiada ou simultânea, a taxa de AVC e morte foi de 7,9%. A maioria das estenoses carotídeas (80%) eram assintomáticas. Se pacientes com história prévia de isquemia cerebral de qualquer grau forem selecionados, a taxa de AVC em 30 dias chegou a 15%. Os dados refutam a indicação de ACS neste cenário, de urgência e/ou emergência. Caso seja indispensável a realização de uma ACS associada à CRM, a antiagregação com AAS iniciada no pré-operatório deve ser mantida, e clopidogrel iniciado 12 horas após a cirurgia^349^ .

D’Agostino et al.^353^ e Naylor et al.^5^ , em suas revisões sobre o tema, concluem que há indicação de tratamento estagiado ou simultâneo das lesões carotídeas com CRM nos pacientes com história prévia de AIT ou AVC e em indivíduos com estenoses carotídeas de 70-99% bilaterais ou neste nível de obstrução associado à oclusão da carótida contralateral.

Uma das áreas com maior controvérsia na literatura médica concerne à conduta nos pacientes que necessitam uma CRM e apresentam estenoses carotídeas graves assintomáticas. Existem vários registros prospectivos e retrospectivos mono e multicêntricos de peso e valor, muitos estudos clínicos randomizados e metanálises com milhares de pacientes arrolados, sem que se tenha alcançada uma conclusão definitiva. Até diretrizes de grandes sociedades da especialidade divergem. A maioria das publicações estadunidenses, inclusive as Diretrizes atuais da SVS, recomenda estudos de imagem das carótidas (eDc, inicialmente) em todos os pacientes a serem submetidos à CRM. A EC ou eventualmente uma ACS deve ser realizada antes da CRM se possível ou, se as lesões forem graves nos dois territórios, operar concomitantemente. Essas Diretrizes consideram significativas estenoses carotídeas de 50-99%^9^ . A conduta foi baseada em muitos trabalhos estadunidenses, mas é acompanhada por estudos alemães^9,305,357-360^ . Já há trabalhos multicêntricos estadunidenses que indicam que não há diferença estatísticas entre EC simultâneas, estagiadas ou na realização CRM (com ou sem CEC), sem carotídea associada^361-363^ . E, no outro extremo, há trabalhos de peso, em sua maioria do Reino Unido, que recomendam que as carótidas nem deveriam ser estudadas nos indivíduos assintomáticos a serem submetidos à CRM, pois os resultados da cirurgia estagiada ou concomitante não diferem estatisticamente^364-370^ . Recentemente, um novo participante veio integrar a polêmica: a possibilidade de realizar uma ACS por acesso direto à carótida comum através da esternotomia, com utilização da técnica de reversão de fluxo e implante do *stent*, imediatamente antes instalação da CEC ou logo após; assim, sem nenhum acesso adicional e com pouco tempo cirúrgico agregado, uma estenose carotídea grave poderá ser tratada concomitantemente^371^ .

Com toda a experiência clínica acumulada, é evidente que o indivíduo com doença carotídea e coronária grave concomitante tem risco de complicações bem maior do quando a enfermidade é prevalente em um desses territórios. É também claro que a adição de um procedimento carotídeo coloca o paciente em risco elevado de complicações perioperatórias na CRM, de forma que, se indicado uma abordagem das coronárias e das carótidas, as evidências indicam que se deve:

- tratar o território sintomático primeiro, sempre que possível;
- sempre que viável, realizar a EC antes da CRM;
- intervenção simultânea somente na presença de –
  - angina instável e doença carotídea grave;
  - sintomas coronarianos e cerebrovasculares.
- nas cirurgias estagiadas com cirurgia carotídea inicial, realizar a CRM o mais breve possível;
- casos selecionados poderiam ser submetidos à CRM sem tratamento das carótidas.

Sem ter a menor pretensão de ter o poder de uma recomendação, tendo em vista as contundentes discussões acima expostas, o Coordenador destas Diretrizes toma a liberdade de apresentar aqui a conduta aplicada há 35 anos de tratamento de doença carotídea e coronária concomitante. Nossa preferência é pela cirurgia estagiada, sempre que possível, com a realização da EC sob ALR, com o paciente levemente sedado, mas desperto, pelo menos 4 dias antes da CRM^372,373^ . Na presença de DCCG com sintomas de ambos os territórios ou em coronariopatia grave com estenose carotídea assintomática, com estenose acima de 75% unilateral ou com oclusão contralateral, indicamos cirurgia simultânea. Situação complexa são pacientes com DCCG com comprometimento carotídeo bilateral e doença coronária sintomática. Nestes casos, incomuns, optamos por realizar endarterectomia da carótida mais obstruída junto com a CRM. Se simétricas, optamos pelo hemisfério dominante primeiro; a segunda carótida será tratada assim que as condições clínicas do paciente o permitirem. Indivíduos com estenoses assintomáticas de mais de 75% das carótidas, detectadas no pré-operatório de CRM, são tratados em cirurgia simultânea. Há necessidade de uma harmonia entre as equipes de cirurgia cardíaca e vascular, em benefício do paciente. Essa conduta é amplamente corroborada pela literatura^9,305,357-360^ .

### Como realizar um procedimento de EC e CRM simultâneo?

Embora existam algumas divergências de conduta entre os envolvidos neste tema, pela sua especificidade, pela falta de publicações sobre o assunto em nosso meio, vamos apresentar sumariamente nossa conduta e tática operatória nesses procedimentos combinados, padronizadas desde 1988: na presença de doença carotídea assintomática ou sintomática com indicação cirúrgica, na presença de coronariopatia assintomática, os pacientes são operados inicialmente da carótida, de preferência sob ALR, acordados, levemente sedados^372-374^ .

A tática operatória foi padronizada desde nossos primeiros casos: sob anestesia geral, enquanto os cirurgiões cardíacos realizam a colheita e preparo da veia safena ou de outros enxertos autógenos, nós acessamos a carótida. Eventualmente, a abordagem cervical se estende à esternotomia e ao preparo das artérias torácicas internas. Dissecção meticulosa e hemostasia com cautério são fundamentais. Após a finalização dos acessos e obtenção dos enxertos, o paciente é heparinizado com 1,2 mg/kg peso e as carótidas sequencialmente clampeadas; uma arteriotomia longitudinal interessando toda a extensão da placa a ser removida é realizada, e uma derivação interna temporária (*shunt*) implantada; segue-se a endarterectomia de toda a lesão, sob magnificação; todas as placas visíveis são removidas, com detalhamento do final da mesma (*end point*).

Certamente esta é a seção mais controversa destas Diretrizes. O volume de literatura consultada e as conclusões, às vezes conflitantes, demonstram que é um tema que deverá ser decidido no futuro^9,282,305,357-369^ .

Além da CRM, outros procedimentos da cirurgia cardíaca tem uma taxa importante de AVC durante sua hospitalização, sendo sempre o perioperatório o período com maior risco. As taxas de AVC variam com os procedimentos: uma CRM concomitante com troca valvar tem uma incidência de 4,2-13%, e nas trocas valvares aórticas percutâneas (TAVI), a incidência pode chegar a 3%^5,343,344^ . Os mecanismos são os mesmos descritos acima e a conduta em relação às carótidas também é semelhante^5^ .

### Doença carotídea e outras cirurgias não cardíacas

É de conhecimento comprovado que estenoses carotídeas com elevado grau de obstrução indicam um alto risco de AVC isquêmico^268^ . Teoricamente, qualquer indivíduo assintomático com uma estenose carotídea importante estaria em risco ao ser submetido a qualquer procedimento cirúrgico, nos quais sempre há tensão emocional, alterações da PA e frequentemente modificações da volemia, somente para citar alguns fatores.

### Como identificar os pacientes em risco?

O critério de indicar intervenção em assintomáticos com estenose a partir de 70% levaria a um volume excessivo de intervenções desnecessárias. Os custos com a intervenção são justificados? Onde está o bom senso?^370,375-383^

Frequentemente somos confrontados com questões referentes à necessidade de intervir em carótidas antes de cirurgias de vários tipos e categorias, em indivíduos com estenoses carotídeas assintomáticas, pelo receio do desenvolvimento de um AVCI perioperatório. O risco nas cirurgias em geral é bastante baixo – 0,2%. Ele se eleva a 0,5% nos portadores de sopros carotídeos, a 2,9% nos pacientes que sofreram um AVCI prévio, a 3,6% naqueles com estenoses carotídeas sintomáticas, e até 6% quando os sintomas são do território vertebrobasilar^282,364-388^ .

Sabemos dos trabalhos já citados de Schlosser & Venkachathalam que há elevada prevalência de estenose carotídea > 70% em pacientes com aneurisma da aorta abdominal e nos portadores de DAP aortoilíaca (DAP-AoIl)^343,344^ .

Pacientes com DAP têm a maior prevalência de doença carotídea importante simultânea – 14-49%. Essa prevalência tem uma relação direta com o estágio da DAP, assim como a idade: abaixo de 50 anos ela é próxima do zero, mas chega a 41% acima dos 80 anos. Em 2008, o Coordenador desta Diretriz fez um levantamento em no seu material, em 166 portadores de AAA e 184 pacientes com DAP-AoIl. Nos pacientes com AAA, levantamos retrospectivamente estenoses carotídeas importantes em 8,3% dos assintomáticos, e 73% nos que já haviam tido um AVCI. Nos pacientes com DAP-AoIl, o percentual era de 13,3% nos assintomáticos e 60% nos que já haviam sofrido isquemia cerebral (Arno von Ristow, dados não publicados).

Os mais importantes marcadores de doença obstrutiva carotídea são a idade acima dos 70 anos, história de AVCI prévio, história de doença coronariana, DAP e de aneurismas aórticos, além de baixo índice tornozelo/braquial e DM. Conhecendo tais fatores de risco, os pacientes pertencentes a esses grupos devem ter seus TSAs estudados, primariamente com eDc^282,343,344,375-388^ .

### Recomendação 8.1.1

Pacientes com doença cerebrovascular assintomática, com indicação de cirurgias eletivas em geral, sem fatores de risco para doença aterotrombótica cerebrovascular conhecidos, podem ser operados sem realização de exame físico específico ou estudos de imagem dos TSAs (I/A)^370,375-383,388^ .

### Recomendação 8.1.2

Pacientes com doença cerebrovascular assintomática, com indicação de cirurgias eletivas em geral, mas portadores de um ou mais fatores de risco para doença aterotrombótica cerebrovascular conhecidos (idade acima dos 70 anos, história de AVC-I prévio, história de doença coronariana, história de DAP, baixo índice tornozelo/braquial e DM), devem receber investigação para detecção de doença carotídea, com exame físico específico e estudo com eDc dos TSAs (I/A)^281,343,344,370,375-388^ .

A metanálise de Jørgensen et al.^389^ confirmou que dos 7.137 pacientes operados de cirurgias, em geral operados dentro de 3 meses após sofrerem uma isquemia cerebral (de um total de 481.183 indivíduos), apresentaram 11,9% de AVCI nas cirurgias realizadas. Esse percentual caiu para 4,5% entre 3 e 6 meses, e para 1,8% entre meio e 1 ano. A maioria dos AVCs foram isquêmicos, por doença obstrutiva arterial ou cardioembólico. Já nos operados que nunca haviam sofrido um AVC, a taxa foi de 0,1%.

### Recomendação 8.1.3

Pacientes com indicação de cirurgias eletivas em geral que apresentaram sintomas de doença cerebrovascular nos últimos 6 meses devem receber investigação para detecção de doença carotídea, com exame físico específico e estudo com eDc dos TSAs (I/A)^343,344,370,384-387,389^ .

### Recomendação 8.1.4

Em pacientes com indicação de cirurgias eletivas em geral que apresentaram sintomas de doença cerebrovascular nos últimos 6 meses relacionados com doença carotídea, nos quais foram detectadas estenoses de 50-99% das carótidas, uma revascularização carotídea deve ser realizada antes da cirurgia geral programada (I/B)^384-387,389-391^ .

Além dos vários fatores citados, a suspensão dos medicamentos AAP e de anticoagulantes agravam os prognósticos, devendo ser retirados pelo menor tempo possível e substituídos por agentes de curta duração, como HBPM ou mesmo heparina não fracionada até próximo da cirurgia^390-392^ . Estatinas não necessitam e não devem ser suspensas ou terem sua dosagem reduzida^390-392^ .

### Recomendação 8.2.1

Pacientes com indicação de cirurgias eletivas em geral, em uso de estatinas, não devem ter seu uso suspenso ou reduzido para a realização do procedimento programado (I/B)^390-392^ .

### Recomendação 8.2.2

Pacientes com indicação de cirurgias eletivas em geral, em uso de AAP, devem ter seu risco hemorrágico individualmente determinado. Se for julgada necessidade de suspensão temporária dos AAP por risco hemorrágico, seu uso deve ser retomado o mais breve possível após a intervenção, de preferência no dia seguinte (I/B)^390-392^ .

### Recomendação 8.2.3

Pacientes com indicação de cirurgias eletivas em geral, em uso de anticoagulantes, devem ter seu risco hemorrágico individualmente determinado. Se for julgada necessidade de suspensão temporária dos anticoagulantes por risco hemorrágico, proteção adicional pode ser obtida com uso de HBPM ou heparina não fracionada venosa até o mais próximo possível da cirurgia, com retorno do uso do anticoagulante usual o mais breve possível após a intervenção, de preferência no dia seguinte (I/B)^390-392^ .

As diretrizes de grandes sociedades internacionais da especialidade divergem sobre a conduta nos indivíduos com doença carotídea obstrutiva assintomática neste cenário de cirurgia geral, não cardíaca. A Diretriz alemã-austríaca não aborda o tema^154^ .

A ESVS não recomenda nenhuma intervenção carotídea como profilaxia de complicações cerebrais na presença de estenoses assintomáticas de 50-99%^5^ . A SVS estadunidense indica que a conduta de investigação e tratamento deve ser a mesma para a população geral^9^ .

### Recomendação 8.4

Indivíduos com doença cerebrovascular assintomática, com indicação de cirurgias eletivas em geral, mas portadores de estenoses carotídeas significativas (> 75%), devem ter a conduta em relação ao tratamento das carótidas baseada na experiência do grupo responsável por seu tratamento. A indicação deve ser a mesma definida para indivíduos com doença carotídea assintomática sem indicação de cirurgias eletivas em geral. Se indicada intervenção, deve ser preferencialmente realizada antes das cirurgias eletivas em geral (III/B)^5,9,385^ .

Um dos questionamentos que nos defrontamos com maior frequência é a necessidade ou não de suspender ACO e, sobretudo, AAPs, antes de procedimentos oftalmológicos, principalmente as facectomias – cirurgias de cataratas. Como a faixa etária destes procedimentos incide em uma população com muitas enfermidades cardiovasculares que utilizam essa classe de drogas, há preocupação de todos os envolvidos com a situação – oftalmologistas, cardiologistas, cirurgiões vasculares e, é claro, os pacientes. Cerca de um cada quatro pacientes operados de catarata estavam em uso de drogas antitrombóticas em três grandes metanálises publicadas^393-395^ . Metanálises com dezenas de milhares de pacientes^393-395^ e várias publicações de centros únicos, bem elaboradas, em estudos randomizados, sugerem que as facectomias por facoemulsificação podem ser realizadas sem a suspensão dos AAP, se o procedimento for realizado sob anestesia tópica e sedação^396,397^ . Já quando se realiza a infiltração anestésica retrobulbar, o risco de hemorragia sobe e recomenda-se a suspensão dessas drogas para a cirurgia (o risco de hemorragias sobe de 0,2-1,0%, sem, entretanto, comprometer o resultado final da intervenção)^393,394^ . Existem estudos que recomendam a mesma conduta quando um ACO do grupo da varfarina estiver em uso, o que atualmente é incomum, uma vez que a maioria dos pacientes anticoagulados atualmente utilizam os ACO diretos – rivaroxabana, dabigatrana, apixabana e edoxabana. Em relação à tais drogas, os ACOs diretos, não há estudos conclusivos, mas o bom senso indica que devam ser suspensos 48 horas antes da cirurgia ocular e reiniciados no dia seguinte se for empregada a anestesia por infiltração retrobulbar. Nos pacientes de altíssimo risco cardiovascular de suspensão dos AAPs e dos ACOs recomenda-se intervir sem suspensão das drogas e realizar a cirurgia sob anestesia tópica e sedação^393-399^ .

### Recomendação 8.5.1

Cirurgias de catarata com facoemulsificação e implante de lente intraocular, nas quais seja contraindicada a suspensão de AAP pelo elevado risco de eventos cardiovasculares, podem ser realizadas sob anestesia tópica e sedação (I/A)^393-399^ .

### Recomendação 8.5.2

Em cirurgias de catarata com facoemulsificação e implante de lente intraocular sob anestesia retrobulbar, os AAP e anticoagulantes devem ser suspensos pelo período adequado para cessar o efeito específico de cada droga. Os AAP e/ou anticoagulantes devem ser reiniciados no dia seguinte à cirurgia (I/A)^393-396,398^ .

### Recomendação 8.5.3

Pacientes em uso de anticoagulantes com alto risco de eventos trombóticos com a suspensão dessas drogas, com indicação de cirurgia de catarata com facoemulsificação e implante de lente intraocular, podem ser operados sob anestesia tópica sem suspensão dos anticoagulantes, com baixo risco de complicações hemorrágicas. Recomenda-se acesso a equipes de oftalmologia de grande conhecimento técnico (II/B)^394,395,399^ .

### Doença arterial obstrutiva dos troncos supra-aórticos

Consideram-se como TSAs os ramos originados diretamente da croça da aorta, que são o tronco braquiocefálico (TBC), a artéria carótida comum esquerda (ACCE) e a artéria subclávia esquerda (ASE). Na prática cirúrgica, as artérias carótida comum direita (CCD) e a subclávia direita, embora esta última seja raramente envolvida em processos obstrutivos, também são englobadas nesta classificação, pois além de participarem da irrigação cerebral e dos membros superiores, como os três troncos proximais, compartilham entre si as mesmas patologias e problemas operatórios^310^ . A etiologia mais frequente das obstruções dos TSAs é a aterotrombose. Entre etiologias mais raras, temos as arterites (doenças de Takayasu e Horton, principalmente) dissecções, aneurismas e tortuosidades associadas a acotovelamentos estenóticos, lesões congênitas com comprometimento obstrutivo (agenesias, divertículo de Kommerell) e outras, como embolias, lesões actínicas, displasia fibromuscular, ergotismo e fibrose mediastinal^310^ . Os procedimentos de revascularização dos TSAs são realizados sobretudo para tratamento de sintomas de insuficiência vascular cerebral, isquemia de membro superior e para revascularizar uma subclávia obstruída, como preparo para uso da artéria torácica interna em pontes coronarianas^310^ .

A cirurgia dos TSAs apresenta duas características singulares dentro da cirurgia vascular: existem várias patologias envolvidas e seu tratamento exige competência em procedimentos de revascularização cerebral, de cirurgia torácica e, mais recentemente, de angiorradiologia. A cirurgia reconstrutora dos TSAs sempre foi incomum nos serviços de cirurgia vascular e se tornou ainda mais rara com a possibilidade de TE na maioria dos casos^310,400,401^ . Somente em uma pequena porcentagem, de 3-7% do total de revascularizações das artérias de irrigação cerebral, atua-se nos TSAs^310,400,401^ .

### Histórico

A primeira ressecção de aneurisma do TBC foi realizada por Oudot, em 1952. Deve-se a Bahnson a primeira ponte para tratamento de lesão obstrutiva do TBC, e Davies realizou a primeira endarterectomia desta artéria. Cate & Scott realizaram a endarterectomia da subclávia esquerda em 1957, por toracotomia. North, DeBakey e Crawford, em 1961, realizaram este procedimento por via cervical e no mesmo trabalho publicaram a primeira ponte carotidosubclávia. Parrot, em 1963, implantou a subclávia na carótida. Seguiram-se vários procedimentos de pontes transcervicais, por Warren, Ehrenfeld & Myers^310,400,401^ . A síndrome do furto da subclávia recebeu este nome por sugestão de Fischer, após a descrição do quadro clínico por Contorni, em 1960. Indiscutivelmente, este interessante fenômeno circulatório aumentou o interesse pela doença vertebrobasilar^310,400,401^ .

A introdução da abordagem endovascular veio dar novo impulso ao tratamento da doença arterial obstrutiva dos TSAs. Mathias realizou a primeira angioplastia com balão de subclávia, na Alemanha, em 1980^402^ . É discutível quem realizou o implante do primeiro *stent* em subclávias, pois a com a introdução deste dispositivo, muitos o aplicaram em diferentes artérias, quase simultaneamente^400,401^ . Atualmente, o TE das doenças obstrutivas dos TSAs é o mais empregado, eventualmente associado à cirurgia em um procedimento híbrido^310,400,401^ .

Na literatura, são relativamente poucas as publicações com grande volume de casos envolvendo o tratamento da doença arterial obstrutiva dos TSAs, sobretudo em relação às lesões ostiais no arco aórtico. Desta forma, as Recomendações nesta Seção são baseadas muito em trabalhos monocêntricos e pouco em estudos multicêntricos e em somente uma metanálise sistemática^9,403-410^ . Poucas diretrizes de outras sociedades de especialidade abordam em profundidade o tema^5^ .

A primeira Recomendação é derivada de consenso entre especialistas, de que lesões assintomáticas dos TSAs não necessitam terapia cirúrgica ou endovascular, devendo ser mantidos em tratamento conservador. Esta é uma situação encontrada em muitos pacientes com obstrução do terço proximal da artéria subclávia esquerda, que, se assintomáticos, não necessitam revascularização.

### Recomendação 9.1

Indivíduos portadores de lesões obstrutivas assintomáticas dos TSAs não têm necessidade de intervenção cirúrgica ou endovascular, devendo ser mantidos em tratamento conservador. III/C – Consenso de especialistas.

As oclusões arteriais crônicas dos TSAs e das AVs podem acarretar uma grande variedade de sintomas e sinais, muitas vezes idênticos aos associados à doença da bifurcação carotídea. Uma detalhada história clínica e um minucioso exame físico são essenciais para detectar estas lesões. O quadro clínico pode-se manifestar de quatro formas básicas: isquemia cerebral anterior, isquemia vertebrobasilar, isquemia dos membros superiores e sintomas associados^310^ . No exame físico, atenção especial deve ser dada à palpação dos pulsos carotídeos, subclávios e das artérias ao longo do membro superior. As artérias devem ser auscultadas ao nível do foco aórtico, fúrcula esternal, fossa supraclavicular e nos terços médio e distal do pescoço (ângulo da mandíbula). Sopros são encontrados na maioria dos casos (85%), assim como pulsos de amplitude diferentes nos membros superiores (65%)^310^ .

O eDc têm sido extensivamente usados no diagnóstico da patologia dos TSAs, frequentemente com resultados imprecisos e contraditórios na investigação^5,310^ . Há necessidade de profundo conhecimento anatômico, de fisiologia do fluxo sanguíneo e da fisiopatologia envolvidas, além de um elevado índice de suspeita por parte do examinador, para que possamos obter uma avaliação de valor. Muito úteis na avaliação da direção do fluxo sanguíneo, muitas vezes alterado nestas situações, a determinação do grau de estenose e a extensão das lesões são ainda imprecisas com este método.

O estudo detalhado do arco aórtico e dos TSAs é imprescindível para o planejamento operatório. A ATC substitui progressivamente os exames angiográficos para fins de diagnóstico. Embora a arteriografia com subtração digital ainda seja considerada padrão-ouro para a avaliação da doença obstrutiva dos TSAs, a ATC obtida com tomógrafos de múltiplos detectores, aliada a diferentes tipos de reconstrução de imagens, fornece imagens precisas da anatomia patológica e permite avaliar a presença e a extensão das lesões, as dimensões dos vasos e de seu lúmen, além das características da parede vascular. A ATC do tórax auxilia, sobremaneira, na avaliação dos aneurismas e das anomalias congênitas. É o exame mais completo para o estudo dos TSAs disponível atualmente^5,9,310,400,401,403,405,407,409,410^ .

### Recomendação 9.2

Na avaliação diagnóstica das obstruções dos TSAs, o estudo com ATC, complementado ou não com angiografia digital, é o exame que fornece mais informações para o planejamento terapêutico (IIa/A)^5,9,403,405,407,409,410^ .

À semelhança com outros territórios da nossa especialidade, como os aneurismas da aorta abdominal, a abordagem dos TSAs foi realizada por décadas por cirurgia direta, com excelentes resultados e baixa morbimortalidade^310,406,408^ . Nos últimos 30 anos, o TE tomou a dianteira e, atualmente, são raros os casos em que se exige uma abordagem cirúrgica totalmente aberta^310,403-405,407,409,410^ . As possibilidades de reconstrução cirúrgica direta são múltiplas e merecem a atenção em publicações extensas em nosso meio^310^ . Das publicações empregando a cirurgia direta, destacamos a clássica de Fry et al.^406^ , de 1992, com 20 pontes subclaviocarotídeas, nas quais quatro tiveram uma EC associada: um óbito hospitalar por IAM e nenhuma restenose tardia em 5 anos. A maioria das pontes era confeccionada com veia safena autógena naquela época, mas a degeneração tardia sobretudo com alongamento dos enxertos e o aperfeiçoamento das próteses de, especialmente de PTFe, fazem com que esses sejam os condutos mais utilizados atualmente^406-408,410^ . Outro trabalho importante é de Takach et al.^408^ , de 2005, com 113 casos tratados por toracotomia e 44 por vias extra-anatômicas, com idade média 54 anos. CRM concomitante foi realizada em 37 destes (23,6%) e EC em 26 (16,6%). Surpreendentemente, ocorreu AVC maior nas revascularizações extra-anatômicas (2,7% *versus* 6,8%), mas mortalidade precoce semelhante nos dois grupos (2,7% *versus* 2,3%). Houve resultados tardios melhores no grupo de cirurgia direta (94,4% de perviedade em 10 anos contra 60,3%), independentemente do número de anastomoses distais. Dos trabalhos comparativos entre cirurgia direta com procedimentos híbridos e exclusivamente endovasculares destacamos os seguintes: de Vries et al.^407^, já em 2005, destacaram que o TE teve sucesso técnico em 93% de seus 110 casos. O acesso femoral foi o mais empregado, 81%, o braquial em 5,4%, e o combinado em 13,6%. *Stents* só foram implantados em 58% dos pacientes. Relatam AVC em 2% dos casos e morbimortalidade de 3,6%. Perviedade em 5 anos: 89%.

Reoperações foram necessárias por recidiva de sintomas em 7,2%, sendo que a metade necessitou de cirurgia direta. Chamam atenção para a necessidade de acompanhamento mais frequente nos primeiros 2 anos após TE^403^ . Bakken et al.^403^ , em 2008, relatam sucesso técnico em 98% com o TE dos TSAs, com mortalidade zero e morbidade maior em 2%, A perviedade em 3 anos foi de 88%, sendo necessário reintervir em 7% dos casos. Outro estudo monocêntrico foi publicado por van der Weijer et al.^409^ , com 114 pacientes portadores de 144 lesões dos TSAs, com idade média de 66,3 anos. O TE foi indicado em 137 lesões, a maioria tratada por recanalização, angioplastia e implante de *stents* (angioplastia pura/com implante de stent): TBC-9/54; CCE 0/7; ASE 11/56. Houve sete insucessos, abortando o procedimento. O sucesso técnico foi de 94,4%, com zero morbimortalidade em 30 dias. A perviedade em 5 anos foi de 83,2%, bem correlacionada com ausência de recidiva de sintomas em 82,3% (a maioria destas dentro de 2 anos). As recidivas puderam ser tratadas mais uma vez por TE. Na avaliação diagnóstica das obstruções dos TSAs, o estudo com ATC, complementado ou não com angiografia digital, é o exame que fornece mais informações para o planejamento terapêutico. Trabalhos recentes chamam atenção: Zacharias et al.^410^ publicaram os resultados de revascularizações abertas, híbridas e endovasculares do TBC em seu centro, em 2020: de 33 casos, 64% eram sintomáticos, e nos 36% restantes, o procedimento foi combinado com EC. Em quatro pacientes, uma ponte originária da aorta ascendente foi necessária em virtude de lesões altamente calcificadas do arco aórtico, envolvendo o óstio do TBC e este tronco mesmo, ou por falha do TE. Utilizaram sistemas de proteção cerebral sempre que possível, através de clampeamento distal nos procedimentos híbridos e filtros distais, nos puramente endovasculares. Obtiveram bons resultados – 97% de sucesso técnico, com um caso de óbito (3%) por AVC/reperfusão, e excelente perviedade em longo prazo, com restenoses em 9% dos casos^410^ .

A metanálise sistemática de 1.969 pacientes de 77 estudos de revascularizações dos TSAs, publicados ao longo de 57 anos, avaliados por Robertson et al.^407^ em 2020, apresenta resultados semelhantes: 7% de morbimortalidade na cirurgia aberta, 3,3% na cirurgia híbrida, e 1,5% na puramente endovascular. Restenoses tardias se situaram entre 2,6-10,5%, chamando atenção para a necessidade de acompanhamento por toda vida. Já o estudo multicêntrico baseado no banco de dados da VQI da SVS estadunidense, publicado por DeCarlo et al.^405^ em 2021, avalia 18.886 casos de ACS, dos quais 809 apresentavam lesões sequenciais nas carótidas comuns proximais, associadas a estenoses da bifurcação carotídea. Risco de AVC /morte dobrou nos casos de TE concomitante de ambas as lesões – 3,4 *versus* 1,8% – independentemente de os pacientes serem ou não sintomáticos. Recomendam não realizar o TE puro na associação de lesões.

Todos estes trabalhos recomendam o TE dos TSAs como seguro e eficaz e o método de eleição, quando factível. Situações pontuais demandam cirurgias, inclusive com esternotomia, extra-anatômicas ou híbridas, com pequeno aumento do risco operatório e bons resultados tardios^406-408,410^ . Lesões associadas dos TSAs com estenoses graves da bifurcação carotídea são tratadas com melhor resultado com procedimentos híbridos: TE de lesão proximal e EC da bifurcação, usando medidas protetivas antiembólicas^5,9,310,401,405,407,410^ . Toda literatura chama atenção para a necessidade de acompanhamento mais frequente nos primeiros 2 anos após TE, quando as recidivas são mais frequentes, e recomendam a necessidade de acompanhamento por toda vida, pois a maioria dos pacientes são mais jovens do que os arteriopatas que usualmente se apresentam com aterotrombose e podem ter longa sobrevida^403-405,407-410^ .

### Recomendação 9.3

Pacientes com lesões obstrutivas sintomáticas dos TSAs devem ser preferencialmente tratados com procedimentos endovasculares ou híbridos (IIa/B)^403-405,407,409,410^ .

### Recomendação 9.4

Quando situações anatômicas, anatomopatológicas ou clínicas impedem o TE, lesões obstrutivas dos TSAs em pacientes sintomáticos podem ser tratadas com procedimentos cirúrgicos diretos ou extra-anatômicos (I/B)^405,406,408,410^ .

### Recomendação 9.5

Lesões nos TSAs associadas a estenoses graves da bifurcação carotídea são tratadas com melhor resultado com procedimentos híbridos: iniciar com o TE da lesão proximal e EC da bifurcação, usando medidas protetivas antiembólicas (I/B)^5,9,310,401,405,407,410^ .

O conhecimento médico atual e os vários métodos diagnósticos disponíveis atualmente permitem um diagnóstico preciso e estabelecimento de um prognóstico com muita acurácia. Os métodos de prevenção dos AVCI e AVCH são eficientes e custo efetivos. Indivíduos assintomáticos pertencentes aos grupos de risco conhecidos devem ser criteriosamente avaliados, e aqueles com risco elevado de isquemia cerebral devem ser tratados especificamente, se tiverem acesso a serviços de níveis de complicações mínimos.

## COMPLICAÇÕES

### Complicações do tratamento cirúrgico aberto das carótidas

O tratamento cirúrgico por meio da EC tem um papel relevante no tratamento da doença aterosclerótica da carótida extracraniana desde os anos 1950. Ao longo destas sete décadas, a EC foi minuciosamente estudada em todos os seus aspectos: indicações, técnicas e complicações. A vasta literatura acumulada permite a análise das complicações associadas à EC e sua prevenção.

As complicações da EC podem ser classificadas em:

- anestésicas e hemodinâmicas;
- cirúrgicas;
- vasculares;
- neurológicas.

### Complicações anestésicas e hemodinâmicas

A anestesia para EC pode ser geral ou locorregional. A anestesia geral é a técnica original, utilizada desde a década de 1950. A partir dos anos 1970, foi introduzido o bloqueio locorregional cervical (BLC) para EC. Ambas as técnicas são amplamente utilizadas. Uns poucos serviços utilizam a anestesia local por infiltração com sedação leve para monitorizar o *status* neurológico do paciente durante todo o procedimento^411^ .

### Anestesia geral X bloqueio locorregional cervical

As vantagens da anestesia geral são: controle da via aérea durante todo o procedimento, a abolição da ansiedade do paciente; a possibilidade de usar anestésicos neuroprotetores (por exemplo, propofol, isofluorano) e melhor controle da PA. As desvantagens são: depressão do miocárdio com hipotensão, necessidade de monitorização da isquemia cerebral durante o clampeamento da carótida, maiores taxas de isquemia do miocárdio e complicações pulmonares^412^ .

A principal vantagem do BLC é a monitorização contínua da perfusão cerebral no paciente desperto. As desvantagens são a ansiedade e desconforto do paciente, que podem desencadear taquicardia e HAS; maior incidência de hematomas cervicais nos pacientes com dupla antiagregação plaquetária^413^ ; e complicações anestésicas, como o bloqueio do nervo frênico e anestesia subdural inadvertida^414^ . O estudo GALA^254^ , o maior RCT já realizado comparando anestesia geral e BLC e uma revisão Cochrane^415^ não mostraram diferenças significativas quanto às taxas de AVC/óbito em 30 dias entre as duas técnicas. No entanto, outras metanálises de RCTs mostram que a anestesia por BLC reduziu significativamente: lesões isquêmicas cerebrais pós-operatórias; complicações pulmonares; lesão de nervos cranianos; duração da cirurgia; tempo de permanência no hospital; perda sanguínea e taxas do agregado AVC/óbito em 30 dias^416-422^ .

Como qualquer outra decisão clínica, a escolha da técnica de anestesia para EC deve levar em conta a expertise da equipe anestésica e a situação clínica e preferência do paciente.

### Recomendação 10.1

O método de anestesia – geral ou bloqueio locorregional – deve ser decidido pela equipe cirúrgica/anestésica, levando em conta a experiência local, a condição clínica e a preferência do paciente (IIa/B)^253,415-422^ .

### Hipertensão arterial sistêmica

A HAS, definida como PA sistólica > 180 mmHg durante a EC e no pós-operatório imediato, tem influência significativa sobre as seguintes complicações: sangramento e formação de hematomas; infarto do miocárdio; insuficiência cardíaca; IAM; ICC aguda; hemorragia intracraniana (HIC); e a SHC^423^ . A EC por eversão está associada a taxas de HAS intra e pós-operatórias significativamente mais altas do que a EC convencional^424^ .

O controle estrito da PA durante a operação e nas 24-48 horas seguintes é a medida mais importante para reduzir essas complicações^215,425^ . Protocolos de controle da PA no período crítico que compreende a operação e o pós-operatório imediato estão disponíveis na literatura^215^ . Embora não haja estudos publicados, é razoável assumir que os riscos da HAS descontrolada também se aplicam aos pacientes submetidos à ACS.

### Recomendação 10.2

O controle estrito da HAS durante e no pós-operatório da EC reduz complicações cirúrgicas, cardíacas e neurológicas. Protocolos de controle da PA devem ser implementados em centros que executam EC (IIa/B)^215,425^ .

### Hipotensão arterial

A hipotensão pode ocorrer durante a EC por diferentes causas^426^ . Na indução anestésica, a hipotensão é causada pelo efeito depressor das drogas anestésicas sobre o miocárdio. No decorrer da operação, pelo nível da anestesia e o uso da protamina para reverter os efeitos da heparina. No pós-operatório, uma causa comum é o efeito hipotensor da estimulação dos barorreceptores da bifurcação da carótida^427^ . Nessa condição, a remoção de uma placa de ateroma rígida faz com que a parede da carótida recupere a pulsatilidade natural, estimulando fortemente os barorreceptores localizados no seio carotídeo e desencadeando bradicardia e hipotensão. A hipotensão pode se prolongar por vários dias. Em séries de pacientes que desenvolveram hipotensão/bradicardia após EC e ACS, não foram observados efeitos adversos graves ou aumento da morbimortalidade^428^ .

### Complicações cirúrgicas

#### Lesão de nervos cranianos e cervicais

No seu trajeto no pescoço, a carótida comum e seus dois ramos, carótida interna e carótida externa, cruzam ou são adjacentes a cinco nervos cranianos: VII – facial (ramo marginal da mandíbula); IX – glossofaríngeo; X – vago; XI – acessório; e XII – hipoglosso. Dada a proximidade anatômica, a lesão de nervos cranianos é complicação relativamente comum da EC, ocorrendo em cerca de 5% das operações, sendo a do ramo marginal da mandíbula a mais frequente^236^ . Os fatores que predispõem a lesão de nervos cranianos são: AG, operações de urgência, reoperações por sangramento e operações em pescoço irradiado^429^ .

As lesões mais comuns são do ramo mandibular do nervo facial, do nervo hipoglosso e do nervo laríngeo recorrente (ramo do nervo vago)^430^ . Lesões do ramo mandibular do nervo facial provocam desvio da rima labial. As lesões do nervo hipoglosso e do nervo glossofaríngeo provocam dificuldade de mastigação e disfagia.

Quase todas essas lesões de nervos cranianos se resolvem espontaneamente em semanas ou meses. A exceção é a lesão do nervo laríngeo recorrente, que pode resultar em paralisia permanente da corda vocal e distúrbios de fala e da deglutição. O paciente com lesão permanente de um nervo laríngeo recorrente não deve ser submetido à EC contralateral^431^ .

#### Hemorragia e hematoma cervical

Praticamente todos os pacientes que são submetidos à EC estão sob o efeito de drogas antiplaquetárias^432^ . Durante a operação, é administrada heparina EV em dose terapêutica, visando a anticoagular totalmente o paciente. Esses dois fatores, mais a abundante circulação arterial do pescoço, fazem com que sangramento persistente e a formação de hematoma cervical, levando à reoperação, ocorram em 2-10% das operações^433^ . Os hematomas quase sempre acontecem nas primeiras horas depois da cirurgia, geralmente em pacientes com PA não controlada. Outra causa de sangramento persistente é a heparinização, que deve ser revertida pela infusão lenta de protamina antes do fechamento incisional. Metanálise de sete estudos mostrou que a administração de protamina reduz em mais de 50% a formação de hematomas que requerem reoperação^434^ .

O controle estrito da PA, conforme preconizado na Recomendação 10.2, e a reversão dos efeitos da heparina com a protamina são as medidas necessárias para reduzir as complicações hemorrágicas da EC.

### Recomendação 10.3

Nos pacientes submetidos a EC, protamina deve ser administrada de rotina antes do fechamento da incisão (IIa/B)^434^ .

Outra questão controvertida é a drenagem da incisão da EC. Metanálise de seis estudos prospectivos não demonstrou benefícios no uso rotineiro do dreno^435^ . Porém, em casos de sangramento persistente não corrigido pelo controle da HPA e pela protamina, o cirurgião deve usar seu julgamento clínico para decidir sobre o uso do dreno.

### Recomendação 10.4

Nos pacientes submetidos a EC, recomenda-se o uso seletivo de drenagem da incisão cirúrgica (IIa/C)^435^ .

### Infecção da incisão cirúrgica

Infecção da incisão cervical para EC é infrequente. Em grandes séries, a taxas de infecção são inferiores a 1%. Apesar da EC ser classificada como cirurgia limpa, a administração de antibióticos é prática corrente, justificada pelo fato de ser cirurgia arterial com uso de remendo de material sintético ou biológico.

### Contaminação do remendo

Existem fortes evidências que no fechamento da arteriotomia na carótida um remendo (*patch*) deve ser usado de rotina^261^ . O material do remendo pode ser veia autóloga, lâminas de tecido sintético (dácron ou PTFE) e pericárdio bovino. Infecção com contaminação do remendo ocorre em 0,5-1% das operações^436^ . A infecção pode ser precoce, com o aparecimento de sinais inflamatórios na região operada, formação de abscesso e drenagem de pus. Mais comumente, a infecção tem apresentação crônica, com sinais inflamatórios discretos e a formação de uma fístula cutânea na incisão, com drenagem intermitente de secreção purulenta. A infecção contaminando o remendo pode terminar na formação de pseudoaneurisma e ruptura catastrófica da carótida^436^ . O tratamento da contaminação de remendo consiste na reoperação, com ressecção do segmento que contém o remendo e reconstrução da carótida interna com derivação de veia safena ou de artéria autógena^437,438^ .

Em pacientes de alto risco cirúrgico e/ou com pseudoaneurismas infectados, tem sido proposta a técnica EndoVAC^439^ . Esta técnica de exceção consiste em: 1. implantação de *stent* revestido no segmento infectado; 2. desbridamento cirúrgico da região infectada e remoção do remendo, sem clampeamento da carótida; 3. terapia a vácuo para permitir a granulação da ferida e cicatrização por segunda intenção e administração de antibióticos por longo prazo.

### Recomendação 10.5

Nos pacientes com contaminação de remendo, recomenda-se reoperação com ressecção do segmento que contém o remendo contaminado e reconstrução com enxerto autólogo venoso (veia safena) ou artéria autógena (IIa/B)^436-440^ .

### Restenose

No seguimento dos pacientes submetidos a EC, por vezes é detectada uma nova estenose na carótida operada. A incidência depende dos critérios usados para se definir restenose; em grandes séries, a taxa de restenose varia de 3-12%^240^ . Os mecanismos de restenose são a hiperplasia intimal no sítio da endarterectomia, lesão pelo clampeamento e, tardiamente, pela recidiva da aterosclerose da carótida^284^ . As restenoses são geralmente assintomáticas e o risco de novo evento isquêmico atribuível a elas é baixo^240,284^ .

Restenoses devem ser acompanhadas com exames periódicos de eDc. Se houver aumento progressivo do grau de estenose para > 70%, deve-se considerar a intervenção eletiva: nova EC ou ACS. A decisão deve ser tomada de preferência por uma equipe multidisciplinar, levando-se em conta a idade, as comorbidades, o risco cirúrgico e a *expertise* da equipe nas duas opções de tratamento.

Ocasionalmente, pacientes em seguimento pós-operatório desenvolvem evento isquêmico (AIT ou AVC), com sintomas correspondentes à carótida operada. Exames de imagem devem confirmar a presença de restenose. Se o grau de estenose é < 50%, o paciente deve ser mantido em TCO e acompanhado de perto; se o grau de estenose for > 50%, deve ser avaliado para nova EC ou para ACS^441,442^ .

### Recomendação 10.6

Em pacientes com eventos isquêmicos tardios e restenose > 50% ipsilateral aos eventos, recomenda-se refazer a EC ou ACS (I/B)^441,442^ .

### Complicações vasculares

#### Isquemia intraoperatória por *déficit* de perfusão

O clampeamento da carótida necessário para a EC provoca queda abrupta da perfusão cerebral. Nos pacientes com um círculo de Willis completo, a circulação colateral pela carótida contralateral e pelas AV mantém fluxo sanguíneo suficiente para manter a perfusão cerebral adequada durante o clampeamento da carótida. No entanto, em cerca de 10% dos casos, a circulação colateral não é adequada para manter a perfusão do hemisfério irrigado^442^ . Nestes casos, deve-se inserir um *shunt* para evitar o AVCI. O *shunt* pode provocar complicações, como dissecção da carótida, trombose com embolização distal e ruptura da carótida interna. Nos pacientes operados sob BLC, a decisão de inserir o *shunt* é baseada no *status* neurológico do paciente desperto. Nos pacientes operados sob AG, são necessários métodos de monitorização da perfusão cerebral durante o clampeamento. Estes métodos incluem: medida da pressão retrógada da carótida interna; eletroencefalograma; potencial evocado cerebral; eco-Doppler transcraniano; oximetria cerebral transcraniana; e espectroscopia infravermelho próximo^443^ .

Existe uma vasta literatura sob o uso dos métodos de monitorização na decisão de se inserir o *shunt*. Uma revisão Cochrane^444^ e uma análise da SVS-VQI^260^ não mostraram diferença entre os diversos métodos de monitorização e nem mesmo diferença entre o uso do *shunt* seletivo ou de rotina^258^ .

As Diretrizes europeias de 2021^103^ e a estadunidense de 2022^9^ concordam que não há diferenças estatísticas entre o uso rotineiro, uso seletivo e não-uso do *shunt* nos resultados da EC.

#### Recomendação 10.7

Nos pacientes submetidos a EC, a decisão de usar ou não um *shunt* deve ficar a critério do cirurgião (IIa/C)^9,103,258,260,444^ .

#### Embolização intraoperatória

Embolização cerebral pode ocorrer em qualquer etapa da EC. Em pacientes com placas instáveis, a própria dissecção das carótidas pode desencadear embolia cerebral, conforme mostram estudos de monitorização intraoperatória com eco-Doppler transcraniano^445^ . Embolização pode ocorrer durante a inserção do *shunt* ou através deste no decurso da operação^446^ . A etapa onde ocorre a maior carga embólica é no desclampeamento das carótidas^447^ .

Medidas para reduzir a probabilidade de embolização intraoperatória incluem: dissecção delicada das carótidas; heparinização plena; usar o *shunt* apenas quando necessário; remoção completa da placa de ateroma, sem deixar fragmentos no leito da endarterectomia; eliminação de potenciais detritos ao exterior antes do encerramento da arteriotomia e desclampeamento sequencial, dirigindo o fluxo anterógrado inicialmente para a carótida externa, antes de liberar o fluxo para a carótida interna.

#### Trombose per e pós-operatória da carótida interna de endarterectomia

A trombose da carótida durante ou imediatamente após a EC ocorre em cerca de 3% das operações^448^ . Causas da trombose são: falhas técnicas no decurso da endarterectomia, estenose da carótida interna por placa residual, defeitos na arteriorrafia, dissecções da carótida interna distal, heparinização inadequada e trombofilia com resistência à heparina^449^ .

O manejo da trombose perioperatória consiste na imediata detecção e correção da causa da obstrução. A suspeita pode vir da ausência de pulso na ACI, ausência de fluxo com o Doppler intraoperatório ou evidência de déficit neurológico ao despertar, nos casos em que se empregou AG. O paciente não deve deixar a sala de operações sem antes despertar. Estenose da carótida interna distal por placas pode ser tratadas com implante de *stents* coronarianos (geralmente disponíveis em todos hospitais terciários), na ACI distal; falhas técnicas na endarterectomia podem ser tratadas com a complementação da mesma (no caso de placas residuais) ou por substituição do segmento afetado por prótese de PTFe; defeitos na arteriorrafia geralmente demandam uma angioplastia com *patch*; dissecções distais da ACI e craniais à linha de Blaisdell usualmente requerem o implante de um *stent*, como acima relatado. Distúrbios de coagulação devem ser corrigidos com heparinização adequada, controlada pelo tempo de coagulação ativada. Eventualmente, é necessário um estudo angiográfico intraoperatório da carótida operada, o que pode ser obtido com a punção da ACC com um simples *scalp*, que será substituído por uma bainha 5F em caso de necessidade de intervenção^5,9,448-451^ .

Na ocorrência de um déficit neurológico pós-operatório precoce com o paciente já no CTI, o tempo sugere a etiologia. Até 6 horas após a cirurgia, a principal causa é trombose da ACI ou embolia de trombo mural da zona endarterectomizada. Após decorridas 6 horas, pode estar ocorrendo SHC, edema cerebral, hemorragia intracraniana ou até mesmo uma embolia. Até recentemente havia recomendação de exploração imediata nessas situações. Nos últimos anos, um Consenso europeu recente, ao qual outros trabalhos e diretrizes se seguiram, sugerem que em todo paciente que desenvolve um déficit neurológico nas primeiras horas do pós-operatório é preciso obter imagens rápidas da carótida, antes de reabordar o local operado^5,446,450^ .

Um exame de eDc pode ser realizado à beira-leito e, se houver evidências de obstrução da ACI, deve-se proceder imediatamente com a exploração do sítio operatório. Se houver adequada perviedade do eixo carotídeo operado, deve-se estudar rapidamente a circulação cervical e intracraniana com uma ATC^9,446,448^ . Este exame pode esclarecer se se trata de edema, SHC ou uma hemorragia intracraniana e uma trombose distal ou embolia. Trombos no segmento endarterectomizado são removíveis sob visão direta, e aqueles que se estenderam pela carótida interna devem ser retirados delicadamente com cateter de Fogarty 3F^9^ . Infusões de pequenas doses de trombolíticos podem ser úteis para dissolver trombos/êmbolos que não são acessíveis mecanicamente. Dose iniciais de 3 a 5 mg, podendo chegar até a 20 mg têm sido recomendadas, sempre com acompanhamento angiográfico intraoperatório. Em centros que dispõem de especialistas em neurointervenção, sugere-se o uso de trombolíticos por cateter diretamente na carótida interna distal e na ACM^451^ . Deve ser considerada revisão da arteriorrafia, com colocação de *patch* (de preferência, venoso) e manutenção de heparina em doses terapêuticas. Alguns autores sugerem o uso de Dextran 40 (não disponível no Brasil no momento) por infusão contínua nas 24 horas seguintes^215^ .

### Recomendação 10.8.1

Em pacientes nos quais é detectado déficit neurológico central ao final da EC, deve-se corrigir a causa do déficit imediatamente, antes de o paciente deixar o centro cirúrgico (I/B)^5,9,215,448-451^ .

### Recomendação 10.8.2

Pacientes que desenvolvem déficit neurológico horas após um despertar normal de EC devem ser estudados com eDc à beira do leito. Se detectada oclusão do segmento operado, o paciente deve ser imediatamente reoperado, com correção da causa da trombose. Se a carótida operada estiver pérvia, o paciente deve ser submetido a estudo imediato por angiotomografia, o que deverá determinar a conduta terapêutica (I/C)^5,9,215,448-451^ .

Uma prática que reduz significativamente o risco de trombose aguda, bem como da restenose tardia, é o uso rotineiro de remendo no fechamento da arteriotomia^262,452,453^ . A alternativa à EC convencional com remendo é a EC por eversão. Esta técnica reduz significativamente o risco de trombose aguda e de restenose da carótida^263^ .

### Recomendação 10.9

Nos pacientes submetidos a EC, recomenda-se o fechamento da arteriotomia com remendo, e não o fechamento primário. Uma alternativa é a endarterectomia de eversão (I/A)^262,263,452,453^ .

### Complicações neurológicas

#### Acidente vascular cerebral isquêmico

O AVCI no perioperatório da EC pode ser causado por isquemia cerebral durante o clampeamento, por embolização de fragmentos da placa ou trombos durante ou imediatamente após o ato cirúrgico, e por trombose do sítio da endarterectomia na carótida interna.

As medidas que reduzem o risco de AVCI são: controle rigoroso da PA no intra e pós-operatório, uso seletivo do *shunt*; heparinização adequada e uso rotineiro do remendo no fechamento da arteriotomia ou a técnica de EC por eversão^5,9,260,262,443,444,452-454^ .

#### Síndrome de hiperperfusão cerebral

A SHC é uma complicação infrequente, mas grave, do tratamento invasivo da doença carótida por EC ou ACS^263^ . O principal fator de risco para SHC é a elevação persistente da PA no intra ou no pós-operatório imediato. Fatores adjuvantes são: déficit crônico de perfusão cerebral por oclusão ou estenose crítica da carótida contralateral; infarto cerebral recente^455-457^ . O risco de SCH é muito mais alto nos pacientes submetidos à ACS do que a EC^458^ . Estudos prospectivos mostram que a SHC pode ser minimizada pela monitorização e controle agressivo da PA durante a EC e no pós-operatório imediato (24-48 horas)^459,460^ .

#### Hemorragia intracerebral

A HIC é a complicação mais grave da EC, com mortalidade > 50% dos casos^236^ . HIC é causada por HAS descontrolada, pela transformação hemorrágica de um infarto cerebral recente, ou como uma complicação da SHC. HIC ocorre com muito mais frequência em pacientes submetidos à ACS do que a EC^461,462^ .

O manejo consiste no controle estrito da PA, ventilação assistida e monitorização da pressão intracraniana. Está indicada consulta à equipe de neurocirurgia para avaliação e medida da pressão intracraniana e possível tratamento cirúrgico do hematoma cerebral e descompressão craniana.

#### Complicações do tratamento endovascular das carótidas

Grande parte das complicações foram descritas nas seções anteriores e são relacionadas à escolha do acesso, tipo de *stent*, constituição e aspecto anatômico das placas^308^ . O Conselho de Especialistas do National Institute for Health and Care Excellence (NICE), do Reino Unido, listou os eventos adversos conhecidos para o tratamento ACS, sendo que a trombose, embolia, pseudoaneurisma ou ruptura arterial podem estar relacionadas tanto ao local de acesso como à artéria carótida. Em relação às complicações sistêmicas, associam as reações ao material de contraste (alergias e insuficiência renal induzida) e neoplasia secundária a radiação^279^ .

O declínio da memória verbal é outra complicação que pode estar presente na evolução pós-operatória de angioplastia carotídea (1 mês pós-intervenção, p = 0,039). Enquanto pacientes com idade inferior a 80 anos, doença carotídea aterosclerótica grave e escores de memória episódica de linha de base baixos se beneficiam da intervenção carotídea (especificamente EC), indivíduos ≥ 80 anos apresentam piora da memória relacionada à intervenção (1 mês, p = 0,046; e 6 meses, p = 0,043), sendo o implante de *stent* na artéria carótida um preditor independente de piora da memória verbal^463^ .

#### Pós-operatório de angioplastia carotídea

Instabilidade hemodinâmica necessitando de suporte com vasopressores ocorre em 19% das ACSs. Em uma metanálise (27 estudos, n = 4.204), 12% dos pacientes com ACS foram tratados por hipotensão, 12% por bradicardia, e 13% tiveram tratamento para ambas. Houve associação significativa entre hipotensão persistente após ACS e história de EC ipsilateral, calcificação com envolvimento do bulbo carotídeo, estenose mais significativa e placa excêntrica^464^ .

A prevenção da instabilidade hemodinâmica durante a ACS envolve hidratação, suspensão dos anti-hipertensivos (dependentes de protocolos específicos em conjunto com o grupo anestésico e da unidade de tratamento intensivo) e monitoramento contínuo, em tempo real, da ecocardiografia e da PA. O glicopirrolato (derivado sintético da atropina) foi comparado com a atropina em um estudo retrospectivo (n = 115), sendo mais eficaz na prevenção de bradicardia pós-operatória (30% *versus* 72%, p = 0,002) e hipotensão (2,5% *versus* 36%, p = 0,001), com taxas mais baixas de hipertensão compensatória (2,5% *versus* 16%, p = 0,047)^464^ . A hipotensão está relacionada à diminuição da resistência vascular com perda do tônus simpático, e não por hipovolemia. Portanto, o uso de vasopressores (norepinefrina, dobutamina, fenilefrina) pode ser necessário para manter a PAS > 90 mmHg^465^ .

A utilização de acesso invasivo para o controle pressórico e a utilização de punção de acesso venoso profundo para o tratamento da hipotensão são medidas que facilitam o acompanhamento e condução destes pacientes, mas dependem de protocolos individuais de cada serviço, definindo o local do acesso e os casos nos quais deverão ser realizados^464,465^ .

### Recomendação 10.10.1

A suspensão do uso de betabloqueadores nos dias que antecedem a ACS deve ser definida por protocolo da equipe anestésica e cirúrgica. Painel de especialistas^463^ .

### Recomendação 10.10.2

A suspensão do tratamento anti-hipertensivo após ACS deve ser definida por protocolo da equipe da terapia intensiva e cirúrgica. Painel de especialistas^463^ .

### Recomendação 10.11

O acesso invasivo para controle pressórico e o acesso venoso profundo durante e após a ACS para o tratamento da hipotensão devem ser definidos por protocolo da equipe cirúrgica. Painel de especialistas^463,464^ .

## LISTA DE ABREVIAÇÕES

AAS Ácido acetilsalicílico

AAP Antiagregação plaquetária/antiagregantes plaquetários

ACAS Asymptomatic Carotid Artery Study

ACI Artéria carótida interna

ACM Artéria cerebral média

ACO Anticoagulação oral

ACRS Asymptomatic Carotid Stenosis and Risk of Stroke Study

ACS Angioplastia carotídea com *stent*

ACST-1 Asymptomatic Carotid Surgery Trial

ACTI Asymptomatic Carotid Surgery Trial

ACTRIS Endarterectomy combined with optimal medical therapy (OMT) vs OMT alone in patients with asymptomatic severe atherosclerotic carotid artery stenosis at higher-than-average risk of ipsilateral stroke

AG Anestesia geral

AHA American Heart Association

AITc Ataque isquêmico transitório em *crescendo*

AITs Acidentes isquêmicos transitórios

ALR Anestesia locorregional

ARC-EC Alto risco para endarterectomia de carótidas

ARM Angiorressonância magnética

ATC Angiotomografia computadorizada

AV Artéria vertebral

AVC Acidente vascular cerebral

AVCe Acidente vascular cerebral em evolução ou flutuante

AVCH Acidente vascular cerebral hemorrágico

AVCI Acidente vascular cerebral isquêmico

AVK Antivitamina K (anticoagulante e varfarina, por exemplo)

BLC Bloqueio locorregional cervical

CREST Carotid Revascularization Endarterectomy vs Stenting Trial

CRM Cirurgia de revascularização do miocárdio

DAC Doença arterial coronariana

DAP Doença arterial periférica

DM Diabetes melito

DPCA Dispositivos de proteção cerebral antiembólica

DPOC Doença pulmonar obstrutiva crônica

DTAT Dupla terapia antitrombótica (antiagregantes + DOAC)

EAM Eventos adversos maiores – acidente vascular cerebral e morte

EC Endarterectomia de carótida

ECST European Carotid Surgery trial

eDc eco-Doppler colorido

EPR Estudo prospectivo randomizado

eRm Escala de Rankin modificada

ESVS European Society for Vascular Surgery

FA Fibrilação atrial

HAS Hipertensão arterial sistêmica

HBPM Heparina de baixo peso molecular

HIC Hemorragia intracerebral

HMG-CoA 3-hidroxi-3-methyl-glutaril-CoA redutase

IAM Infarto agudo do miocárdio

IBP Inibidores de bomba de prótons

ICC Insuficiência cardíaca congestiva

IVB Insuficiência vertebrobasilar

LDL-c LDL-colesterol (*low-density lipoprotein cholesterol*)

NICE National Institute for Health and Care Excellence

NASCET North American Symptomatic Carotid Artery Trial

NIHSS Escala de Isquemia Cerebral do Instituto Norte-Americano de Saúde

NNT Número de pacientes que precisam ser tratados para beneficiar um paciente

OCT Tomografia de coerência ótica

OMS Organização Mundial da Saúde

PA Pressão arterial

PCSK9 Pro-proteína convertase subtilisina-kexina tipo 9

PO Pós-operatório

PTFe Politetrafluoroetileno expandido

RCT Estudos multicêntricos randomizados e controlados

RM Ressonância magnética

RM-dif Ressonância magnética com estudo de difusão

RR Redução de risco

SHC Síndrome de hiperperfusão cerebral

SVS Society for Vascular Surgery

TE Tratamento endovascular

TC Tomografia computadorizada

TCAR Angioplastia transcervical da carótida com *stent*

TCO Tratamento clínico otimizado

TCP Tomografia computadorizada de perfusão

TE Tratamento endovascular

TSA Troncos supra-aórticos

VACS Veterans Affairs Cooperative Study

VB Vertebrobasilar

VDF Velocidade diastólica final

VPS Velocidade de pico sistólico

VQI Vascular Quality Initiative da Society for Vascular Surgery
